# A methodology for the design of experiments in computational intelligence with multiple regression models

**DOI:** 10.7717/peerj.2721

**Published:** 2016-12-01

**Authors:** Carlos Fernandez-Lozano, Marcos Gestal, Cristian R. Munteanu, Julian Dorado, Alejandro Pazos

**Affiliations:** 1 Information and Communications Technologies Department, University of A Coruna, A Coruña, Spain; 2 Complexo Hospitalario Universitario de A Coruña (CHUAC), Instituto de Investigacion Biomedica de A Coruña (INIBIC), A Coruña, Spain

**Keywords:** Computational intelligence, Machine learning, Methodology, Statistical analysis, Experimental design, RRregrs

## Abstract

The design of experiments and the validation of the results achieved with them are vital in any research study. This paper focuses on the use of different Machine Learning approaches for regression tasks in the field of Computational Intelligence and especially on a correct comparison between the different results provided for different methods, as those techniques are complex systems that require further study to be fully understood. A methodology commonly accepted in Computational intelligence is implemented in an R package called RRegrs. This package includes ten simple and complex regression models to carry out predictive modeling using Machine Learning and well-known regression algorithms. The framework for experimental design presented herein is evaluated and validated against RRegrs. Our results are different for three out of five state-of-the-art simple datasets and it can be stated that the selection of the best model according to our proposal is statistically significant and relevant. It is of relevance to use a statistical approach to indicate whether the differences are statistically significant using this kind of algorithms. Furthermore, our results with three real complex datasets report different best models than with the previously published methodology. Our final goal is to provide a complete methodology for the use of different steps in order to compare the results obtained in Computational Intelligence problems, as well as from other fields, such as for bioinformatics, cheminformatics, etc., given that our proposal is open and modifiable.

## Introduction

Experimental Design (ED) in Computational Intelligence (CI) is one of the most important aspects in every research process, thus it is crucial to correctly define all the steps that should be taken to ensure obtaining good results. An incorrect ED or an incorrect definition of one of its steps can lead to making a wrong choice of the best method to solve the involved problem. Indeed, available data in cheminformatics have been shown to include multiple flawed structures (up to 10%) due to poor experimental design and pre-processing of the data (Gilad, Nadassy & Senderowitz, 2015).

Moreover, nowadays we are living in an era of publicly available information, open databases, and open data, especially when considering that the availability of datasets in public domain has skyrocketed in recent years. There seem to be no commonly accepted guidance or set of procedures for data preparation (Fourches, Muratov & Tropsha, 2010).

This work proposes a generic normalization of the framework for the ED to address this situation and defines the four phases that should be followed in any ED: dataset, pre-processing of data, learning and selection of the best model. These phases include the operations or steps that any researcher should follow to get reproducible and comparable results in their research studies with either state-of-the-art approaches or other researchers’ results. It is of extreme importance to avoid model oversimplification and to include a statistical external validation process of the model in order to generate reliable models (Tropsha, 2010), not only aimed at searching for differences within different experimental runs. All phases proposed in the experimental design are important, but the final selection phase of the best model is where errors or variations to our proposal may occur, or simply our recommendations may not be taken into account. For this reason, the proposed methodology pays particular attention to this point, providing a robust statistical guidelines to ensure the reproducibility of the results, and proposes some improvements or modifications to the previously published methodology in order to achieve the ultimate objective, that is, reliability in *in silico* prediction models.

The framework is obviously not a fixed workflow of the different phases, because it should be adaptable to different fields, each of them with different and distinctive internal steps. Thus, this is a general proposal that can be taken as a good working practice, valid for any type of experimentation where machine learning algorithms are involved. The methodology proposed in this work is checked against an integrated framework developed in order to create and compare multiple regression models mainly in, but not limited to, cheminformatics, called RRegrs (Tsiliki et al., 2015a). This framework implements a well-known and accepted methodology in the cheminformatics area in form of an R package.

Focusing on the last step of our proposal, the best regression model in this methodology is selected based on the next criterion: the authors evaluate the performance of all models (with the test set) according to R-squared (*R*^2^) and establish a ranking. Next, they take into consideration all the models which are in the range (±0.05) according to this measure, and reorder the ranking choosing the one with the lowest Root Mean Squared Error (RMSE) obtained in test. Thus, they find the best model of all the runs, combining the use of two performance measures with a particular criterion. This package also allows using an *adjusted R*^2^ in order to select the best model. This criterion, to the best of our knowledge, is not the most accurate when dealing with Machine Learning (ML) algorithms.

The present work presents a conceptual framework for experimental designs, which pay special attention to the phase where the best model is selected, using a more statistical-based approach. This new approach provides a more robust, stable and reproducible way to perform an Experimental Design. Scientific irreproducibility is a major and growing concern (Baker, 2016b) as it was found that of more than 1,500 researchers, 87% named poor experimental design as the major cause of reproducibility and also, a very high 89% detect flaws in statistical analysis (Baker, 2016a).

This proposal is tested against five of the most well-known and simple datasets used for standard regression from the UC Irvine machine learning repository (Lichman, 2015) and three real Cheminformatics datasets. Finally, the results of these tests are compared against the results obtained using the methodology proposed by RRegrs.

The aim of this study is to present a set of guidelines for performing multivariate analysis in order to achieve statistically sound machine learning models for an accurate comparison of different results obtained by these methods. Furthermore, another objective is to draw up a comprehensive methodology and support for predictive in silico modeling. The current paper is organized as follows: the Methods section describes the methodology and the particular modifications proposed in the different steps: dataset, data pre-processing, model learning and best model selection; the Results section includes a comparison with the RRegrs machine learning algorithms and five state-of-the-art standard datasets, and an experimental analysis of the performance of the proposed methodology against those previously published; finally, the Discussion and the Conclusions sections are presented.

## Methods

### Proposed methodology

This paper proposes a normalization of experimental designs for computational intelligence problems, such as those from cheminformatics or bioinformatics, as well as from all related disciplines where it is necessary to select the best ML model. In order to evaluate our methodology, a well-known methodology implemented in an R package was used to automate predictive modeling in regression problems. In Tsiliki et al. (2015a) and Tsiliki et al. (2015b), authors observed that there was a need for standardization of methodologies in different parts of the analysis: data splitting, cross-validation methods, specific regression parameters and best model criteria.

**Figure 1:**
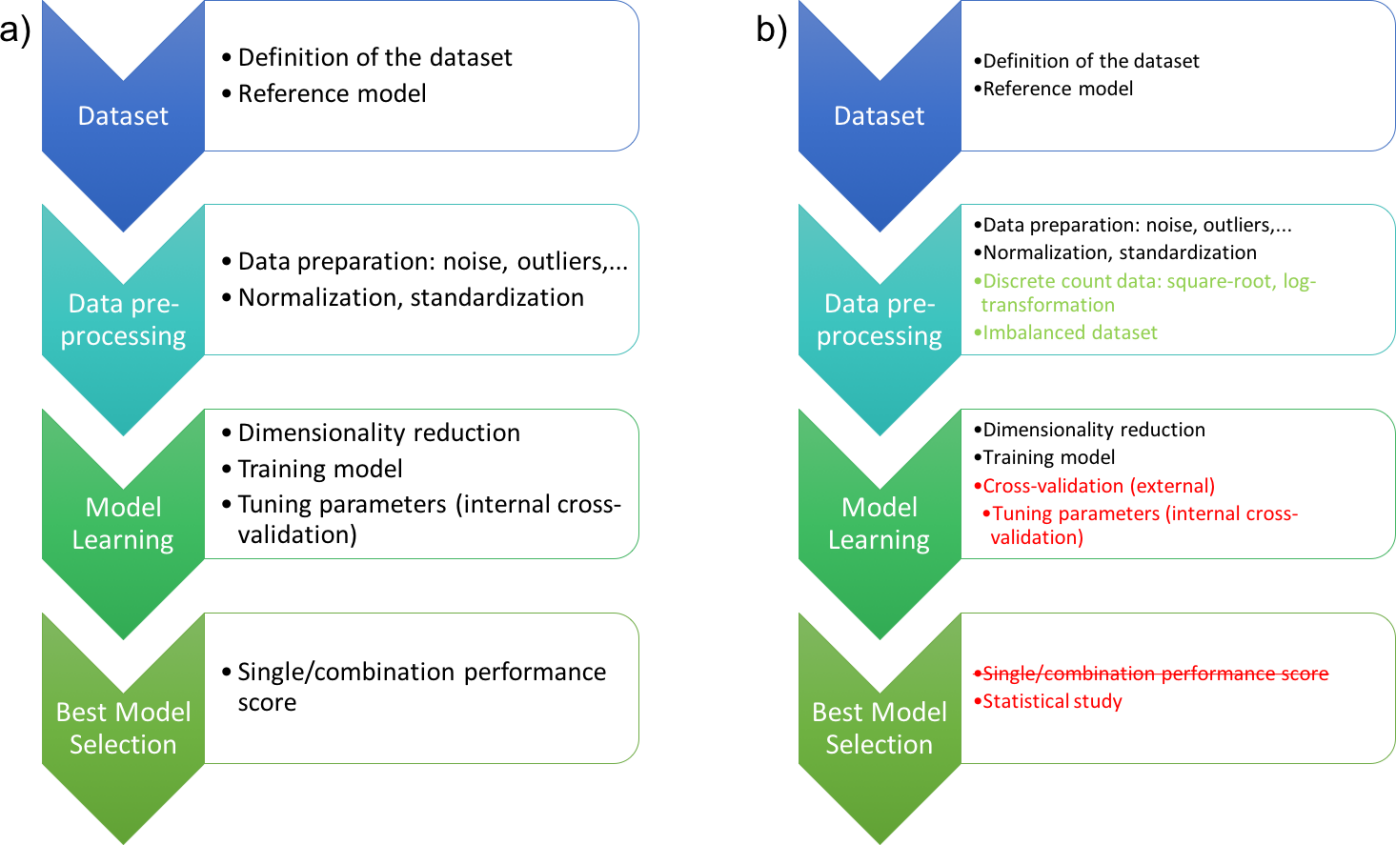
(A) shows the workflow of the experimental design in computational intelligence previously published in the literature and (B) details the phases where methodological changes are proposed to ensure that the performance of the machine learning models is not biased and that the resulted models are the best.

Their need for normalization was formalized based on the definition of a workflow that contains the following phases in order to clearly state where the differences are located within our proposal: dataset, pre-processing of data, learning and selection of the best model, which are graphically represented in Fig. 1A. The following paragraphs describe more in depth each of them.

It is important to note that this methodology uses ML algorithms in order to solve regression problems and consequently, it is a universal methodology. Unfortunately, despite the ability of those techniques to solve real-world problems, they also have drawbacks and obviously particular limitations that should be taken into account when used. More precisely, the methodology proposed by Tsiliki et al. (2015a) does not take into account that the performance of ML techniques is directly related to the observations used for training the models. Thus, a statistical analysis of the variability and stability of the techniques is essential within different runs and different initial seeds to separate the data. Moreover, cross-validation is necessary not only to select the best parameters (internal tuning phase) for each technique as proposed, but also externally, to ensure that the training of the model is not biased or flawed as shown in Fig. 1B. There is also a minor consideration about the pre-processing of the data that arise when machine learning models are applied: how to deal with count data and imbalanced datasets.

#### Dataset

Firstly, the dataset should be generated, defining its particular characteristics. The definition must contain the variables involved in the study and a brief description of each of them to ensure the reproducibility of the tests by external researchers. In order to ensure that the data are representative enough for the particular problem under study, the help of experts is needed to define the cases (i.e., regions of interest for medical imaging or case-control patients).

In this work, five standard regression datasets from the UCI Machine Learning Repository were used: housing, computer hardware, wine quality, automobile and Parkinson’s disease telemonitoring datasets. Non-numeric columns were eliminated. For further information (number of cases, number of features, etc.) please refer to the UCI Machine Learning official website (Lichman, 2015). Finally, once our methodology provided satisfactory results using simple and well-known toy datasets, it was decided to increase the difficulty by studying three real datasets in order to compare not only the results, but also the best models.

#### Data pre-processing

After the generation of the dataset, data are in a raw or pure state. Raw data are often difficult to analyze, thus they usually require a preliminary study or a pre-processing stage. This study will check that there are no data with incomplete information, outliers or noise. In case that some of the aforementioned situations are present in the dataset, different approaches should be applied to avoid them. Only once this process is finished, it is considered that data are ready for analysis. To understand the importance of this step, it is often said that 80% of the effort of a data analysis, is spent compiling data correctly for analysis (Dasu & Johnson, 2003).

Furthermore, the variables typically present different scales or sizes, making them difficult to compare in equality of conditions. Thus, normalization or standardization techniques are required to make data comparable. Both techniques have certainly drawbacks and none is better than the other. Moreover, the dataset should be studied for each particular problem before applying it. For example, if there is an attempt of performing a normalization step and there are outliers in the data (not previously removed), this step will scale useful data to a small interval. This is a non-desirable behavior. After a normalization step, data are scaled in the range [0,1] in case of numeric values. In case a standardization process was performed, data present a zero average value and a standard deviation equal to one, thus they are independent of the unit of measure. Depending of the type of data, there are other well-known approaches to minimize the influence of the values (raising a feature to a power, taking the logarithm, etc.). In the case of discrete count data, the use of the square-root transformation is recommended in order to normalize count data and then log transform them for subsequent analysis (Cuesta et al., 2008). However, recent works have suggested that further studies are necessary in order to choose the best technique to deal with the original data without transformations (O’Hara & Kotze, 2010), rather than the pre-processing of these data.

Another relevant point when using machine learning methods is how to cope with imbalanced datasets. There are mainly two different approaches to deal with those datasets, oversampling (creating synthetic examples from the minority class), as proposed by Chawla et al. (2002) and undersampling (removing samples from the majority class) of the data, as proposed by Seiffert et al. (2010) for balancing purposes.

In this work, the NA (Not Available) values and the NZV (near zero variance) features were removed from the datasets at the first stage. Next, a study about the correlation among variables was performed and the correlated variables with a cutoff higher than 0.90 were removed from the dataset. Finally, a standardization phase was applied in order to center and re-scale data, including the output variables, so that they could have a zero mean value and a standard deviation equal to one.

#### Model mearning

This may be the most important step in computational intelligence. First of all, a reference model is needed to check the results achieved for a proposed model or technique. This reference model can be extracted from a literature study of the field (state-of-the-art model) or constructed from a set of standard data (*gold standards* or *ground truth*), for example. In both cases, the results obtained from this reference model will be the ground truth, along with the following experimental design.

Once the reference model is established, it is time to build and test the model intended to be developed in order to provide better solutions. The range of techniques available to solve any problem is usually very high. Some of these techniques are dependent on the field of study, consequently the researcher should review the state-of-the-art in their research field in order to choose the most suitable method.

Some key points arise at this time, such as the need for measuring the performance that should clearly indicate how well the techniques have performed during this training phase. There are different well-known performance scores, such as Area Under Receiver Operating Characteristic (AUROC), accuracy or F-measure in classification problems or Mean Square Error (MSE), RMSE or *R*^2^ in regression problems. However, sometimes it is necessary to evaluate the performance of the model using an ad-hoc measure. Furthermore, the over-training and over-fitting of those techniques should be avoided in order to ensure that the obtained model provides good results using unknown data. Techniques such as *cross-validation* (CV) (McLachlan, Do & Ambroise, 2005) can be useful in this case.

There are two main aims when using CV during the model learning process. Firstly, CV was used to measure the degree of generalization of the model during the training phase, assessing the particular performance of the model and estimating the performance with unknown data. Secondly, using a CV approach, a comparison was performed betweeenvtwo or more algorithms that were trained under the same conditions and with the same dataset. Instead of running our experiments one time only, at least ten runs were performed and ten different initial seeds were used to separate the data for each dataset independently. Thus, as the CV error alone is not adequate as a decision value to compare a set of algorithms, relevant information is available regarding the behaviour of the models to discard bias related to the particular position of the observations within the file data. For each one of the ten runs, each original dataset was separated in ten random splits (75% training and 25% test) and the CV process was performed only with the training data. Once the model was finally trained, they remaining absolutely unknown 25% of the original data were used for validation purposes. However, the key point in this case is that they proposed this cross-validation process was proposed internally and only in order to select the best combination of parameters for each technique.

**Figure 2:**
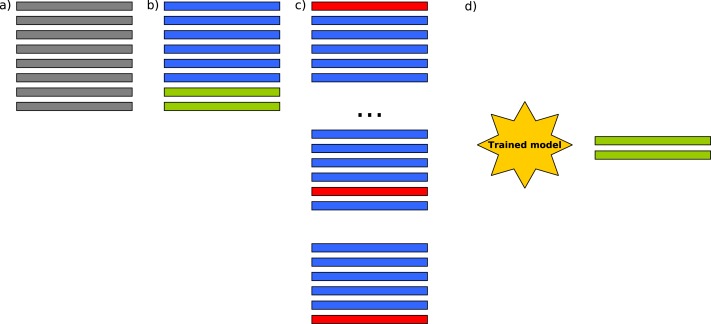
In (A) original data are separated in (B) 75% training and 25% test. A 10-fold cross-validation is run in (C) in order to train the machine learning models, and finally in (D) *R*^2^ and RMSE are evaluated with the unknown 25% of the data.

The aforementioned CV process was improved by adding an additional previous step with the formalization of an external CV process to ensure that the models were not over-trained and that the best performance score was not found for a particular combination of parameters/observations. By doing so, a flawed experimental design was avoided, due to the particular distribution of the observations in the dataset. Thus, within each fold, the internal CV process was maintained for the parameter selection proposed initially by the authors, but it was ensured that an external set of observations was unknown for the model and that during this tuning process over-training was avoided, or at least, it could be detected in order to discard these results. Thus, we ensure that we are avoiding a possible selection bias with an external cross-validation to the feature selection process. Furthermore, this is also necessary when the number of observations is much lower than the number of features Ambroise & McLachlan (2002). The process shown in Fig. 2, is repeated ten times for each ML technique/dataset. The final performance measure reported is in this case the average of the values computed for all folds and splits. RMSE, *R*^2^ and adjusted *R*^2^ were computed. Our methodology modified the proposal made by Tsiliki et al. (2015a) by adding this external cross-validation step in Fig. 2C in order to ensure that, in each one of the folds, an internal cross-validation was performed to select the best set of parameters as proposed, but no bias or overfitting (model fitting the data) were possible, as independent data were used for validation in each fold.

The RMSE is the square root of the variance of the residuals and has the same units as the response variable. Furthermore, it is an absolute measure that indicates the fit of the model to the data. Finally, this measure is useful to check the ability of the models to predict values for the dependent variable in an absolute sense. The coefficient of determination *R*^2^ is in the range from zero to one, the closer the coefficient to one, the better the results. However, this criterion is not appropriate for the comparison of candidate models because overfitting increases artificially its value (Bontempi & Ben Taieb, 2011), as well as the number of features. Finally, this measure is useful to check how well the independent variables explain the variability of the dependent one. The adjusted coefficient of determination incorporates the degrees of freedom of the model in order to avoid the aforementioned problems. It is interpreted as the proportion of total variance, which is explained properly by the model.

Finally, the dimensionality of data should be taken into account. The bigger the dimensionality of the input data, the higher is the number of examples necessary for learning. Moreover, the techniques for dimensionality reduction are usually significant (Saeys, Inza & Larrañaga, 2007) for providing the best possible model (Donoho, 2000) with the lowest dimensionality. Thus, these techniques allow for a complexity reduction of the generated model. Furthermore, they also imply a reduction of the time needed and improve the overall capacity of the system.

Ten linear and non-linear regression models included in the RRegrs package were employed in this work: Multiple Linear Regression (lm) (Kutner, Nachtsheim & Neter, 2004), Generalized Linear Model with Stepwise Feature Selection (glmStepAIC) (Hocking, 1976), Partial Least Squares Regression (PLS) (Wold et al., 1984), Lasso regression (Lasso.RMSE) (Tibshirani, 1994), Elastic Net regression (glmnet) (Zou & Hastie, 2005), Support Vector Machines using radial functions (svmRadial) (Guyon et al., 2002), Neural Networks regression (nnet) (Bishop, 1995), Random Forest (RF) (Breiman, 2001), Random Forest Recursive Feature Elimination (rfRFE) (Saeys, Inza & Larrañaga, 2007) and Support Vector Machines Recursive Feature Elimination (svmRFE) (Dobson & Barnett, 2008). More specifically, glmStepAIC, Lasso.RMSE, svmRFE, rfRFE and glmnet performs dimensionality reduction.

The Supplementary materials include the results of all models with the state-of-the-art and the three real data datasets, as well as the original data files in csv format.

#### Best model selection

In the previous phase, it has been stated that there were several measures accepted and well known for measuring the performance of a classifier. This does not mean that a researcher is able to compare different classifiers used in the same conditions, using the same dataset with only one run and one error rate. At this point, each technique should be run several times in order to ensure that our results are not biased because of the distribution of the observations within the data or that the number of internal CV runs performed in order to find the best combination of parameters do not bias our results (there is a high probability that a good combination of parameters/performance score is found for a particular distribution of observations when the number of experimental CV runs increases). Consequently, it is a good idea to take into account the mean and standard deviation of all the runs to evaluate the behavior.

With the results obtained by these techniques and in order to determine whether or not the performance of a particular technique is statistically better than that of the others, a null hypothesis test is needed. Furthermore, so that a parametric or a non-parametric test could be used, some required conditions must be checked: independence, normality and heteroscedasticity (García et al., 2010). Note that these assumptions do not refer to the dataset used as input to the techniques, but to the distribution of the performance of the techniques. In statistics, one event is independent of others if the fact that one occurs does not modify the probability of the others. Thus, in computational intelligence, it is obvious that different versions of a set of different algorithms, where initial seeds are used randomly for the separation of data in training and test, comply with the condition of independence. Normality is considered the behavior of an observation that follows a normal or Gaussian distribution; in order to check this condition, there is a different number of tests, such as the Kolmogorov–Smirnov or Shapiro–Wilk (Shapiro & Wilk, 1965). Finally, the violation of the hypothesis of equality of variances, or heteroscedasticity should be checked using, for example, a Levene’s or Bartlett’s tests (Bartlett, 1937).

As part of a good experimental design for an inter-technique comparison, a proper statistical test should be applied, based on the statistical distribution of the observed performance. Most of the computational intelligence comparisons in the literature just apply a *T*-test using the performance scores to check if a technique is significantly better than the others. In some cases, this distribution does not met the requirements of this parametric test, consequently a non-parametric test is required. Despite the fact that a parametric test is more powerful a priori, it should not be used when the conditions of independence, normality and homoscedasticity are not fully met. Instead, it is best to employ a non-parametric test, as it was designed specifically for these cases and the result will be more accurate and adjusted to the intrinsic characteristics of the data. Depending on the particular number of techniques that are used in the comparison, a different statistical test should be applied, some of the most common ones being shown in Table 1.

**Table 1:** The most commonly used parametric and non-parametric statistical tests, according to the number of techniques used in the comparison.

Number of techniques	Parametric	Non-parametric
Repeated measures, *n* = 2	*T*-test	Wilcoxon
Repeated measures, *n* > 2	ANOVA	Friedman, Quade

Despite the fact that the Friedman and Quade tests can be used under the same circumstances, in our methodology the use of the Friedman test was proposed with the Iman-Davenport extension as there are ten regression models to be compared and (García et al., 2010) stated that the Quade (1979) test performed better when the number of algorithms was low, at no more than 4 or 5.

Finally, after the null hypothesis test (parametric or non-parametric) was rejected, a *post hoc* procedure had to be used in order to address the multiple hypothesis testing and to correct the *p-values* with the *adjusted *p*-values* (APV). Among the different *post hoc* procedures (i.e., Bonferroni-Dunn, Holm, Hochberg, Hommel, Finner, etc.) the Finner procedure was chosen, as it is easy to understand and offers better results than the others, as stated in García et al. (2010).

**Figure 3:**
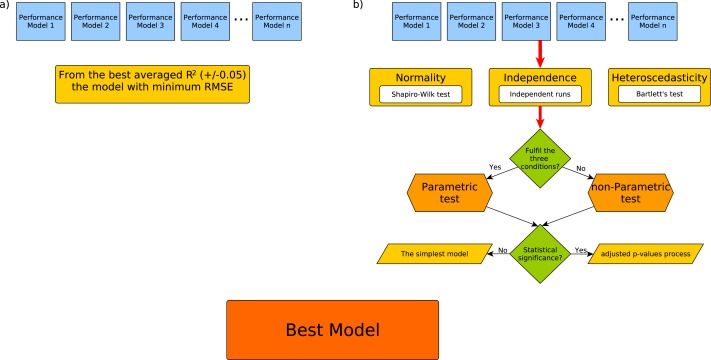
Workflow for the best model selection according to the (A) RRgres methodology and (B) our proposal.

Figure 3A includes a comparison between how the previously published methodology selected the best model under a criterion based on the ranking of the best averaged *R*^2^ and how the best model model was selected with the minimum RMSE from the top ones in the range (±0.05). On the other hand, Fig. 3B presents our proposal to select the best model according to a statistical criterion.

During this last phase, in the event that the results are not statistically significant, the simplest would be chosen from among the models that are winners of the null hypothesis test, either in terms of complexity or runtime. In a more strict sense, one could also perform a new null hypothesis test using only the results of the winning models, but in this case another performance measure should be employed, such as the number of features if a process of feature selection is carried out. Therefore, one could conclude that from among the initially selected winning models, there is a statistically significant difference according to this new second performance criterion (Fernandez-Lozano et al., 2016).

Finally, in order to ensure the power and validity of our results, the estimation of the differences was checked between each pair of machine learning techniques using the approach proposed in Doksum (1967), which is a contrast estimation based on medians aimed at assessing the real numeric difference between both methodologies.

## Results

### Preliminary results from UC Irvine Machine Learning repository datasets

Five of the most relevant datasets used for standard regression are used as the starting point to compare the proposed methodology against the previously described in Tsiliki et al. (2015a). The selected datasets were: Housing, MachineCPU, WineQuality, Automobile and Parkinson.

Table 2 shows *R*^2^ statistic values for these standard datasets and all the methods available in the package (winning model in bold for RRegrs). All experiments were run with RRegrs version 0.0.5 and following the same conditions as in Tsiliki et al. (2015a) with 10 different dataset splits, using 10-fold cross-validation and finally 10 runs with the best model and randomizing the response variable (Y-randomization) in order to ensure that no bias is included in the analysis (Tropsha, 2010).

**Table 2:** RRegs: *R*^2^ values for UCI Datasets (average for 10 splits).

	Housing	Machine CPU	Wine quality	Automobile	Parkinson
rfRFE	0.8765	0.8949	0.5008	**0.9149**	0.9003
RF	0.8744	0.9073	**0.5010**	0.9159	**0.9721**
svmRadial	**0.8444**	0.7655	0.3964	0.8546	0.6370
nnet	0.8349	**0.8771**	0.3693	0.8036	0.5449
svm-RFE	0.7277	0.6766	0.3796	0.6373	0.4793
glmStepAIC	0.7092	0.8249	0.3528	0.8236	0.1534
lm	0.7075	0.8218	0.3547	0.8237	0.1535
glmnet	0.7061	0.8250	0.3546	0.8279	0.1538
Lasso.RMSE	0.7045	0.8279	0.3538	0.8295	0.1538
PLS	0.6603	0.7932	0.3313	0.7839	0.1214

According to our methodology, the same winning model as in the methodology proposed by Tsiliki et al. (2015a) was selected, with the Housing and the Parkinson datasets. Thus, our methodology rejects the best model selected for three out of five datasets: MachineCPU (RF was selected instead of nnet as the best method), WineQuality (rfRFE instead of RF) and Automobile (RF instead of rfRFE). The following paragraphs present the statistical studies that support these differences.

In the first case, the results showed that for 10 different dataset splits, using 10-fold repeated CV and with the MachineCPU dataset, according to a Shapiro–Wilk test with the null hypothesis that the data follow a normal distribution, obtaining a *p-value* of 0.0004087 and *W* of 0.94525. Thus, the null hypothesis was rejected and our results did not follow a normal distribution. A Barlett’s test was performed with the null hypothesis that our results were heteroscedastic. The null hypothesis with a *p-value* of 0.01015 and a *k-squared* value of 21.623 was rejected. At this point, two conditions are not met; consequently, a non-parametric test should be employed, assuming the null hypothesis that all models have the same performance. The null hypothesis was rejected with the Friedman test and the Iman-Davenport extension (*p*-*value* < 1.4121E–10). Hence, at this point, the null hypothesis is rejected with a very high level of significance, showing that the winning model is RF.

The average rankings for the Machine CPU dataset, are shown in Table 3. This table also presents for each technique the test statistic of the Friedman test (z), the *unadjusted p-value* and the *adjusted p-value* with Finner’s procedure.

**Table 3:** Machine CPU dataset results using RF as the control model.

	Ranking	*z* = (*R*_0_ − *R*_*i*_)∕*SE*	*Unadjusted p-value*	*Adjusted p-value* (Finner)
RF	2.30	–	–	–
rfRFE	2.40	0.0738	0.9411	4.59E−6
**nnet**	3.39	0.8124	0.4165	5.52E−4
glmStepAIC	5.44	2.3264	0.0199	0.0020
Lasso.RMSE	5.60	2.4372	0.0148	0.0038
glmnet	5.80	2.5849	0.0097	0.0174
lm	6.55	3.1388	0.0016	0.0221
svmRadial	6.90	0.3973	6.80E−4	0.0256
PLS	7.50	3.8404	1.22E−4	0.4545
svm-RFE	9.10	5.0221	5.11E−7	0.9411

Tables 3–8 present the value of the ranking measure and the value of the test statistic as shown in Eq. (1) (Daniel, 1990) using the Friedman test, the *unadjusted p-value* and the corrected *p*-value using the Finner *post-hoc* procedure.

(1) \begin{eqnarray*}z= \frac{(Ri-Rj)}{\sqrt[]{ \frac{k(k+1)}{6n} }} .\end{eqnarray*}

**Table 4:** Wine Quality dataset results using RF as the control model.

	Ranking	*z* = (*R*_0_ − *R*_*i*_)∕*SE*	*Unadjusted p-value*	*Adjusted p-value* (Finner)
rfRFE	1.40	–	–	–
**RF**	1.60	0.1477	0.8825	0.8827
svmRadial	3.49	1.5509	0.1209	0.1349
nnet	4.80	2.5110	0.0120	0.0154
svm-RFE	5.00	2.6587	0.0078	0.0117
lm	6.40	3.6927	2.21E−4	3.99E−4
glmnet	7.30	4.3574	1.31E−5	2.96E−5
Lasso.RMSE	7.50	4.5051	6.63E−6	1.98E−5
glmStepAIC	7.70	4.6528	3.27E−6	1.47E−5
PLS	9.80	6.2038	5.51E−10	4.96E−9

**Table 5:** Automobile dataset results using RF as the control model.

	Ranking	*z* = (*R*_0_ − *R*_*i*_)∕*SE*	*Unadjusted p-value*	*Adjusted p-value* Finner
RF	1.30	–	–	–
**rfRFE**	1.70	0.2954	0.7676	0.7676
svmRadial	4.39	2.2895	0.0220	0.0247
Lasso.RMSE	5.10	2.8064	0.0050	0.0064
glmnet	5.20	2.8803	0.0039	0.0059
glmStepAIC	6.10	3.5450	3.92E−4	7.06E−4
lm	6.39	3.7665	1.65E−4	3.72E−4
nnet	7.00	4.2097	2.55E−5	7.67E−5
PLS	8.30	5.1698	2.34E−7	1.05E−6
svmRFE	9.50	6.0561	1.39E−9	1.25E−8

**Table 6:** Corona dataset results using RF as the control model.

	Ranking	*z* = (*R*_0_ − *R*_*i*_)∕*SE*	*Unadjusted p-value*	*Adjusted p-value* (Finner)
RF	3.30	–	–	–
Lasso.RMSE	3.39	0.0738	0.9411	0.9411
pls	3.99	0.5169	0.6051	0.6973
glmnet	4.00	0.5169	0.6051	0.6973
svm-RFE	5.00	1.2555	0.2092	0.2968
rfRFE	5.30	1.4470	0.1396	0.2871
**svmRadial**	5.30	1.4770	0.1396	0.2871
nnet	5.89	1.9202	0.0548	0.1556
lm	9.39	4.5051	6.63E−6	5.96E−5
glmStepAIC	9.39	4.5051	6.63E−6	5.96E−5

**Table 7:** Metal oxides dataset results using glmnet as the control model.

	Ranking	*z* = (*R*_0_ − *R*_*i*_)∕*SE*	*Unadjusted p-value*	*Adjusted p-value* (Finner)
glmnet	2.80	–	–	–
nnet	2.99	0.1825	0.8551	0.8551
**pls**	3.40	0.5477	0.5838	0.7069
Lasso.RMSE	3.40	0.5477	0.5838	0.7069
svmRadial	4.60	1.6431	0.1003	0.1689
rf	5.20	2.1908	0.0284	0.0651
lm	6.80	3.6514	2.60E−4	0.0018
glmStepAIC	6.80	3.6514	2.60E−4	0.0018

**Table 8:** Toxicity dataset results using svmRadial as the control model.

	Ranking	*z* = (*R*_0_ − *R*_*i*_)∕*SE*	*Unadjusted p-value*	*Adjusted p-value* (Finner)
svmRadial	2.40	–	–	–
**rfRFE**	2.80	0.2954	0.7676	0.7676
svm-RFE	3.10	0.5169	0.6051	0.6484
rf	4.30	1.4032	0.1605	0.2014
nnet	5.19	2.0679	0.0386	0.0574
glmStepAIC	5.89	2.5849	0.0097	0.0174
glmnet	7.10	3.4711	5.18E−4	0.0011
Lasso.RMSE	7.19	3.5450	3.92E−4	0.0011
lm	7.50	3.7665	1.65E−4	7.44E−4
pls	9.50	5.2436	1.57E−7	1.41E−6

Secondly, results showed that for 10 different dataset splits, using 10-fold repeated CV for and with the WineQuality dataset, according to a Shapiro–Wilk test with the null hypothesis that the data follow a normal distribution, obtaining a *p-value* of 0.0001449 and a *W* of 0.93794. Thus, the null hypothesis was rejected and our results did not follow a normal distribution. A Barlett’s test was performed with the null hypothesis that our results were heteroscedastic. The null hypothesis with a *p-value* of 0.9903 and a *k-squared* value of 2.0706 was rejected. At this point, one condition is not met; consequently, a non-parametric test should be employed assuming the null hypothesis that all models have the same performance. The null hypothesis was rejected with the Friedman test and the Iman-Davenport extension (*p*-*value* < 4.0520E–27). Hence, at this point, the null hypothesis is rejected with a very high level of significance, showing that the winning model is rfRFE due to the higher stability of the method across the ten experimental runs.

As in the previous case, Table 4 shows the average rankings of the techniques compared for the Wine Quality dataset. The ranking was presented, as well as each technique in the comparison with the test statistic of the Friedman test (z), the *unadjusted p-value* and the *adjusted p-value* with Finner’s procedure.

Finally, the results showed that 10 different dataset splits, using 10-fold repeated CV for the Automobile dataset, according to a Shapiro–Wilk test with the null hypothesis that the data follow a normal distribution obtained a *p-value* of 3.375E–10 and *W* of 0.80435. Thus, the null hypothesis was rejected and our results did not follow a normal distribution. A Barlett’s test was performed with the null hypothesis that our results were heteroscedastic. The null hypothesis with a *p-value* of 3.683E–08 and a *k-squared* value of 52.47 was rejected. At this point, two conditions are not met; consequently, a non-parametric test should be employed assuming the null hypothesis that all models have the same performance. The null hypothesis was rejected with the Friedman test and the Iman-Davenport extension (*p*-*value* < 5.031E–20). Hence, at this point the null hypothesis is rejected with a very high level of significance, showing that the winning model is RF.

The average rankings of the techniques compared for the Automobile dataset, and the test statistic of the Friedman test (z), the *unadjusted p-value* and the **adjusted p-value** with Finner’s procedure are shown in Table 5.

### Cheminformatics use case modeling

Once the proposed methodology provided satisfactory results using simple and well-known state-of-the-art datasets, it is tested against more complex problems. In Tsiliki et al. (2015a) authors proposed three case studies to test a methodology related to computer-aided model selection for QSAR predictive modelling in cheminformatics: proteomics data for surface-modified gold nanoparticles (Corona dataset), nano-metal oxides descriptor data (Gajewicz dataset), and molecular descriptors for acute aquatic toxicity data (Toxicity dataset).

#### Protein corona case study

Recent studies have shown that the presence of serum proteins *in vitro* cell culture systems form a protein adsorption layer (a.k.a. the ‘protein corona’) on the surface of nanoparticles (NPs). This section presents the results for the recently published proteomics data that characterizes the serum protein corona ‘fingerprint’ formed around a library of 105 distinct surface-modified gold NPs (Walkey et al., 2014). The authors used LC-MS/MS to identify 129 serum proteins which were considered suitable for relative quantification. From this dataset, the methodology proposed on RRegrs establishes that the best regression algorithm is svmRadial (with an average of *R*^2^: 0.5758) for this dataset.

However, the statistical study included as one of the required steps in the proposed methodology stated that the best model (in terms of accuracy and stability of results) should be RF (with an average *R*^2^ of 0.6191), giving the seventh position to svmRadial. The average rankings of the techniques compared for the use case 1 (methodology application on protein corona data), are shown in Table 6. This table also presents for each technique the comparison between the test statistic of the Friedman test (z), the *unadjusted p-value* and the *adjusted p-value* with Finner’s procedure. The name of the best model according to the methodology proposed in RRegrs is shown in bold.

**Figure 4:**
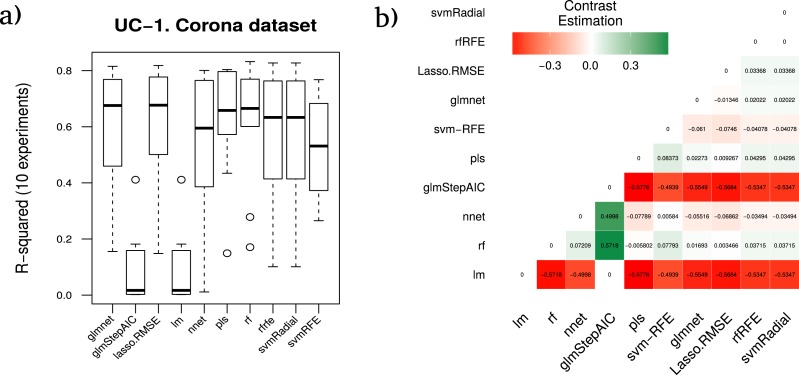
Results for the corona dataset. (A) Boxplot for the ten runs of each technique in order to show the behavior and stability of the method. (B) Contrast estimation based on medians where a positive difference value indicates that the method performs better than the other (green color).

The averaged performance *R*^2^ corresponding to each technique for the ten runs is shown in Fig. 4A as well as the contrast estimation based on medians between the  different approaches.

#### Gajewicz metal oxides case study

The authors of Gajewicz et al. (2015) combined experimental and theoretical measurements to develop a nano-QSAR model that described the toxicity of eighteen nano-metal oxides (MeOx) to the human keratinocyte (HaCaT) cell line, which is a common *in vitro* model for keratinocyte response during toxic dermal exposure. They calculated 32 parameters for each of the 18 samples that quantitatively describe the variability of the nanoparticles’ structure, called nano-descriptors, including particle size and size distribution, agglomeration state, particle shape, crystal structure, chemical composition, surface area, surface chemistry, surface charge, electronic properties (reactivity, conductivity, interaction energies, etc.), and porosity.

In this case, Table 7 shows the average rankings of the techniques compared for this dataset and the test statistic of the Friedman test (z), the *unadjusted p-value* and the *adjusted p-value* with Finner’s procedure for each technique within the comparison.

**Figure 5:**
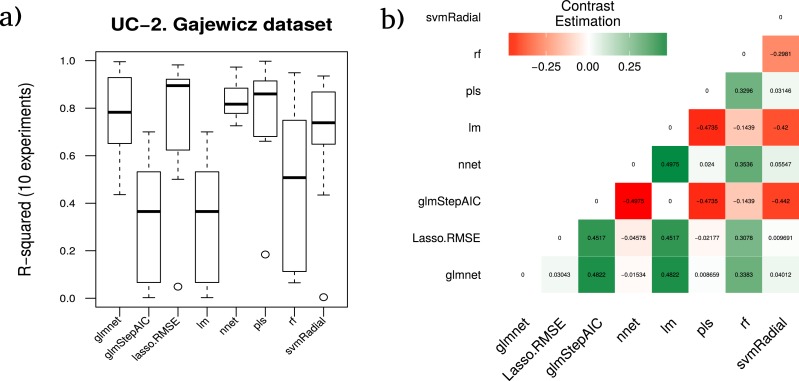
Results for the Gajewicz dataset. (A) Boxplot for the ten runs of each technique in order to show the behavior and stability of the method. (B) Contrast estimation based on medians where a positive difference value indicates that the method performs better than the other (green color).

The best model according to the RRegrs methodology (pls, in bold in Table 7) achieves an average *R*^2^ of 0.7885 whereas a more in depth analytical study of results provides glmnet as the best method with an average *R*^2^ of 0.7684. This is due to the higher stability of the results during the ten experiments provided by glmnet against the results achieved by PLS as shown in Fig. 5A).

The average performance *R*^2^ corresponding to each technique for the ten runs is shown in Fig. 5A, as well as the contrast estimation based on medians between the different approaches in Fig. 5B.

#### Aquatic toxicity case study

In Cassotti et al. (2014) a QSAR model based on 546 organic molecules was presented. It predicted acute aquatic toxicity towards Daphnia magna, which was the organism preferred for short-term aquatic toxicity testing. The data set provided 546 organic molecules and a set of 201 descriptors which were used to perform the classification.

The average rankings of the techniques on aquatic toxicity data are shown in Table 8. This table also presents for each method the statistic value of the Friedman test (z), the *unadjusted p-value* and the *adjusted p-value* with Finner’s procedure. The name of the best model according to the RRegrs methodology is in bold.

In this last dataset, the best model according to RRegrs was rfRFE which achieved an average *R*^2^ of 0.5255. The methodology proposed in our manuscript establishes svmRadial as the most reliable model in terms of stability and accuracy of the results, with an *R*^2^ of  0.5365.

**Figure 6:**
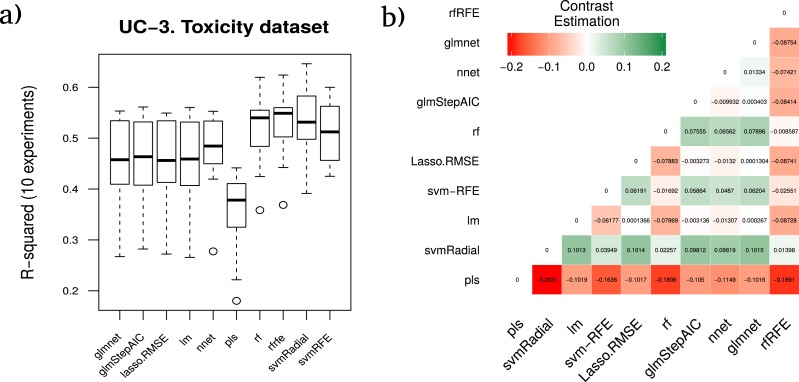
Results for the toxicity dataset. (A) Boxplot for the ten runs of each technique in order to show the behavior and stability of the method. (B) Contrast estimation based on medians where a positive difference value indicates that the method performs better than the other (green color).

The averaged performance *R*^2^ corresponding to each technique for the ten runs is shown in Fig. 6A, as well as the contrast estimation based on medians between the different approaches in Fig. 6B.

## Discussion

The experimental design proposed in this work has been previously used in other research studies, proving to perform well with classification tasks. The authors applied this formalized experimental design to different kinds of data, but mainly in bioinformatics, for example in texture analysis problems for classification of biomedical images (Fernandez-Lozano et al., 2015). The aforementioned study used the four phases of the normalized experimental design, applying different feature selection approaches for dimensionality reduction. The results showed that for all the generated datasets, the proposed methodology reported results that were reproducible, comparable and achieved in equality of conditions. Furthermore, a recent work carried out by the authors of the current study has employed this approach to assess whether the texture information could be used in proteomics to improve the quality of the image analysis of proteins separated on a gel (Fernandez-Lozano et al., 2016). These works were based on the experience of the group obtained in the processing of several cheminformatics datasets related to classification tasks (Fernandez-Lozano et al., 2013; Fernandez-Lozano et al., 2014; Aguiar-Pulido et al., 2013).

In the present work, the same methodology for the formalization of experimental designs was proven over a dataset related to regression tasks. These tests include simple datasets from the UCI Machine Learning repository and more complex case studies for QSAR predictive modeling. The achieved results show an excellent performance of the final selected models and demonstrate the importance of taking into account the variability of the models in order to choose the best one. This selection should not be based only on the best result achieved in one execution or in terms of the previously published methodology. The authors evaluated the performance of all models (with the test set) according to *R*^2^ and established a ranking. In the next step, they took into consideration all the models which are in the range (±0.05) according to this measure, and reordered the ranking, choosing the one with the lowest RMSE obtained in test.

The results also allowed us to understand that selecting the model only according to the best *R*^2^ or the highest accuracy and taking into consideration only one run is not stastically valid. An unusual high value in performance can be an outlier or maybe be due to a particular partition of the data during the model learning and should be discarded in favor of more stable methods. Aspects like stability in the results should be taken into account to make this selection.

Our proposal included the combination of two deep necessary modifications in the previous methodology (external CV process in the learning phase and statistical analysis in the best model selection phase) and a simple consideration about the diversity of the data, in order to handle count data during the pre-processing of the data phase. To the best of our knowledge, our findings are of relevance and it can be assumed that other Machine Learning models, in general, should behave similarly to those presented in this work, as well as the same algorithms with other datasets. Generally speaking, if the training process or the algorithms are stochastic in some way, one should repeat several experimental runs in order to find the best results, and it is crucial to use a statistical comparison to evaluate whether the differences in performance score are relevant before assuming a final best model. Otherwise, the final conclusions could be wrong and the final model should be  discarded.

Furthermore, in order to ensure the power and validity of our results and methodology, using the data resulting from the different splits over the three cheminformatics use cases datasets, the estimation of the differences between each pair of ML techniques was checked, using the approach proposed in Doksum (1967) as shown in Figs. 4–6B. Thus, the real differences between our methodology and the one proposed in RRgres (Tsiliki et al., 2015a) could be computed. This procedure is especially useful to estimate a method’s performance over another (García et al., 2010).

Finally, it was proven that our methodology is applicable in other fields, as it is enough open to add new phases and we test our proposal with eight different datasets from very different scopes. At the same time, given our results, it can be stated that the same behavior is expected with other ML algorithms and further statistical studies are expected to be relevant in this sense.

The main objective of this kind of methodologies in QSAR predictive modeling is to help with the initial exploration of extensive databases of drugs, proteins, molecules, etc. in order to design comprehensive *in silico* screening methods, reducing the economic costs as much as possible. At the same time, the quality and reliability of the ML methods used in the process should be ensured.

## Conclusions

This paper proposed a new multidisciplinary methodology for performing experiments in Computational Intelligence, which was tested with a well-known methodology in the area using the RRegrs package. Ten computational intelligence algorithms for data mining regression tasks and five state-of-the-art datasets were used in this sense. Different critical modifications were proposed and tested in several phases of the previous published methodology, and our results are of relevance. For a better understanding of our proposal, all the steps were applied to the baseline example of an experimental study on well-known state-of-the-art datasets previously published in different fields.

The final statistical study phase was described in detail, as it is the most critical modification, as well as the external cross-validation. With the three datasets in which our results were different from the previous methodology, and with the three real use case datasets, it can be concluded that, following our methodology, the results can be checked for statistical significance and thus, reliable *in silico* models in cheminformatics should be proposed to the scientific community. Furthermore, using our open methodology for regression or classification problems, the generated Machine Learning models can be tested against poorly experimental designs in order to clearly state that the obtained results are stable, reproducible and relevant.

In the near future, our group is planning to include this methodology in the RRegrs package so that it could be used by the scientific community, as this work is distributed under the terms of the Creative Commons Attribution 4.0 International License.

## Supplemental Information

10.7717/peerj.2721/supp-1 Data S1 Original datasets, raw data results, summary file results for each dataset separated in folders Datailed results from UC Irvine Machine Learning Repository (Housing, Machine CPU, Wine Quality, Automobile and Parkinson) and the 3 Use Cases (Protein Corona, Gajewicz Metal Oxides and Aquatic Toxicity)

## Additional Information and Declarations

### Data Availability

The following information was supplied regarding data availability: The raw data has been supplied as a Data S1.
