# Supplementary material for: A methodology for the design of experiments in computational intelligence with multiple regression models
Source: PeerJ. 2016 Dec 1;4:e2721. doi: 10.7717/peerj.2721 (PMC5136129; doi:10.7717/peerj.2721)
Supplement: Data S1 — Datailed results from UC Irvine Machine Learning Repository (Housing, Machine CPU, Wine Quality, Automobile and Parkinson) and the 3 Use Cases (Protein Corona, Gajewicz Metal Oxides and Aquatic Toxicity) [file peerj-04-2721-s001.zip › 1_housing/RRegrsResBest.csv.repeatedcv.split10.pdf]

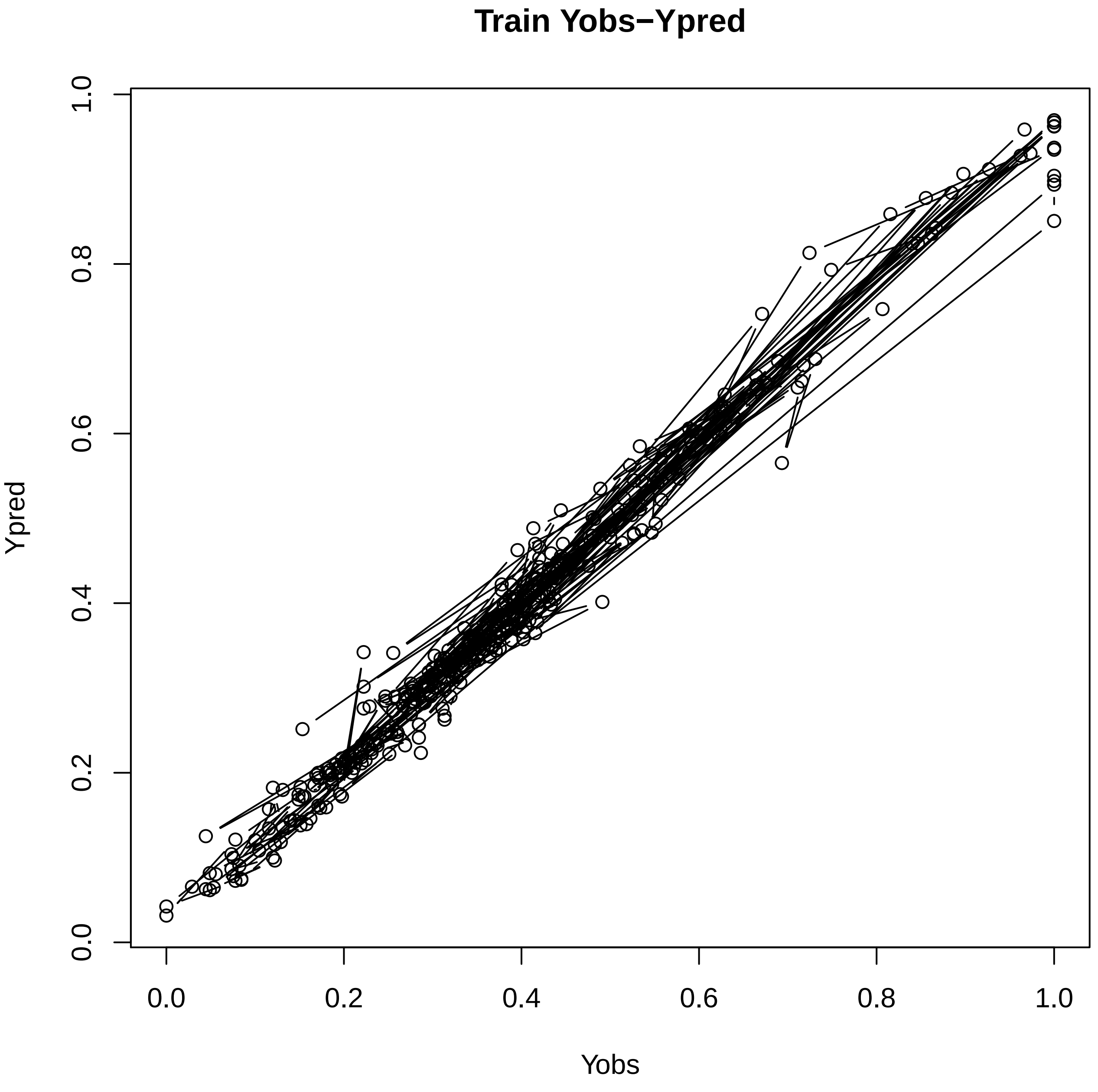
Train Yobs−Ypred
Ypred
Yobs
0.0
0.2
0.4
0.6
0.8
1.0
0.0
0.2
0.4
0.6
0.8
1.0

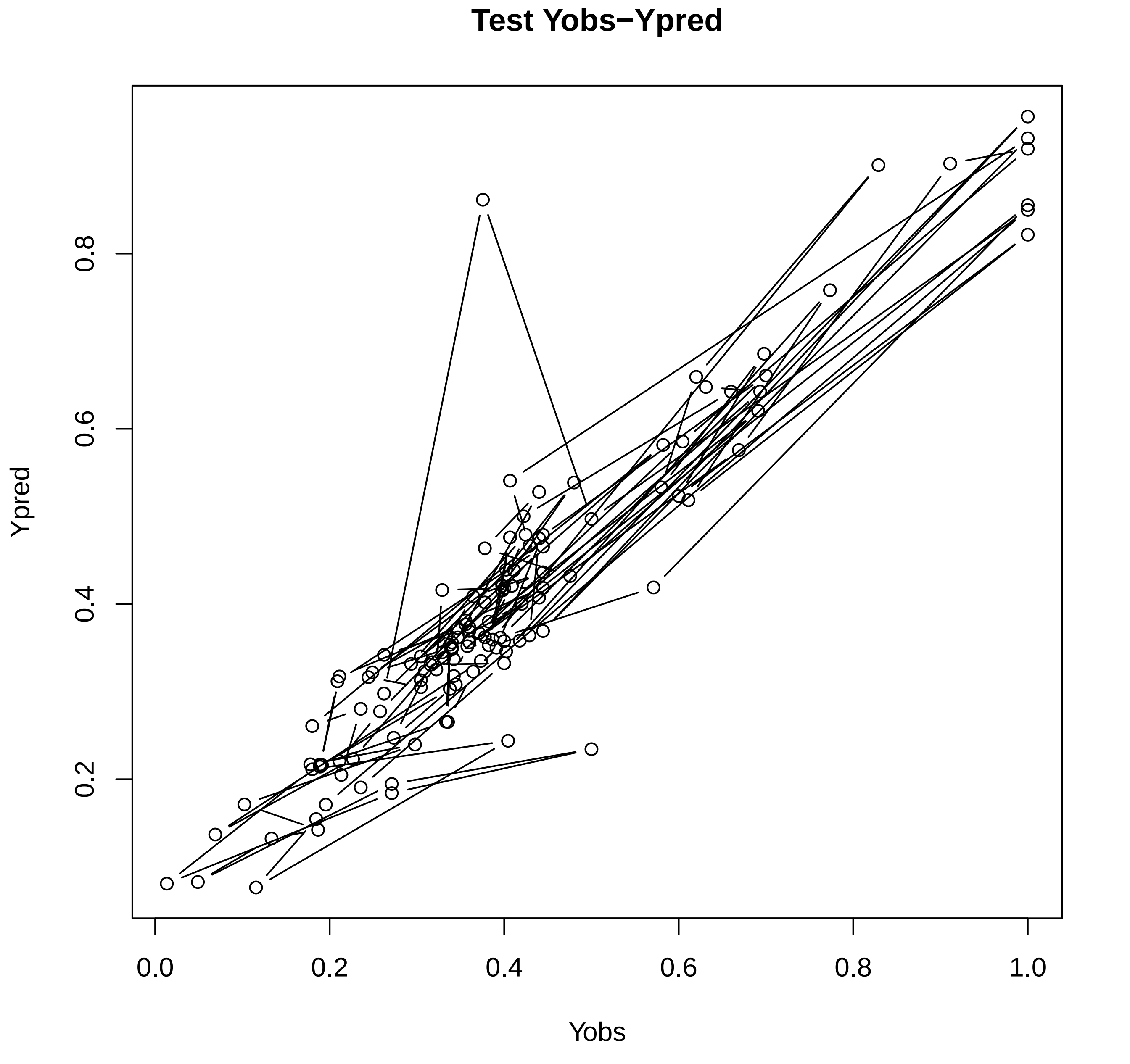
Test Yobs−Ypred
Ypred
Yobs
0.2
0.4
0.6
0.8
0.0
0.2
0.4
0.6
0.8
1.0

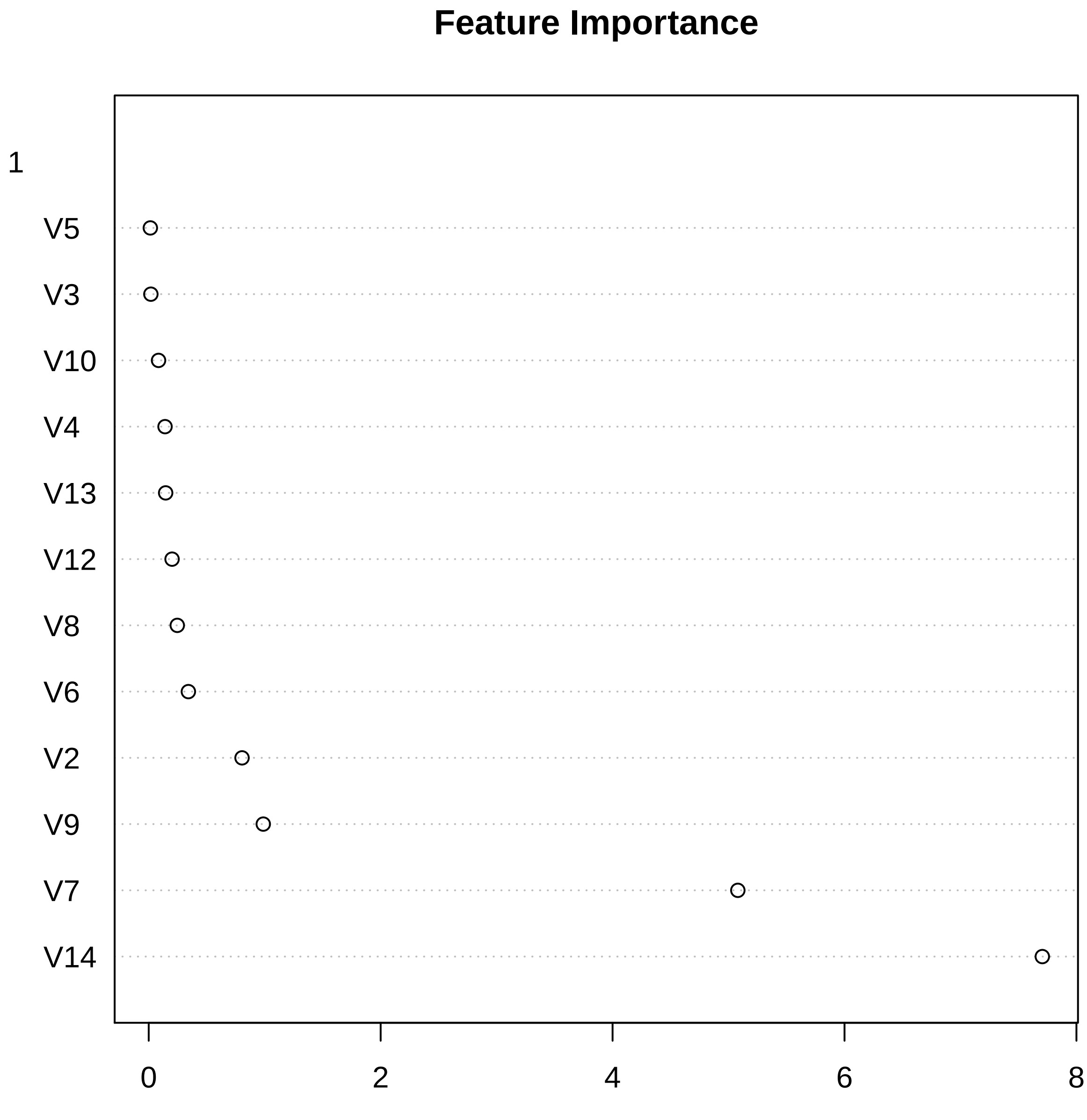
Feature Importance
1
V5
V3
V10
V4
V13
V12
V8
V6
V2
V9
V7
V14
0
2
4
6
8

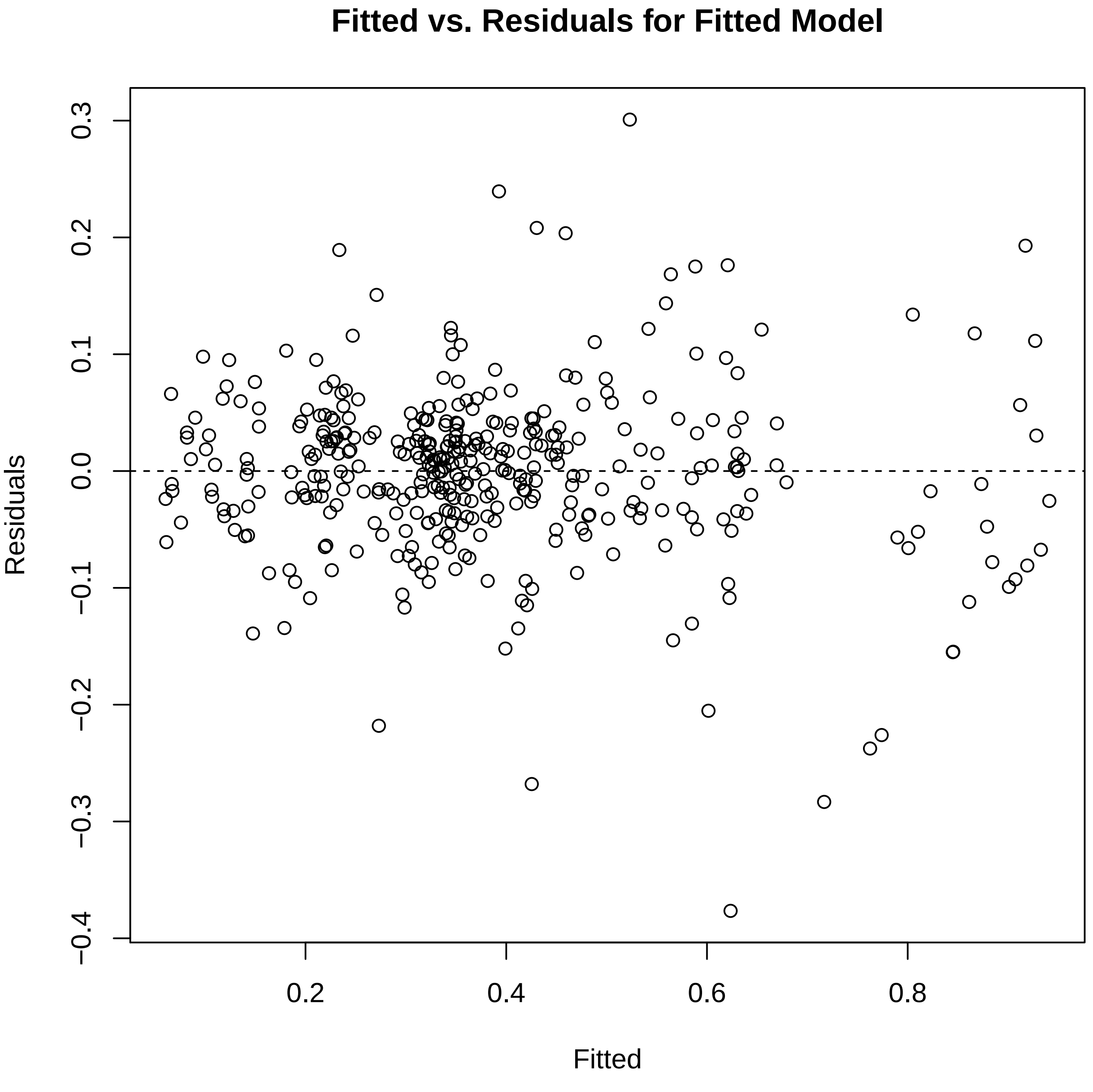
Fitted vs. Residuals for Fitted Model
Residuals
Fitted
0.3
0.2
0.1
0.0
−0.1
−0.2
−0.3
−0.4
0.2
0.4
0.6
0.8

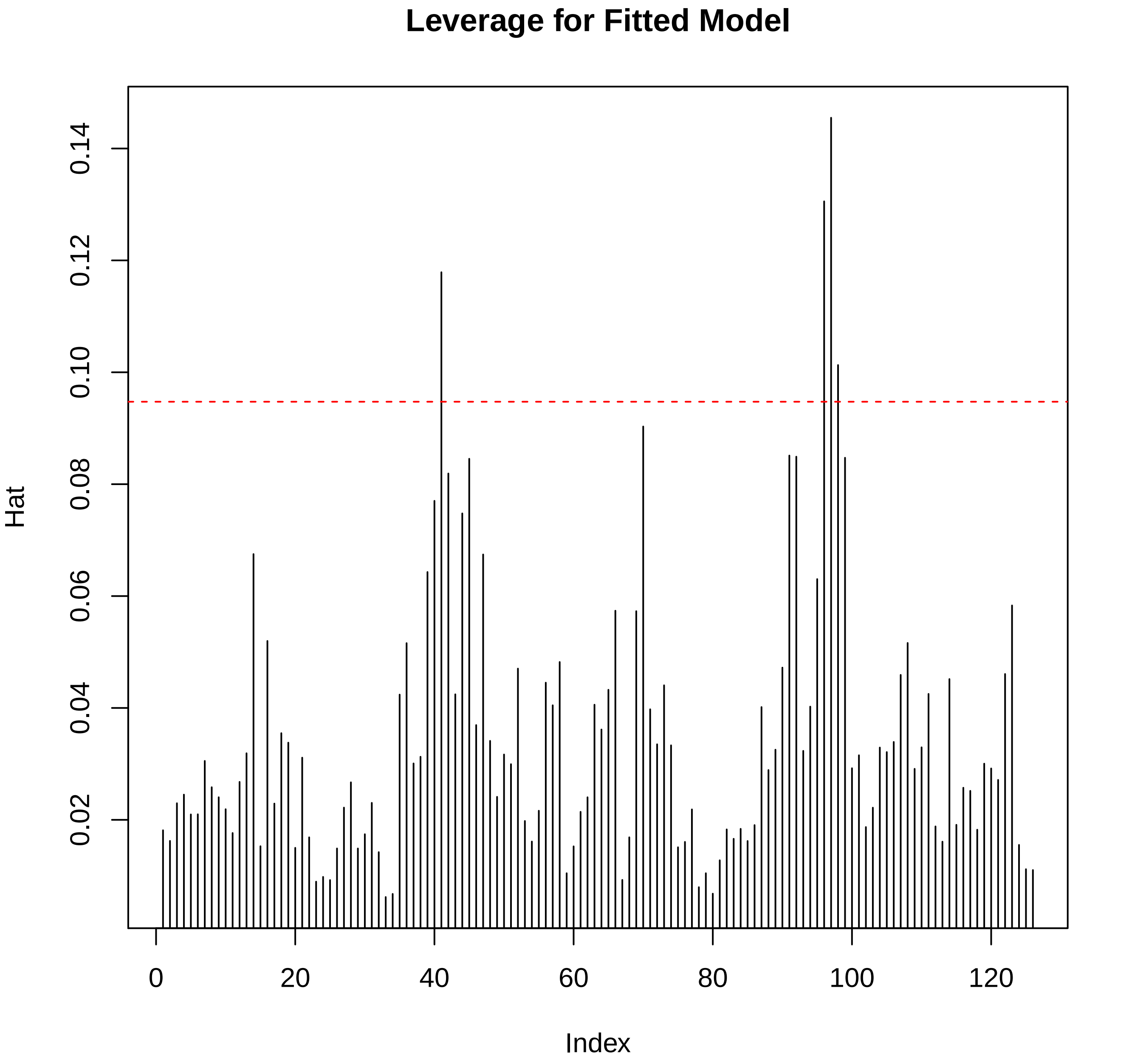
Leverage for Fitted Model
Hat
Index
0.02
0.04
0.06
0.08
0.10
0.12
0.14
0
20
40
60
80
100
120
