# Supplementary material for: A methodology for the design of experiments in computational intelligence with multiple regression models
Source: PeerJ. 2016 Dec 1;4:e2721. doi: 10.7717/peerj.2721 (PMC5136129; doi:10.7717/peerj.2721)
Supplement: Data S1 — Datailed results from UC Irvine Machine Learning Repository (Housing, Machine CPU, Wine Quality, Automobile and Parkinson) and the 3 Use Cases (Protein Corona, Gajewicz Metal Oxides and Aquatic Toxicity) [file peerj-04-2721-s001.zip › 2_machineCPU/RRegrsResBest.csv.repeatedcv.split10.pdf]

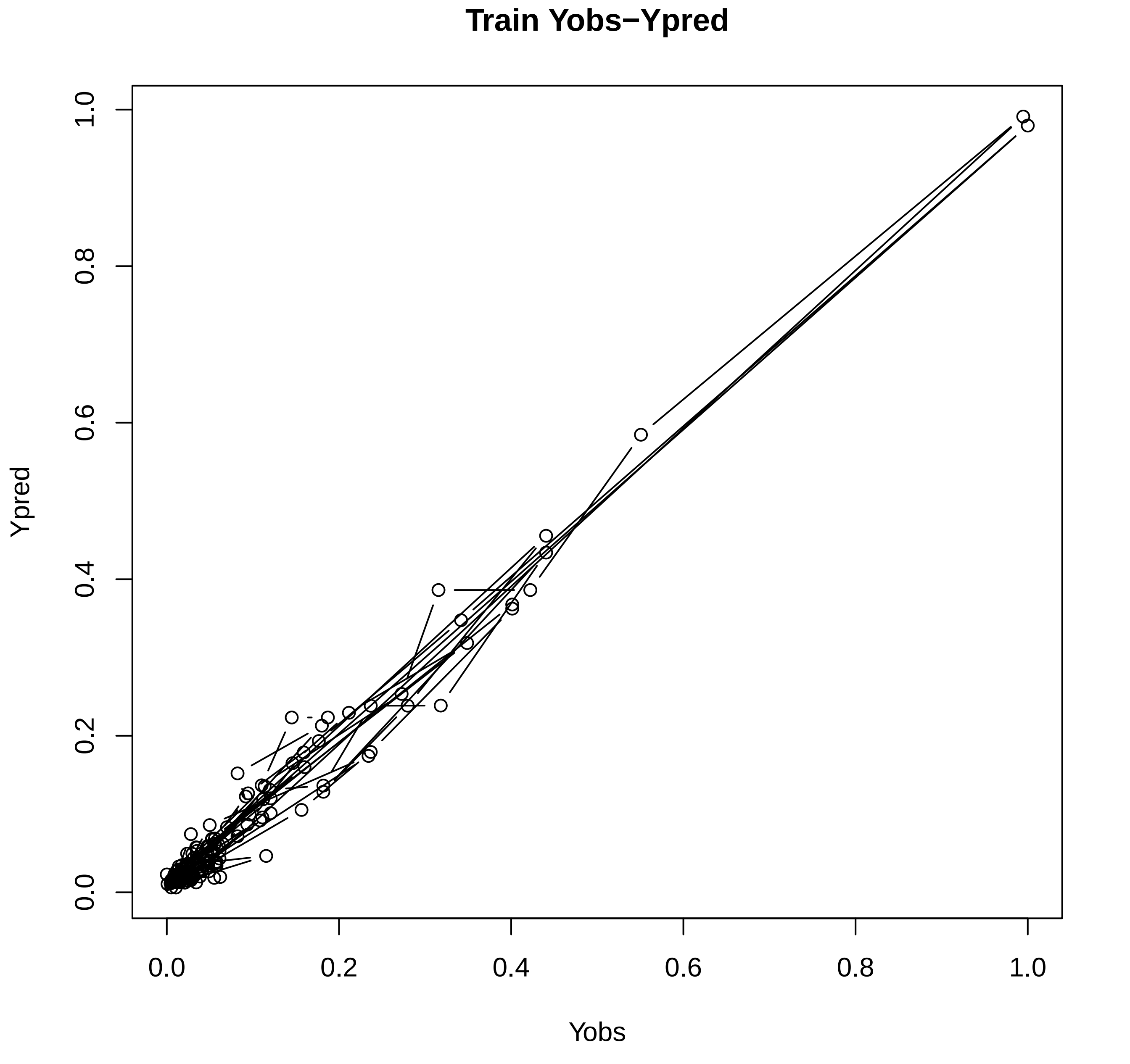

Train Yobs−Ypred
Ypred
Yobs
0.0
0.2
0.4
0.6
0.8
1.0

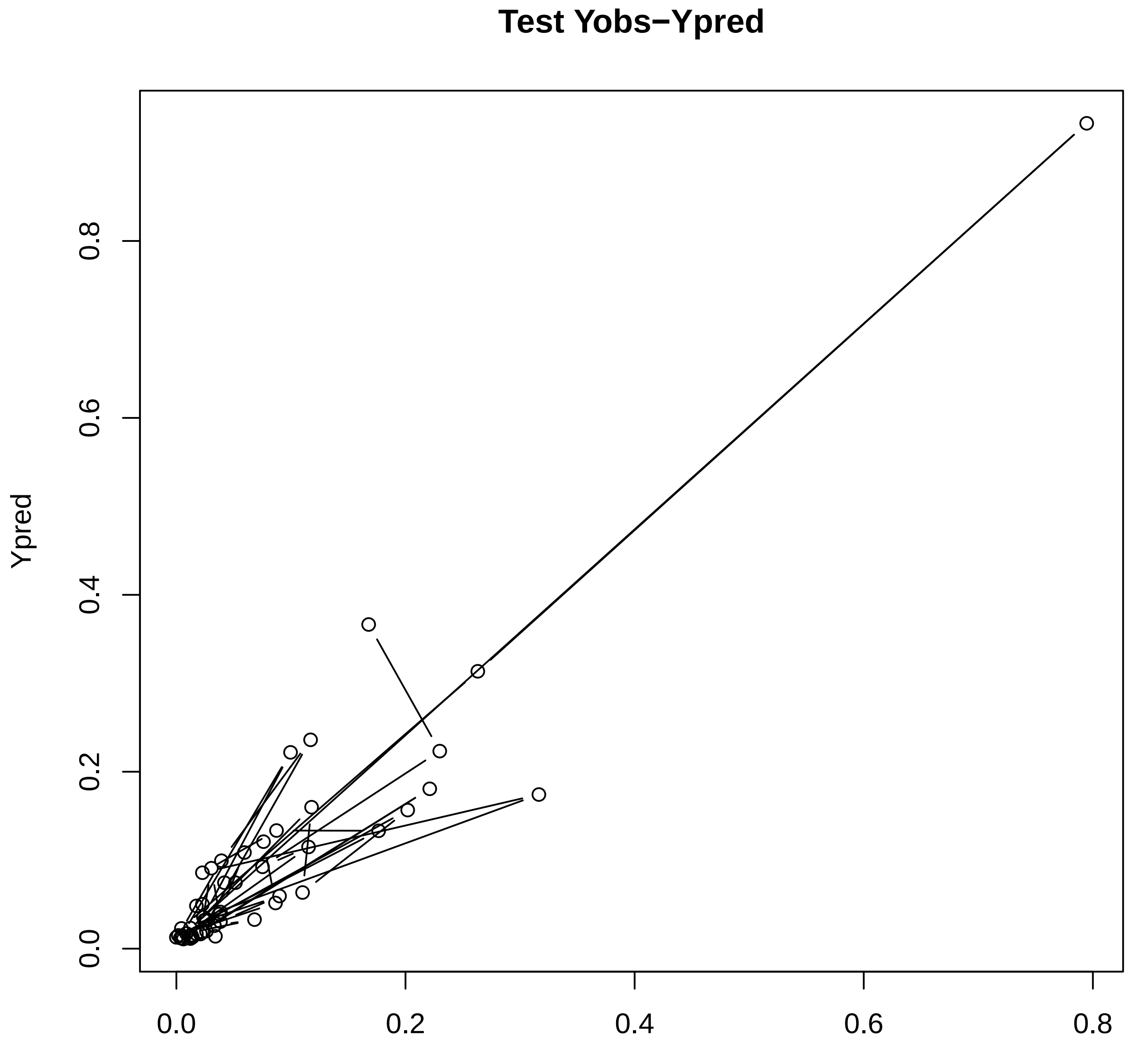
Test Yobs−Ypred
Ypred
Yobs
0.0
0.2
0.4
0.6
0.8
0.0
0.2
0.4
0.6
0.8

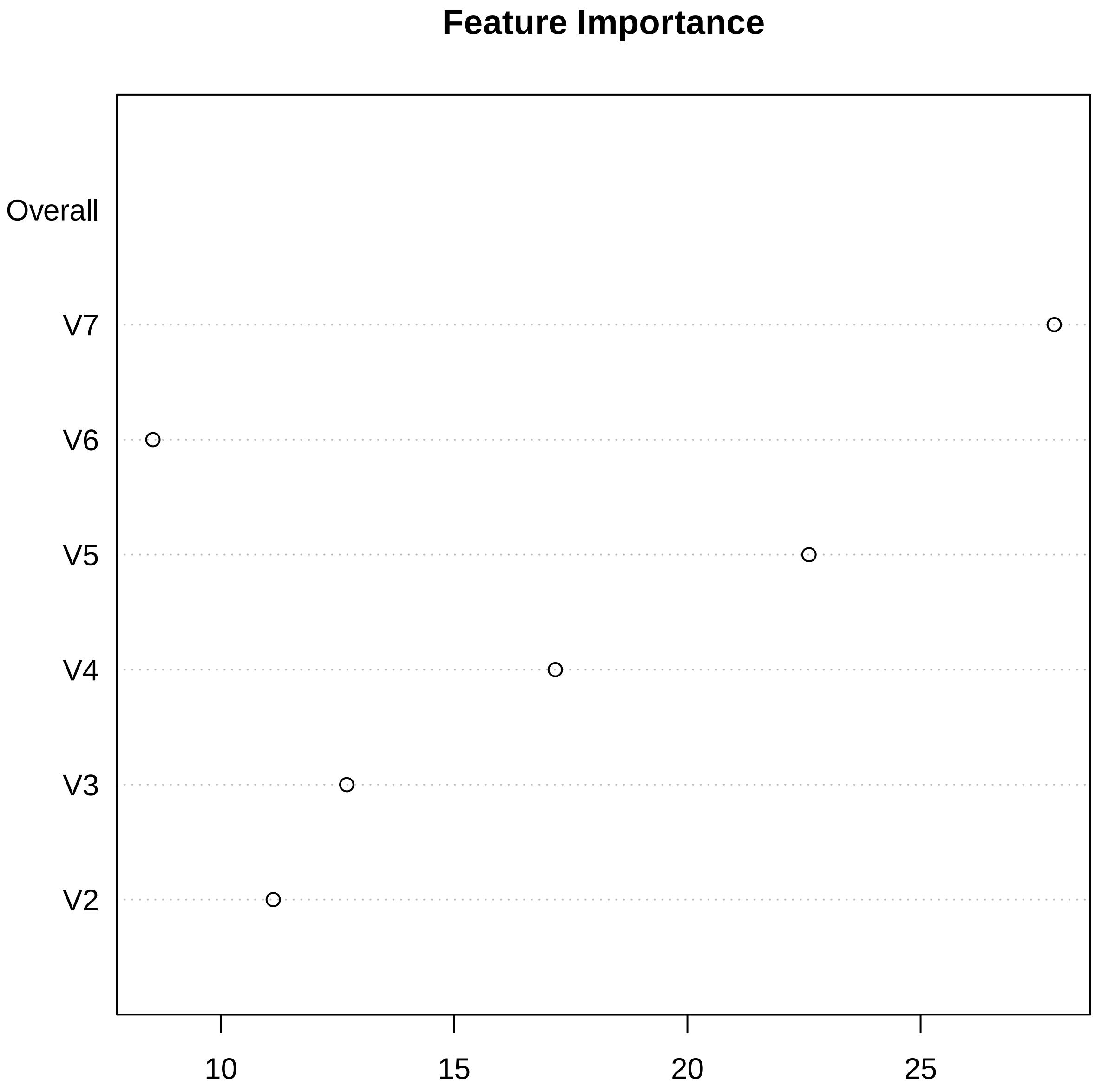
Feature Importance
Overall
V7
V6
V5
V4
V3
V2
10
15
20
25

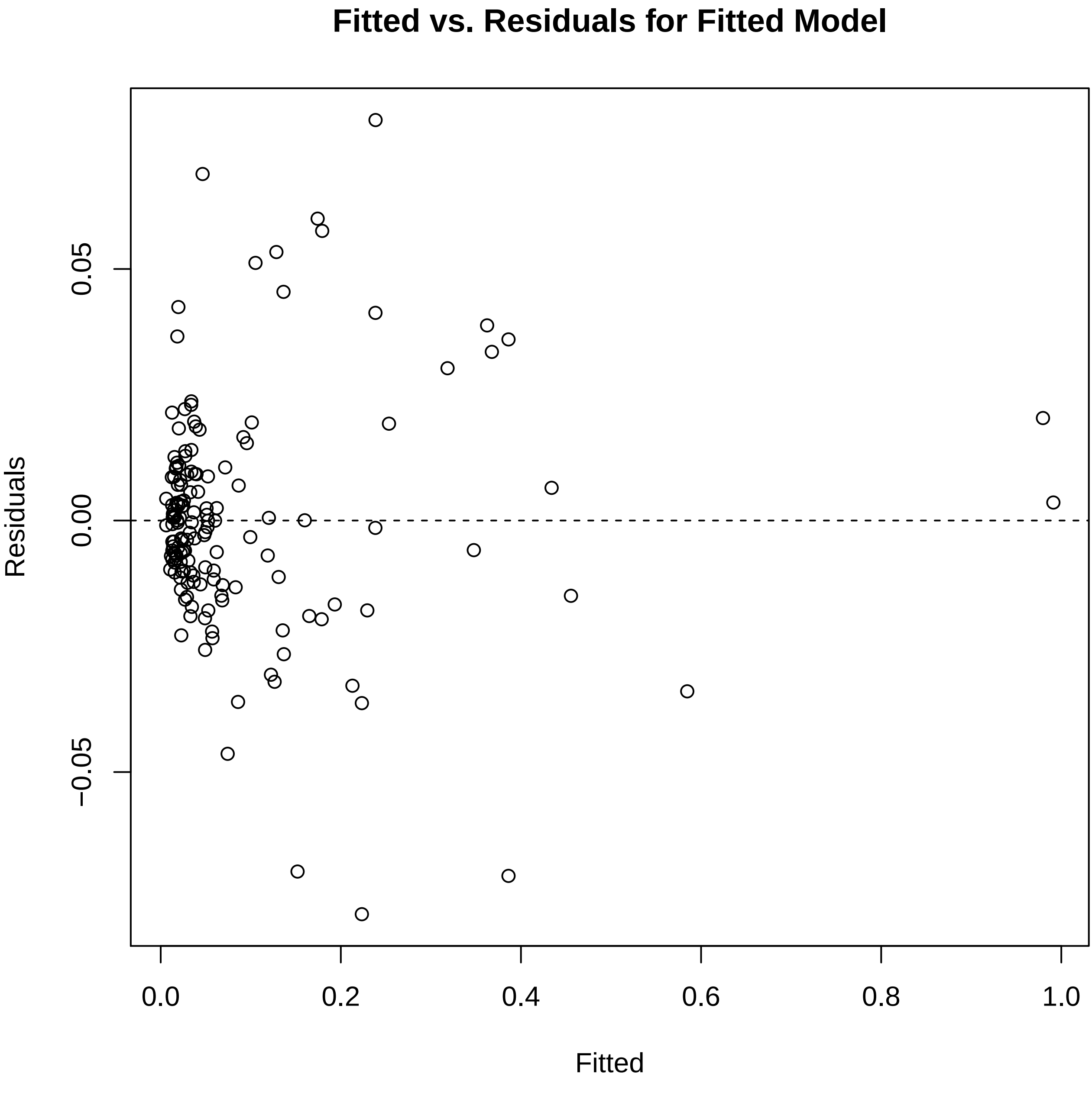

Fitted vs. Residuals for Fitted Model
Residuals
Fitted
0.05
0.00
−0.05
0.0
0.2
0.4
0.6
0.8
1.0

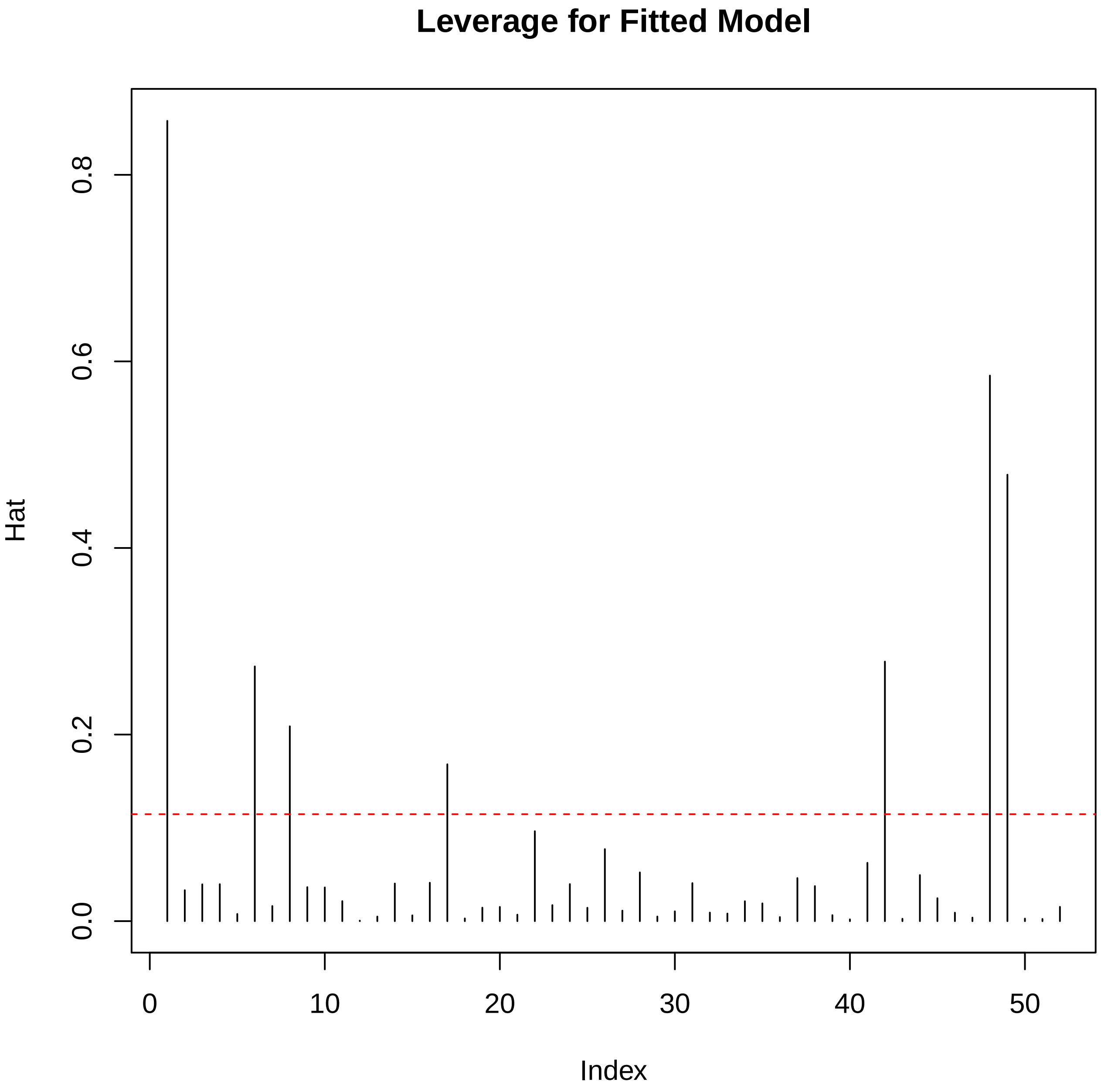

Leverage for Fitted Model
Hat
0.0
0.2
0.4
0.6
0.8
0
10
20
30
40
50
Index
