# Supplementary material for: A methodology for the design of experiments in computational intelligence with multiple regression models
Source: PeerJ. 2016 Dec 1;4:e2721. doi: 10.7717/peerj.2721 (PMC5136129; doi:10.7717/peerj.2721)
Supplement: Data S1 — Datailed results from UC Irvine Machine Learning Repository (Housing, Machine CPU, Wine Quality, Automobile and Parkinson) and the 3 Use Cases (Protein Corona, Gajewicz Metal Oxides and Aquatic Toxicity) [file peerj-04-2721-s001.zip › 3_winequality/RRegrsResBest.csv.repeatedcv.split10.pdf]

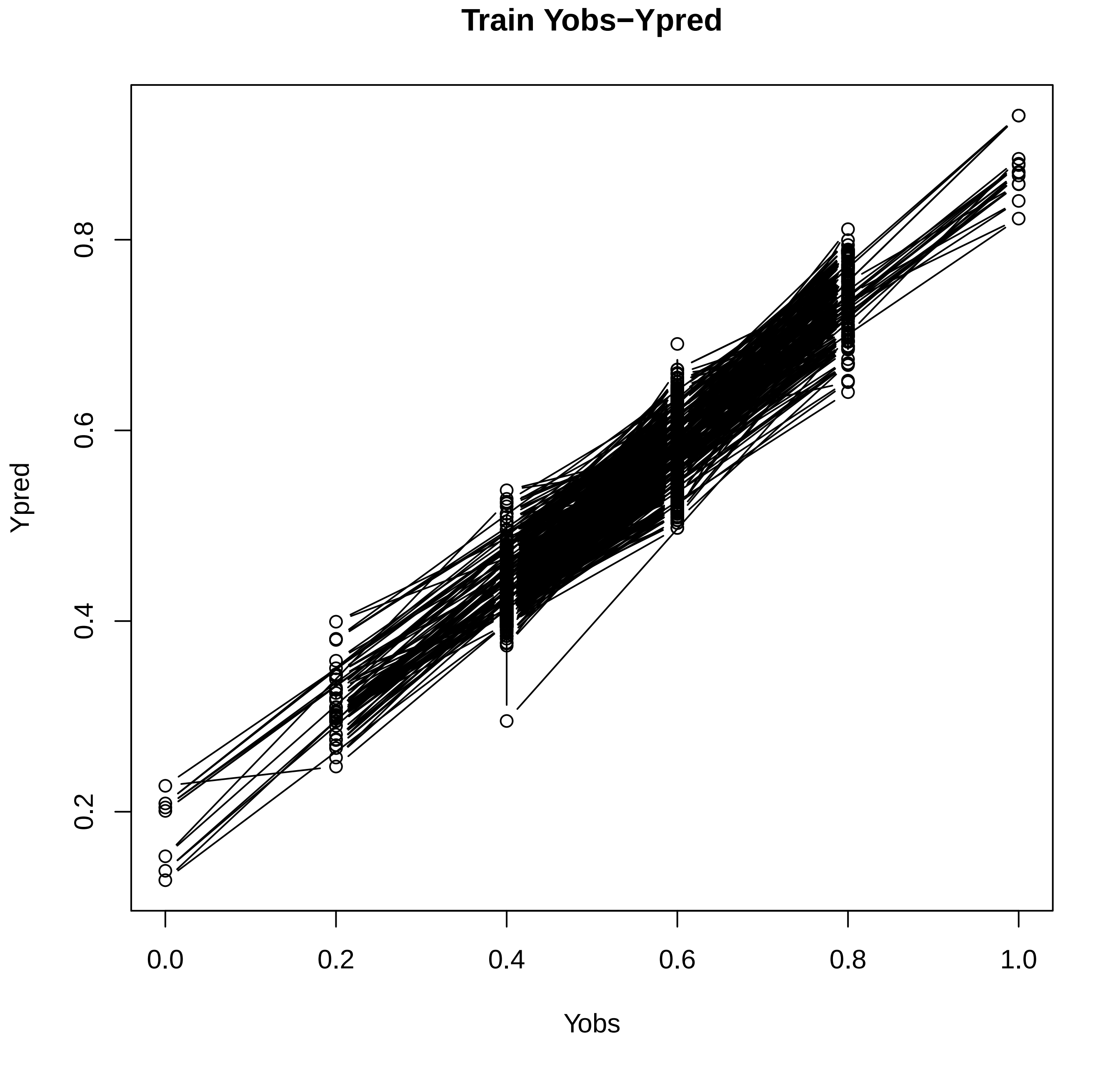

Train Yobs−Ypred
Ypred
Yobs
0.2
0.4
0.6
0.8
0.0
0.2
0.4
0.6
0.8
1.0

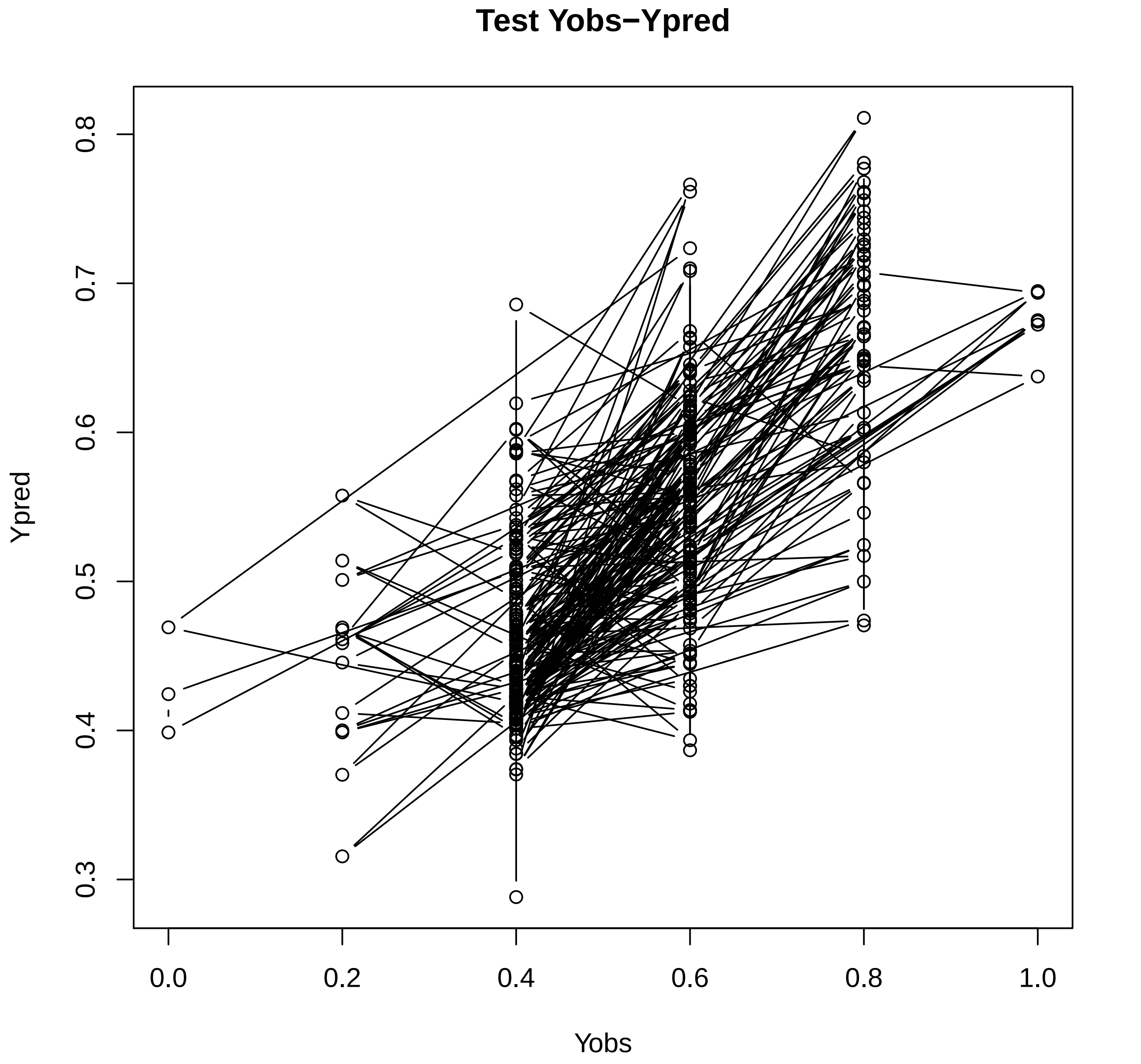

Test Yobs−Ypred
Ypred
0.3
0.4
0.5
0.6
0.7
0.8
Yobs
0.0
0.2
0.4
0.6
0.8
1.0

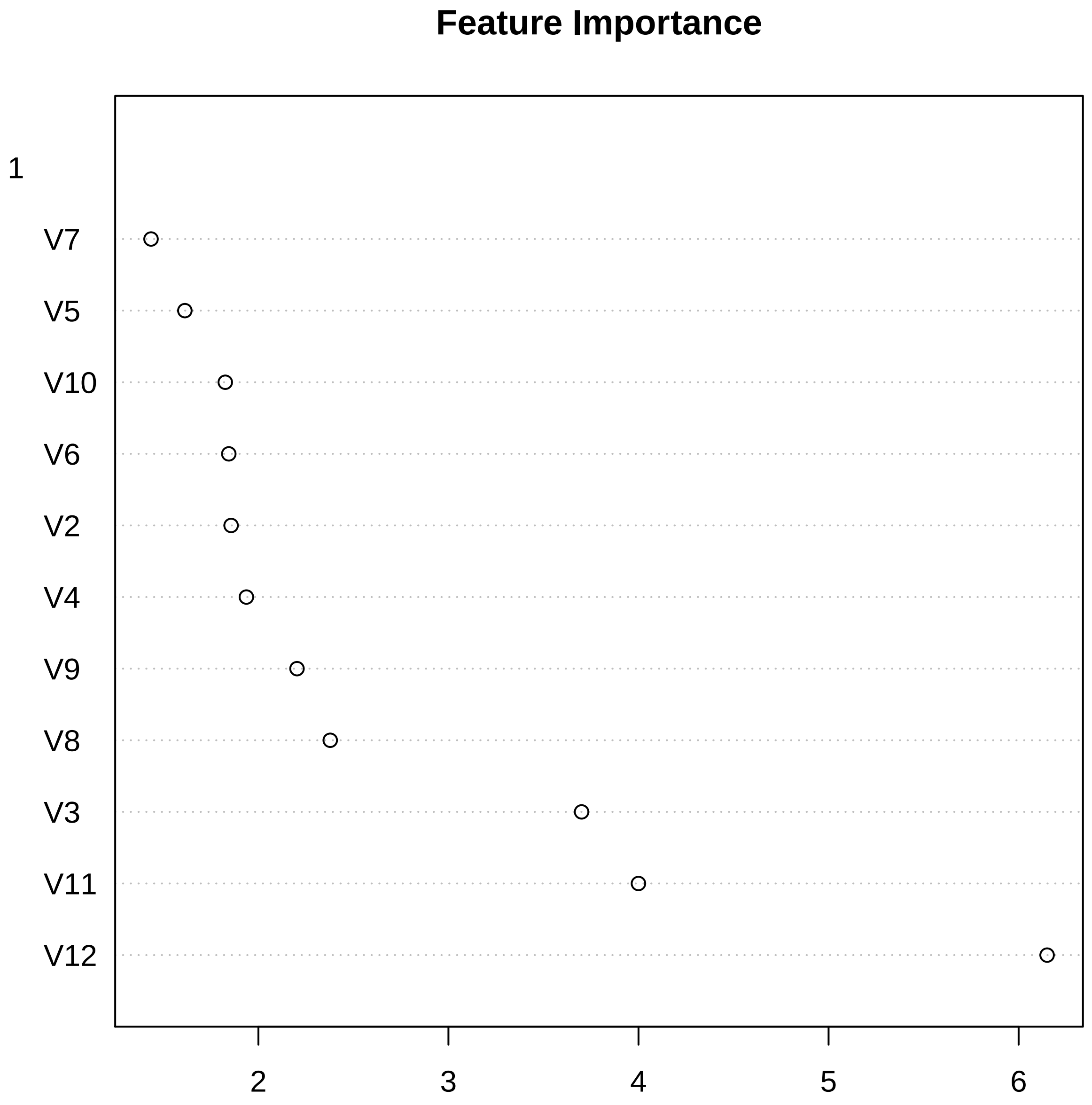
Feature Importance
1
V7
V5
V10
V6
V2
V4
V9
V8
V3
V11
V12
2
3
4
5
6

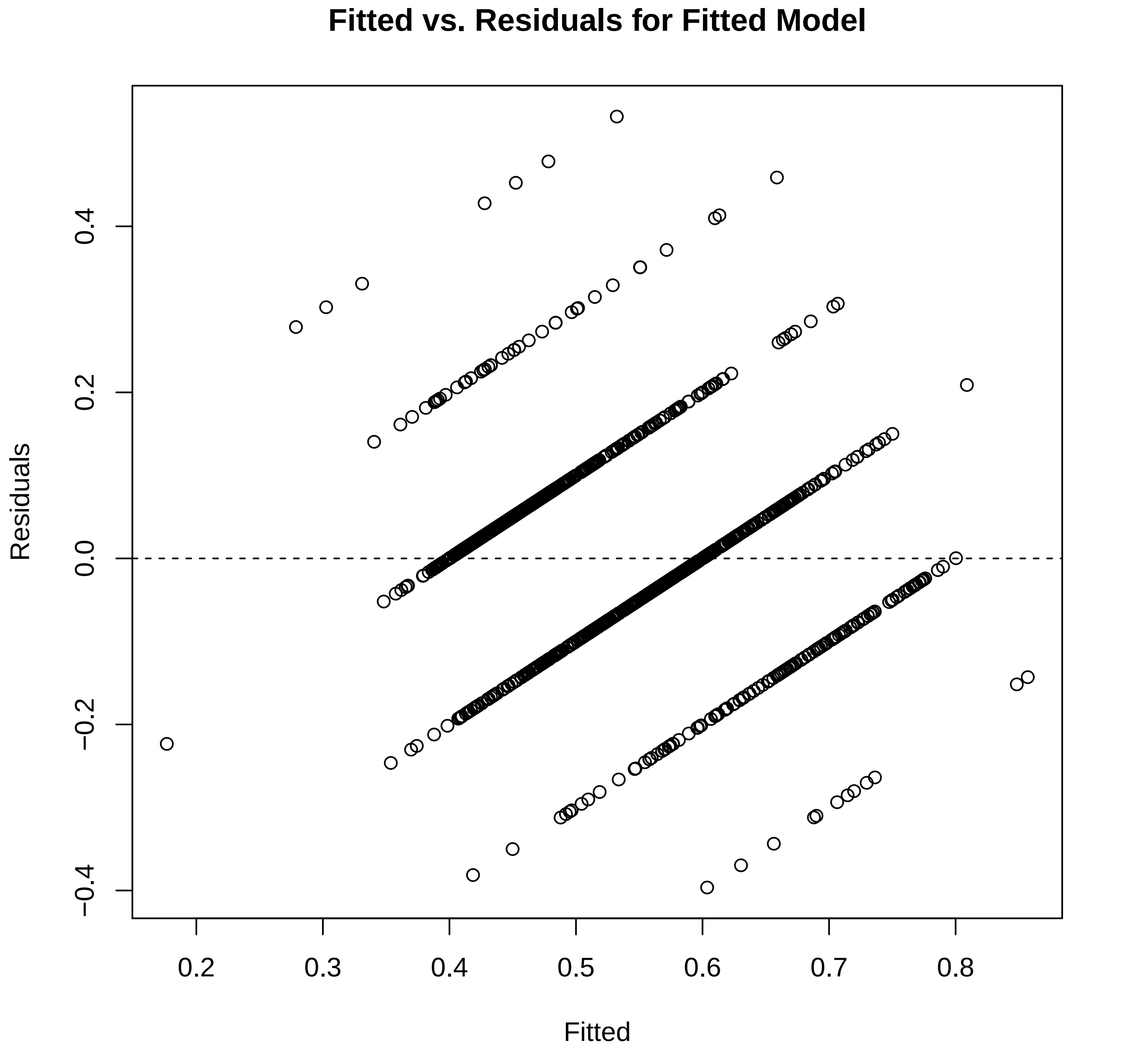

Fitted vs. Residuals for Fitted Model
Residuals
Fitted
0.4
0.2
0.0
−0.2
−0.4
0.2
0.3
0.4
0.5
0.6
0.7
0.8

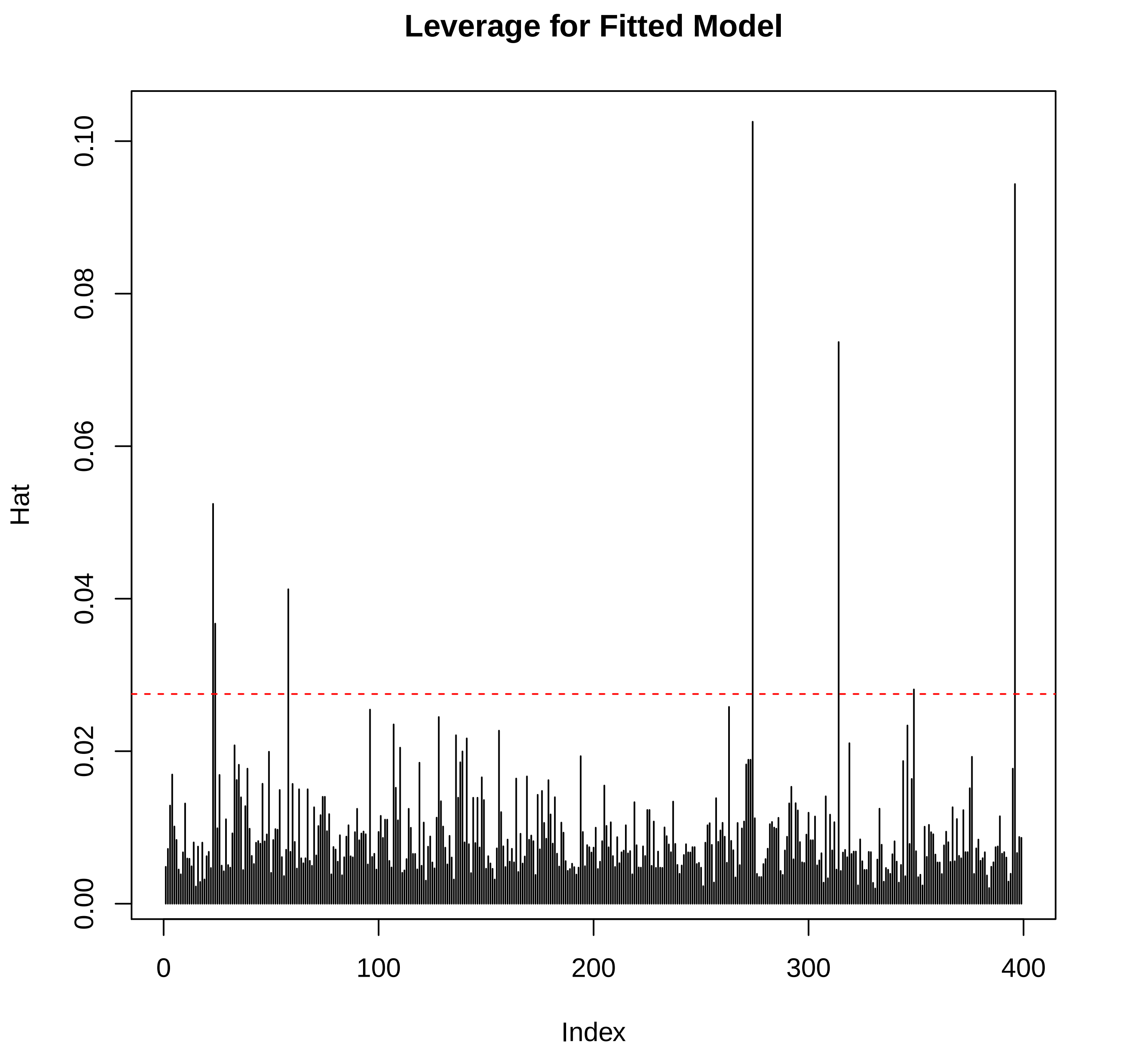

Leverage for Fitted Model
Hat
0.00
0.02
0.04
0.06
0.08
0.10
0
100
200
300
400
Index
