# Supplementary material for: A methodology for the design of experiments in computational intelligence with multiple regression models
Source: PeerJ. 2016 Dec 1;4:e2721. doi: 10.7717/peerj.2721 (PMC5136129; doi:10.7717/peerj.2721)
Supplement: Data S1 — Datailed results from UC Irvine Machine Learning Repository (Housing, Machine CPU, Wine Quality, Automobile and Parkinson) and the 3 Use Cases (Protein Corona, Gajewicz Metal Oxides and Aquatic Toxicity) [file peerj-04-2721-s001.zip › 4_automobile/RRegrsResBest.csv.repeatedcv.split10.pdf]

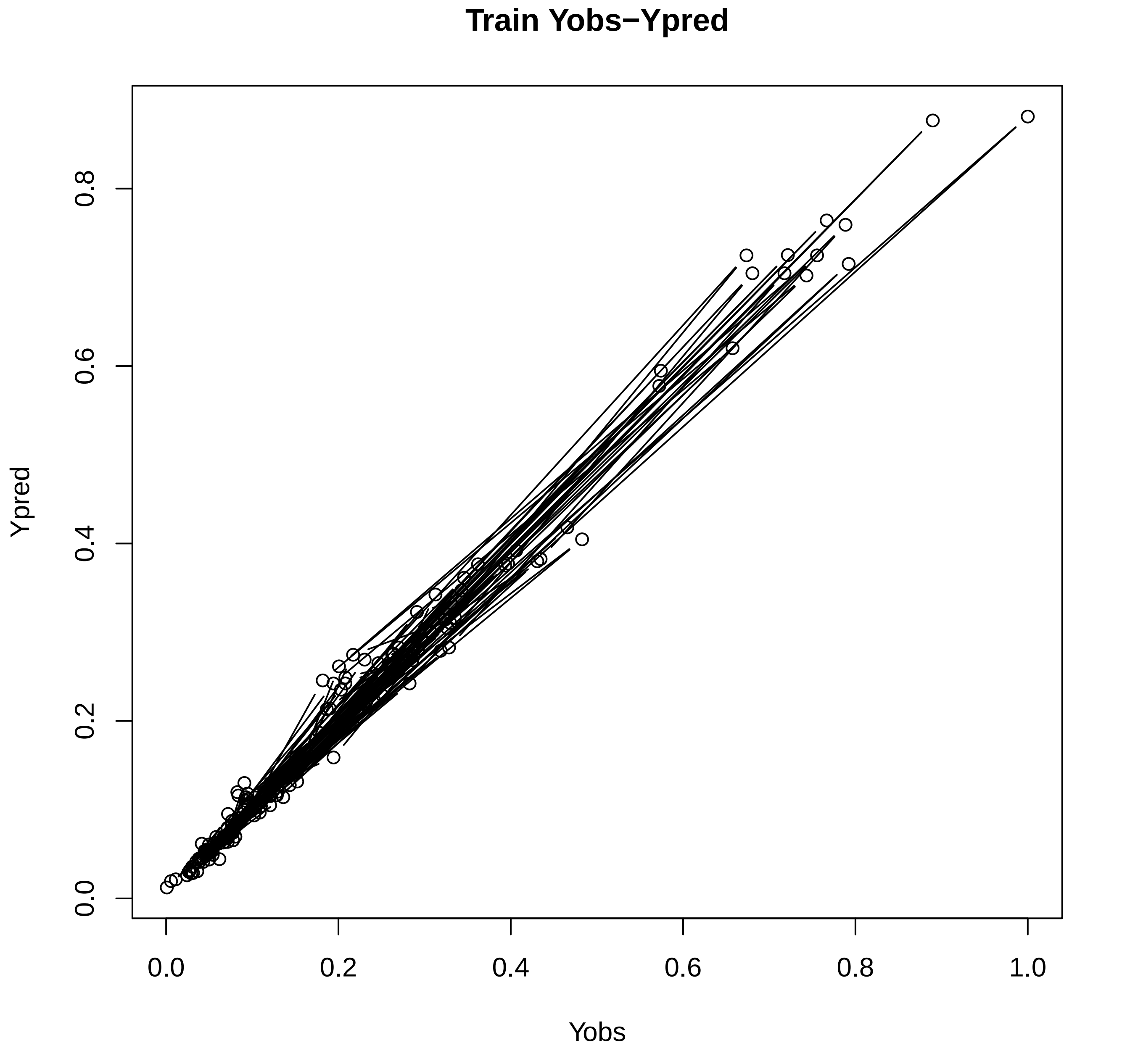
Train Yobs−Ypred
Ypred
0.0
0.2
0.4
0.6
0.8
Yobs
0.0
0.2
0.4
0.6
0.8
1.0

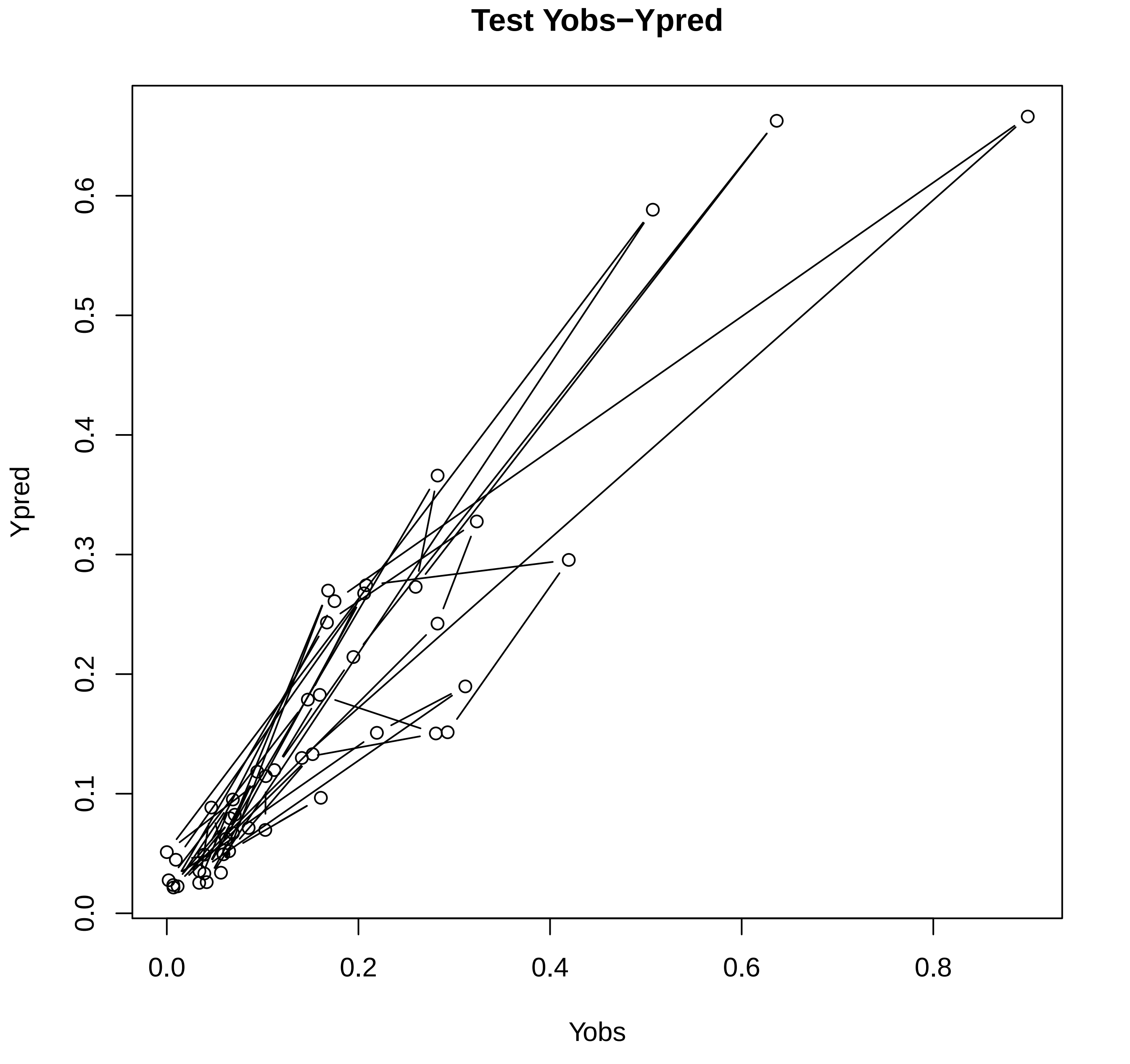

Test Yobs−Ypred
Ypred
Yobs
0.0
0.1
0.2
0.3
0.4
0.5
0.6
0.0
0.2
0.4
0.6
0.8

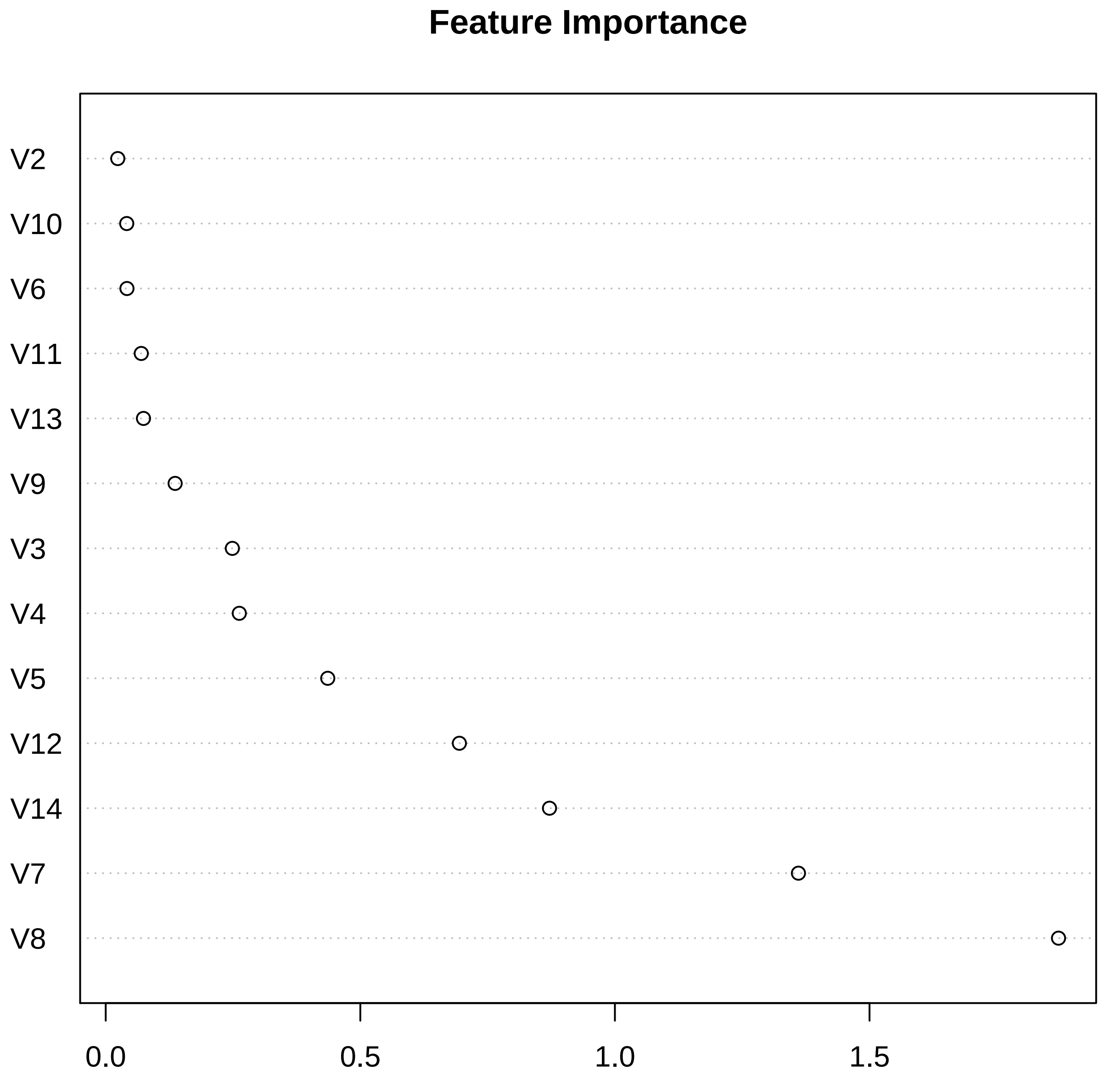
Feature Importance
V2
V10
V6
V11
V13
V9
V3
V4
V5
V12
V14
V7
V8
0.0
0.5
1.0
1.5

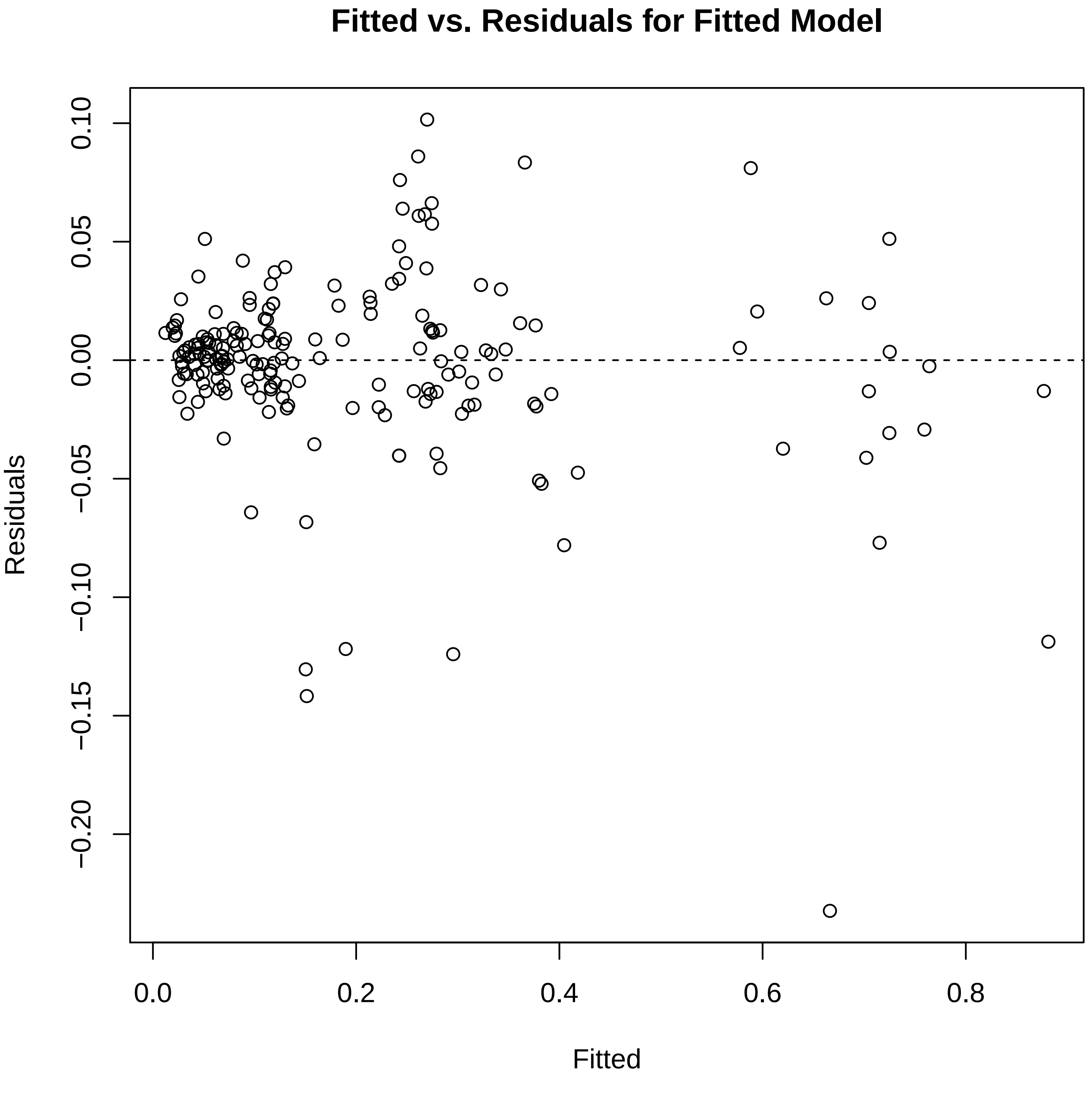
Fitted vs. Residuals for Fitted Model
Residuals
Fitted
0.10
0.05
0.00
−0.05
−0.10
−0.15
−0.20
0.0
0.2
0.4
0.6
0.8

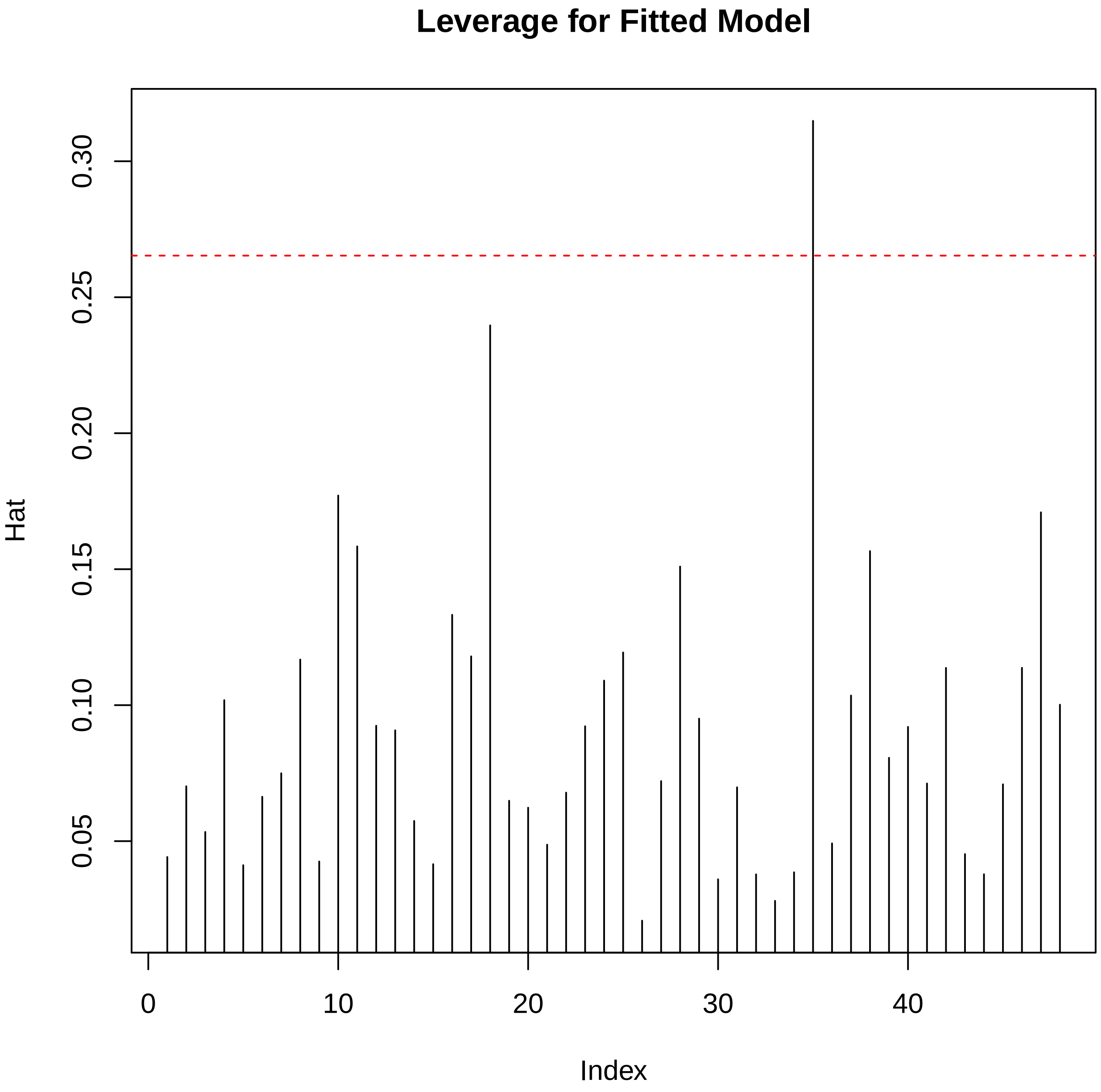

Leverage for Fitted Model
0.05
0.10
0.15
0.20
0.25
0.30
Hat
0
10
20
30
40
Index
