# Supplementary material for: A methodology for the design of experiments in computational intelligence with multiple regression models
Source: PeerJ. 2016 Dec 1;4:e2721. doi: 10.7717/peerj.2721 (PMC5136129; doi:10.7717/peerj.2721)
Supplement: Data S1 — Datailed results from UC Irvine Machine Learning Repository (Housing, Machine CPU, Wine Quality, Automobile and Parkinson) and the 3 Use Cases (Protein Corona, Gajewicz Metal Oxides and Aquatic Toxicity) [file peerj-04-2721-s001.zip › 5_parkinson/RRegrsResBest.csv.repeatedcv.split10.pdf]

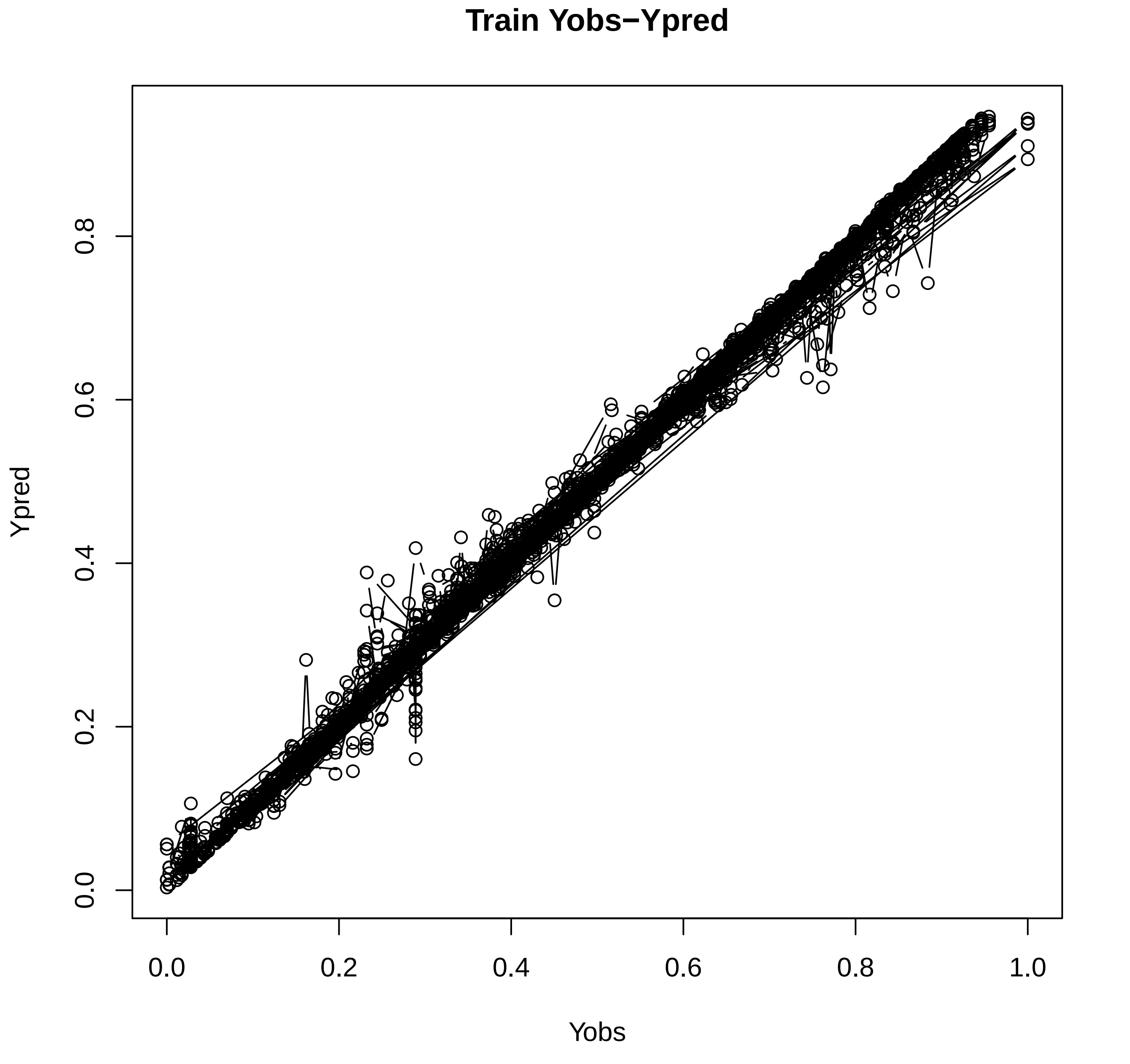

Train Yobs−Ypred
Ypred
0.0
0.2
0.4
0.6
0.8
Yobs
0.0
0.2
0.4
0.6
0.8
1.0

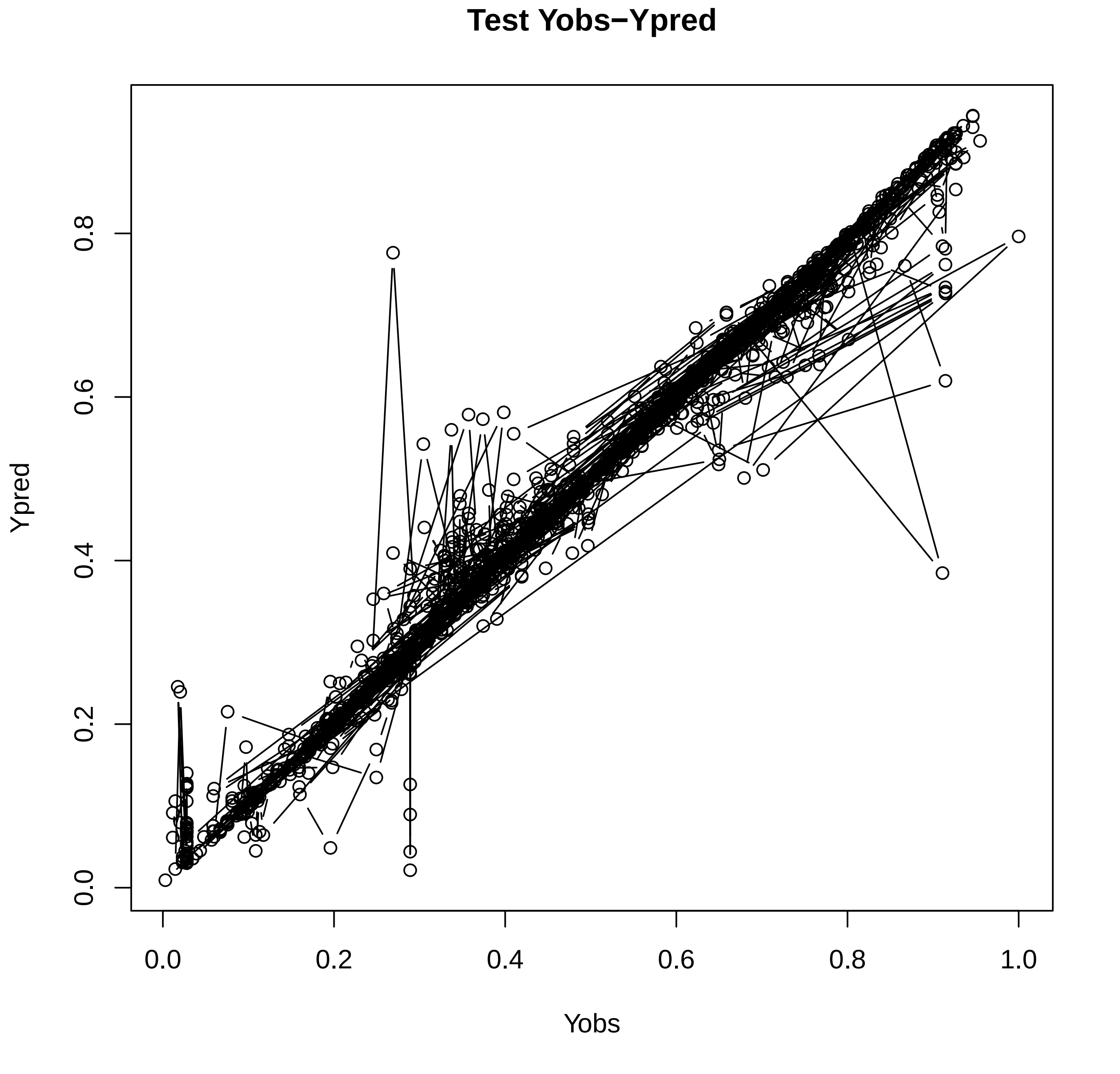

Test Yobs−Ypred
Ypred
Yobs
0.0
0.2
0.4
0.6
0.8
1.0

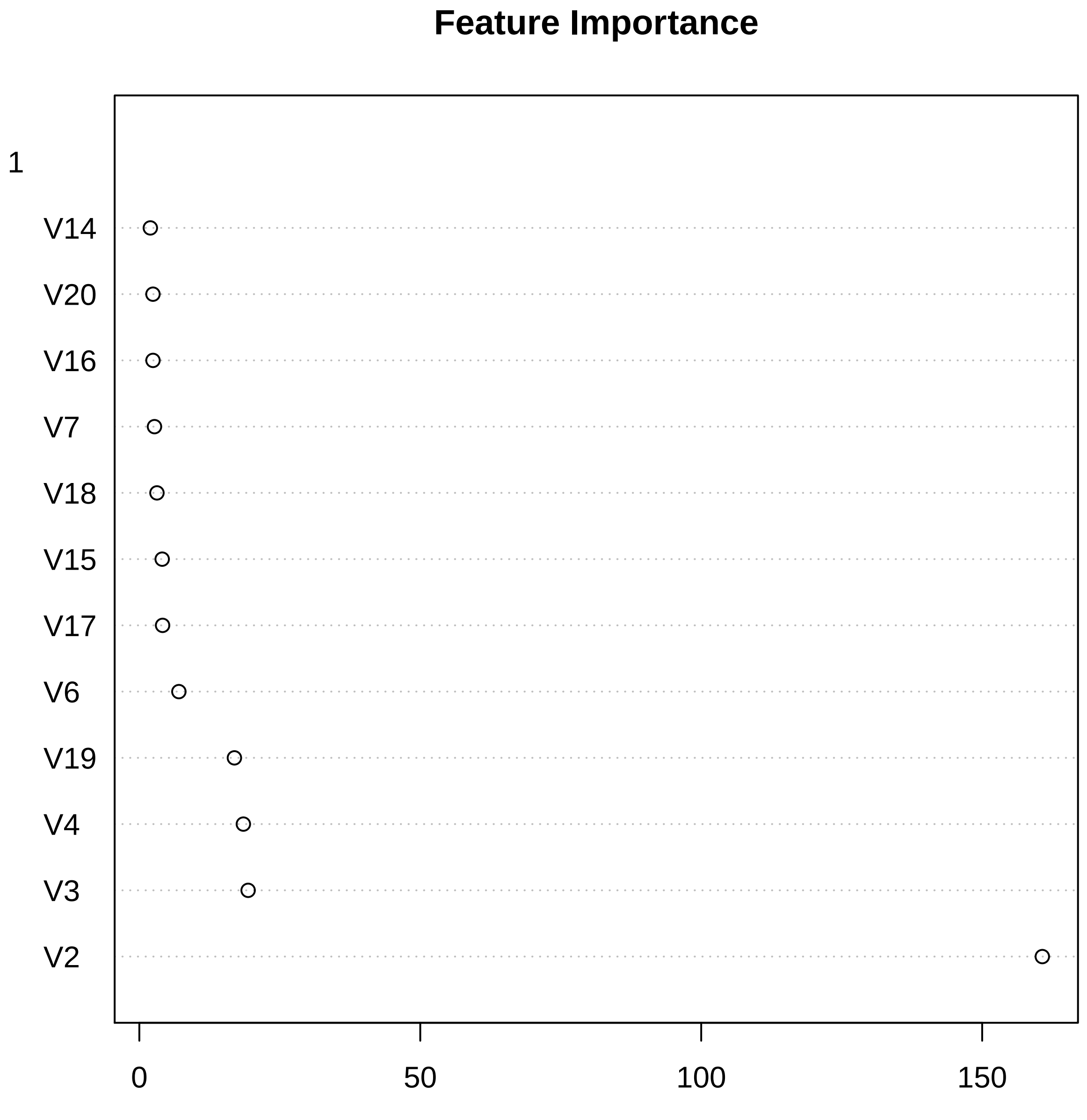
Feature Importance
1
V14
V20
V16
V7
V18
V15
V17
V6
V19
V4
V3
V2
0
50
100
150

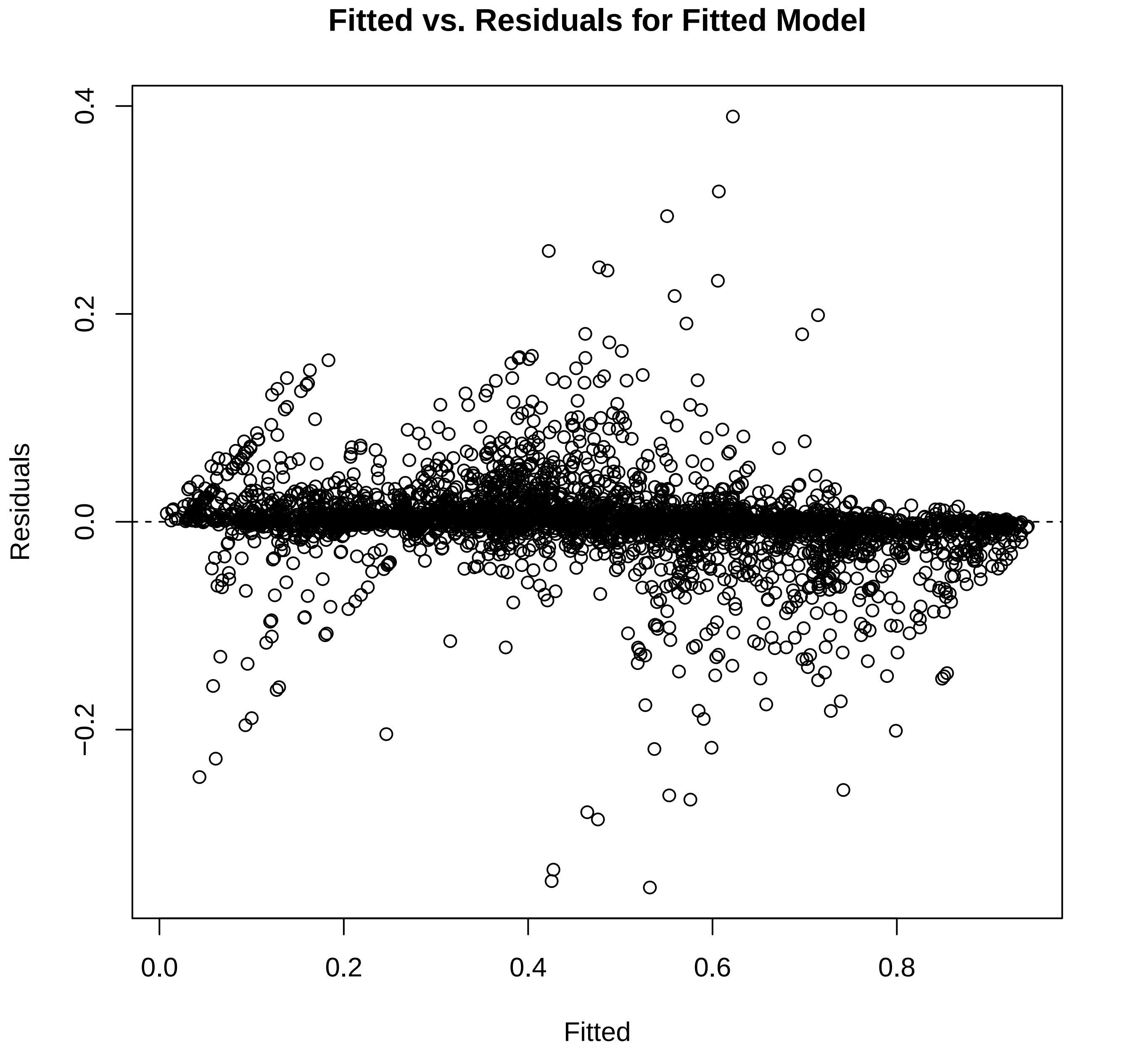

Fitted vs. Residuals for Fitted Model
Residuals
0.4
0.2
0.0
−0.2
Fitted
0.0
0.2
0.4
0.6
0.8

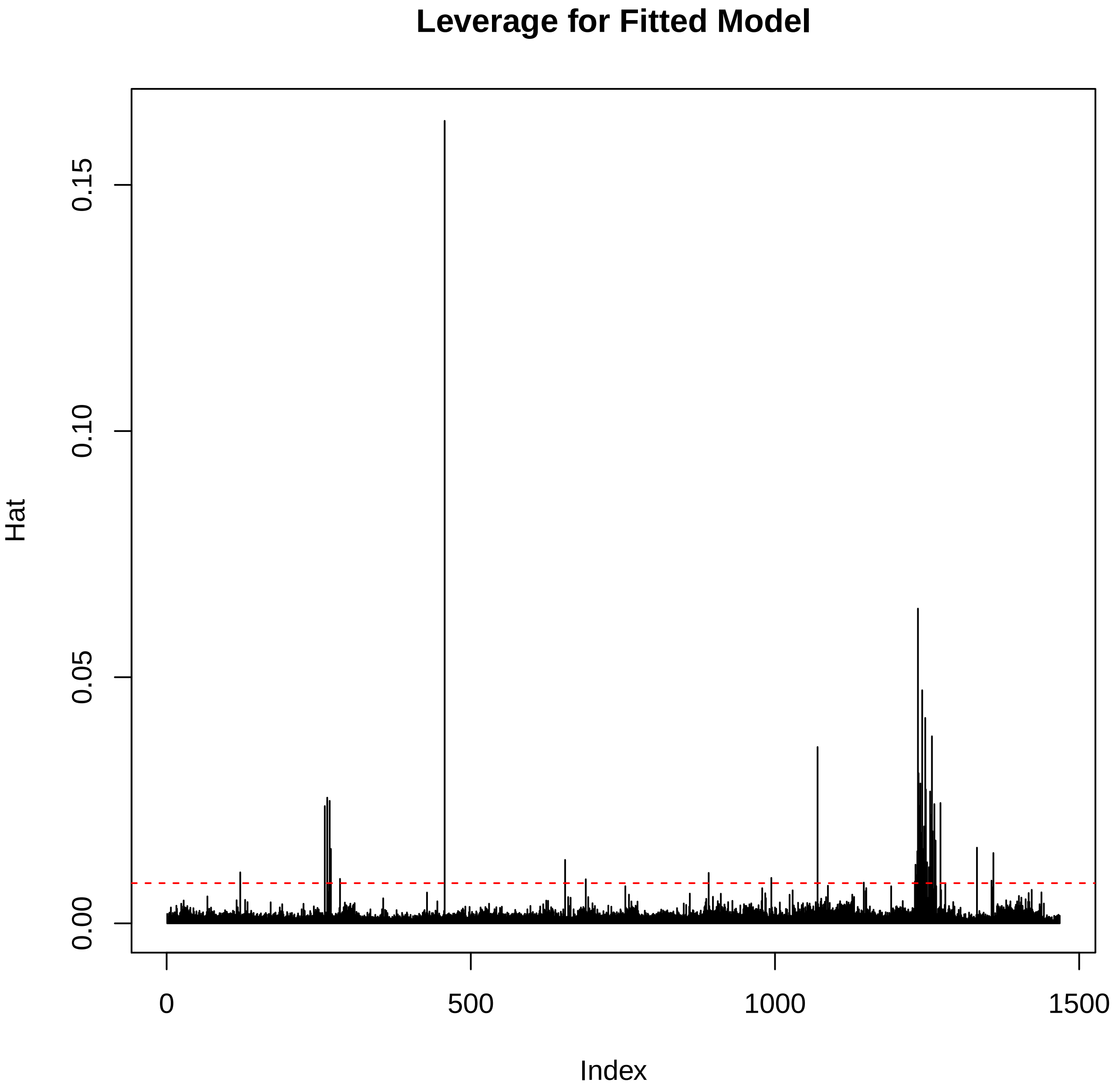

Leverage for Fitted Model
Hat
0.00
0.05
0.10
0.15
Index
0
500
1000
1500
