# Supplementary figures and images for: A methodology for the design of experiments in computational intelligence with multiple regression models (part 1 of 2)
Source: PeerJ. 2016 Dec 1;4:e2721. doi: 10.7717/peerj.2721 (PMC5136129; doi:10.7717/peerj.2721)

# Models' differences on the training set (data split 1)

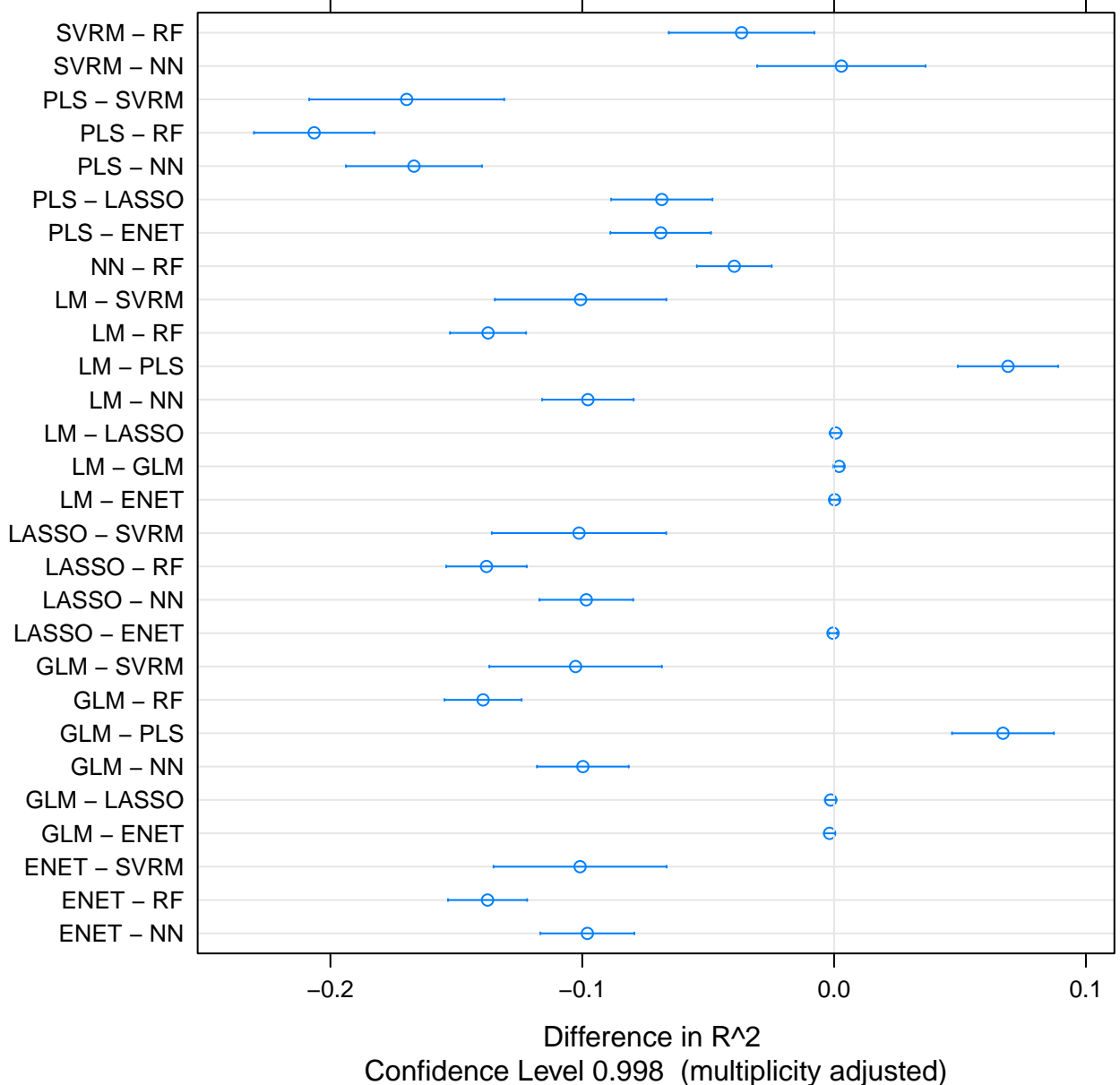

Supplement: Data S1 — Datailed results from UC Irvine Machine Learning Repository (Housing, Machine CPU, Wine Quality, Automobile and Parkinson) and the 3 Use Cases (Protein Corona, Gajewicz Metal Oxides and Aquatic Toxicity) [file peerj-04-2721-s001.zip › 1_housing/DifModels.R2.iSplits.1.pdf]

# Models' differences on the training set (data split 10)

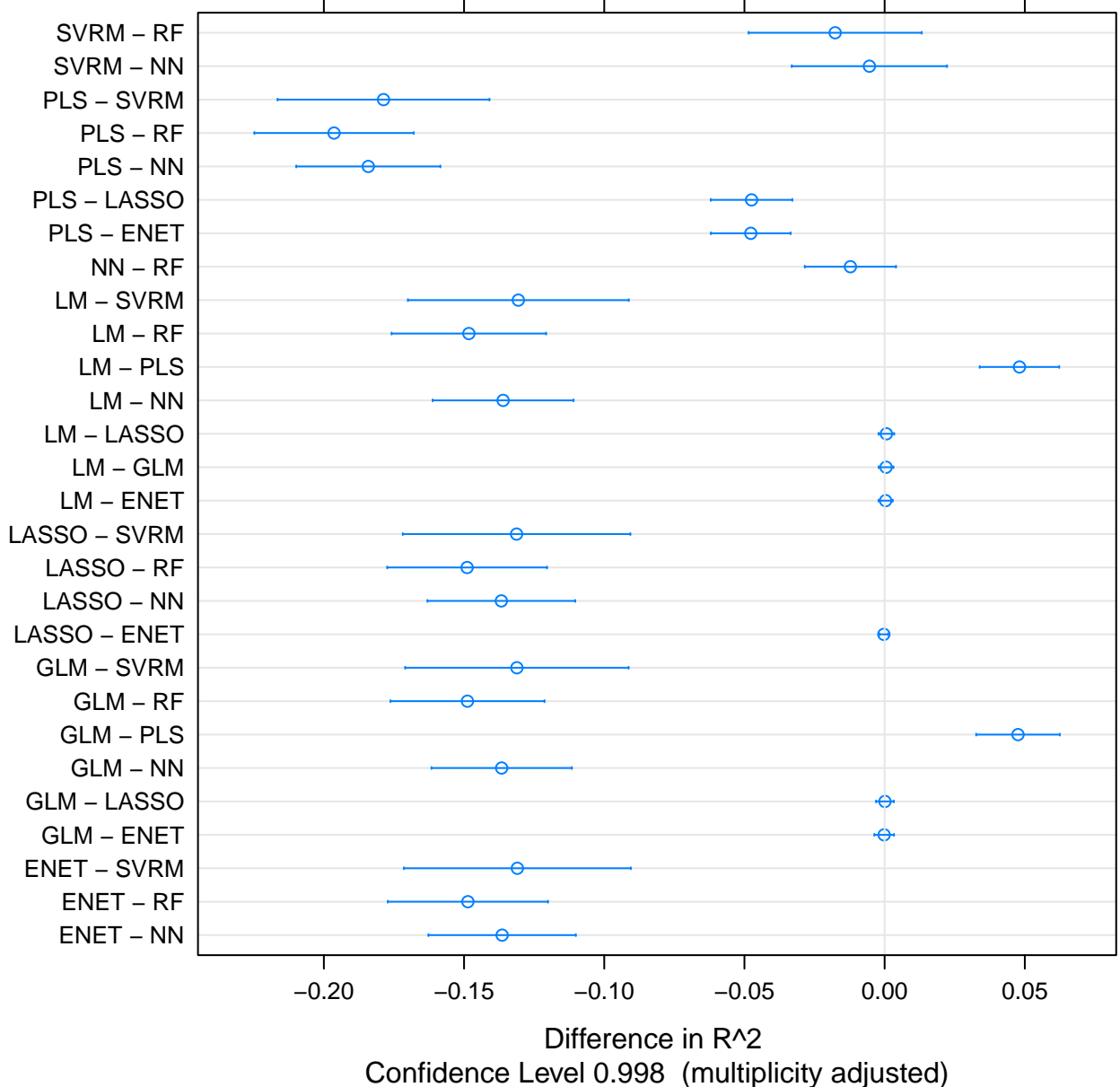

Supplement: Data S1 — Datailed results from UC Irvine Machine Learning Repository (Housing, Machine CPU, Wine Quality, Automobile and Parkinson) and the 3 Use Cases (Protein Corona, Gajewicz Metal Oxides and Aquatic Toxicity) [file peerj-04-2721-s001.zip › 1_housing/DifModels.R2.iSplits.10.pdf]

# Models' differences on the training set (data split 2)

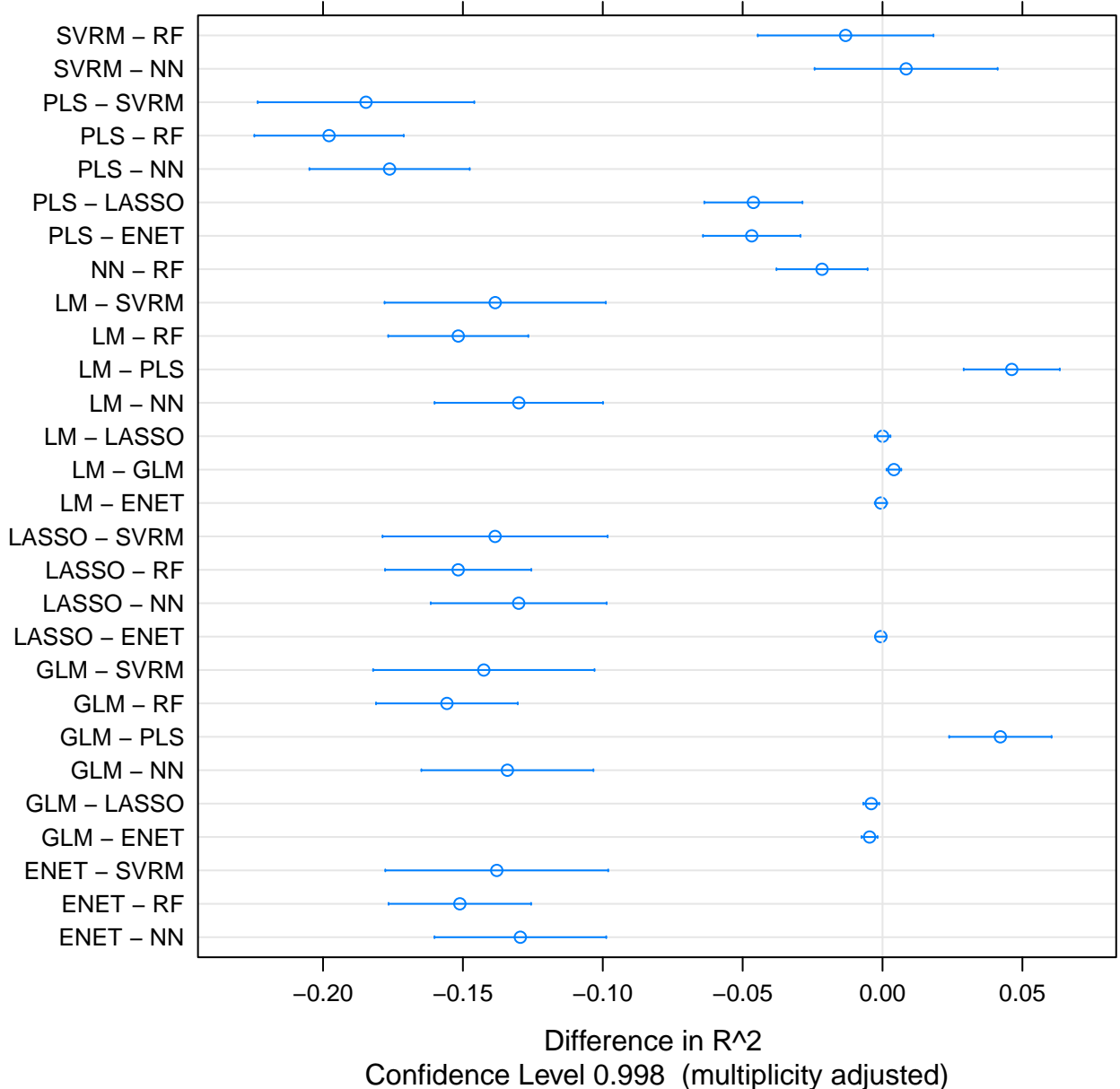

Supplement: Data S1 — Datailed results from UC Irvine Machine Learning Repository (Housing, Machine CPU, Wine Quality, Automobile and Parkinson) and the 3 Use Cases (Protein Corona, Gajewicz Metal Oxides and Aquatic Toxicity) [file peerj-04-2721-s001.zip › 1_housing/DifModels.R2.iSplits.2.pdf]

# Models' differences on the training set (data split 3)

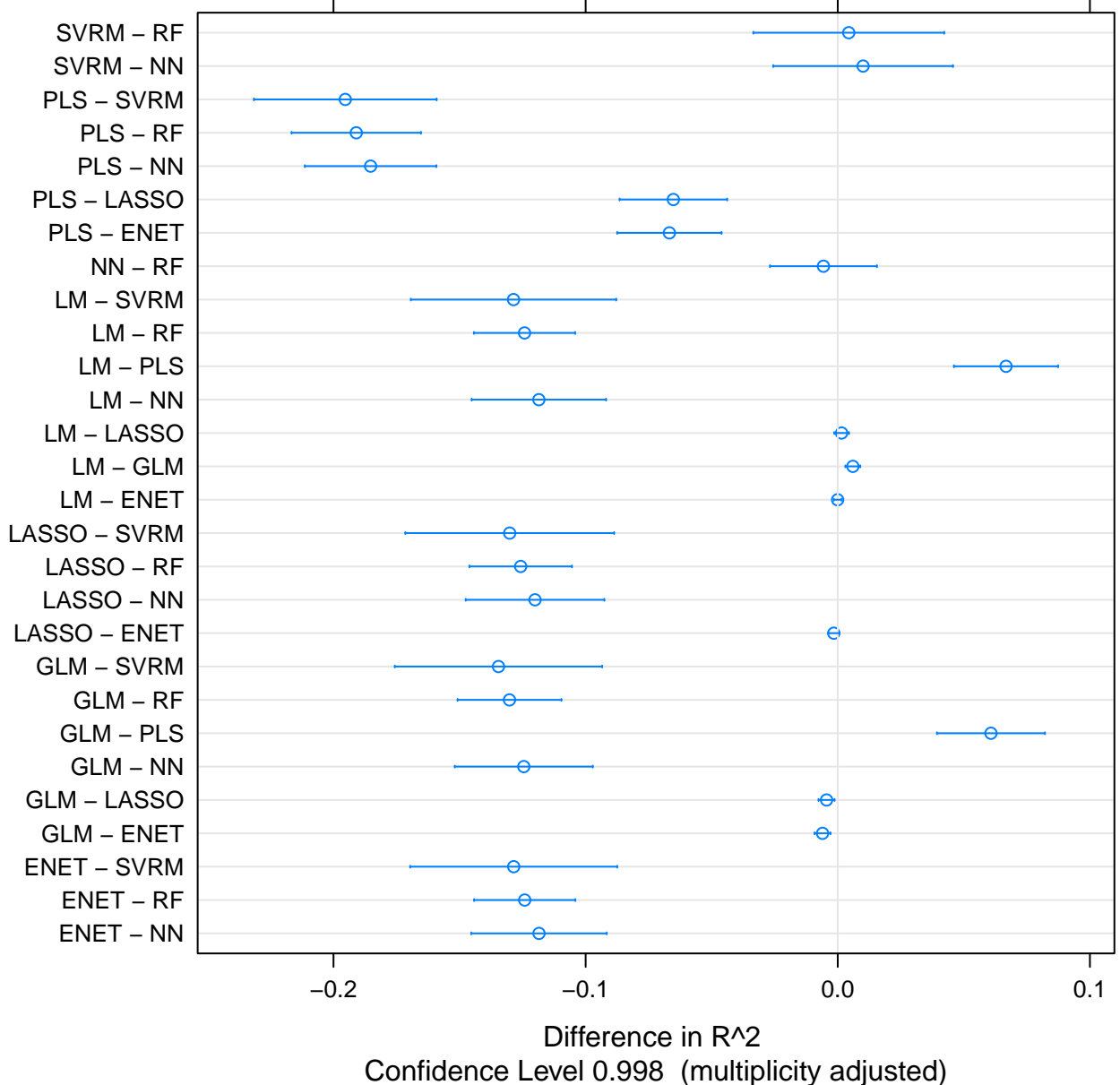

Supplement: Data S1 — Datailed results from UC Irvine Machine Learning Repository (Housing, Machine CPU, Wine Quality, Automobile and Parkinson) and the 3 Use Cases (Protein Corona, Gajewicz Metal Oxides and Aquatic Toxicity) [file peerj-04-2721-s001.zip › 1_housing/DifModels.R2.iSplits.3.pdf]

# Models' differences on the training set (data split 4)

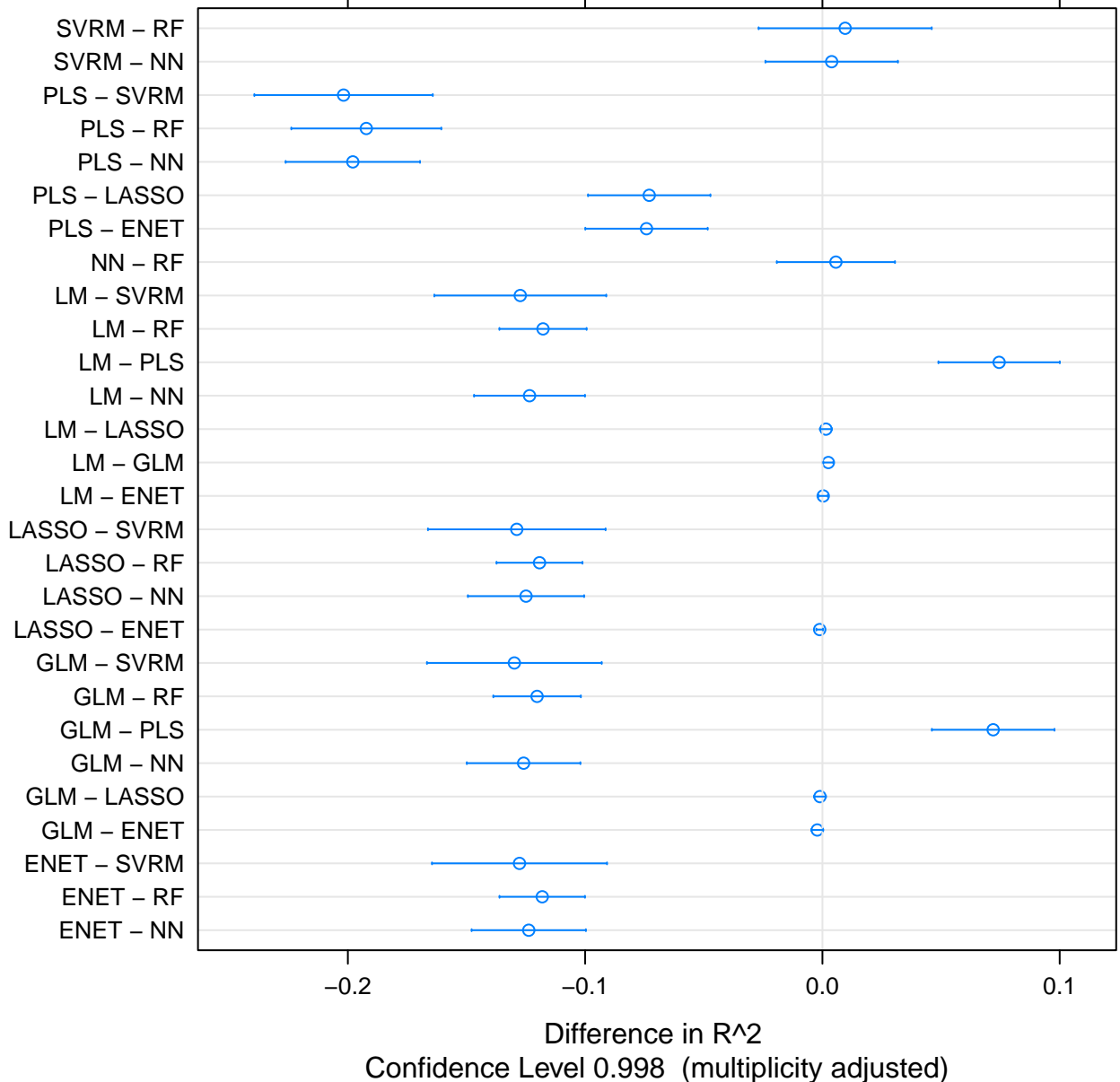

Supplement: Data S1 — Datailed results from UC Irvine Machine Learning Repository (Housing, Machine CPU, Wine Quality, Automobile and Parkinson) and the 3 Use Cases (Protein Corona, Gajewicz Metal Oxides and Aquatic Toxicity) [file peerj-04-2721-s001.zip › 1_housing/DifModels.R2.iSplits.4.pdf]

# Models' differences on the training set (data split 5)

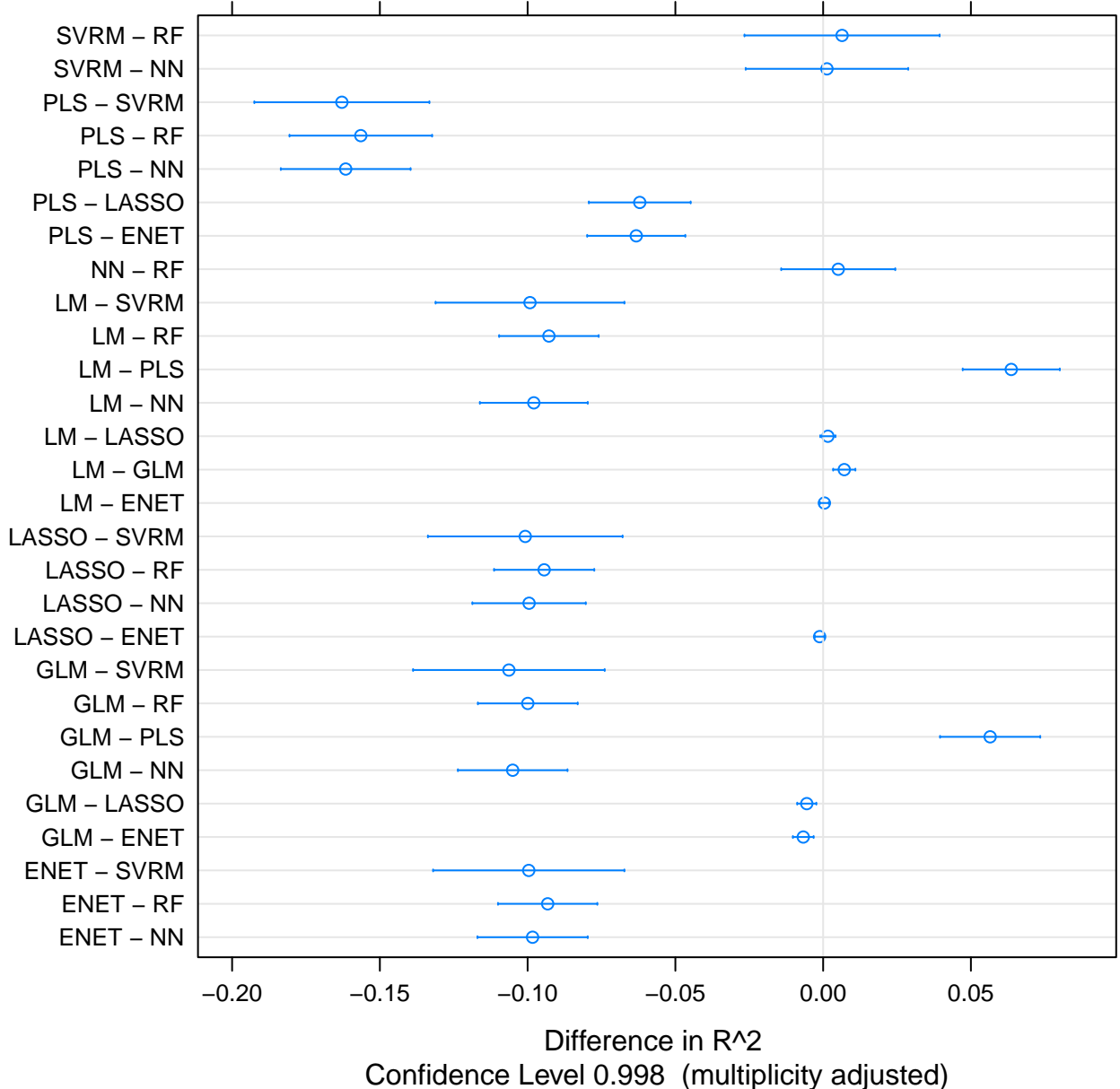

Supplement: Data S1 — Datailed results from UC Irvine Machine Learning Repository (Housing, Machine CPU, Wine Quality, Automobile and Parkinson) and the 3 Use Cases (Protein Corona, Gajewicz Metal Oxides and Aquatic Toxicity) [file peerj-04-2721-s001.zip › 1_housing/DifModels.R2.iSplits.5.pdf]

# Models' differences on the training set (data split 6)

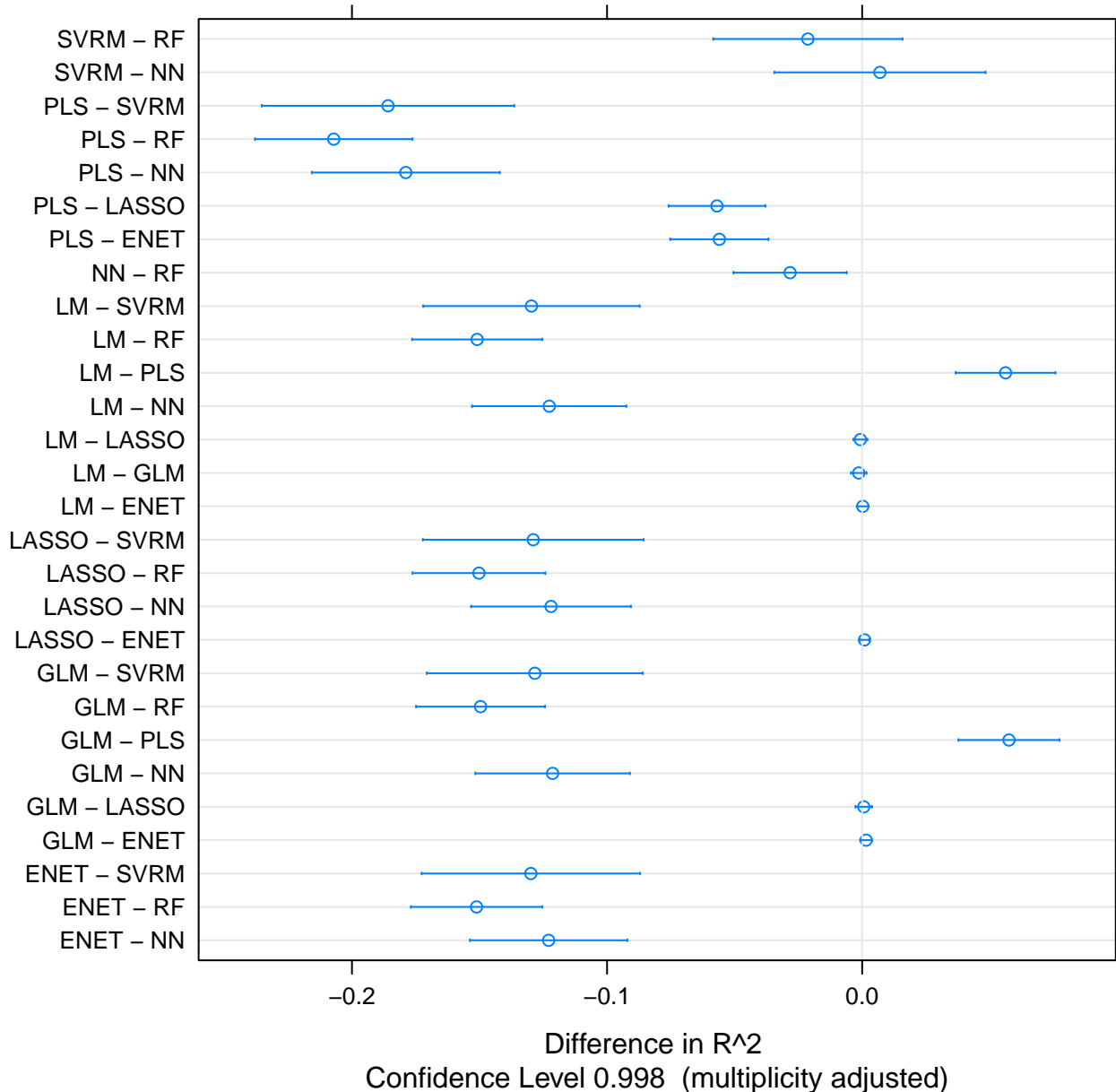

Supplement: Data S1 — Datailed results from UC Irvine Machine Learning Repository (Housing, Machine CPU, Wine Quality, Automobile and Parkinson) and the 3 Use Cases (Protein Corona, Gajewicz Metal Oxides and Aquatic Toxicity) [file peerj-04-2721-s001.zip › 1_housing/DifModels.R2.iSplits.6.pdf]

# Models' differences on the training set (data split 7)

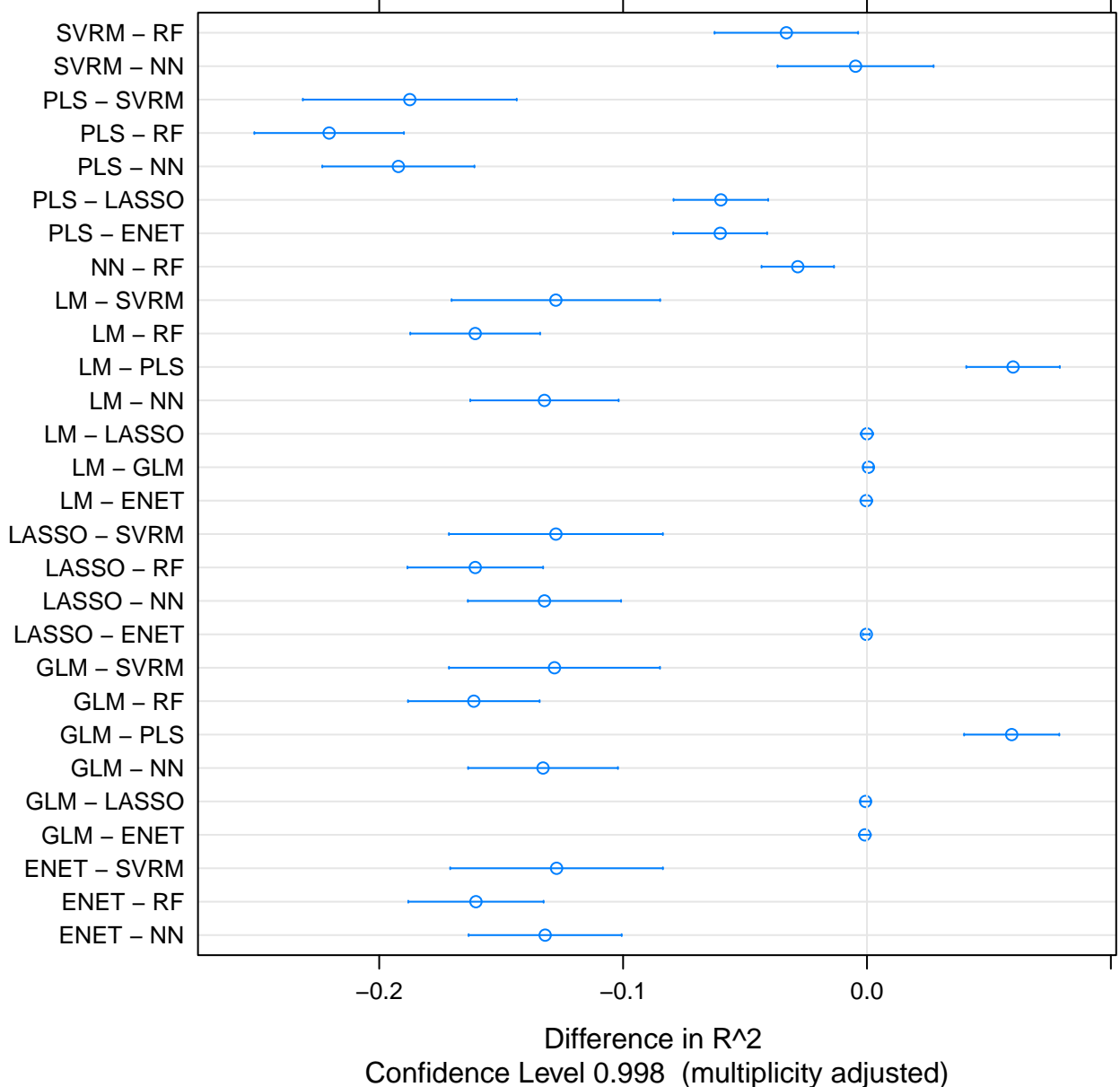

Supplement: Data S1 — Datailed results from UC Irvine Machine Learning Repository (Housing, Machine CPU, Wine Quality, Automobile and Parkinson) and the 3 Use Cases (Protein Corona, Gajewicz Metal Oxides and Aquatic Toxicity) [file peerj-04-2721-s001.zip › 1_housing/DifModels.R2.iSplits.7.pdf]

# Models' differences on the training set (data split 8)

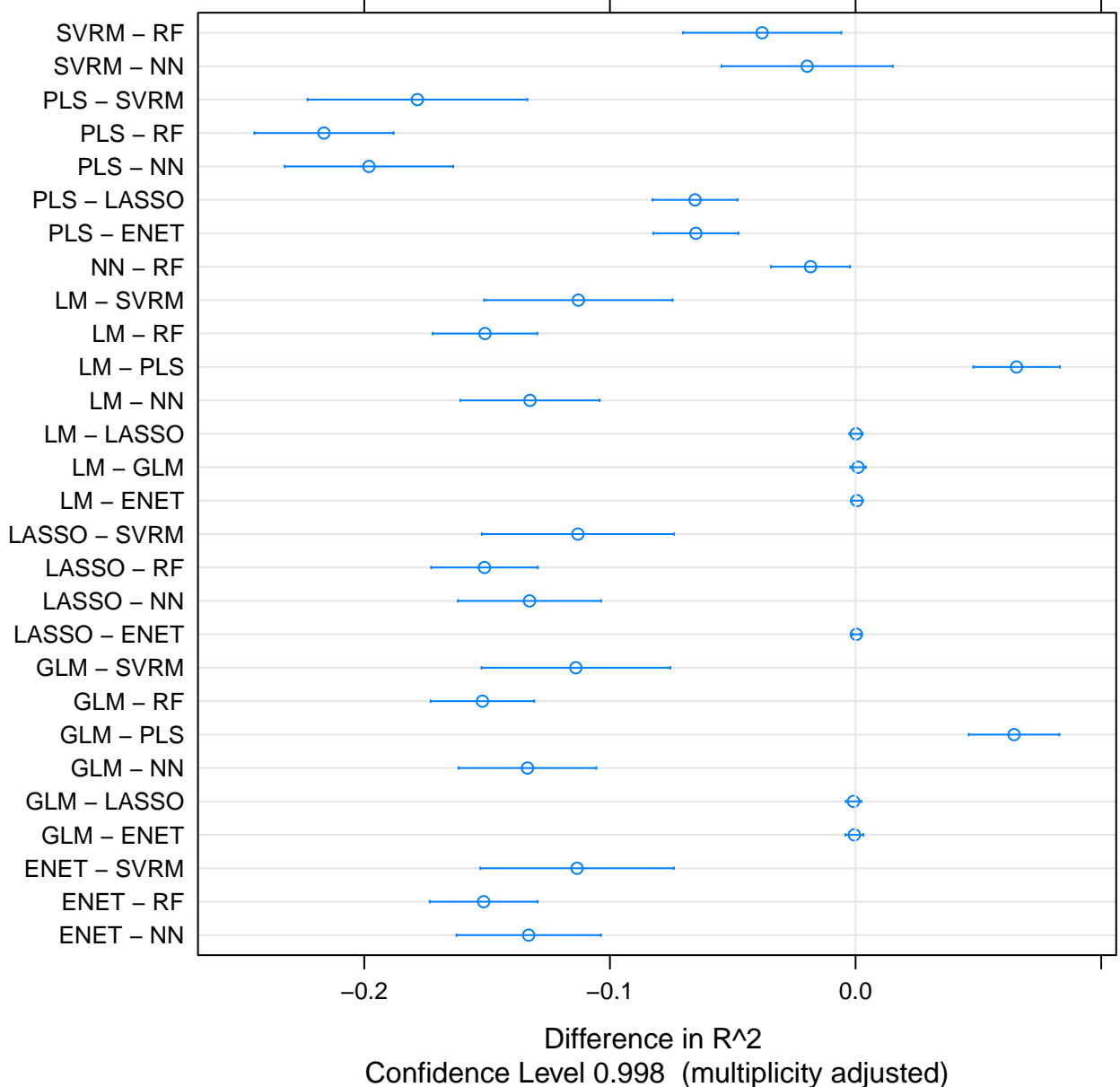

Supplement: Data S1 — Datailed results from UC Irvine Machine Learning Repository (Housing, Machine CPU, Wine Quality, Automobile and Parkinson) and the 3 Use Cases (Protein Corona, Gajewicz Metal Oxides and Aquatic Toxicity) [file peerj-04-2721-s001.zip › 1_housing/DifModels.R2.iSplits.8.pdf]

# Models' differences on the training set (data split 9)

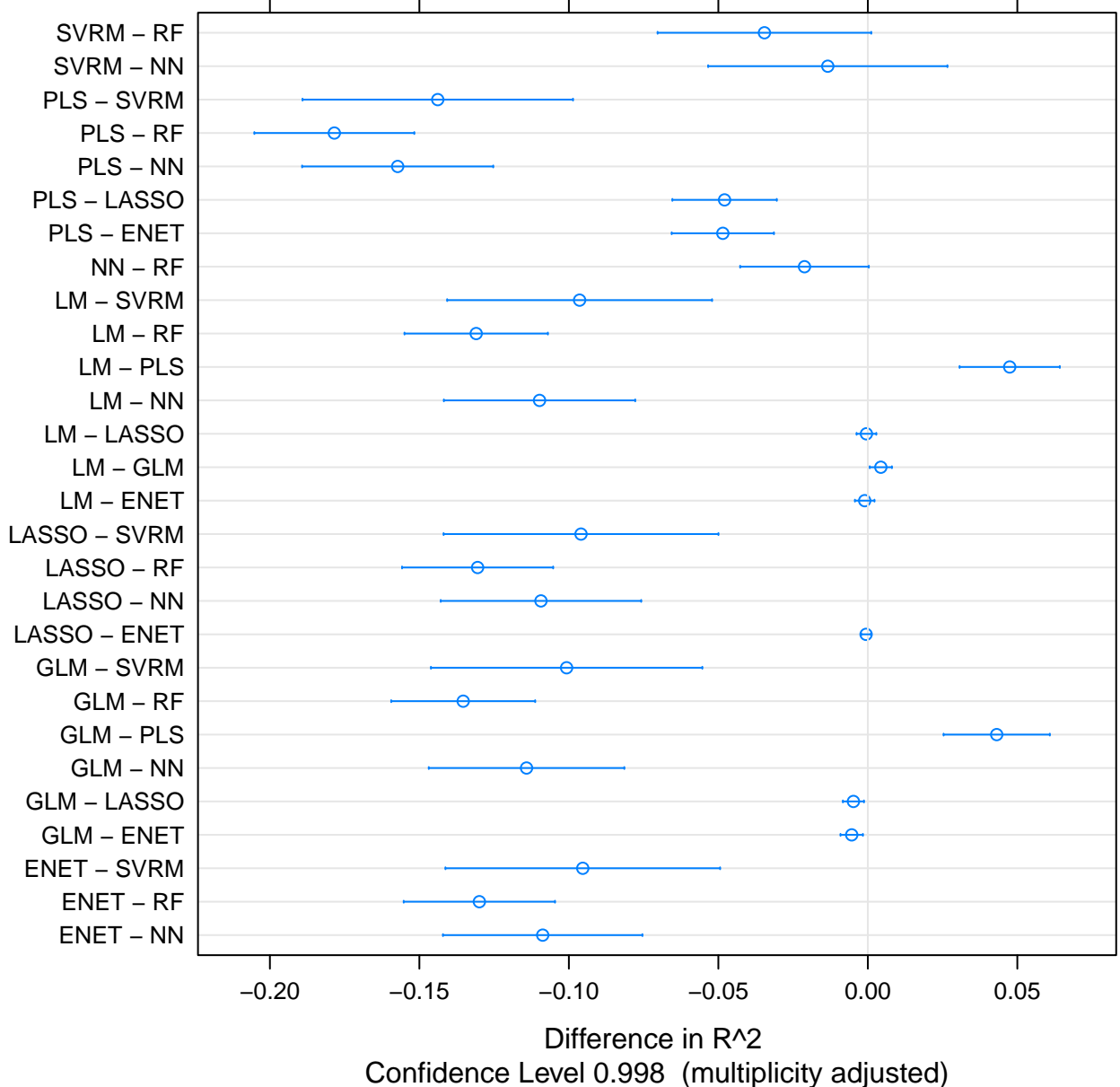

Supplement: Data S1 — Datailed results from UC Irvine Machine Learning Repository (Housing, Machine CPU, Wine Quality, Automobile and Parkinson) and the 3 Use Cases (Protein Corona, Gajewicz Metal Oxides and Aquatic Toxicity) [file peerj-04-2721-s001.zip › 1_housing/DifModels.R2.iSplits.9.pdf]

# Models' differences on the training set (data split 1)

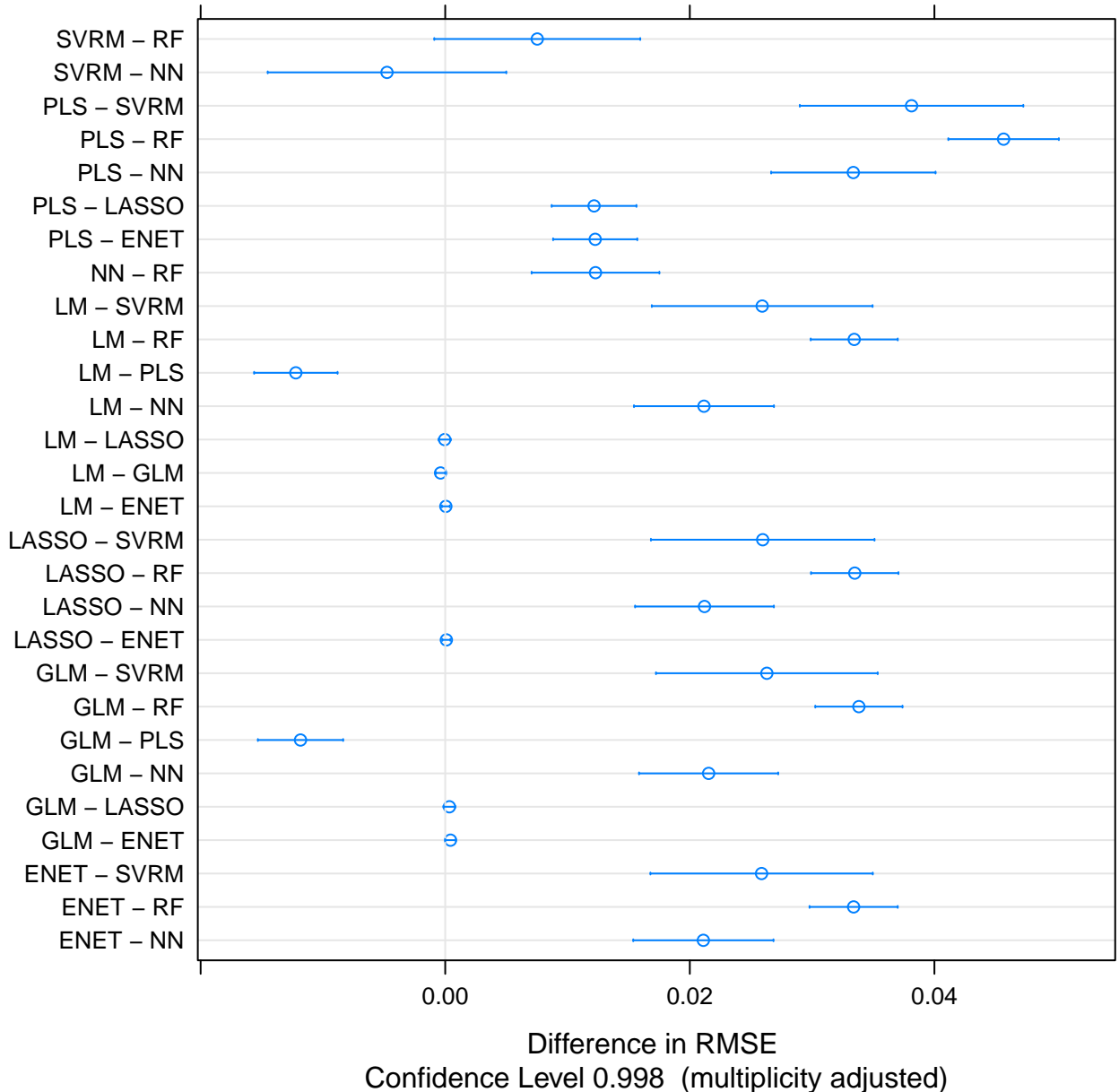

Supplement: Data S1 — Datailed results from UC Irvine Machine Learning Repository (Housing, Machine CPU, Wine Quality, Automobile and Parkinson) and the 3 Use Cases (Protein Corona, Gajewicz Metal Oxides and Aquatic Toxicity) [file peerj-04-2721-s001.zip › 1_housing/DifModels.RMSE.iSplits.1.pdf]

# Models' differences on the training set (data split 10)

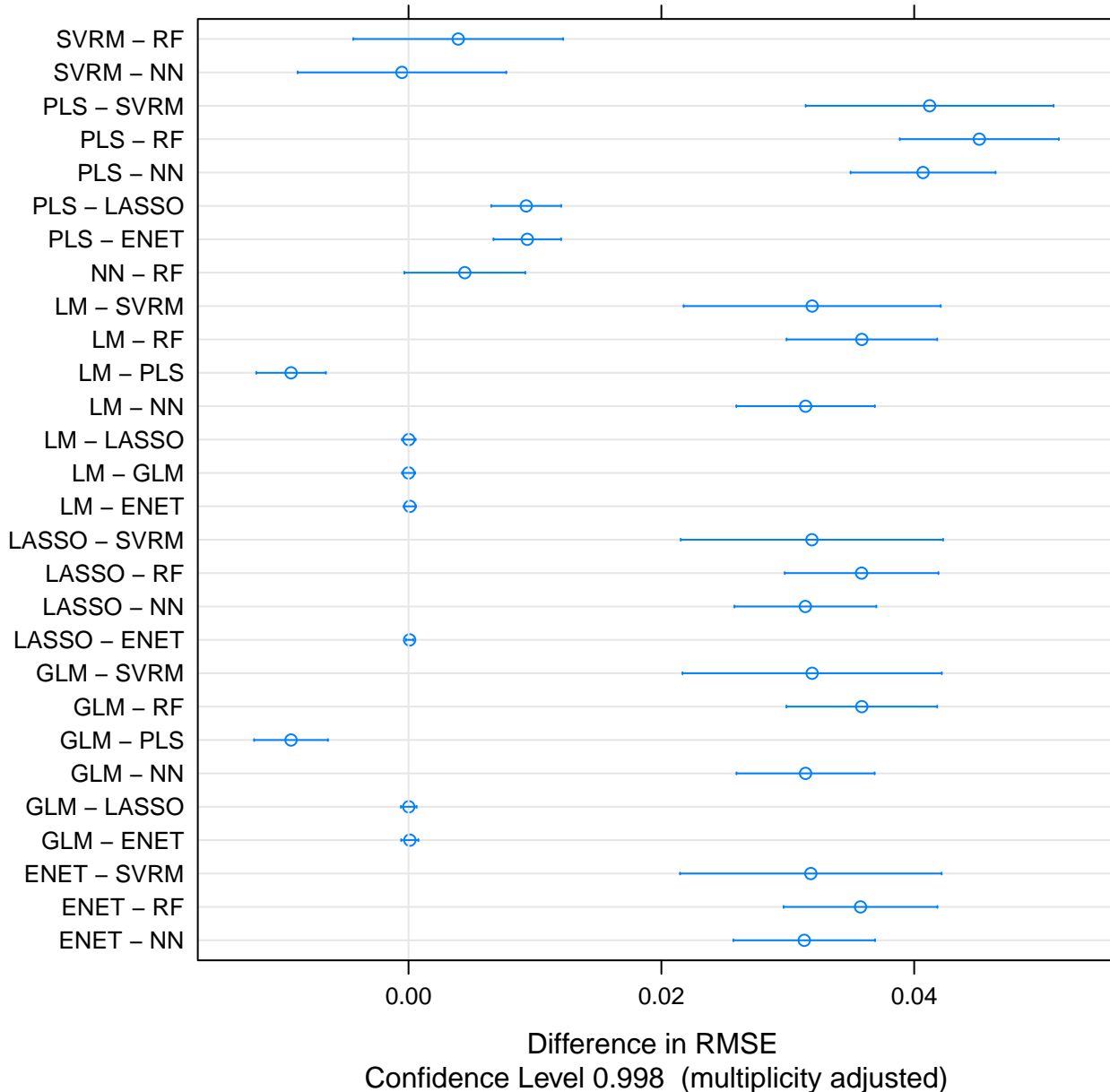

Supplement: Data S1 — Datailed results from UC Irvine Machine Learning Repository (Housing, Machine CPU, Wine Quality, Automobile and Parkinson) and the 3 Use Cases (Protein Corona, Gajewicz Metal Oxides and Aquatic Toxicity) [file peerj-04-2721-s001.zip › 1_housing/DifModels.RMSE.iSplits.10.pdf]

# Models' differences on the training set (data split 2)

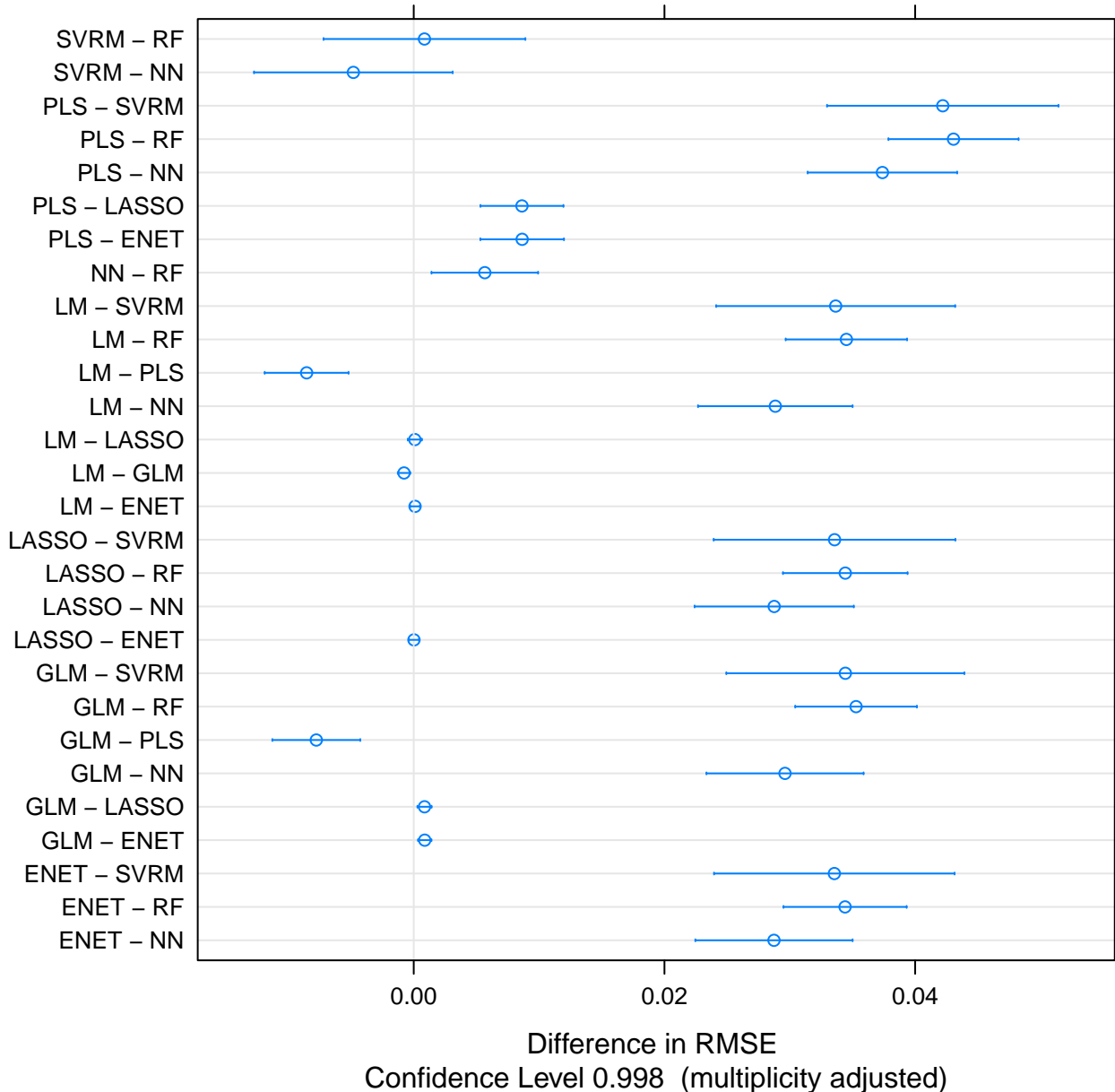

Supplement: Data S1 — Datailed results from UC Irvine Machine Learning Repository (Housing, Machine CPU, Wine Quality, Automobile and Parkinson) and the 3 Use Cases (Protein Corona, Gajewicz Metal Oxides and Aquatic Toxicity) [file peerj-04-2721-s001.zip › 1_housing/DifModels.RMSE.iSplits.2.pdf]

# Models' differences on the training set (data split 3)

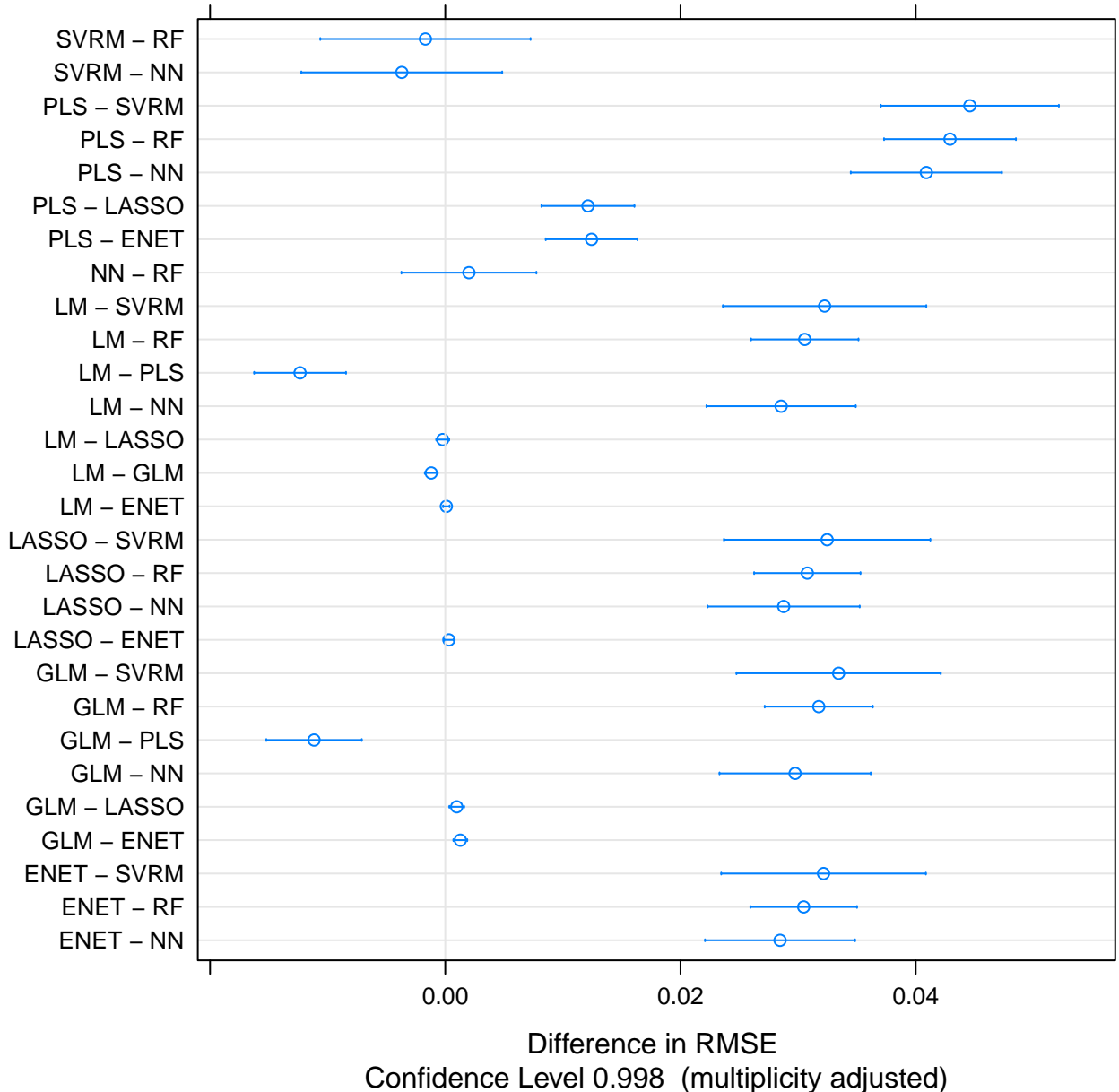

Supplement: Data S1 — Datailed results from UC Irvine Machine Learning Repository (Housing, Machine CPU, Wine Quality, Automobile and Parkinson) and the 3 Use Cases (Protein Corona, Gajewicz Metal Oxides and Aquatic Toxicity) [file peerj-04-2721-s001.zip › 1_housing/DifModels.RMSE.iSplits.3.pdf]

# Models' differences on the training set (data split 4)

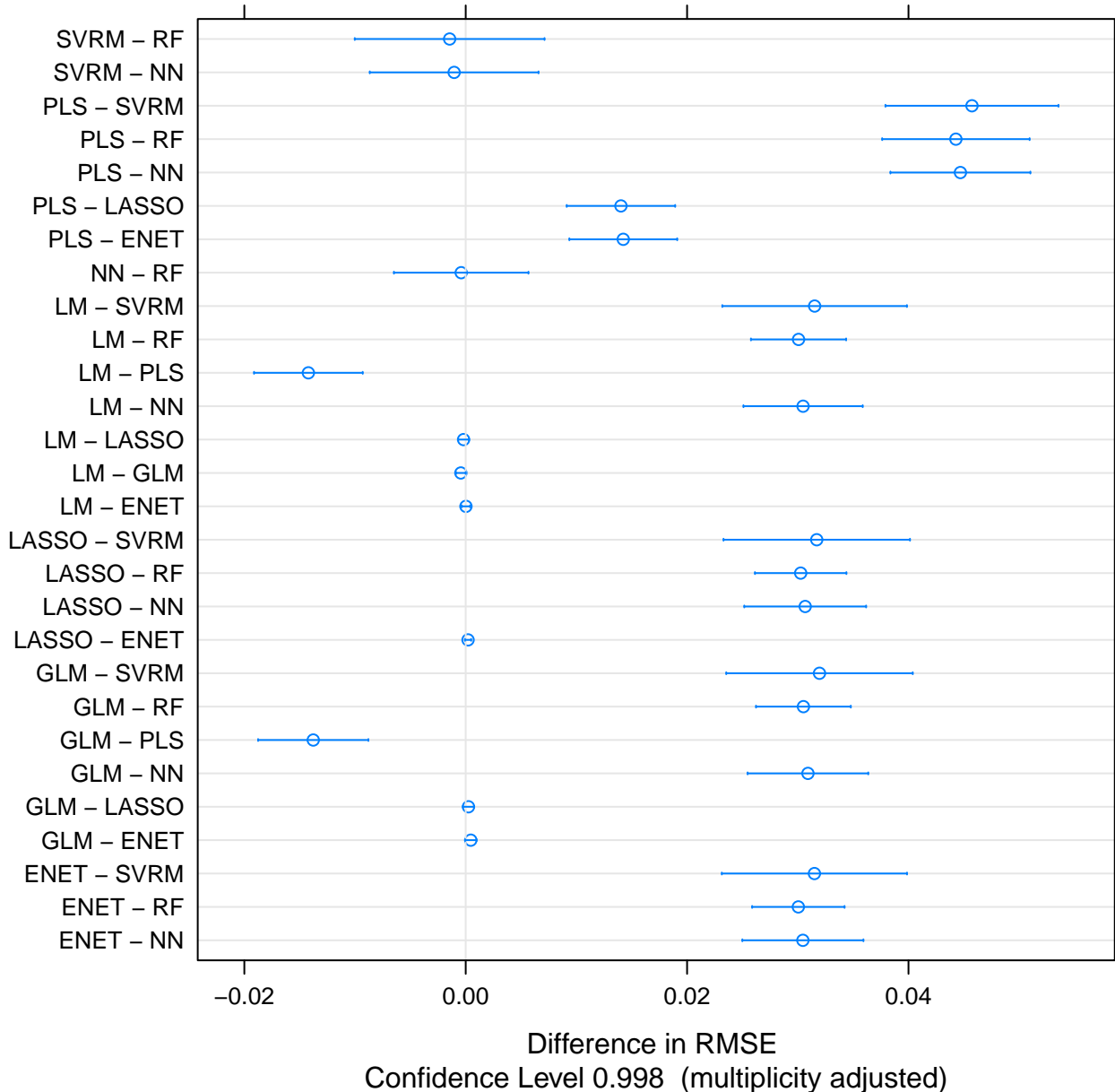

Supplement: Data S1 — Datailed results from UC Irvine Machine Learning Repository (Housing, Machine CPU, Wine Quality, Automobile and Parkinson) and the 3 Use Cases (Protein Corona, Gajewicz Metal Oxides and Aquatic Toxicity) [file peerj-04-2721-s001.zip › 1_housing/DifModels.RMSE.iSplits.4.pdf]

# Models' differences on the training set (data split 5)

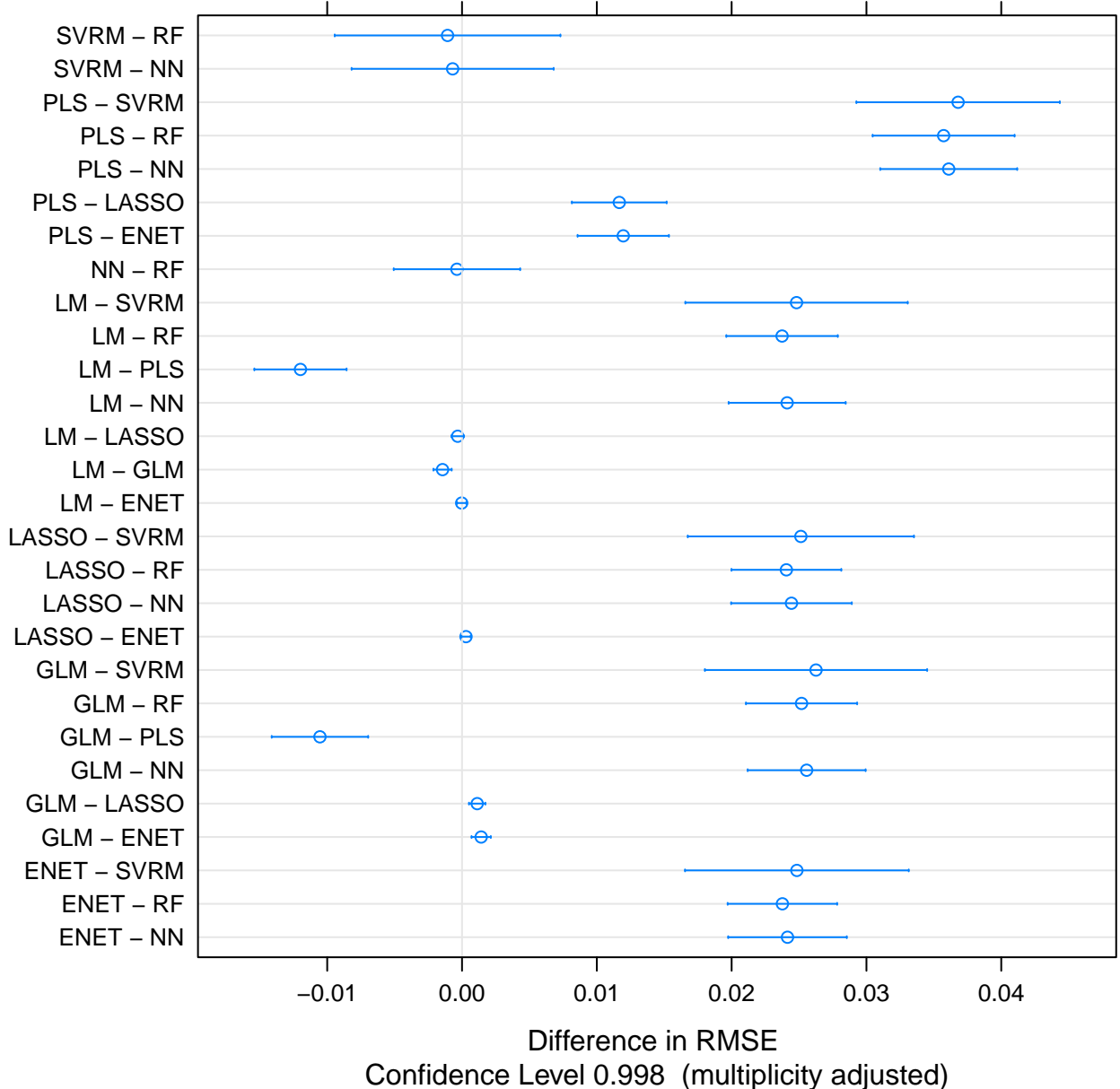

Supplement: Data S1 — Datailed results from UC Irvine Machine Learning Repository (Housing, Machine CPU, Wine Quality, Automobile and Parkinson) and the 3 Use Cases (Protein Corona, Gajewicz Metal Oxides and Aquatic Toxicity) [file peerj-04-2721-s001.zip › 1_housing/DifModels.RMSE.iSplits.5.pdf]

# Models' differences on the training set (data split 6)

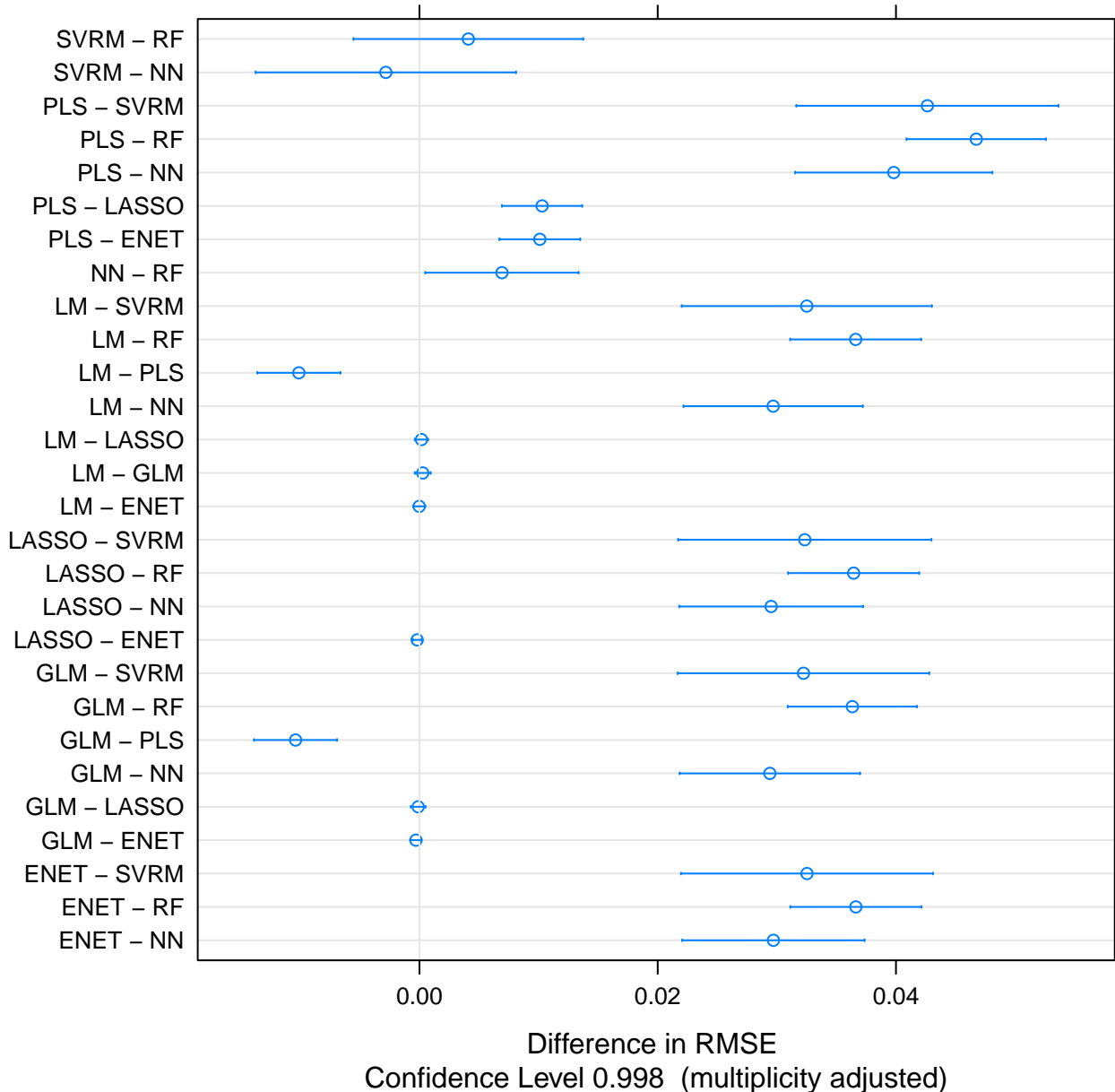

Supplement: Data S1 — Datailed results from UC Irvine Machine Learning Repository (Housing, Machine CPU, Wine Quality, Automobile and Parkinson) and the 3 Use Cases (Protein Corona, Gajewicz Metal Oxides and Aquatic Toxicity) [file peerj-04-2721-s001.zip › 1_housing/DifModels.RMSE.iSplits.6.pdf]

# Models' differences on the training set (data split 7)

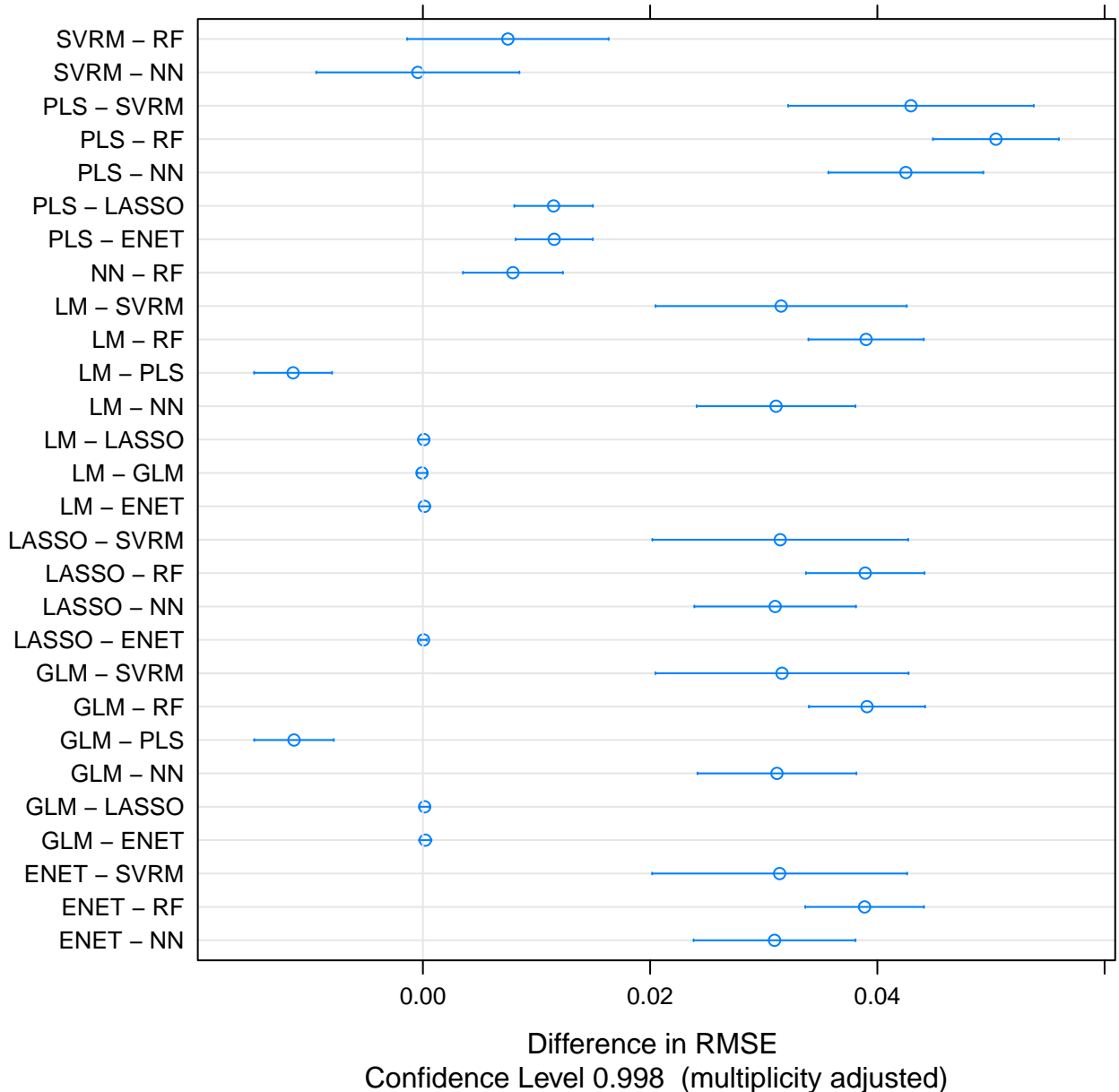

Supplement: Data S1 — Datailed results from UC Irvine Machine Learning Repository (Housing, Machine CPU, Wine Quality, Automobile and Parkinson) and the 3 Use Cases (Protein Corona, Gajewicz Metal Oxides and Aquatic Toxicity) [file peerj-04-2721-s001.zip › 1_housing/DifModels.RMSE.iSplits.7.pdf]

# Models' differences on the training set (data split 8)

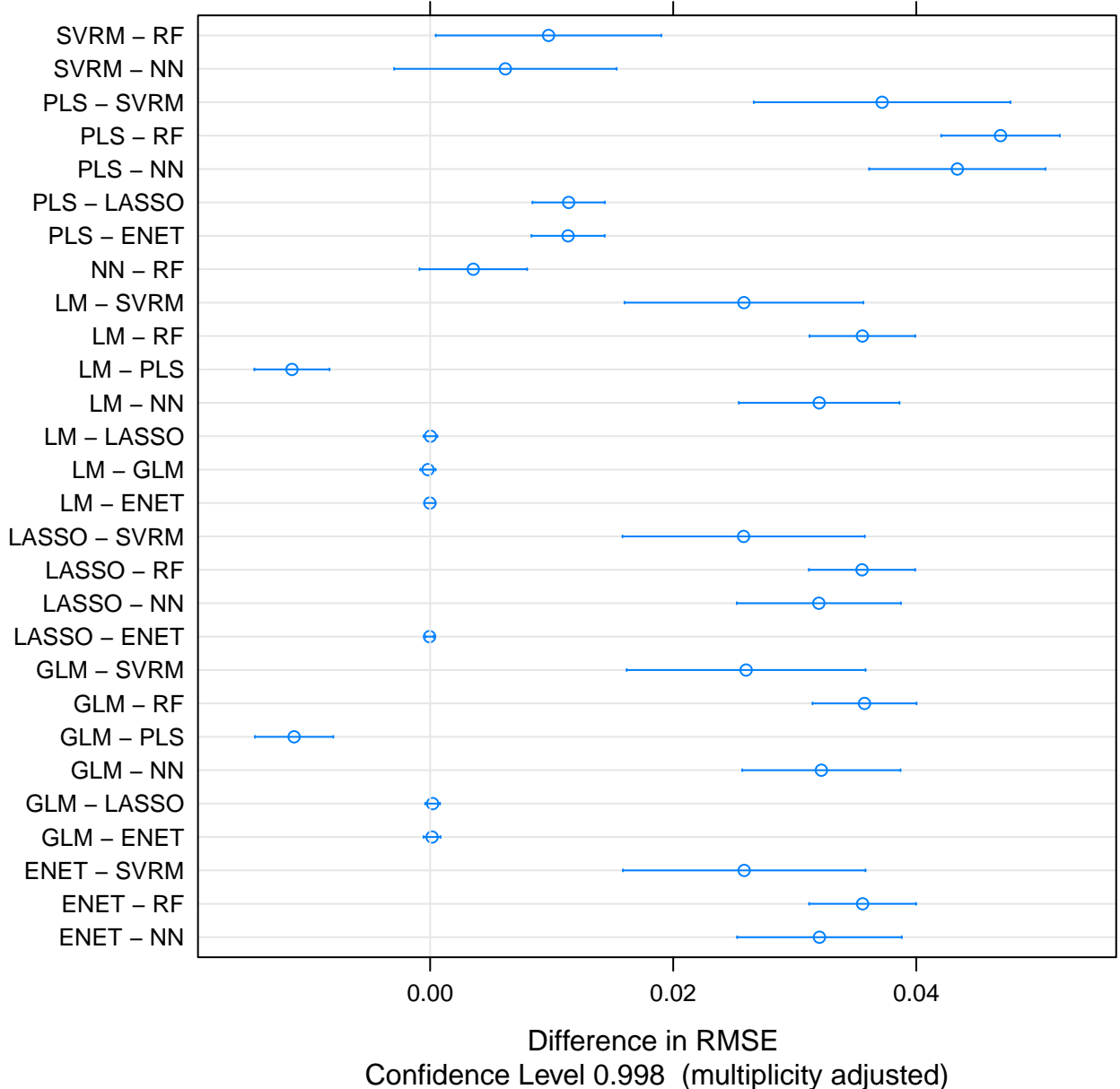

Supplement: Data S1 — Datailed results from UC Irvine Machine Learning Repository (Housing, Machine CPU, Wine Quality, Automobile and Parkinson) and the 3 Use Cases (Protein Corona, Gajewicz Metal Oxides and Aquatic Toxicity) [file peerj-04-2721-s001.zip › 1_housing/DifModels.RMSE.iSplits.8.pdf]

# Models' differences on the training set (data split 9)

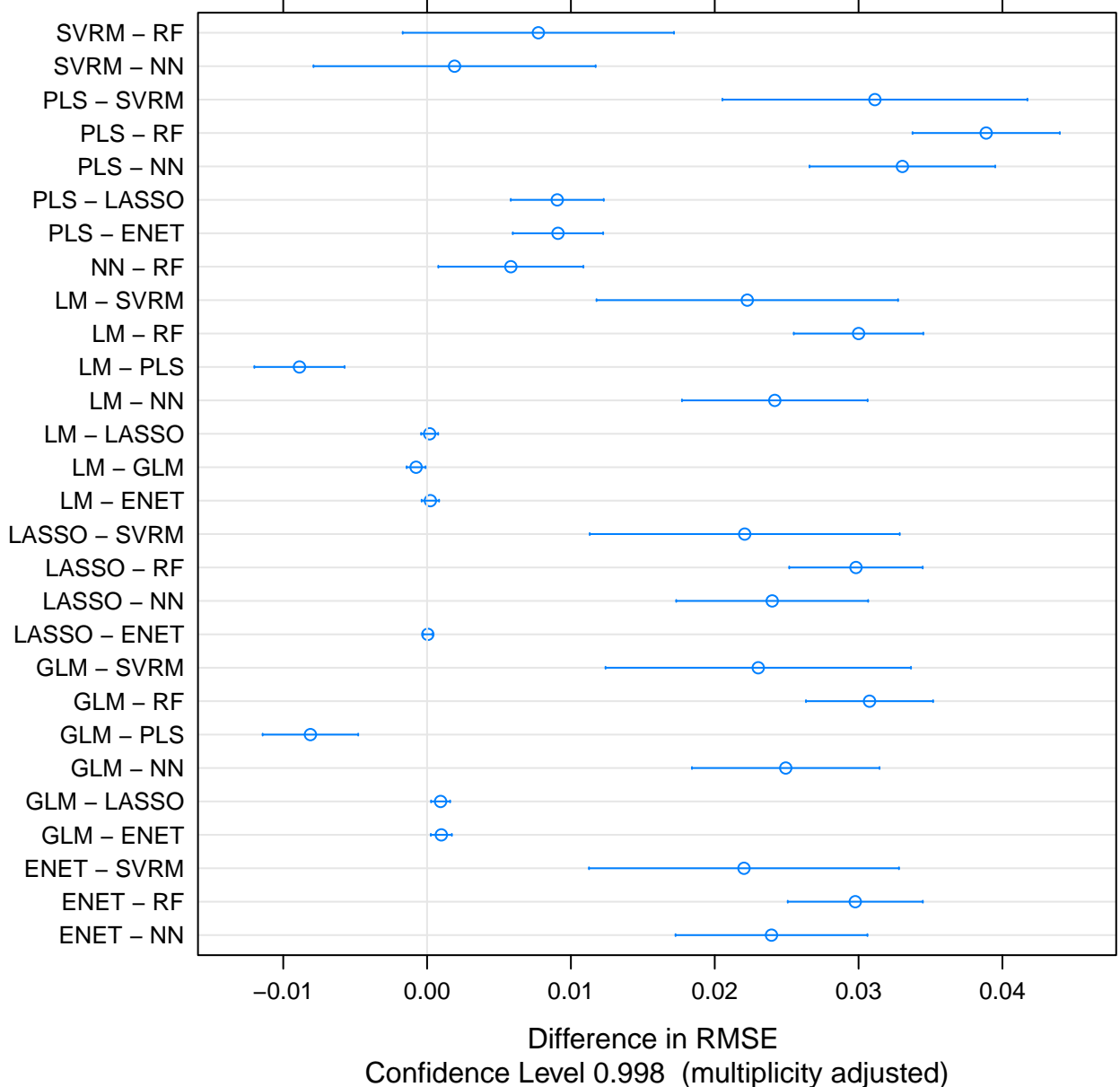

Supplement: Data S1 — Datailed results from UC Irvine Machine Learning Repository (Housing, Machine CPU, Wine Quality, Automobile and Parkinson) and the 3 Use Cases (Protein Corona, Gajewicz Metal Oxides and Aquatic Toxicity) [file peerj-04-2721-s001.zip › 1_housing/DifModels.RMSE.iSplits.9.pdf]

# Resampling results on the training set (data split 1)

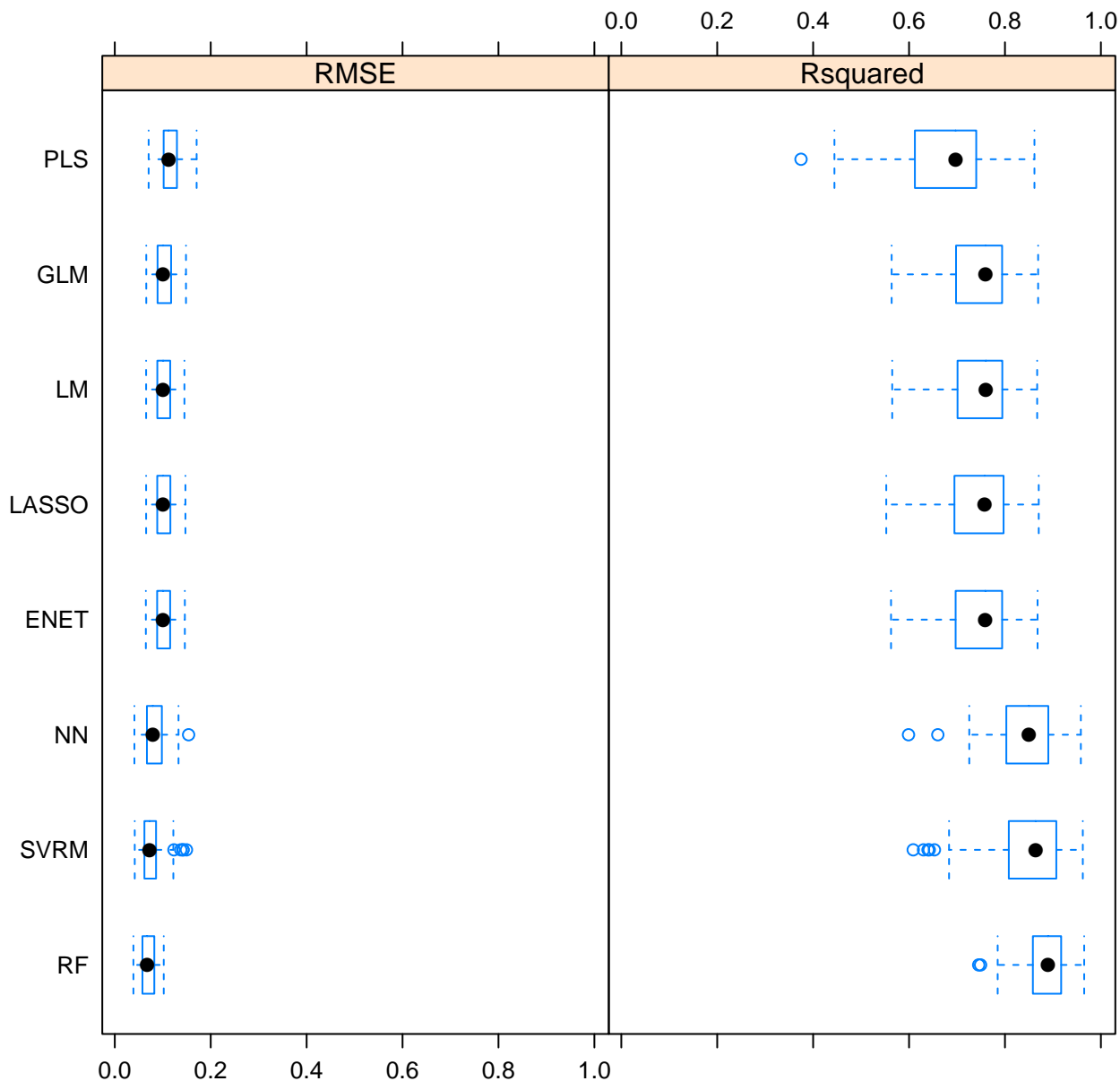

Supplement: Data S1 — Datailed results from UC Irvine Machine Learning Repository (Housing, Machine CPU, Wine Quality, Automobile and Parkinson) and the 3 Use Cases (Protein Corona, Gajewicz Metal Oxides and Aquatic Toxicity) [file peerj-04-2721-s001.zip › 1_housing/ModelsComp.iSplits.1.pdf]

# Resampling results on the training set (data split 10)

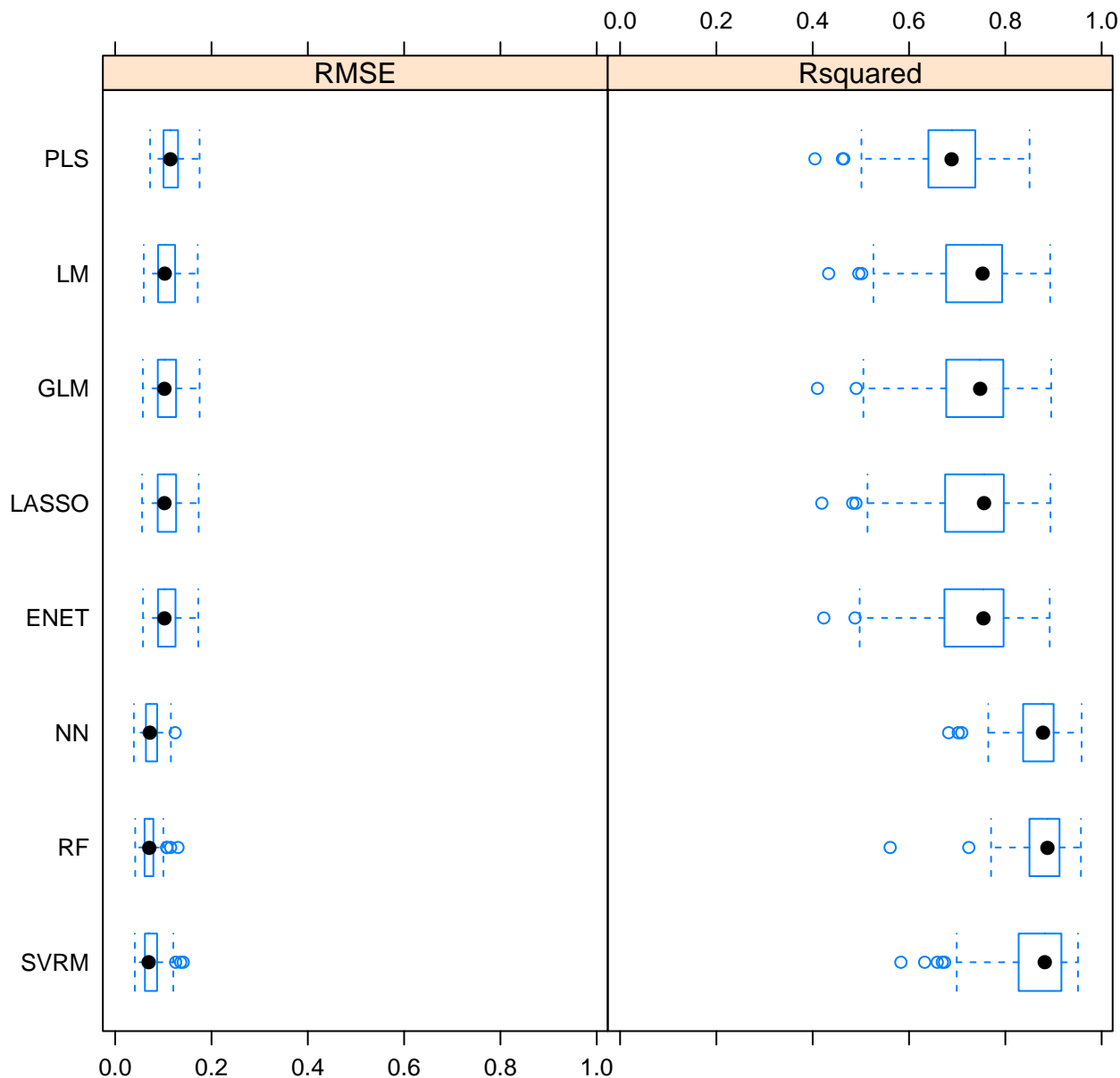

Supplement: Data S1 — Datailed results from UC Irvine Machine Learning Repository (Housing, Machine CPU, Wine Quality, Automobile and Parkinson) and the 3 Use Cases (Protein Corona, Gajewicz Metal Oxides and Aquatic Toxicity) [file peerj-04-2721-s001.zip › 1_housing/ModelsComp.iSplits.10.pdf]

# Resampling results on the training set (data split 2)

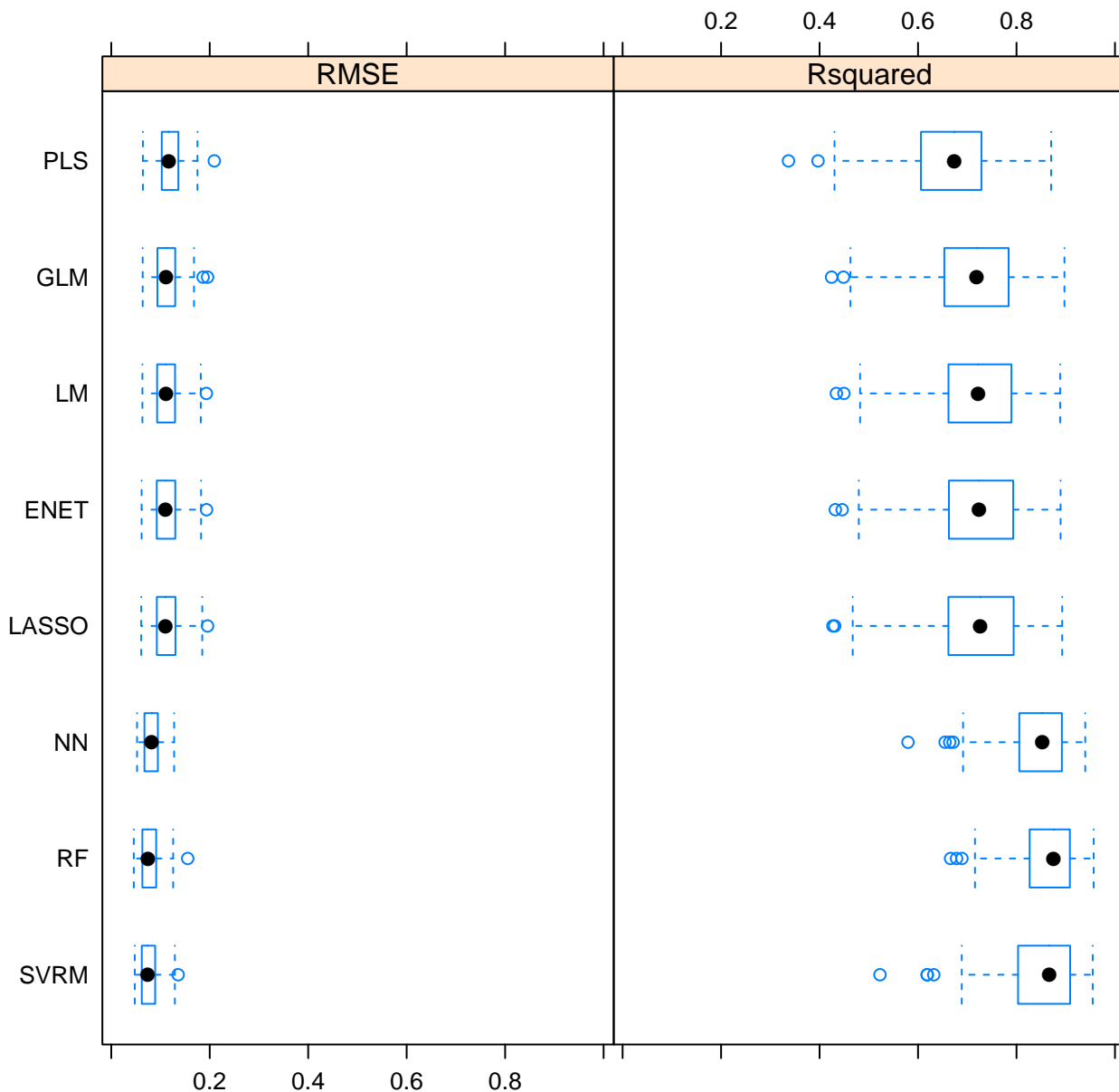

Supplement: Data S1 — Datailed results from UC Irvine Machine Learning Repository (Housing, Machine CPU, Wine Quality, Automobile and Parkinson) and the 3 Use Cases (Protein Corona, Gajewicz Metal Oxides and Aquatic Toxicity) [file peerj-04-2721-s001.zip › 1_housing/ModelsComp.iSplits.2.pdf]

# Resampling results on the training set (data split 3)

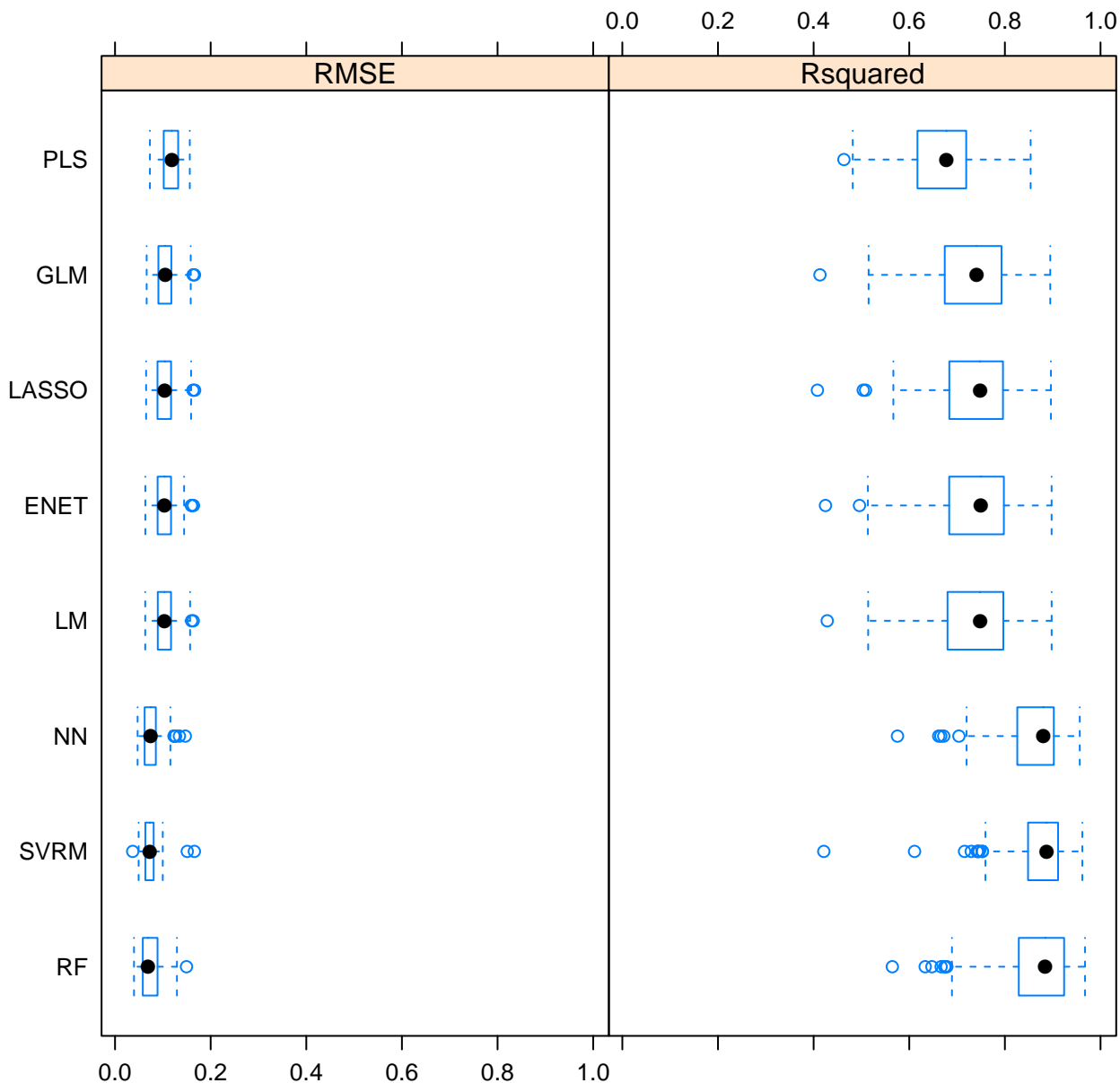

Supplement: Data S1 — Datailed results from UC Irvine Machine Learning Repository (Housing, Machine CPU, Wine Quality, Automobile and Parkinson) and the 3 Use Cases (Protein Corona, Gajewicz Metal Oxides and Aquatic Toxicity) [file peerj-04-2721-s001.zip › 1_housing/ModelsComp.iSplits.3.pdf]

# Resampling results on the training set (data split 4)

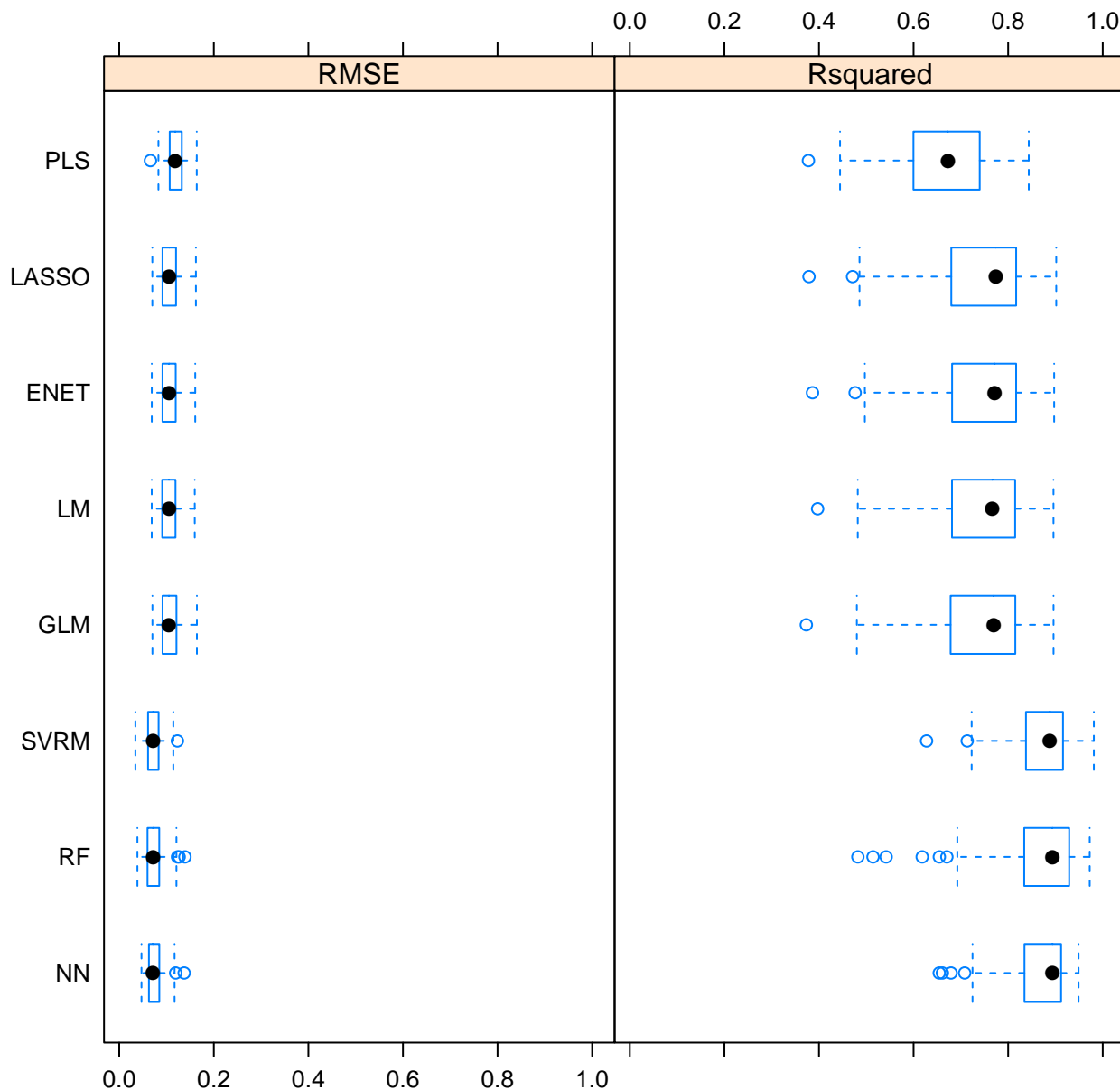

Supplement: Data S1 — Datailed results from UC Irvine Machine Learning Repository (Housing, Machine CPU, Wine Quality, Automobile and Parkinson) and the 3 Use Cases (Protein Corona, Gajewicz Metal Oxides and Aquatic Toxicity) [file peerj-04-2721-s001.zip › 1_housing/ModelsComp.iSplits.4.pdf]

# Resampling results on the training set (data split 5)

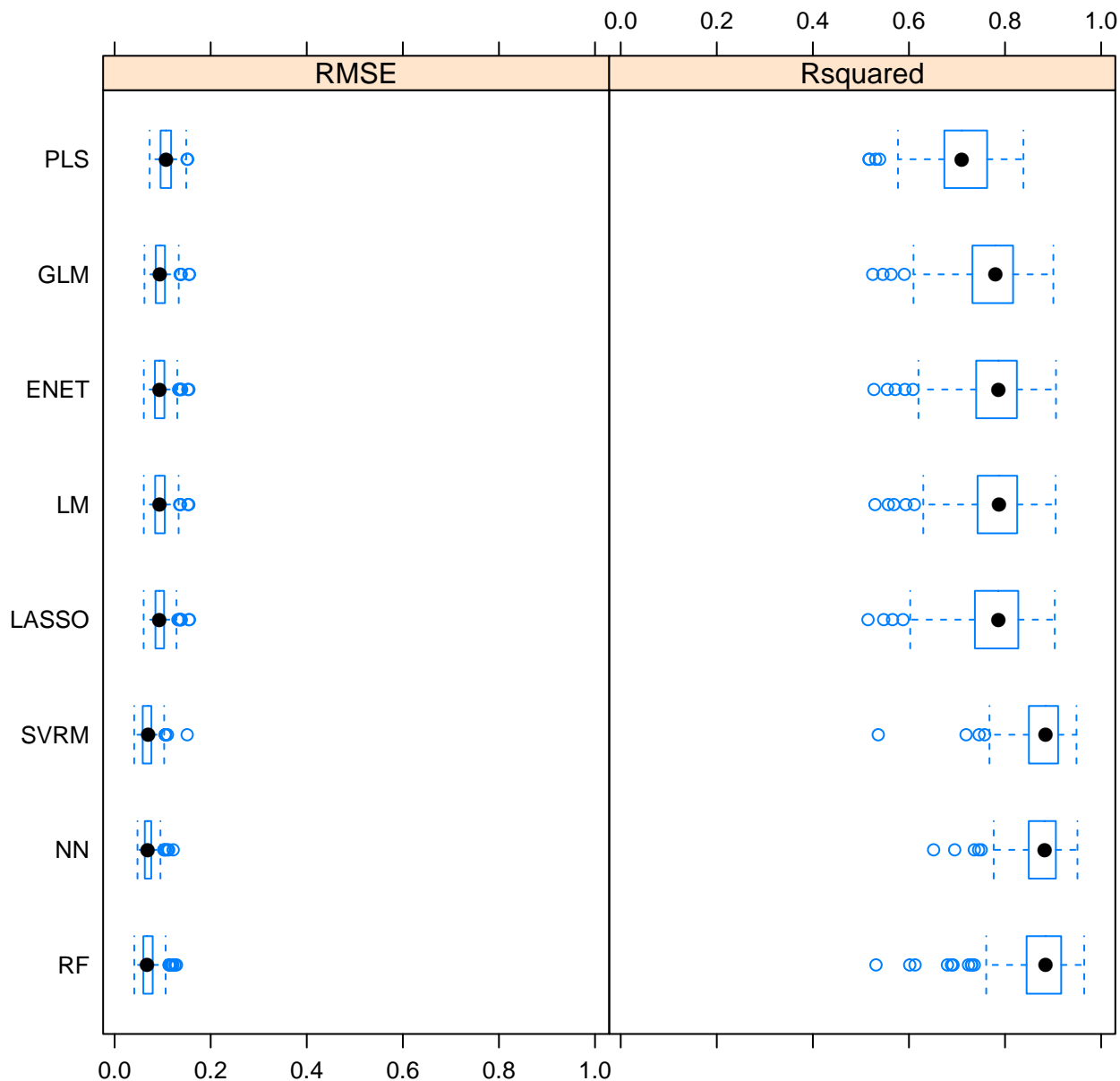

Supplement: Data S1 — Datailed results from UC Irvine Machine Learning Repository (Housing, Machine CPU, Wine Quality, Automobile and Parkinson) and the 3 Use Cases (Protein Corona, Gajewicz Metal Oxides and Aquatic Toxicity) [file peerj-04-2721-s001.zip › 1_housing/ModelsComp.iSplits.5.pdf]

# Resampling results on the training set (data split 6)

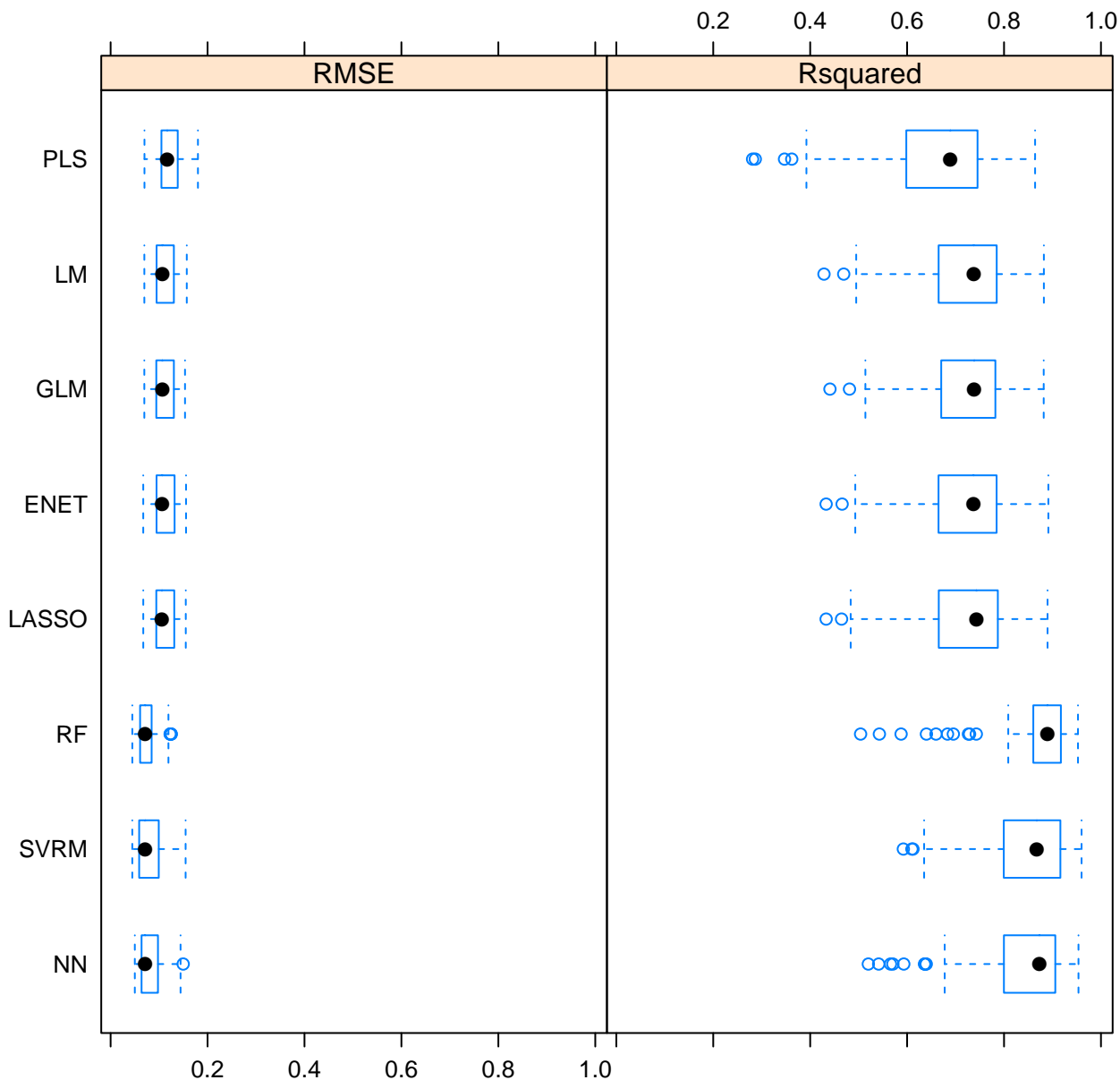

Supplement: Data S1 — Datailed results from UC Irvine Machine Learning Repository (Housing, Machine CPU, Wine Quality, Automobile and Parkinson) and the 3 Use Cases (Protein Corona, Gajewicz Metal Oxides and Aquatic Toxicity) [file peerj-04-2721-s001.zip › 1_housing/ModelsComp.iSplits.6.pdf]

# Resampling results on the training set (data split 7)

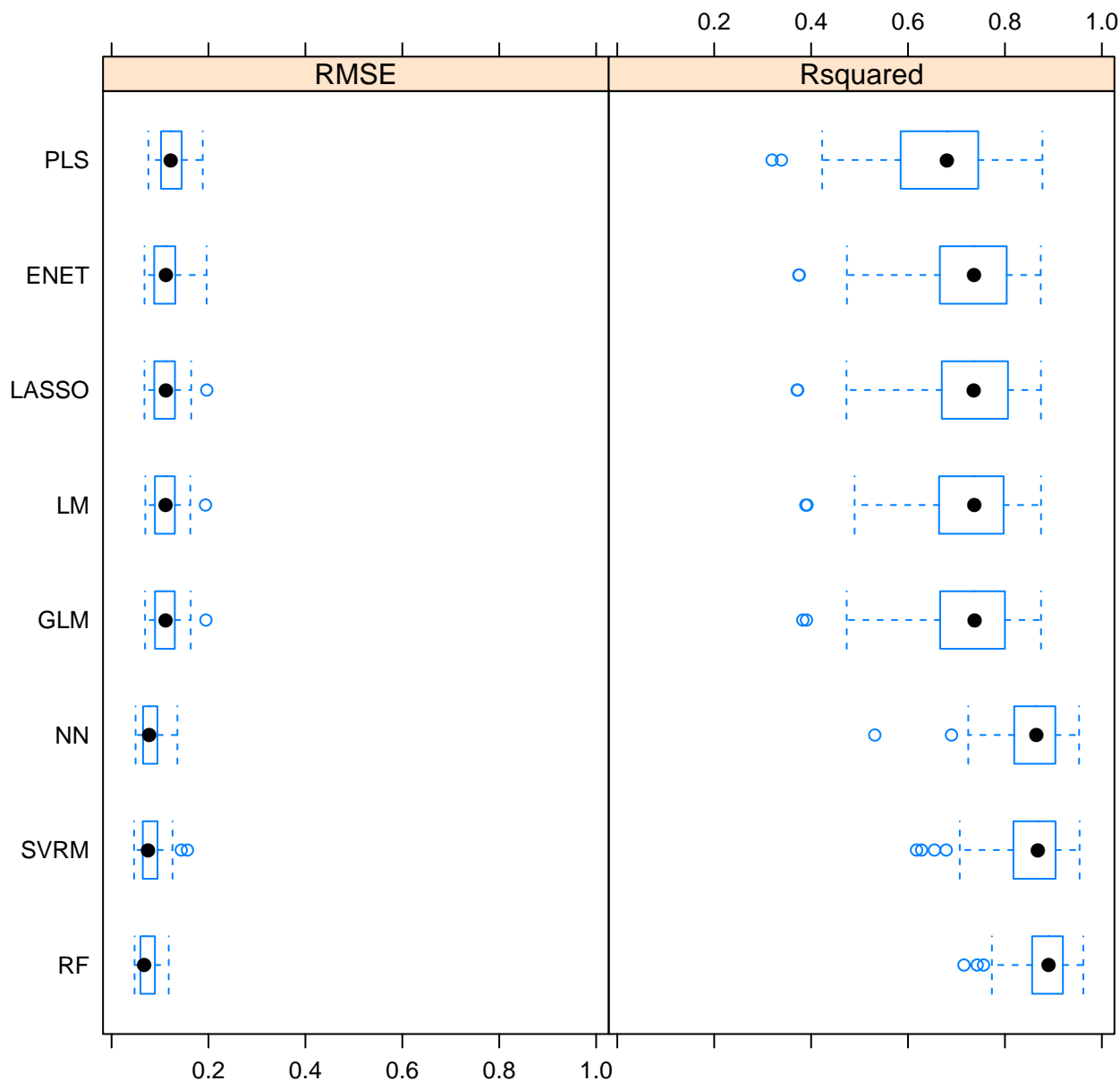

Supplement: Data S1 — Datailed results from UC Irvine Machine Learning Repository (Housing, Machine CPU, Wine Quality, Automobile and Parkinson) and the 3 Use Cases (Protein Corona, Gajewicz Metal Oxides and Aquatic Toxicity) [file peerj-04-2721-s001.zip › 1_housing/ModelsComp.iSplits.7.pdf]

# Resampling results on the training set (data split 8)

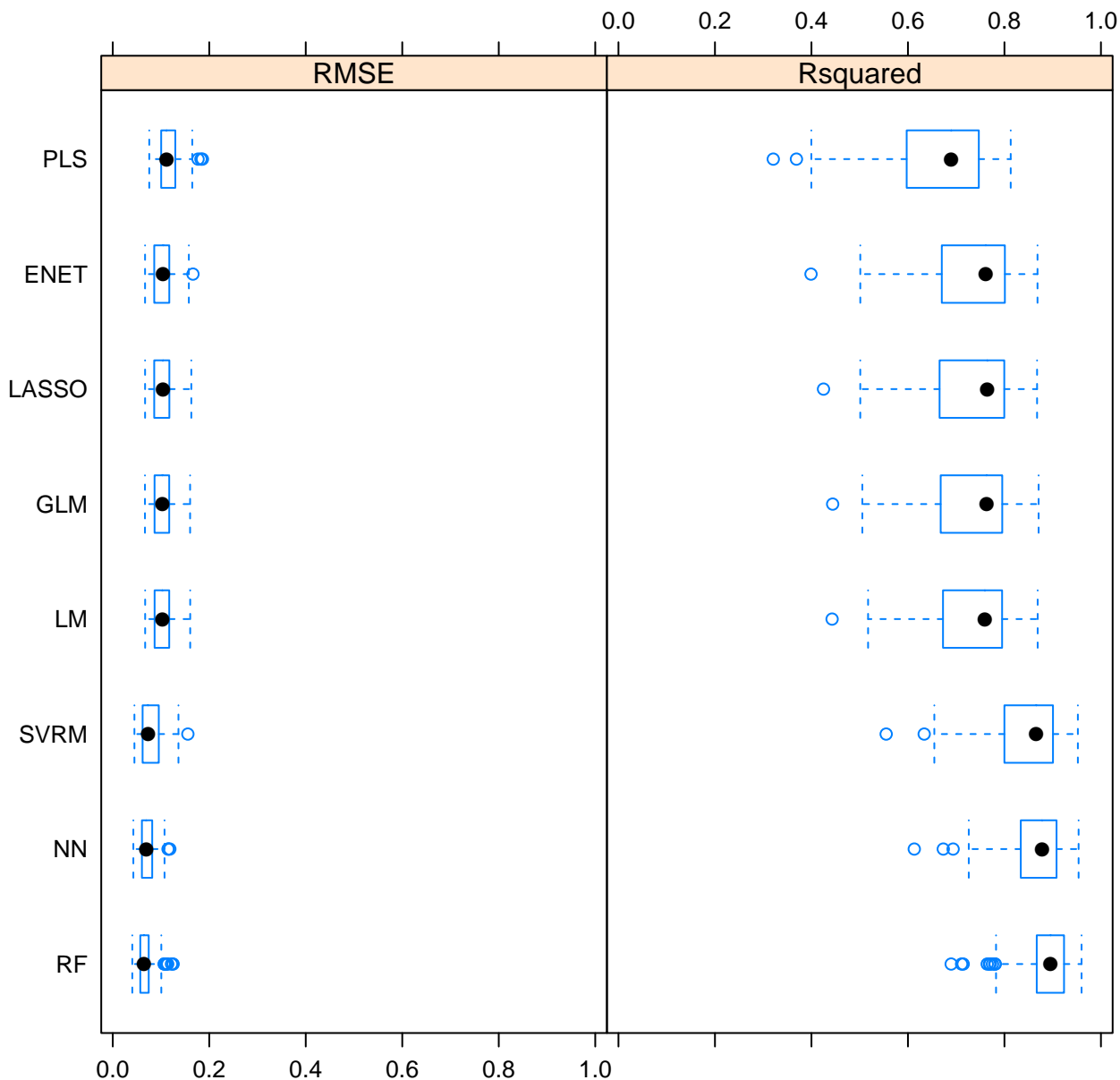

Supplement: Data S1 — Datailed results from UC Irvine Machine Learning Repository (Housing, Machine CPU, Wine Quality, Automobile and Parkinson) and the 3 Use Cases (Protein Corona, Gajewicz Metal Oxides and Aquatic Toxicity) [file peerj-04-2721-s001.zip › 1_housing/ModelsComp.iSplits.8.pdf]

# Resampling results on the training set (data split 9)

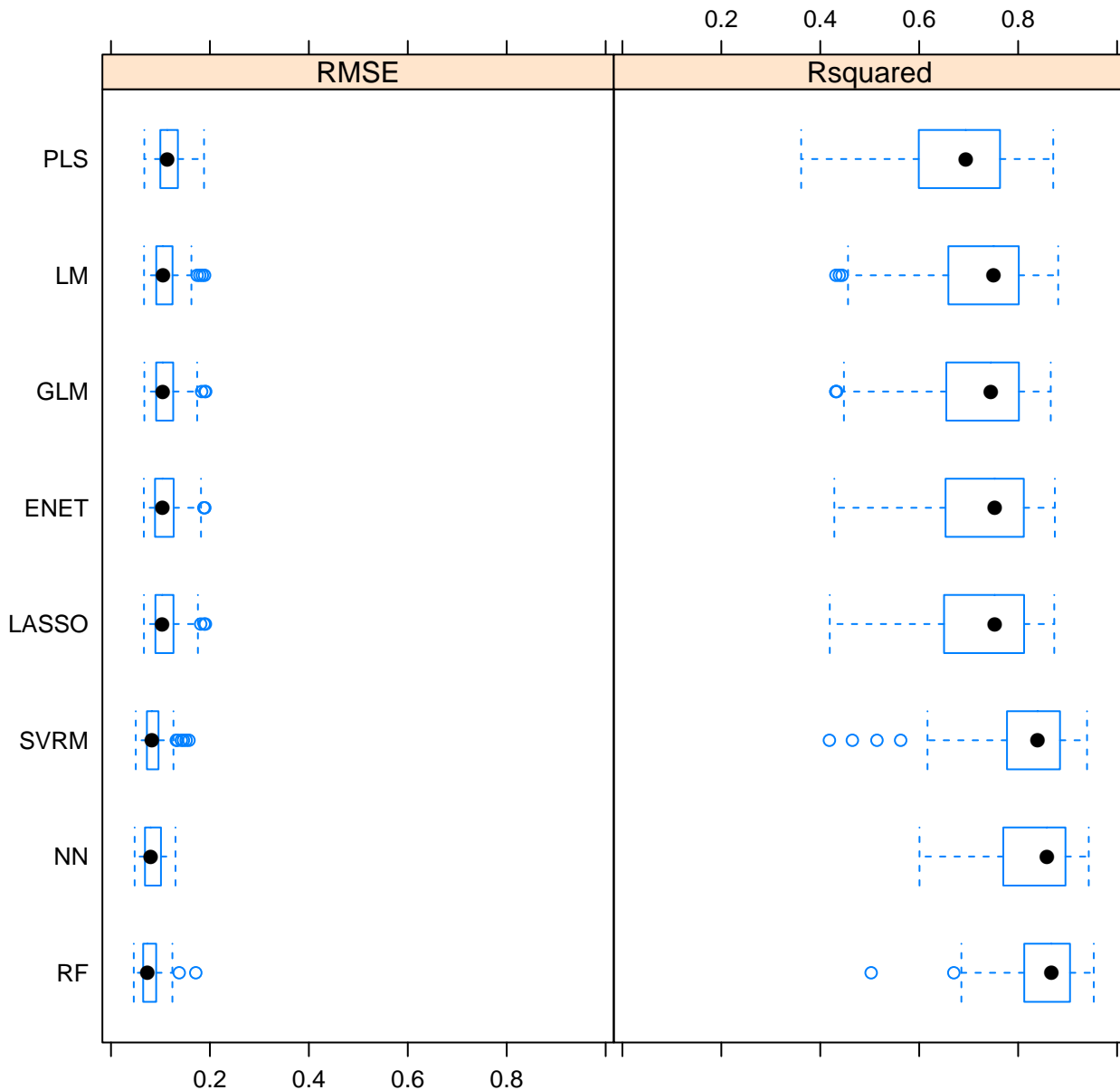

Supplement: Data S1 — Datailed results from UC Irvine Machine Learning Repository (Housing, Machine CPU, Wine Quality, Automobile and Parkinson) and the 3 Use Cases (Protein Corona, Gajewicz Metal Oxides and Aquatic Toxicity) [file peerj-04-2721-s001.zip › 1_housing/ModelsComp.iSplits.9.pdf]

# Models' differences on the training set (data split 1)

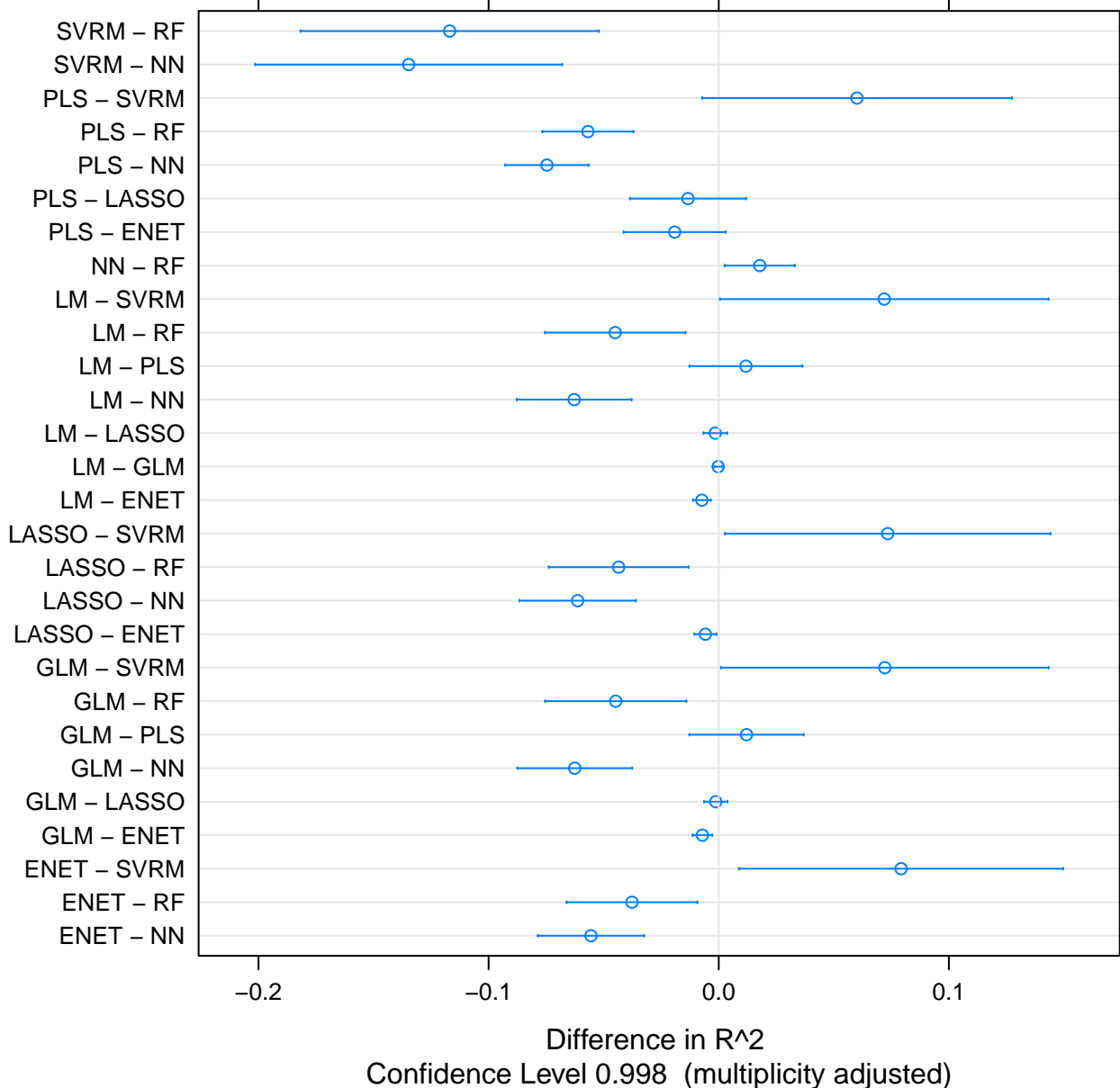

Supplement: Data S1 — Datailed results from UC Irvine Machine Learning Repository (Housing, Machine CPU, Wine Quality, Automobile and Parkinson) and the 3 Use Cases (Protein Corona, Gajewicz Metal Oxides and Aquatic Toxicity) [file peerj-04-2721-s001.zip › 2_machineCPU/DifModels.R2.iSplits.1.pdf]

# Models' differences on the training set (data split 10)

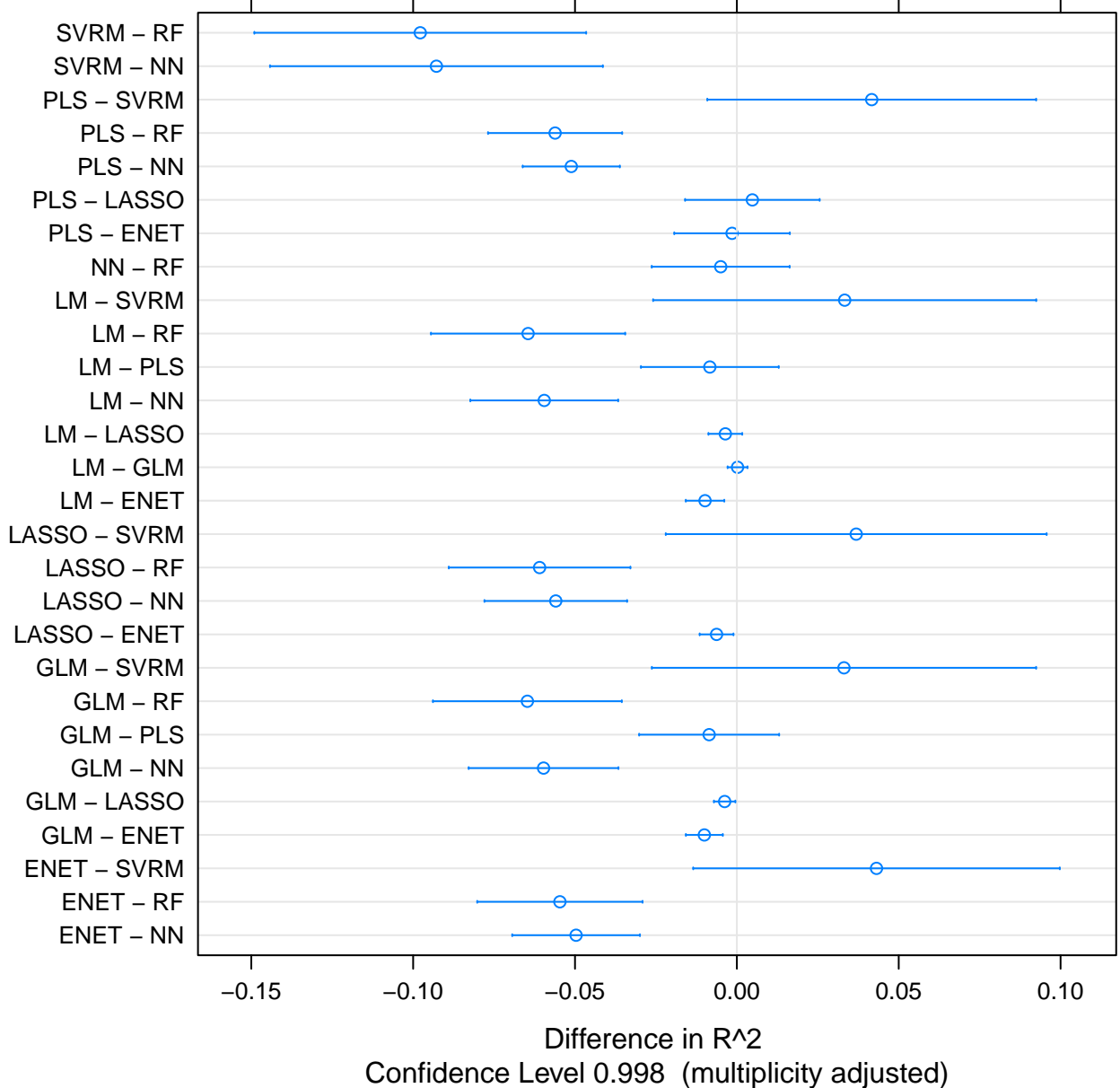

Supplement: Data S1 — Datailed results from UC Irvine Machine Learning Repository (Housing, Machine CPU, Wine Quality, Automobile and Parkinson) and the 3 Use Cases (Protein Corona, Gajewicz Metal Oxides and Aquatic Toxicity) [file peerj-04-2721-s001.zip › 2_machineCPU/DifModels.R2.iSplits.10.pdf]

# Models' differences on the training set (data split 2)

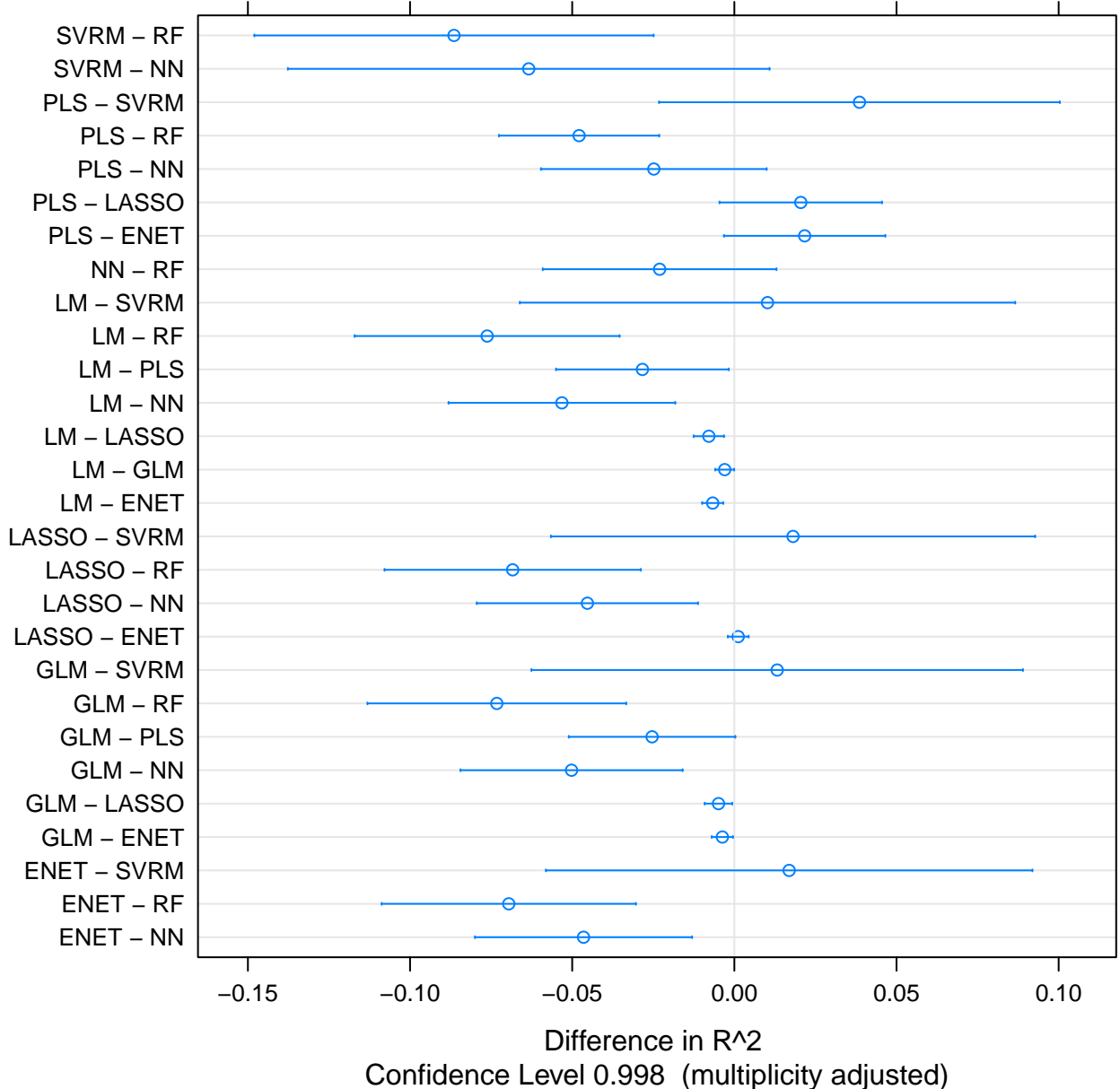

Supplement: Data S1 — Datailed results from UC Irvine Machine Learning Repository (Housing, Machine CPU, Wine Quality, Automobile and Parkinson) and the 3 Use Cases (Protein Corona, Gajewicz Metal Oxides and Aquatic Toxicity) [file peerj-04-2721-s001.zip › 2_machineCPU/DifModels.R2.iSplits.2.pdf]

# Models' differences on the training set (data split 3)

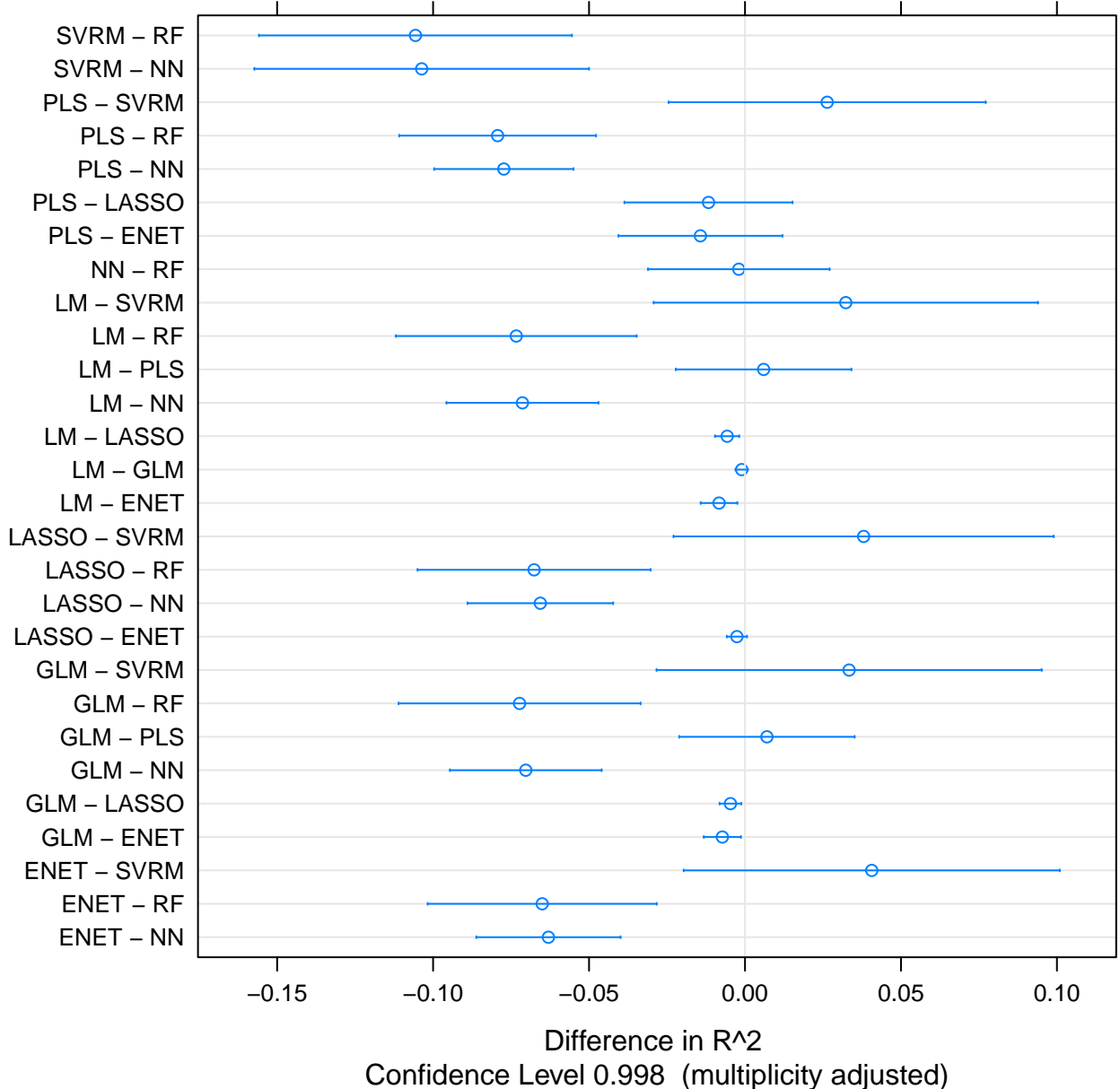

Supplement: Data S1 — Datailed results from UC Irvine Machine Learning Repository (Housing, Machine CPU, Wine Quality, Automobile and Parkinson) and the 3 Use Cases (Protein Corona, Gajewicz Metal Oxides and Aquatic Toxicity) [file peerj-04-2721-s001.zip › 2_machineCPU/DifModels.R2.iSplits.3.pdf]

# Models' differences on the training set (data split 4)

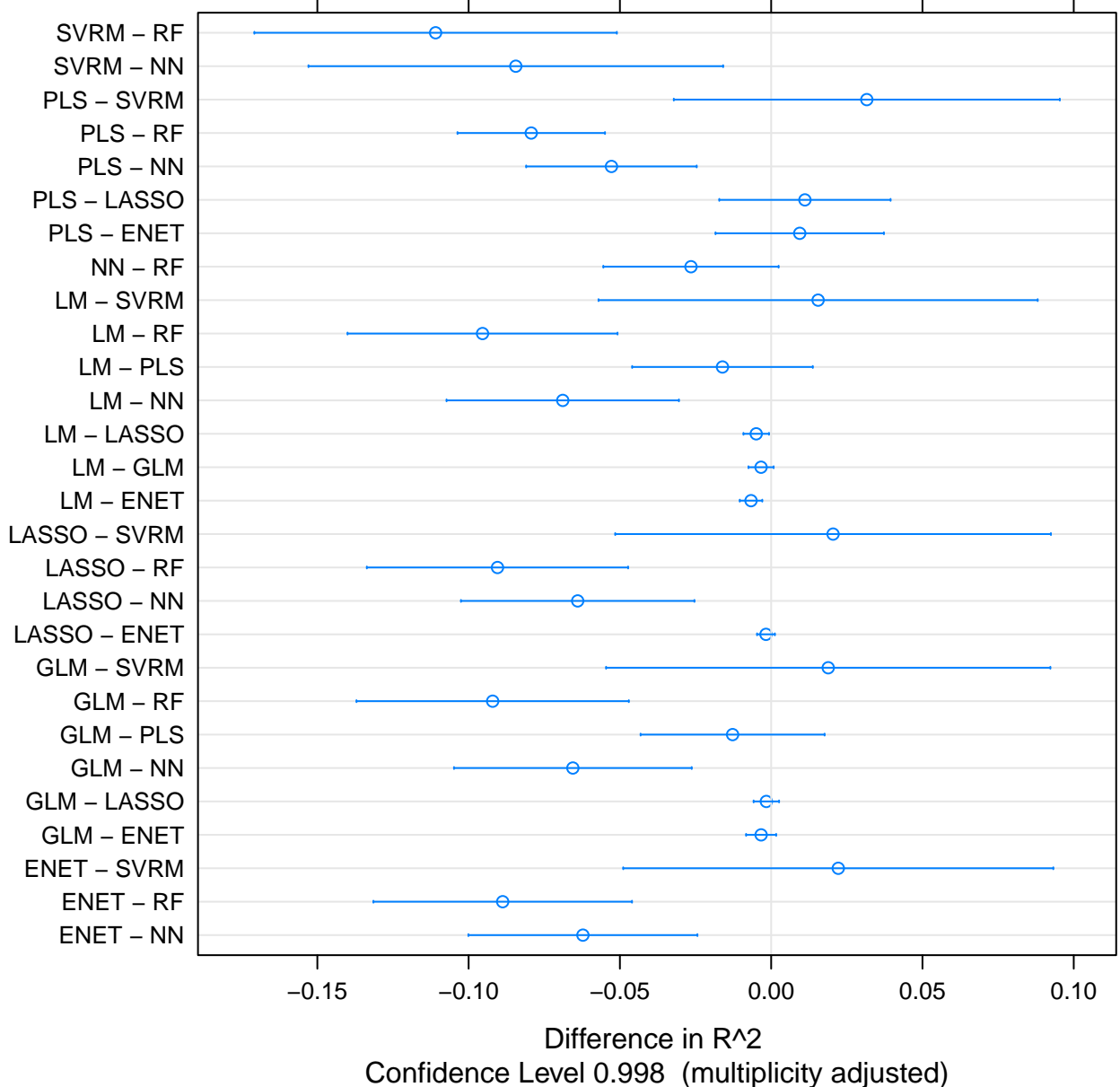

Supplement: Data S1 — Datailed results from UC Irvine Machine Learning Repository (Housing, Machine CPU, Wine Quality, Automobile and Parkinson) and the 3 Use Cases (Protein Corona, Gajewicz Metal Oxides and Aquatic Toxicity) [file peerj-04-2721-s001.zip › 2_machineCPU/DifModels.R2.iSplits.4.pdf]

# Models' differences on the training set (data split 5)

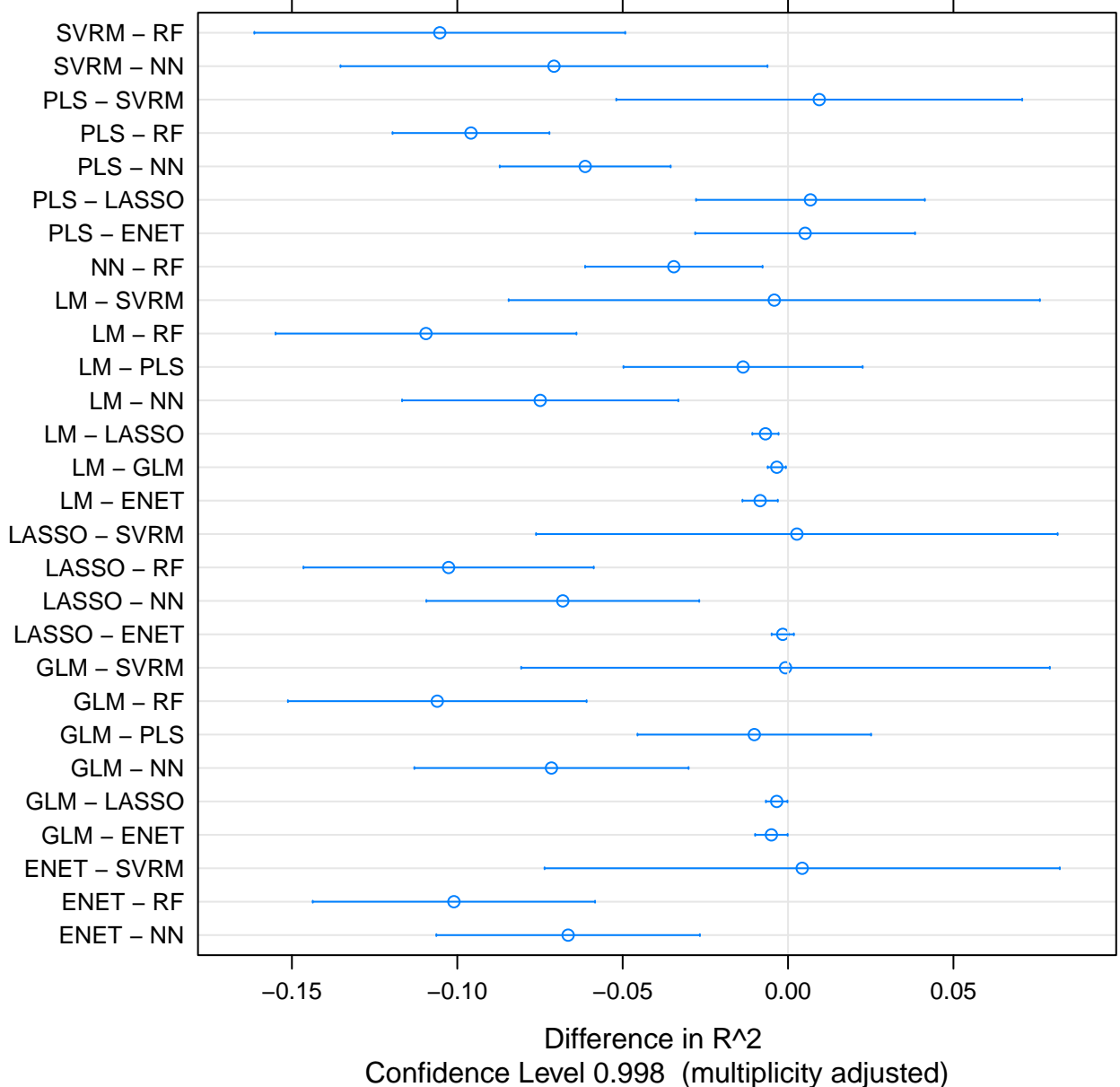

Supplement: Data S1 — Datailed results from UC Irvine Machine Learning Repository (Housing, Machine CPU, Wine Quality, Automobile and Parkinson) and the 3 Use Cases (Protein Corona, Gajewicz Metal Oxides and Aquatic Toxicity) [file peerj-04-2721-s001.zip › 2_machineCPU/DifModels.R2.iSplits.5.pdf]

# Models' differences on the training set (data split 6)

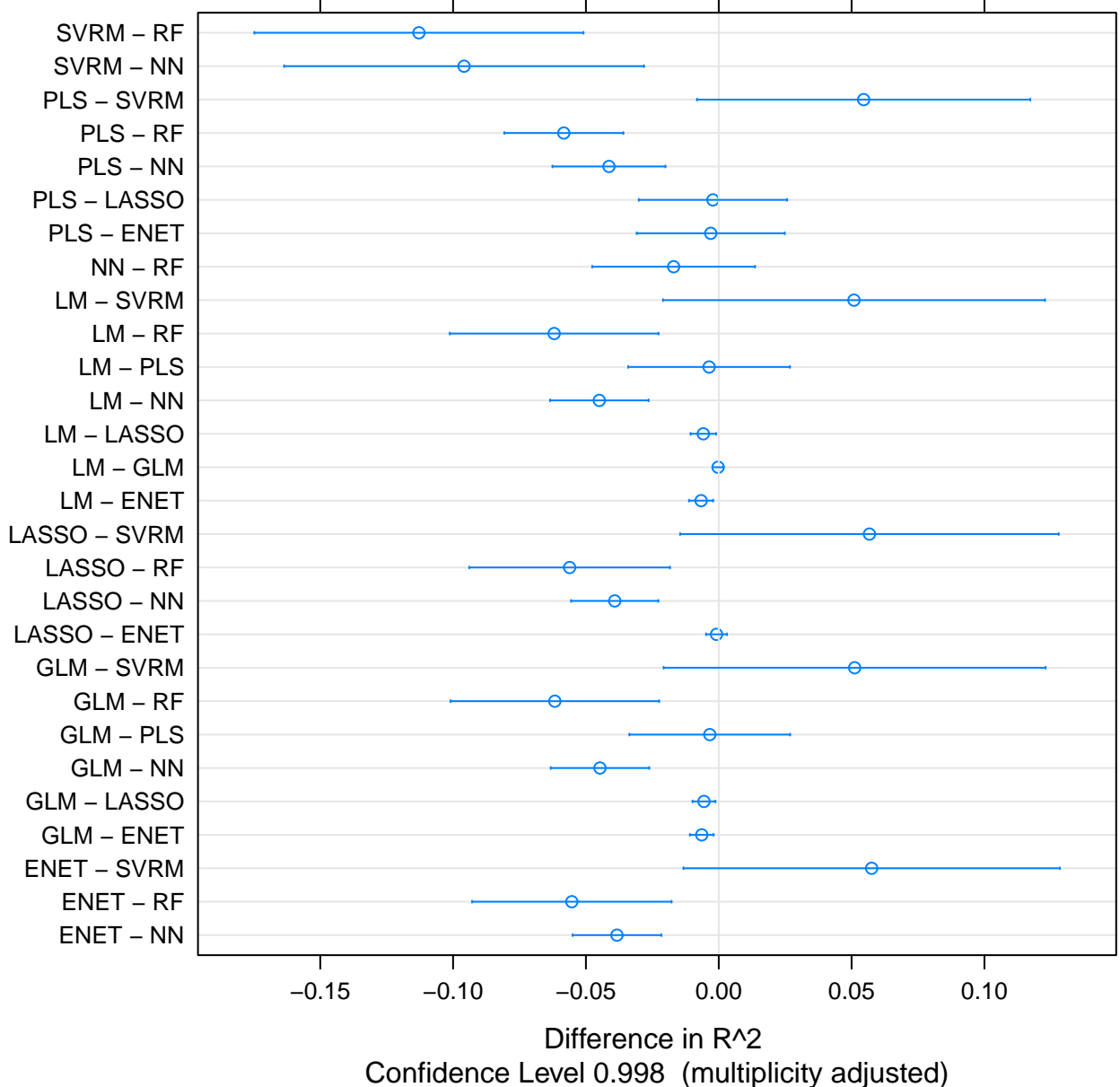

Supplement: Data S1 — Datailed results from UC Irvine Machine Learning Repository (Housing, Machine CPU, Wine Quality, Automobile and Parkinson) and the 3 Use Cases (Protein Corona, Gajewicz Metal Oxides and Aquatic Toxicity) [file peerj-04-2721-s001.zip › 2_machineCPU/DifModels.R2.iSplits.6.pdf]

# Models' differences on the training set (data split 7)

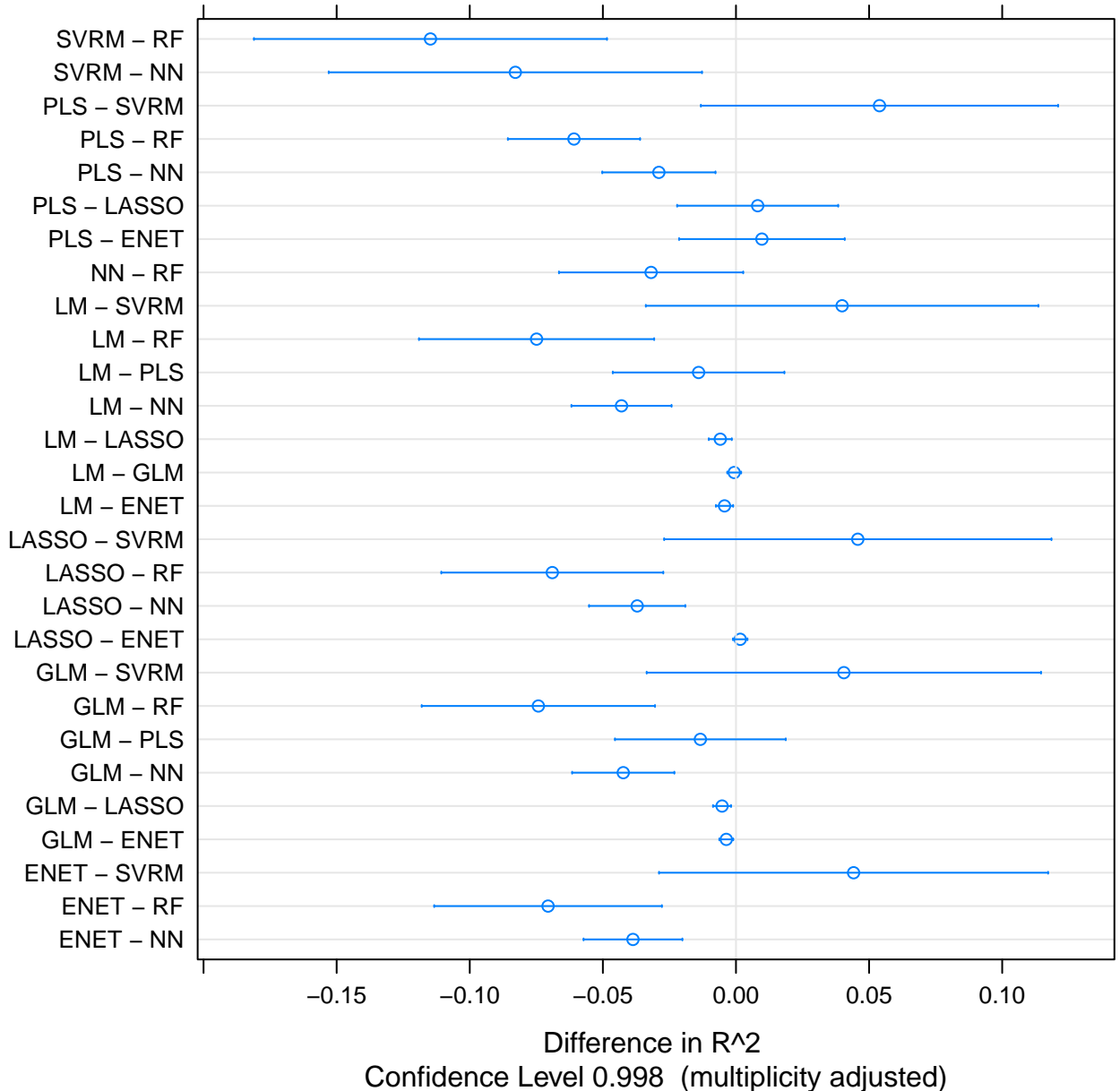

Supplement: Data S1 — Datailed results from UC Irvine Machine Learning Repository (Housing, Machine CPU, Wine Quality, Automobile and Parkinson) and the 3 Use Cases (Protein Corona, Gajewicz Metal Oxides and Aquatic Toxicity) [file peerj-04-2721-s001.zip › 2_machineCPU/DifModels.R2.iSplits.7.pdf]

# Models' differences on the training set (data split 8)

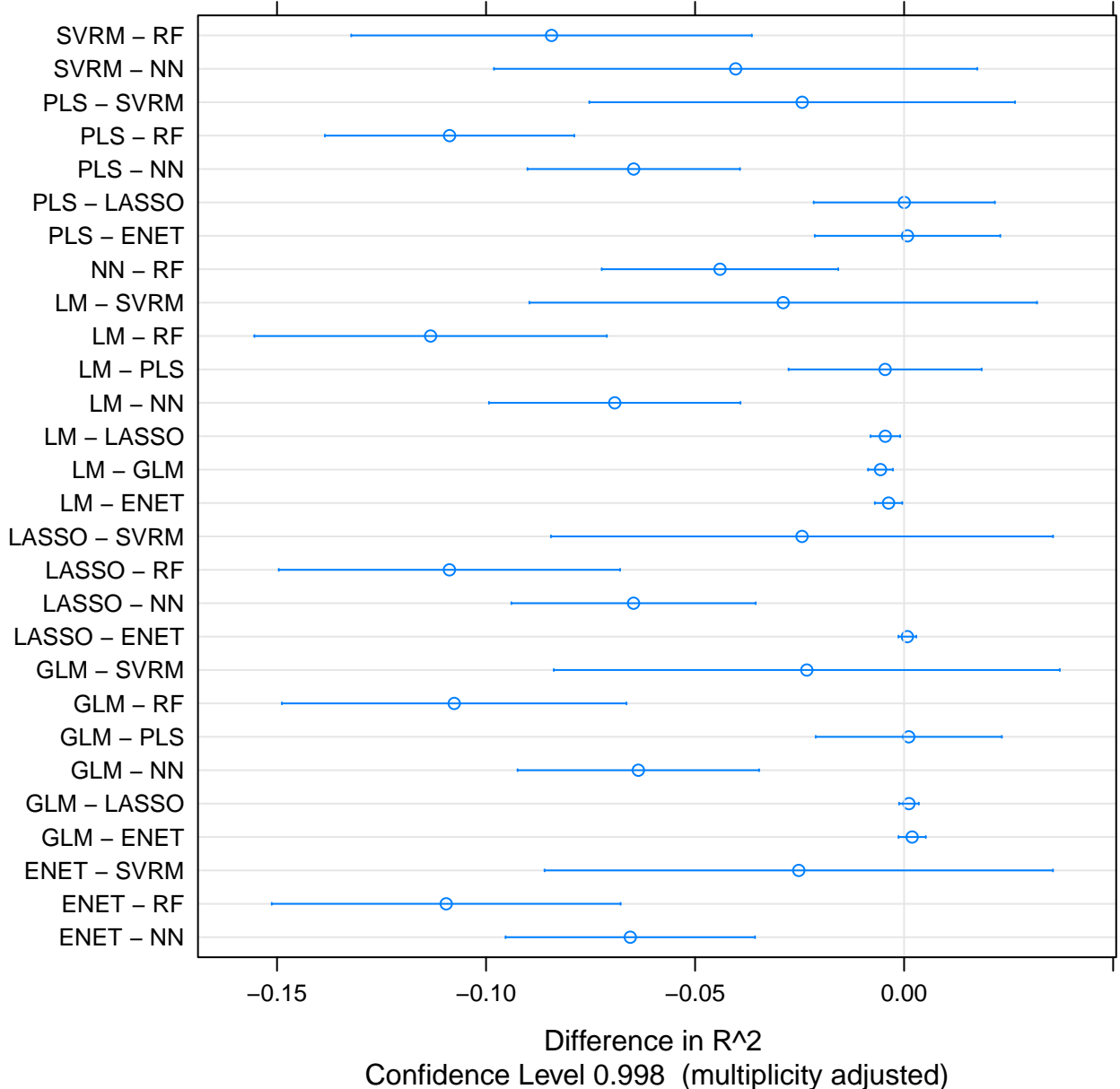

Supplement: Data S1 — Datailed results from UC Irvine Machine Learning Repository (Housing, Machine CPU, Wine Quality, Automobile and Parkinson) and the 3 Use Cases (Protein Corona, Gajewicz Metal Oxides and Aquatic Toxicity) [file peerj-04-2721-s001.zip › 2_machineCPU/DifModels.R2.iSplits.8.pdf]

# Models' differences on the training set (data split 9)

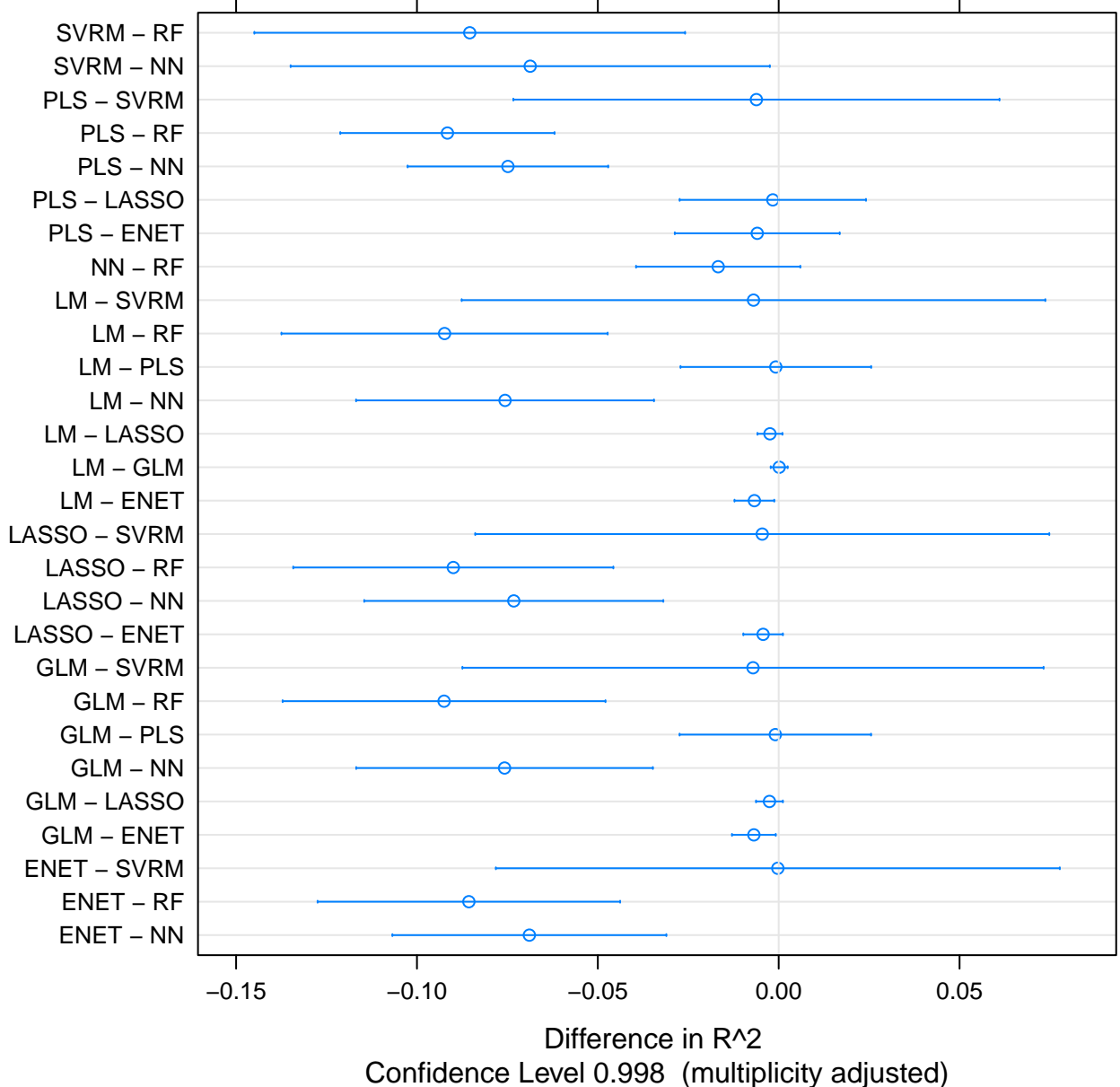

Supplement: Data S1 — Datailed results from UC Irvine Machine Learning Repository (Housing, Machine CPU, Wine Quality, Automobile and Parkinson) and the 3 Use Cases (Protein Corona, Gajewicz Metal Oxides and Aquatic Toxicity) [file peerj-04-2721-s001.zip › 2_machineCPU/DifModels.R2.iSplits.9.pdf]

# Models' differences on the training set (data split 1)

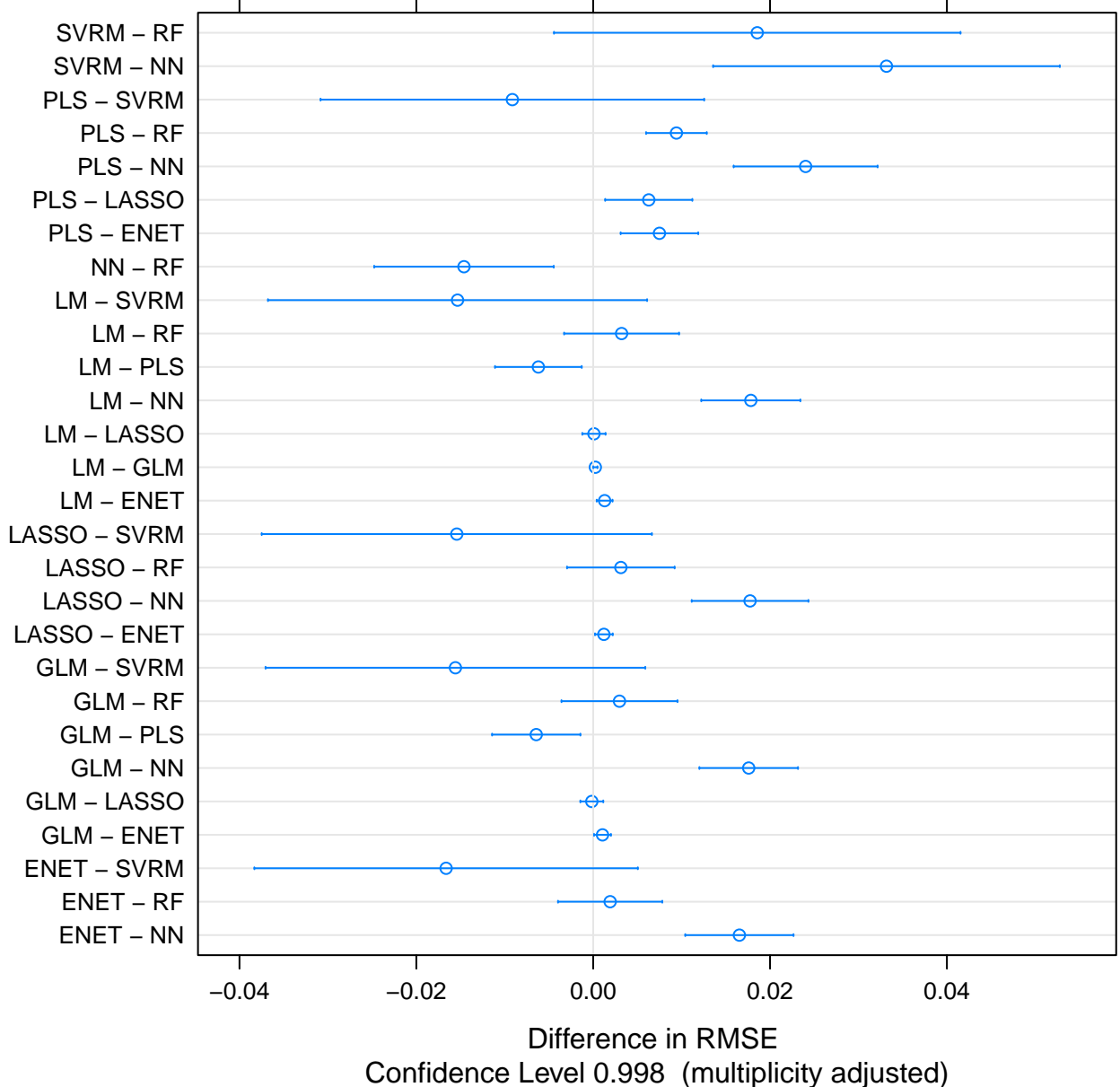

Supplement: Data S1 — Datailed results from UC Irvine Machine Learning Repository (Housing, Machine CPU, Wine Quality, Automobile and Parkinson) and the 3 Use Cases (Protein Corona, Gajewicz Metal Oxides and Aquatic Toxicity) [file peerj-04-2721-s001.zip › 2_machineCPU/DifModels.RMSE.iSplits.1.pdf]

# Models' differences on the training set (data split 10)

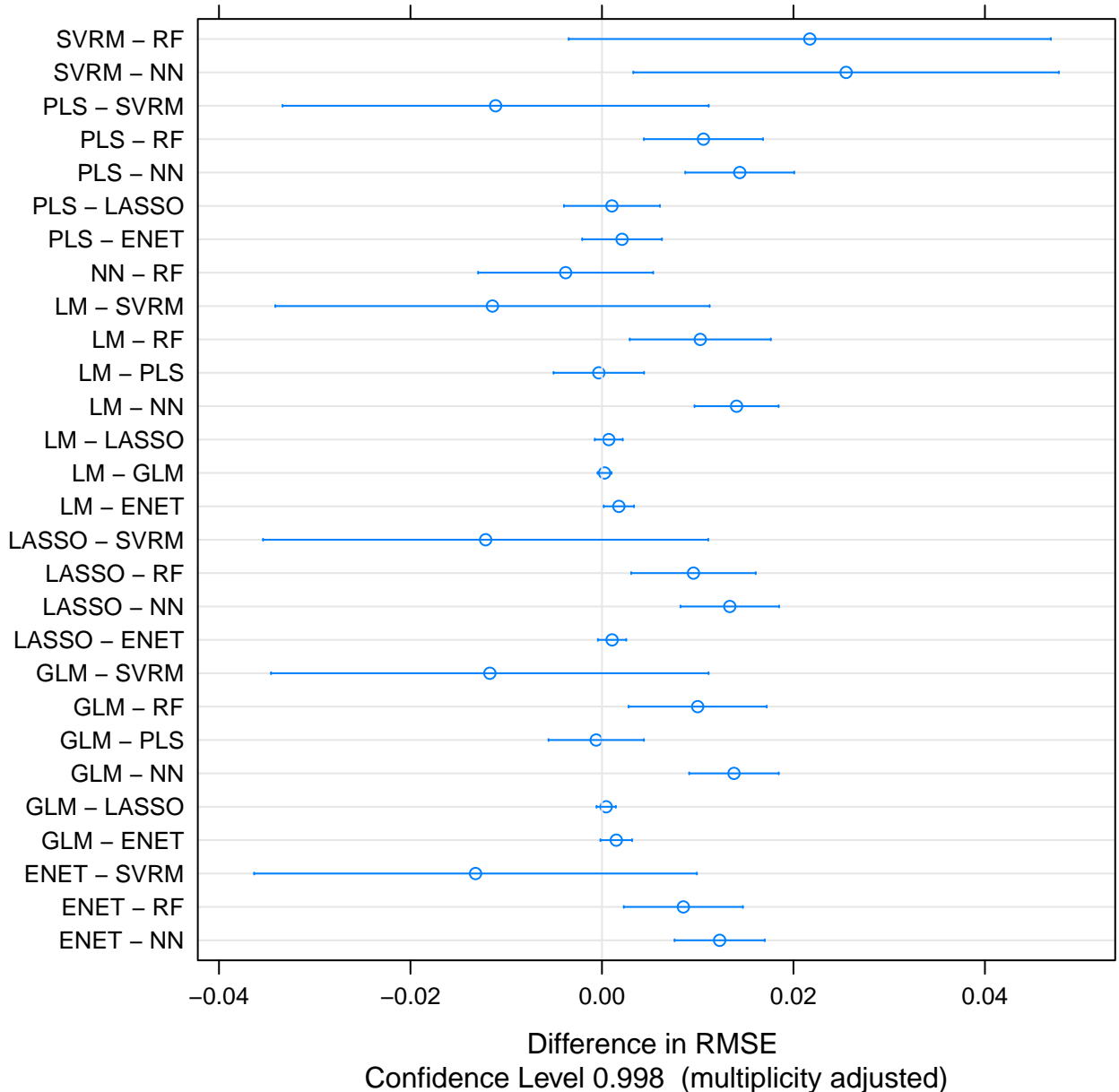

Supplement: Data S1 — Datailed results from UC Irvine Machine Learning Repository (Housing, Machine CPU, Wine Quality, Automobile and Parkinson) and the 3 Use Cases (Protein Corona, Gajewicz Metal Oxides and Aquatic Toxicity) [file peerj-04-2721-s001.zip › 2_machineCPU/DifModels.RMSE.iSplits.10.pdf]

# Models' differences on the training set (data split 2)

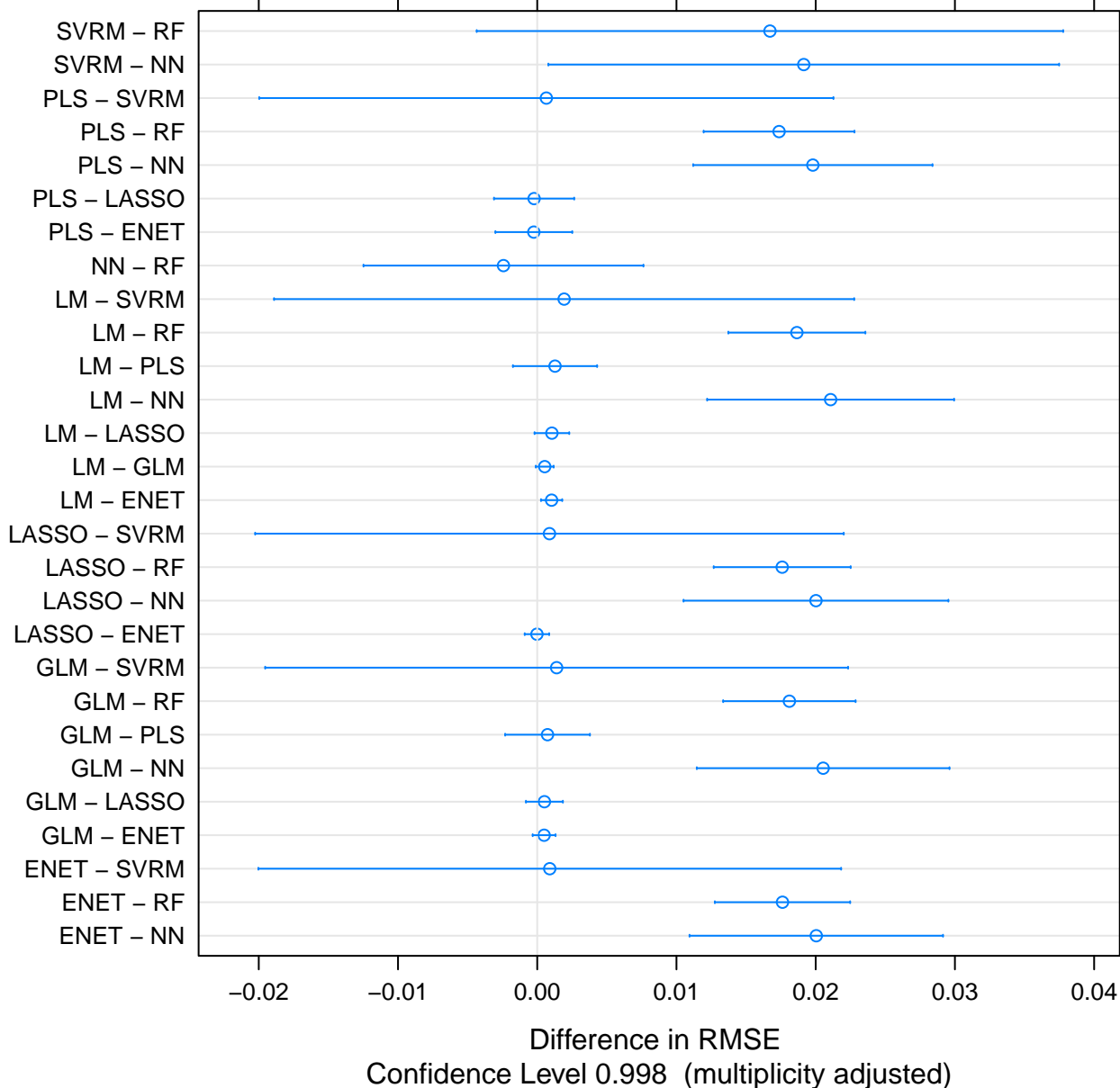

Supplement: Data S1 — Datailed results from UC Irvine Machine Learning Repository (Housing, Machine CPU, Wine Quality, Automobile and Parkinson) and the 3 Use Cases (Protein Corona, Gajewicz Metal Oxides and Aquatic Toxicity) [file peerj-04-2721-s001.zip › 2_machineCPU/DifModels.RMSE.iSplits.2.pdf]

# Models' differences on the training set (data split 3)

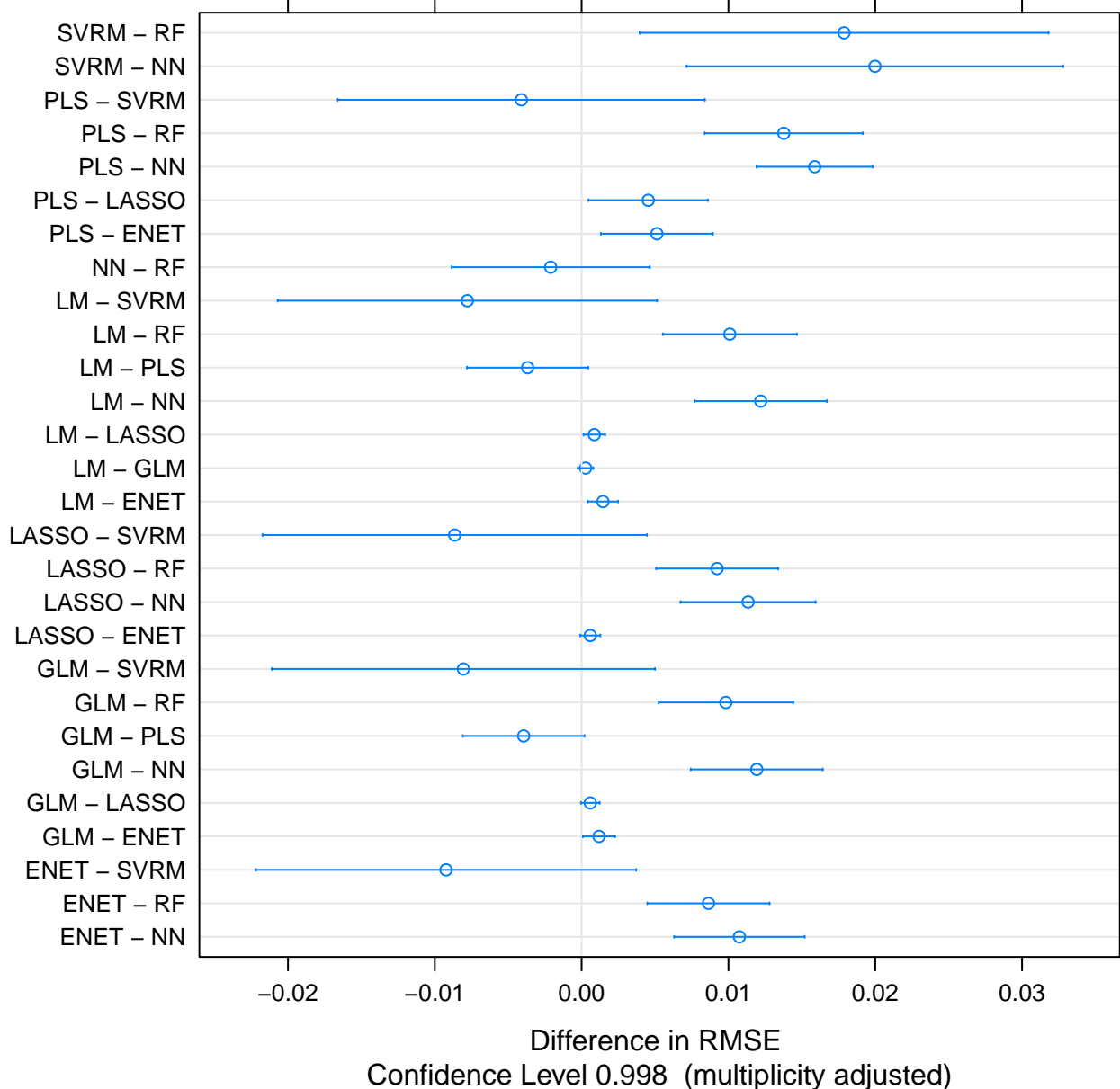

Supplement: Data S1 — Datailed results from UC Irvine Machine Learning Repository (Housing, Machine CPU, Wine Quality, Automobile and Parkinson) and the 3 Use Cases (Protein Corona, Gajewicz Metal Oxides and Aquatic Toxicity) [file peerj-04-2721-s001.zip › 2_machineCPU/DifModels.RMSE.iSplits.3.pdf]

# Models' differences on the training set (data split 4)

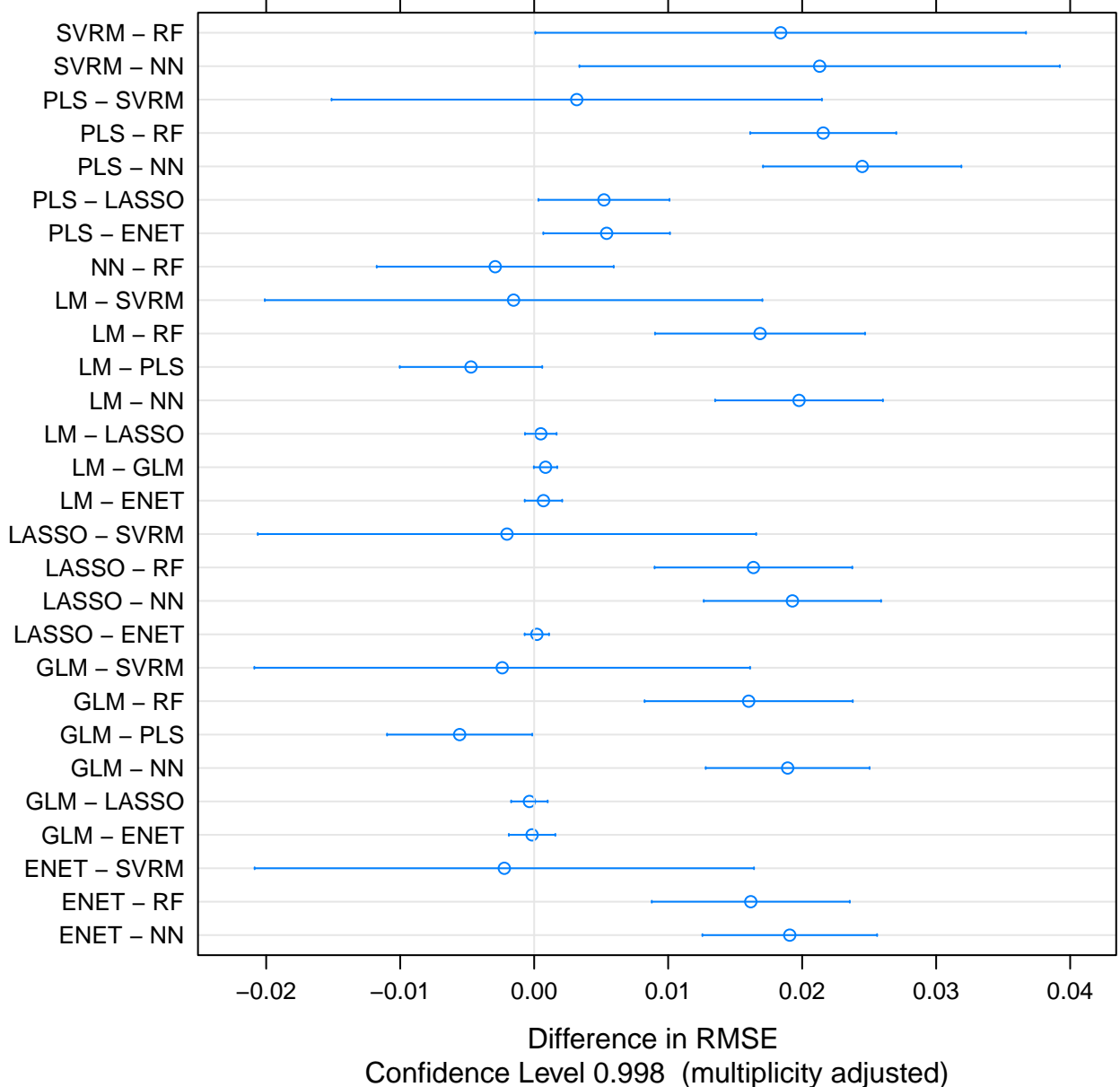

Supplement: Data S1 — Datailed results from UC Irvine Machine Learning Repository (Housing, Machine CPU, Wine Quality, Automobile and Parkinson) and the 3 Use Cases (Protein Corona, Gajewicz Metal Oxides and Aquatic Toxicity) [file peerj-04-2721-s001.zip › 2_machineCPU/DifModels.RMSE.iSplits.4.pdf]

# Models' differences on the training set (data split 5)

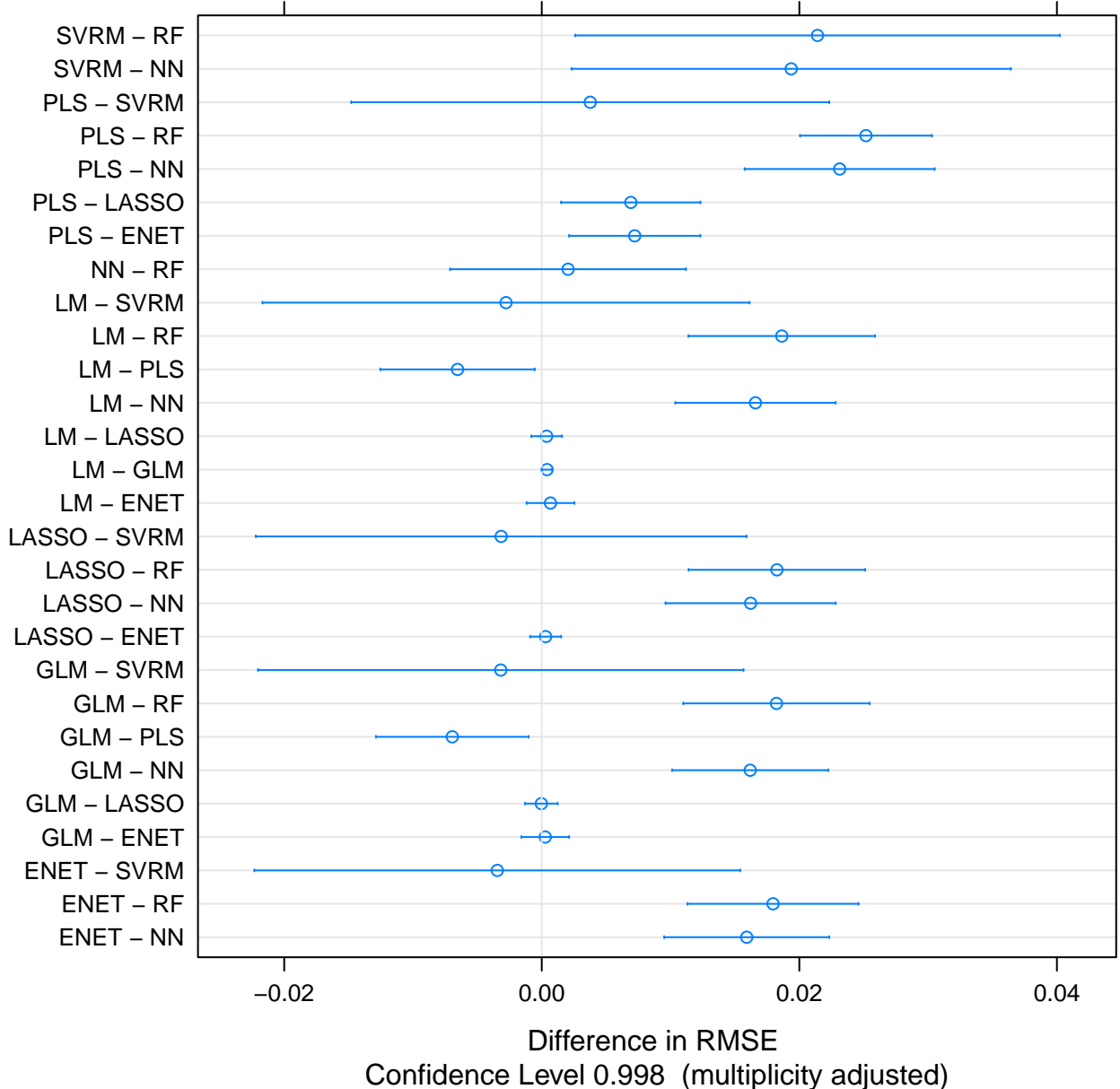

Supplement: Data S1 — Datailed results from UC Irvine Machine Learning Repository (Housing, Machine CPU, Wine Quality, Automobile and Parkinson) and the 3 Use Cases (Protein Corona, Gajewicz Metal Oxides and Aquatic Toxicity) [file peerj-04-2721-s001.zip › 2_machineCPU/DifModels.RMSE.iSplits.5.pdf]

# Models' differences on the training set (data split 6)

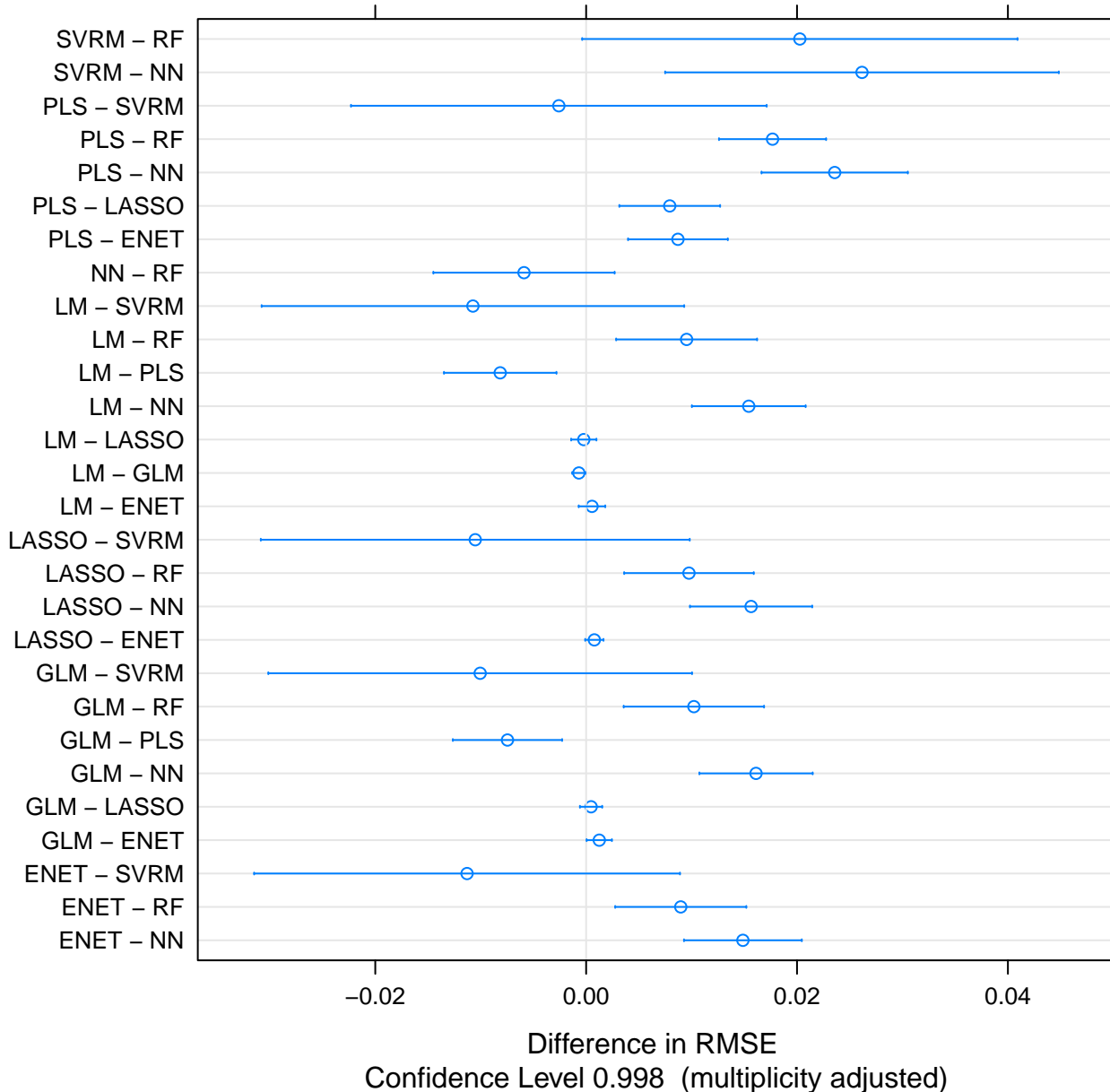

Supplement: Data S1 — Datailed results from UC Irvine Machine Learning Repository (Housing, Machine CPU, Wine Quality, Automobile and Parkinson) and the 3 Use Cases (Protein Corona, Gajewicz Metal Oxides and Aquatic Toxicity) [file peerj-04-2721-s001.zip › 2_machineCPU/DifModels.RMSE.iSplits.6.pdf]

# Models' differences on the training set (data split 7)

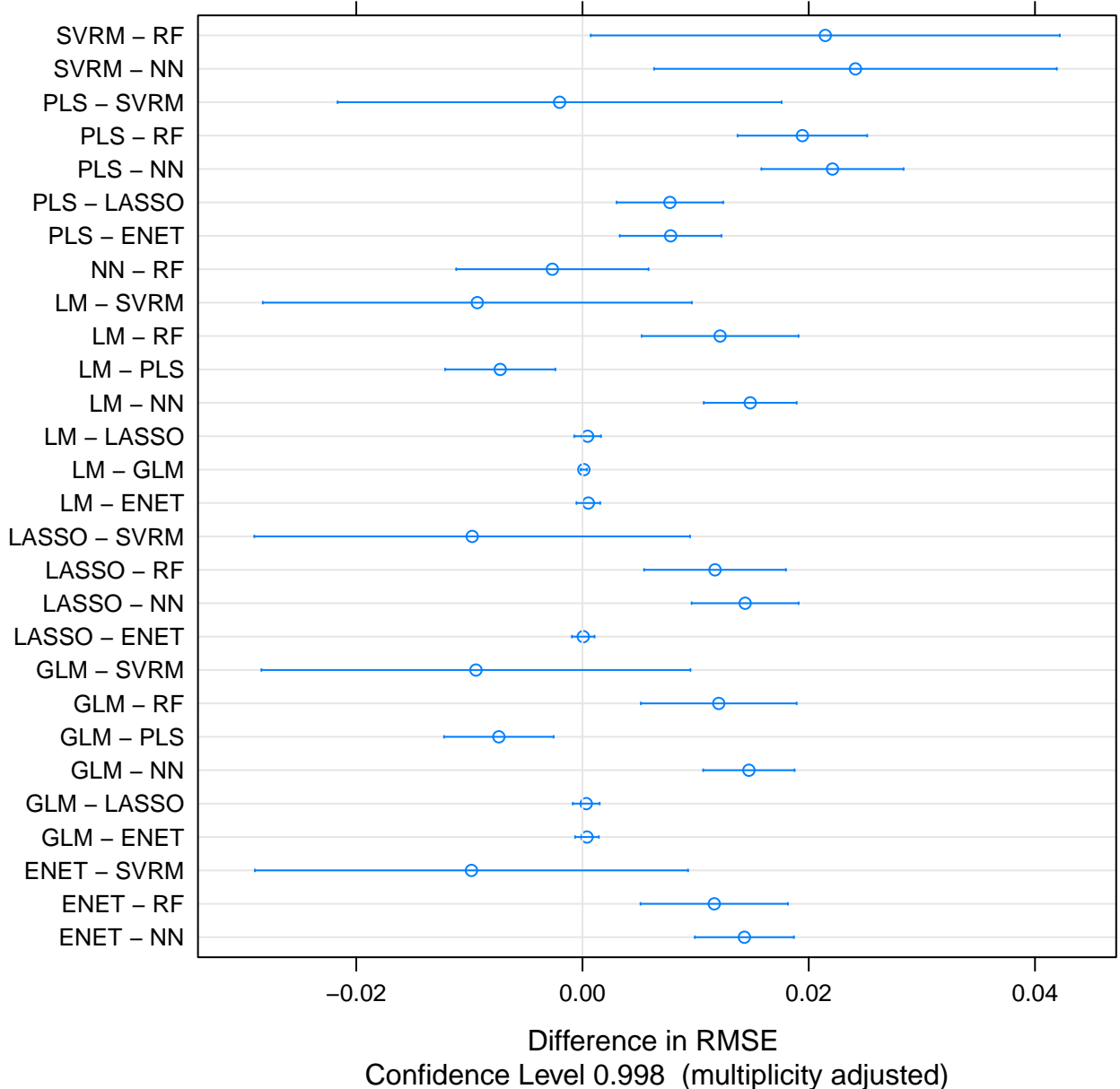

Supplement: Data S1 — Datailed results from UC Irvine Machine Learning Repository (Housing, Machine CPU, Wine Quality, Automobile and Parkinson) and the 3 Use Cases (Protein Corona, Gajewicz Metal Oxides and Aquatic Toxicity) [file peerj-04-2721-s001.zip › 2_machineCPU/DifModels.RMSE.iSplits.7.pdf]

# Models' differences on the training set (data split 8)

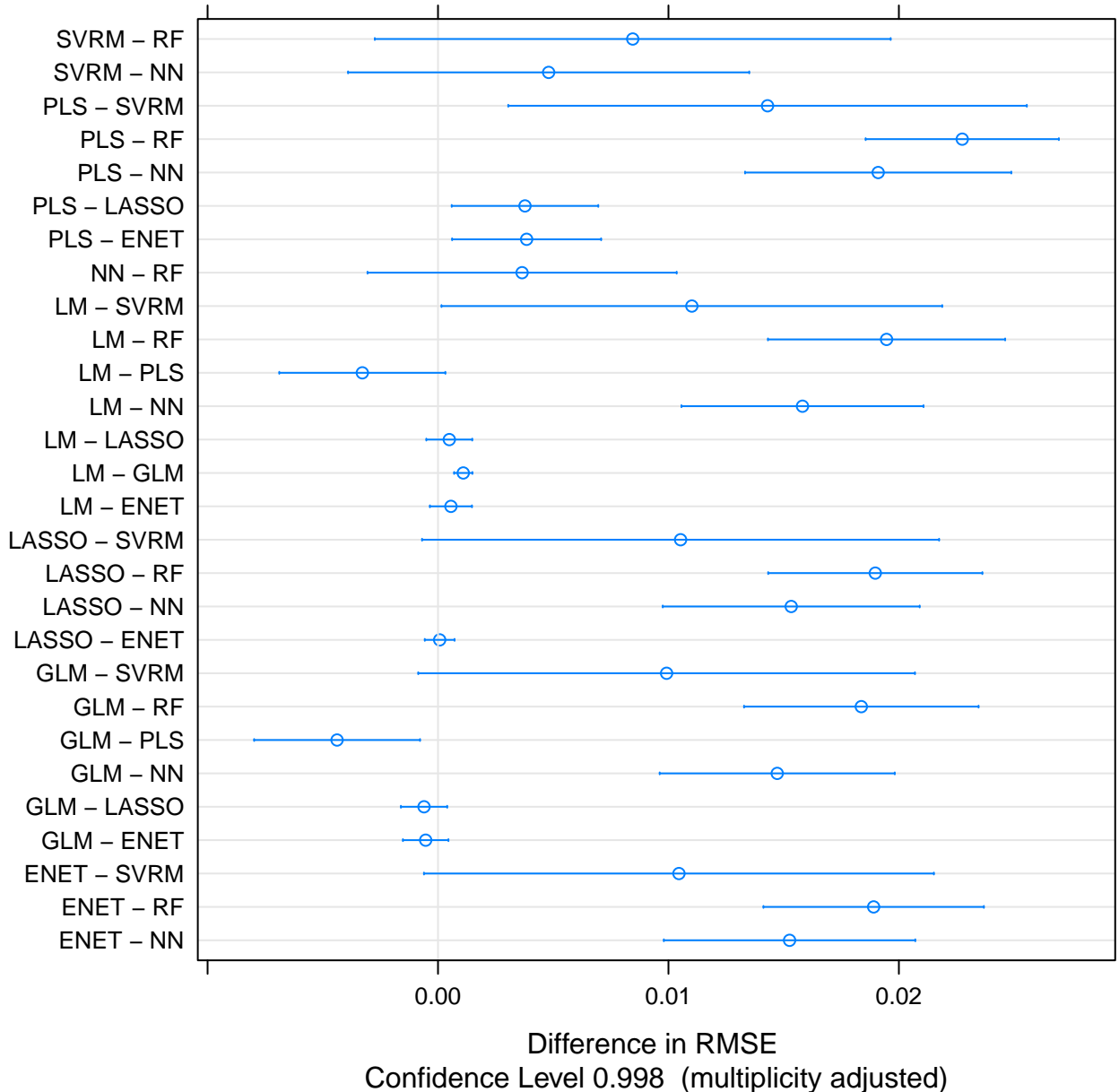

Supplement: Data S1 — Datailed results from UC Irvine Machine Learning Repository (Housing, Machine CPU, Wine Quality, Automobile and Parkinson) and the 3 Use Cases (Protein Corona, Gajewicz Metal Oxides and Aquatic Toxicity) [file peerj-04-2721-s001.zip › 2_machineCPU/DifModels.RMSE.iSplits.8.pdf]

# Models' differences on the training set (data split 9)

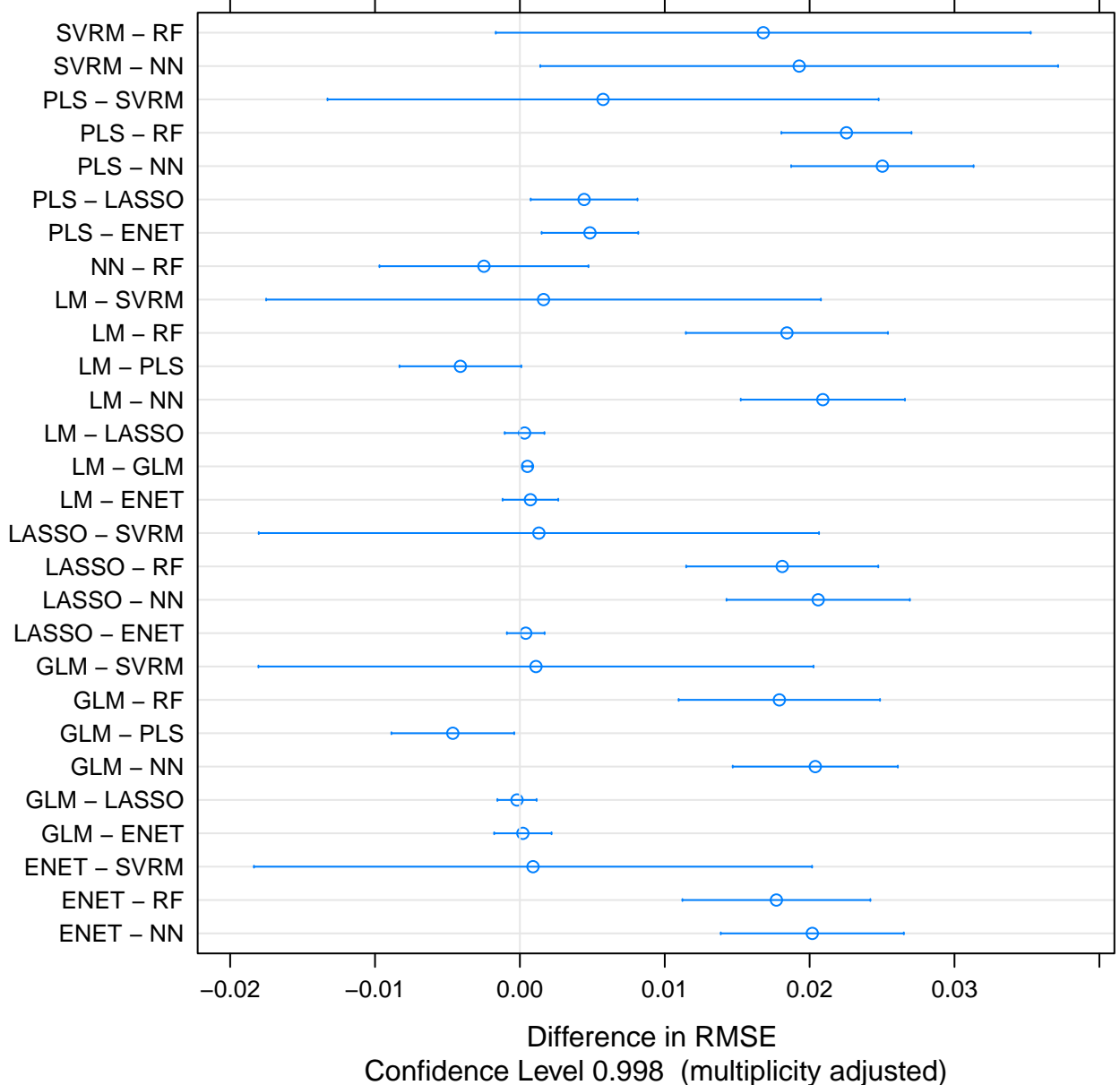

Supplement: Data S1 — Datailed results from UC Irvine Machine Learning Repository (Housing, Machine CPU, Wine Quality, Automobile and Parkinson) and the 3 Use Cases (Protein Corona, Gajewicz Metal Oxides and Aquatic Toxicity) [file peerj-04-2721-s001.zip › 2_machineCPU/DifModels.RMSE.iSplits.9.pdf]

# Resampling results on the training set (data split 1)

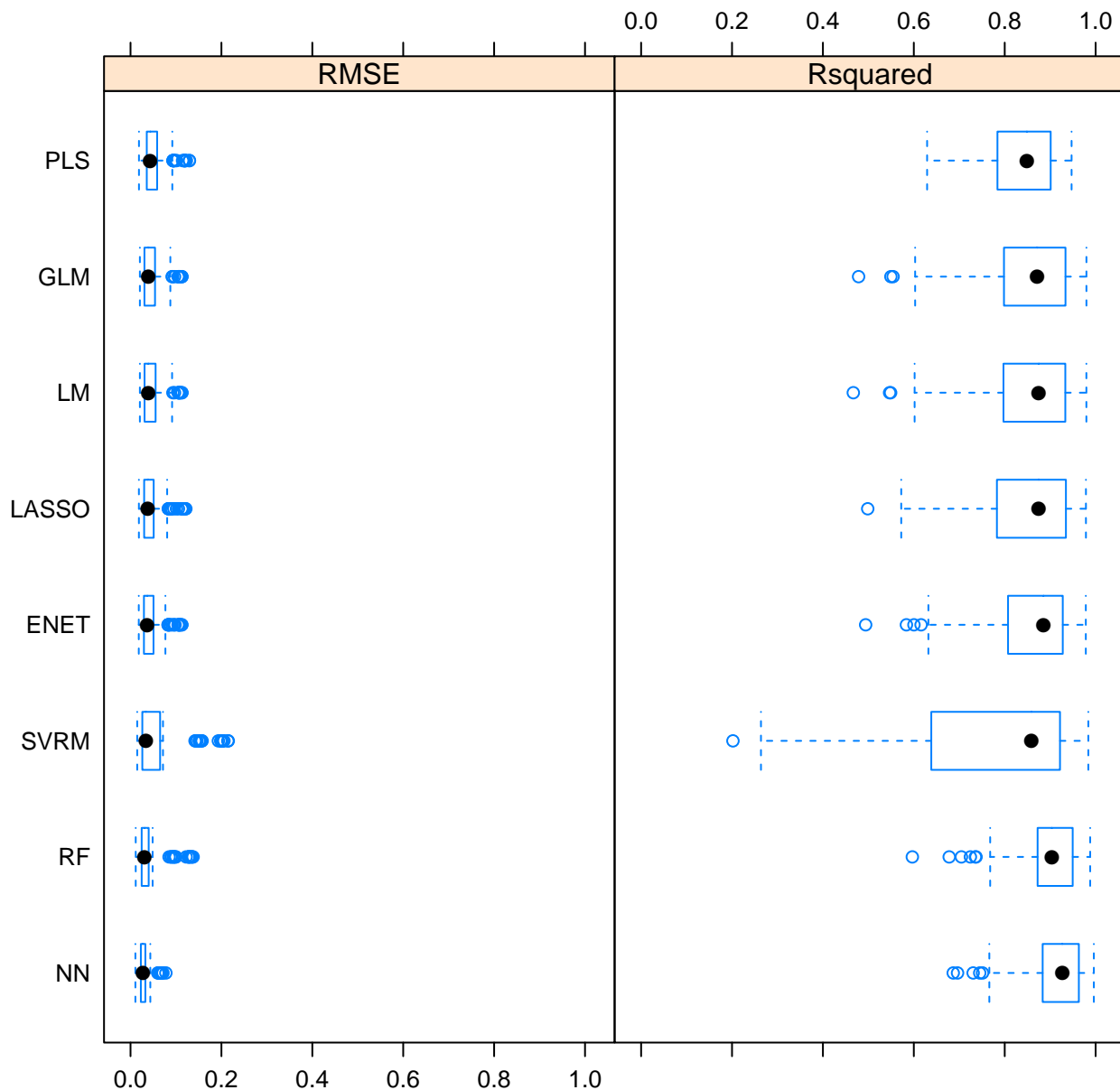

Supplement: Data S1 — Datailed results from UC Irvine Machine Learning Repository (Housing, Machine CPU, Wine Quality, Automobile and Parkinson) and the 3 Use Cases (Protein Corona, Gajewicz Metal Oxides and Aquatic Toxicity) [file peerj-04-2721-s001.zip › 2_machineCPU/ModelsComp.iSplits.1.pdf]

# Resampling results on the training set (data split 10)

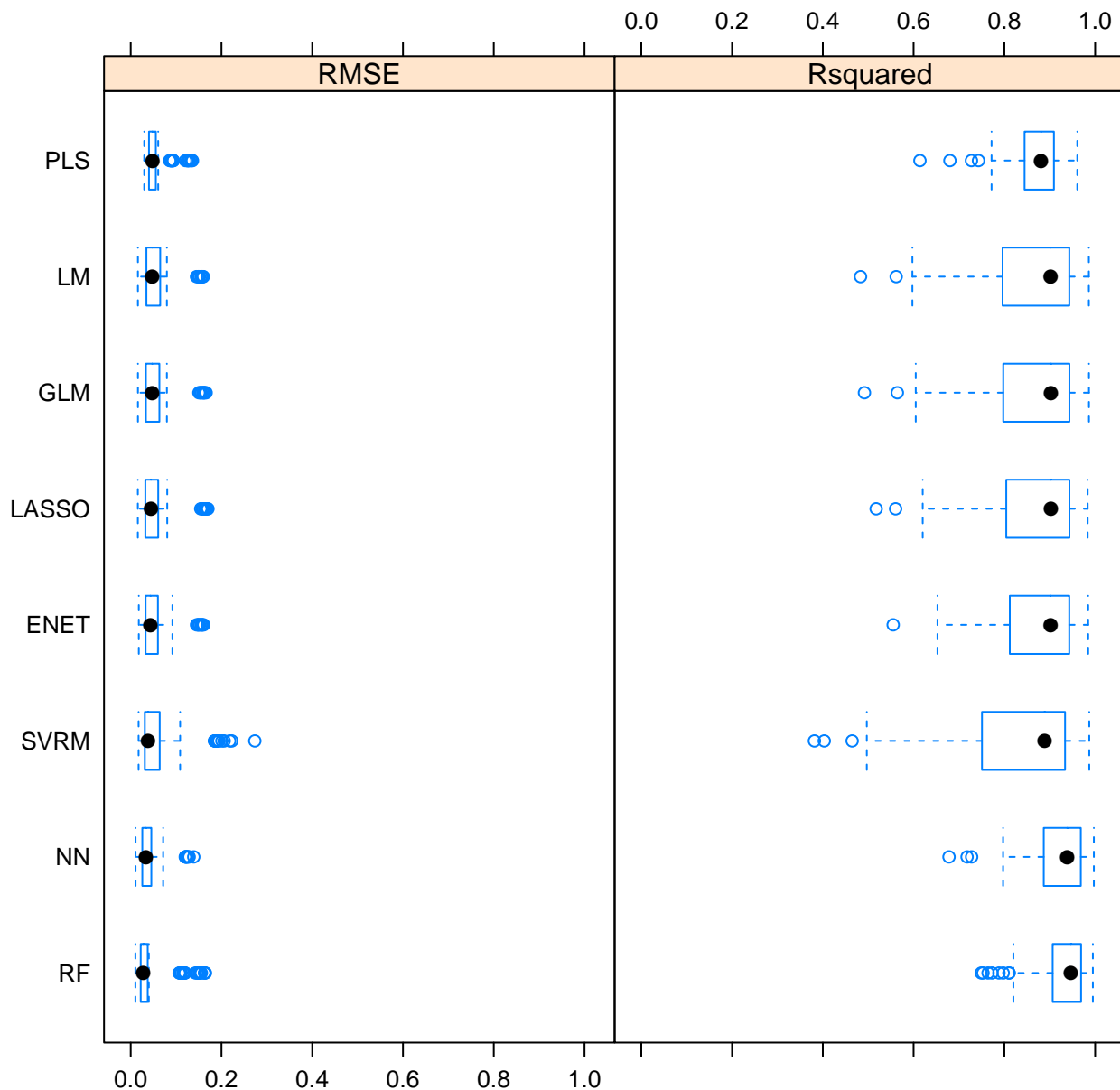

Supplement: Data S1 — Datailed results from UC Irvine Machine Learning Repository (Housing, Machine CPU, Wine Quality, Automobile and Parkinson) and the 3 Use Cases (Protein Corona, Gajewicz Metal Oxides and Aquatic Toxicity) [file peerj-04-2721-s001.zip › 2_machineCPU/ModelsComp.iSplits.10.pdf]

# Resampling results on the training set (data split 2)

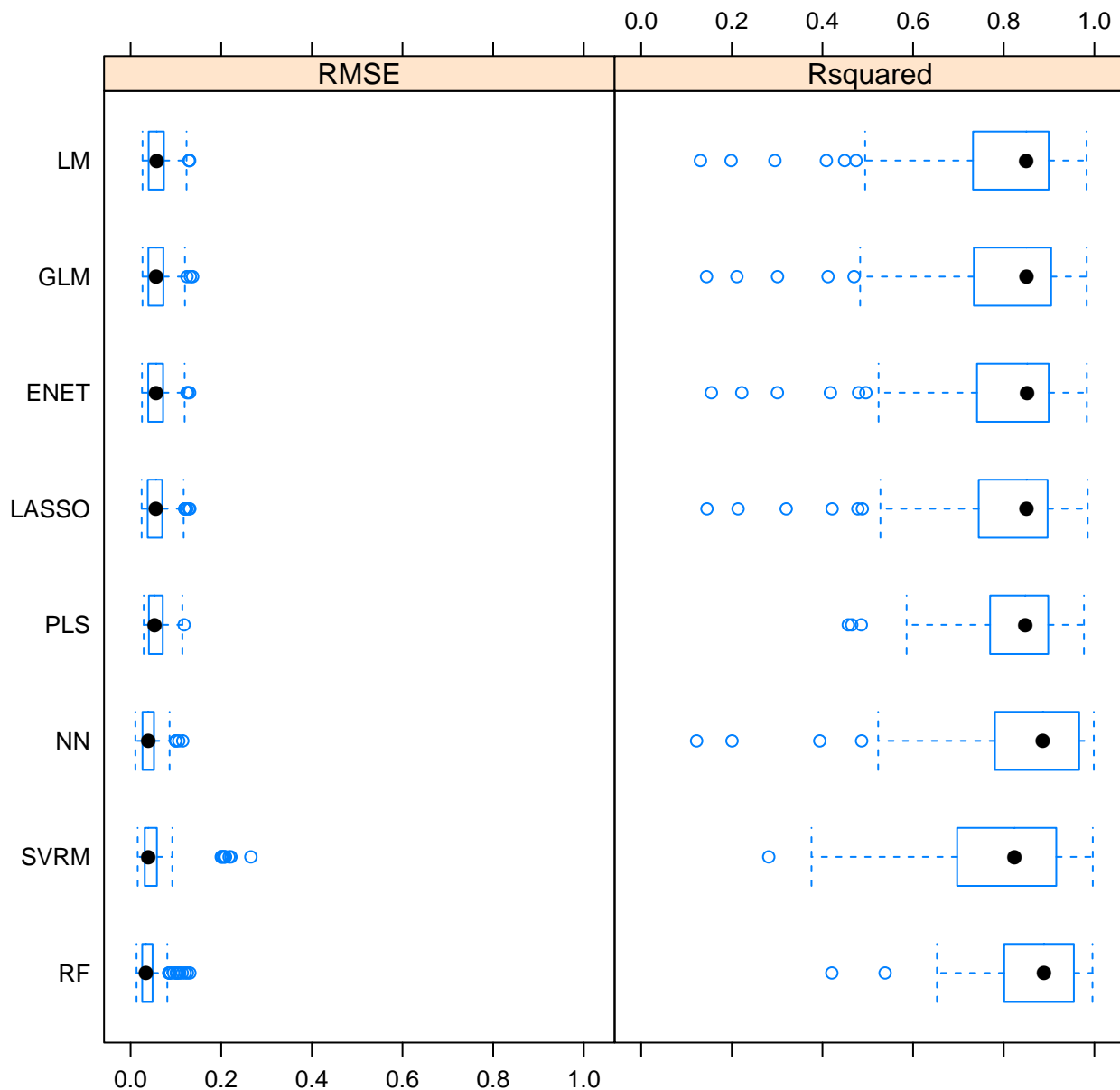

Supplement: Data S1 — Datailed results from UC Irvine Machine Learning Repository (Housing, Machine CPU, Wine Quality, Automobile and Parkinson) and the 3 Use Cases (Protein Corona, Gajewicz Metal Oxides and Aquatic Toxicity) [file peerj-04-2721-s001.zip › 2_machineCPU/ModelsComp.iSplits.2.pdf]

# Resampling results on the training set (data split 3)

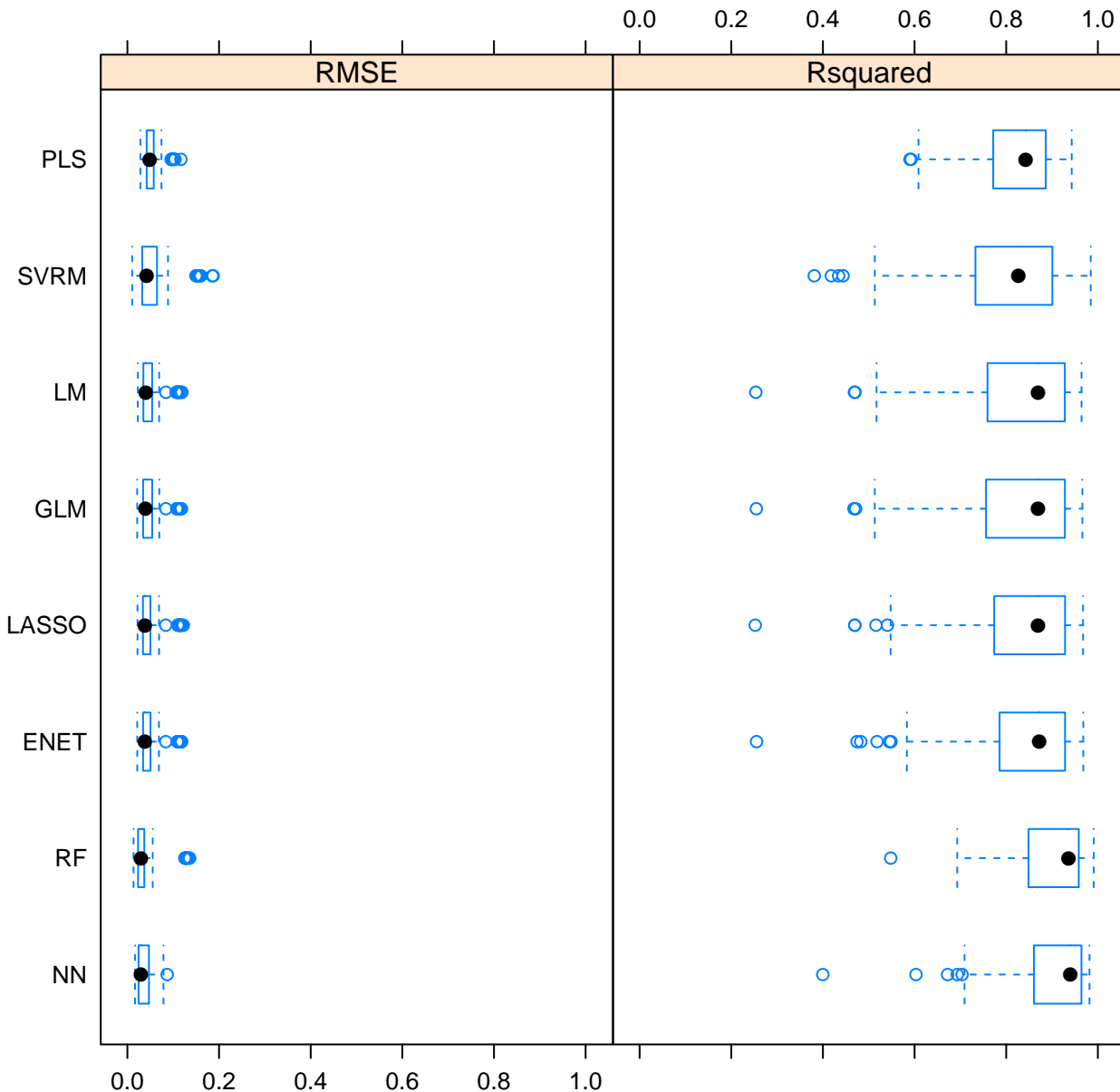

Supplement: Data S1 — Datailed results from UC Irvine Machine Learning Repository (Housing, Machine CPU, Wine Quality, Automobile and Parkinson) and the 3 Use Cases (Protein Corona, Gajewicz Metal Oxides and Aquatic Toxicity) [file peerj-04-2721-s001.zip › 2_machineCPU/ModelsComp.iSplits.3.pdf]

# Resampling results on the training set (data split 4)

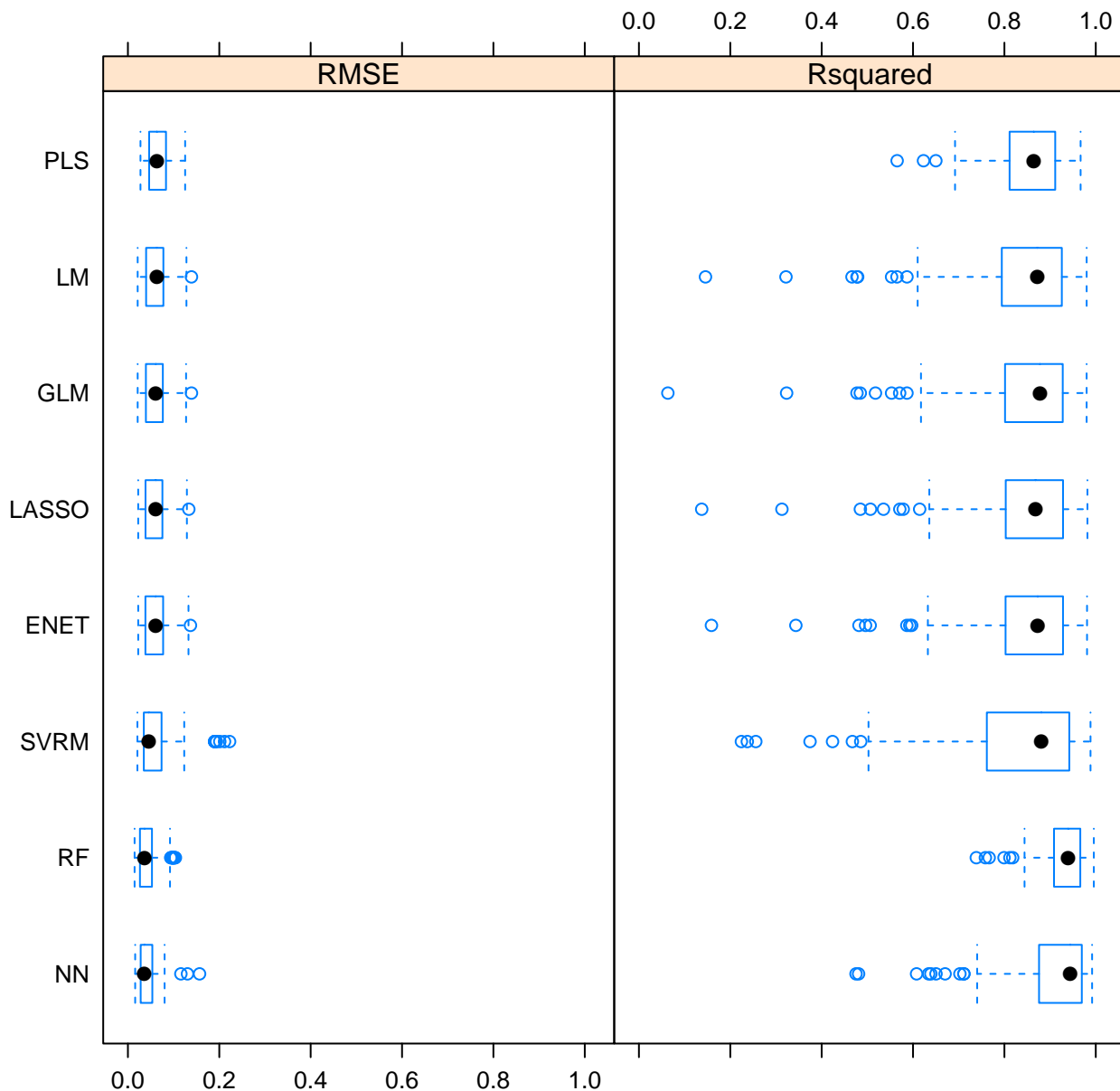

Supplement: Data S1 — Datailed results from UC Irvine Machine Learning Repository (Housing, Machine CPU, Wine Quality, Automobile and Parkinson) and the 3 Use Cases (Protein Corona, Gajewicz Metal Oxides and Aquatic Toxicity) [file peerj-04-2721-s001.zip › 2_machineCPU/ModelsComp.iSplits.4.pdf]

# Resampling results on the training set (data split 5)

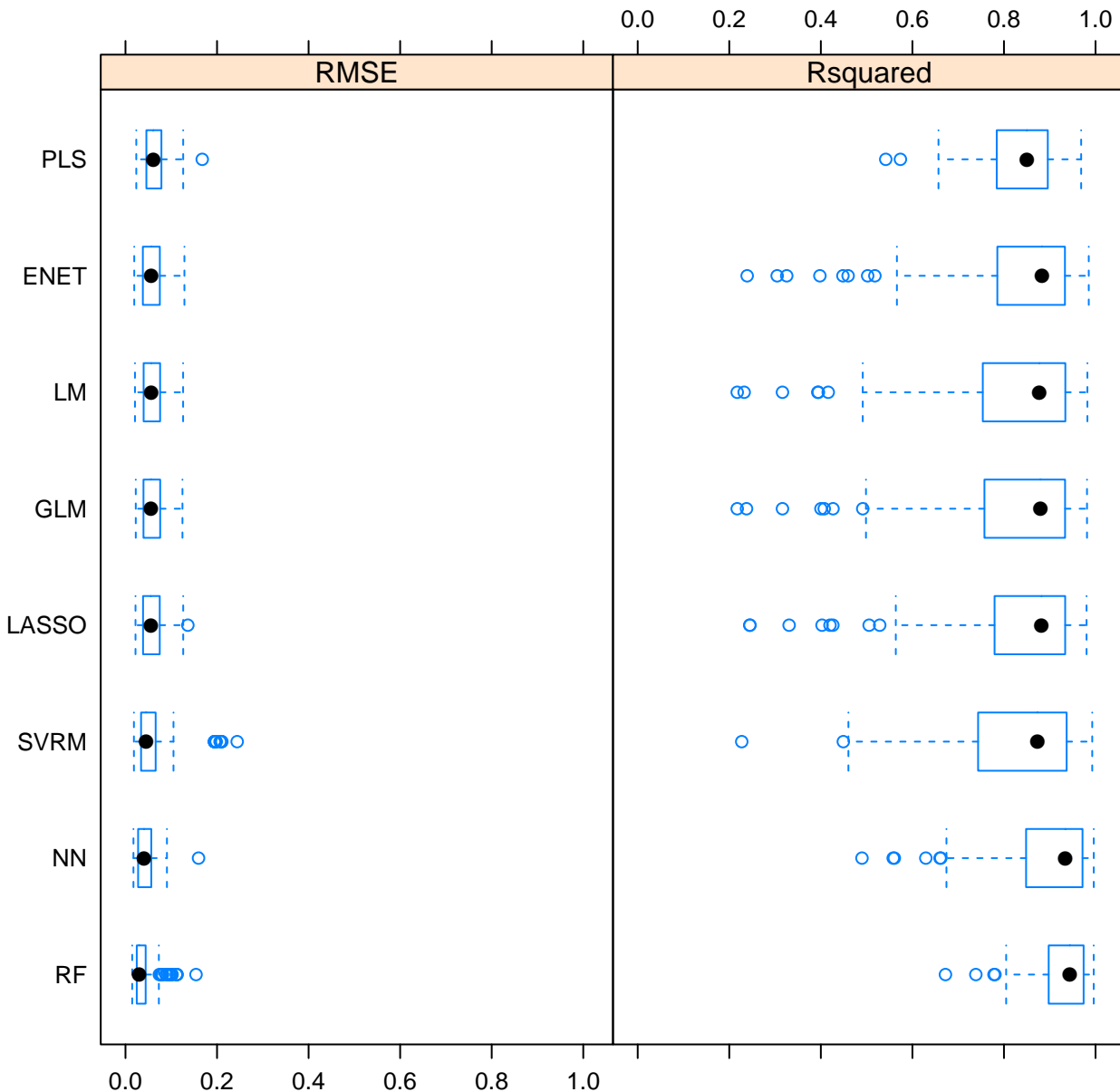

Supplement: Data S1 — Datailed results from UC Irvine Machine Learning Repository (Housing, Machine CPU, Wine Quality, Automobile and Parkinson) and the 3 Use Cases (Protein Corona, Gajewicz Metal Oxides and Aquatic Toxicity) [file peerj-04-2721-s001.zip › 2_machineCPU/ModelsComp.iSplits.5.pdf]

# Resampling results on the training set (data split 6)

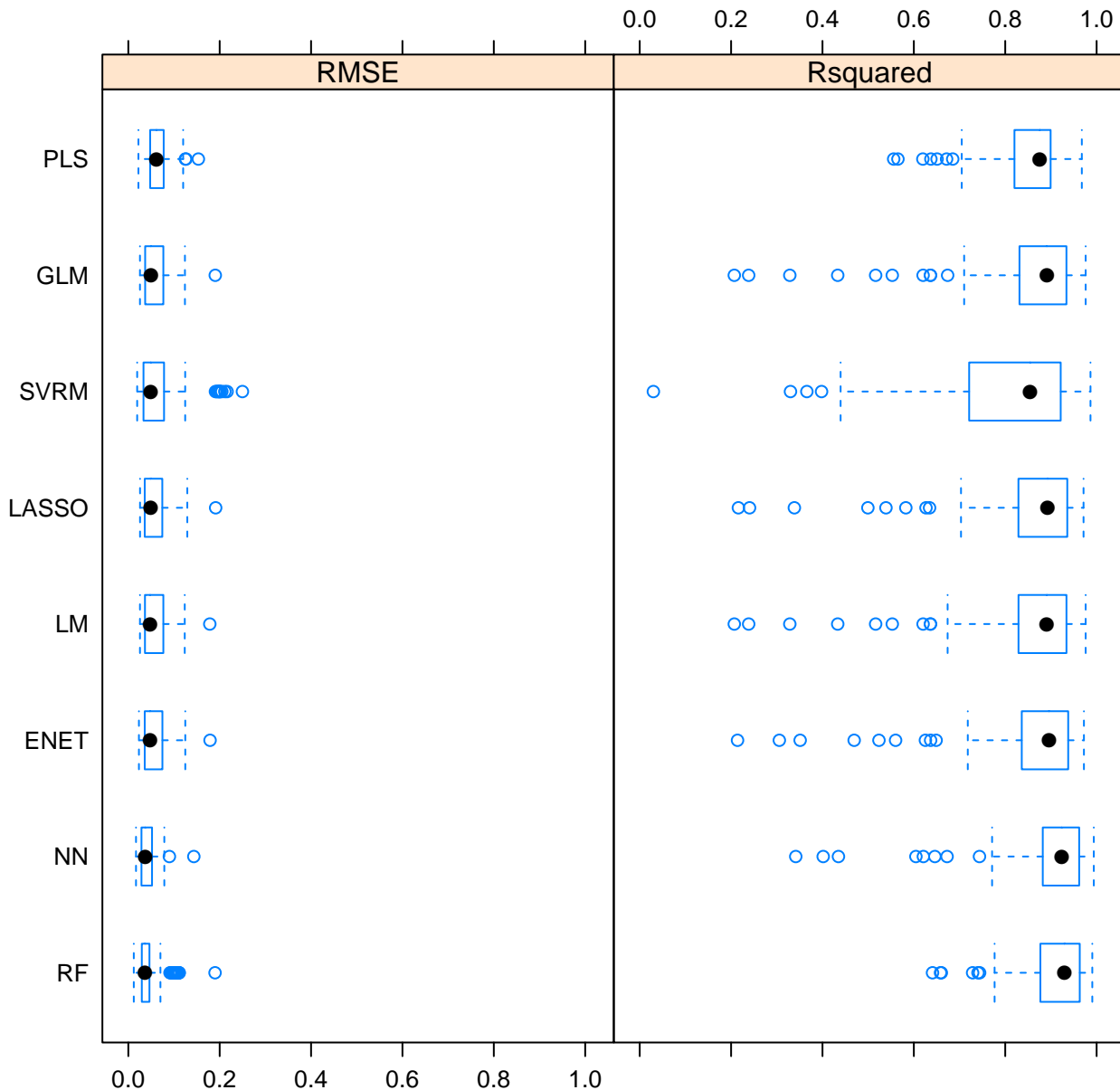

Supplement: Data S1 — Datailed results from UC Irvine Machine Learning Repository (Housing, Machine CPU, Wine Quality, Automobile and Parkinson) and the 3 Use Cases (Protein Corona, Gajewicz Metal Oxides and Aquatic Toxicity) [file peerj-04-2721-s001.zip › 2_machineCPU/ModelsComp.iSplits.6.pdf]

# Resampling results on the training set (data split 7)

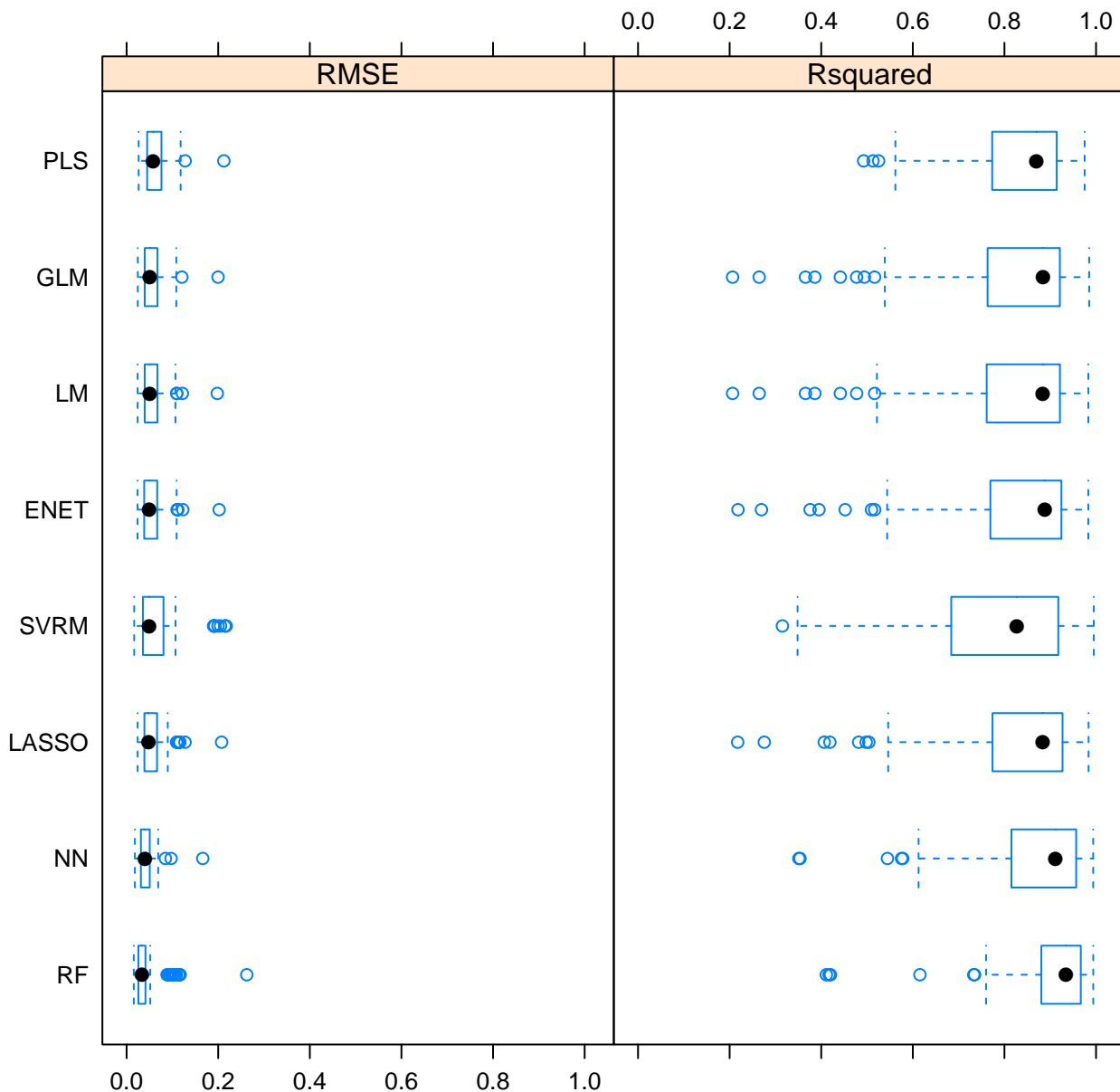

Supplement: Data S1 — Datailed results from UC Irvine Machine Learning Repository (Housing, Machine CPU, Wine Quality, Automobile and Parkinson) and the 3 Use Cases (Protein Corona, Gajewicz Metal Oxides and Aquatic Toxicity) [file peerj-04-2721-s001.zip › 2_machineCPU/ModelsComp.iSplits.7.pdf]

# Resampling results on the training set (data split 8)

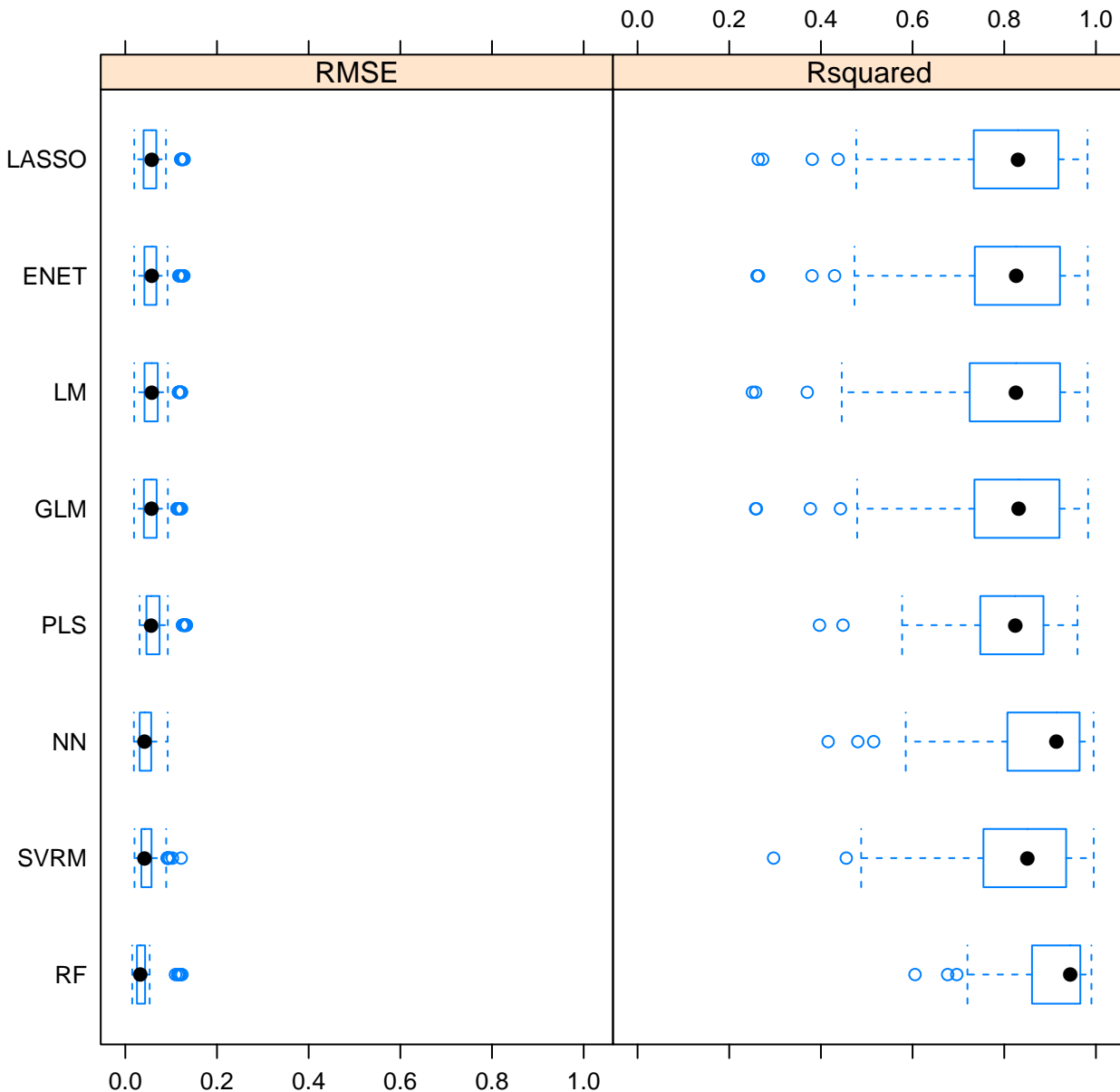

Supplement: Data S1 — Datailed results from UC Irvine Machine Learning Repository (Housing, Machine CPU, Wine Quality, Automobile and Parkinson) and the 3 Use Cases (Protein Corona, Gajewicz Metal Oxides and Aquatic Toxicity) [file peerj-04-2721-s001.zip › 2_machineCPU/ModelsComp.iSplits.8.pdf]

# Resampling results on the training set (data split 9)

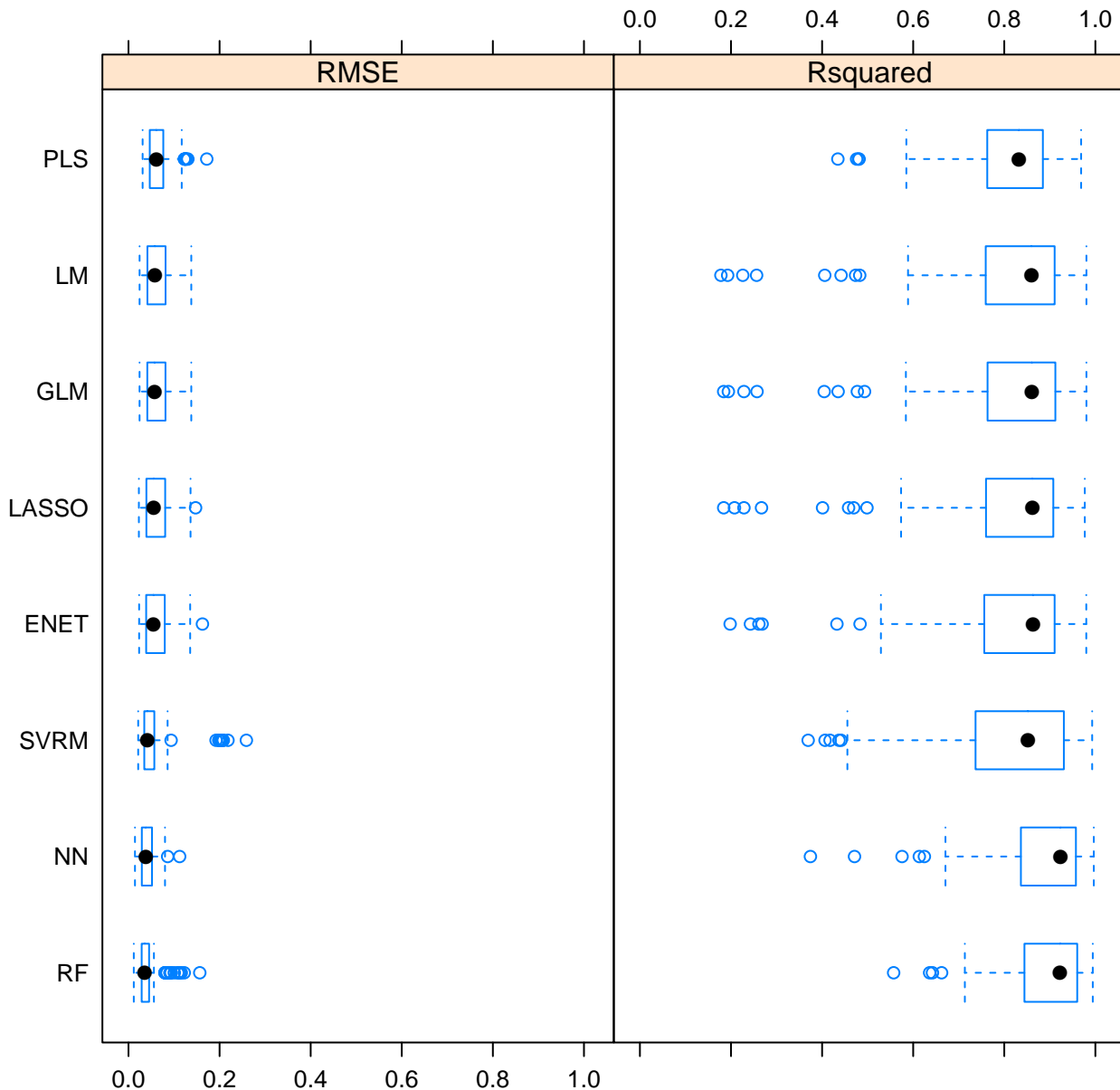

Supplement: Data S1 — Datailed results from UC Irvine Machine Learning Repository (Housing, Machine CPU, Wine Quality, Automobile and Parkinson) and the 3 Use Cases (Protein Corona, Gajewicz Metal Oxides and Aquatic Toxicity) [file peerj-04-2721-s001.zip › 2_machineCPU/ModelsComp.iSplits.9.pdf]

# Models' differences on the training set (data split 1)

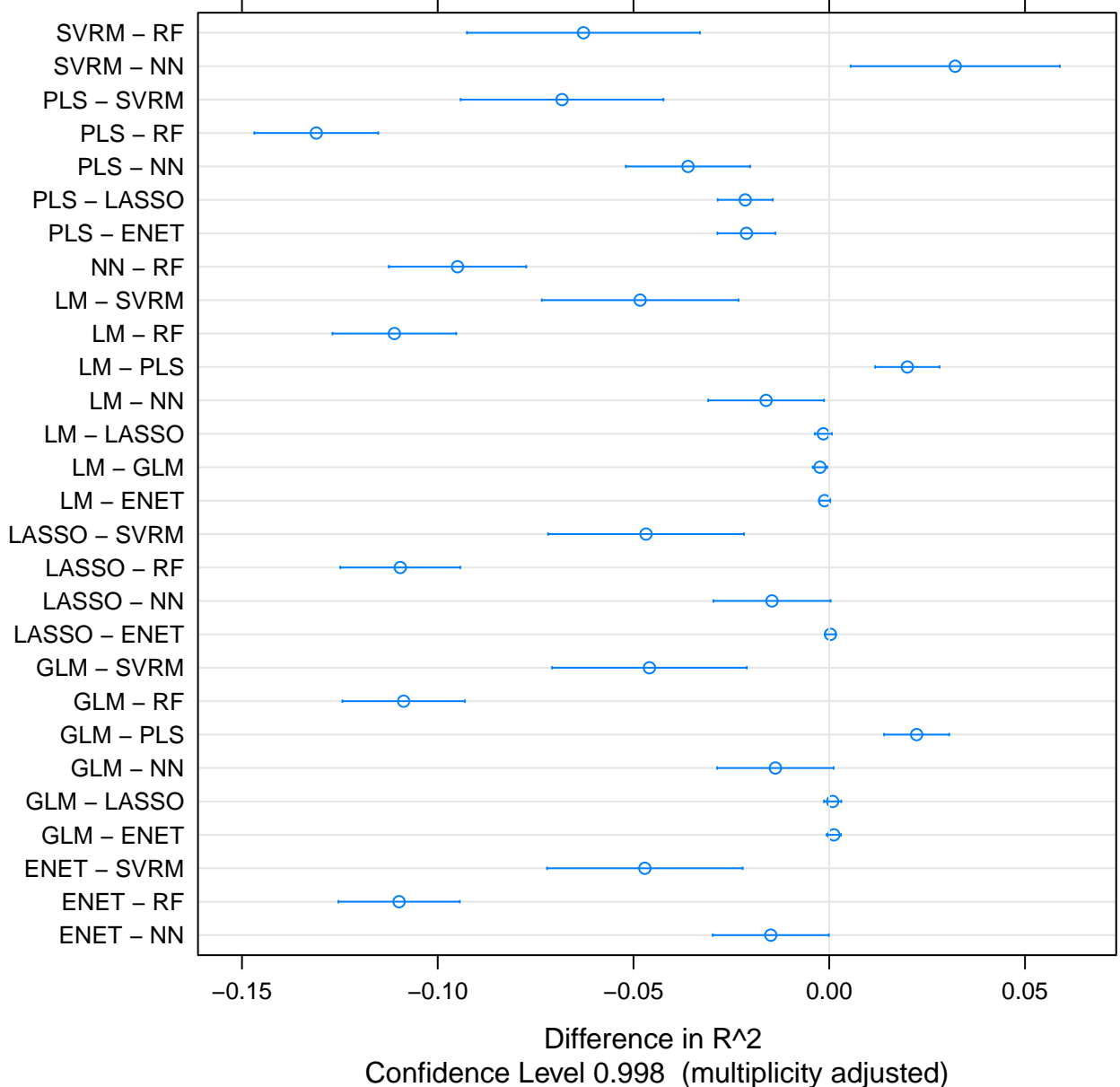

Supplement: Data S1 — Datailed results from UC Irvine Machine Learning Repository (Housing, Machine CPU, Wine Quality, Automobile and Parkinson) and the 3 Use Cases (Protein Corona, Gajewicz Metal Oxides and Aquatic Toxicity) [file peerj-04-2721-s001.zip › 3_winequality/DifModels.R2.iSplits.1.pdf]

# Models' differences on the training set (data split 10)

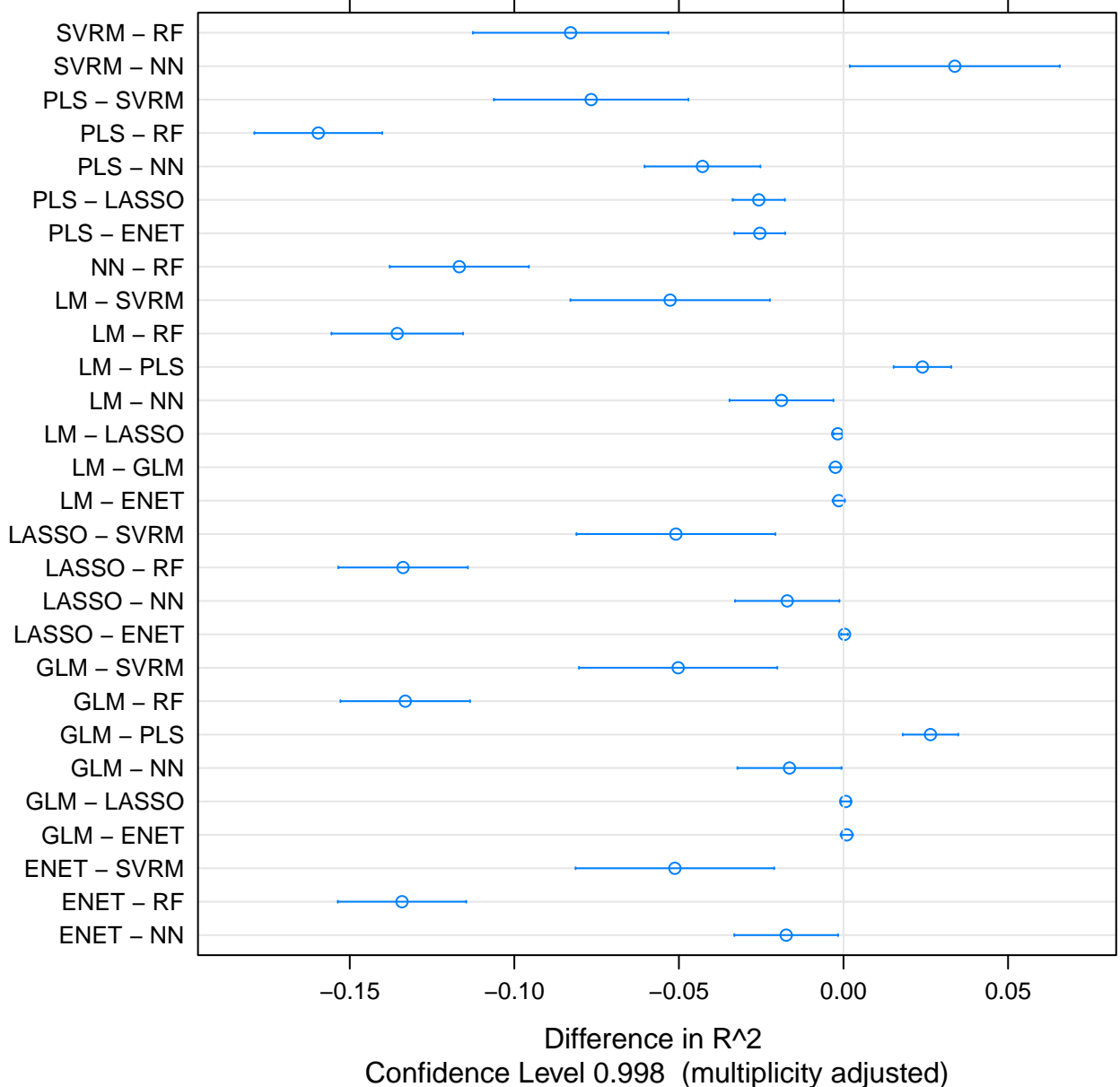

Supplement: Data S1 — Datailed results from UC Irvine Machine Learning Repository (Housing, Machine CPU, Wine Quality, Automobile and Parkinson) and the 3 Use Cases (Protein Corona, Gajewicz Metal Oxides and Aquatic Toxicity) [file peerj-04-2721-s001.zip › 3_winequality/DifModels.R2.iSplits.10.pdf]

# Models' differences on the training set (data split 2)

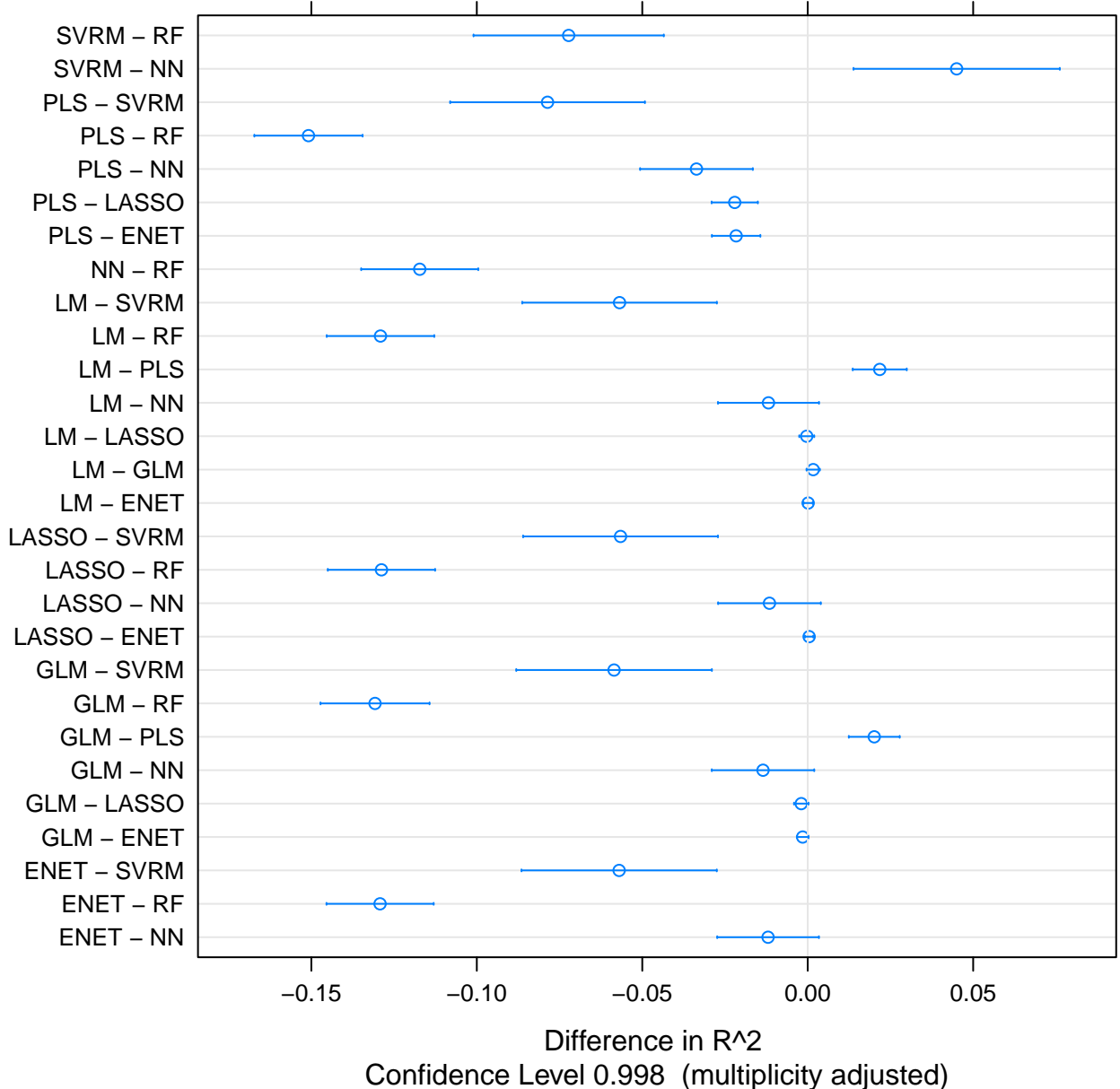

Supplement: Data S1 — Datailed results from UC Irvine Machine Learning Repository (Housing, Machine CPU, Wine Quality, Automobile and Parkinson) and the 3 Use Cases (Protein Corona, Gajewicz Metal Oxides and Aquatic Toxicity) [file peerj-04-2721-s001.zip › 3_winequality/DifModels.R2.iSplits.2.pdf]

# Models' differences on the training set (data split 3)

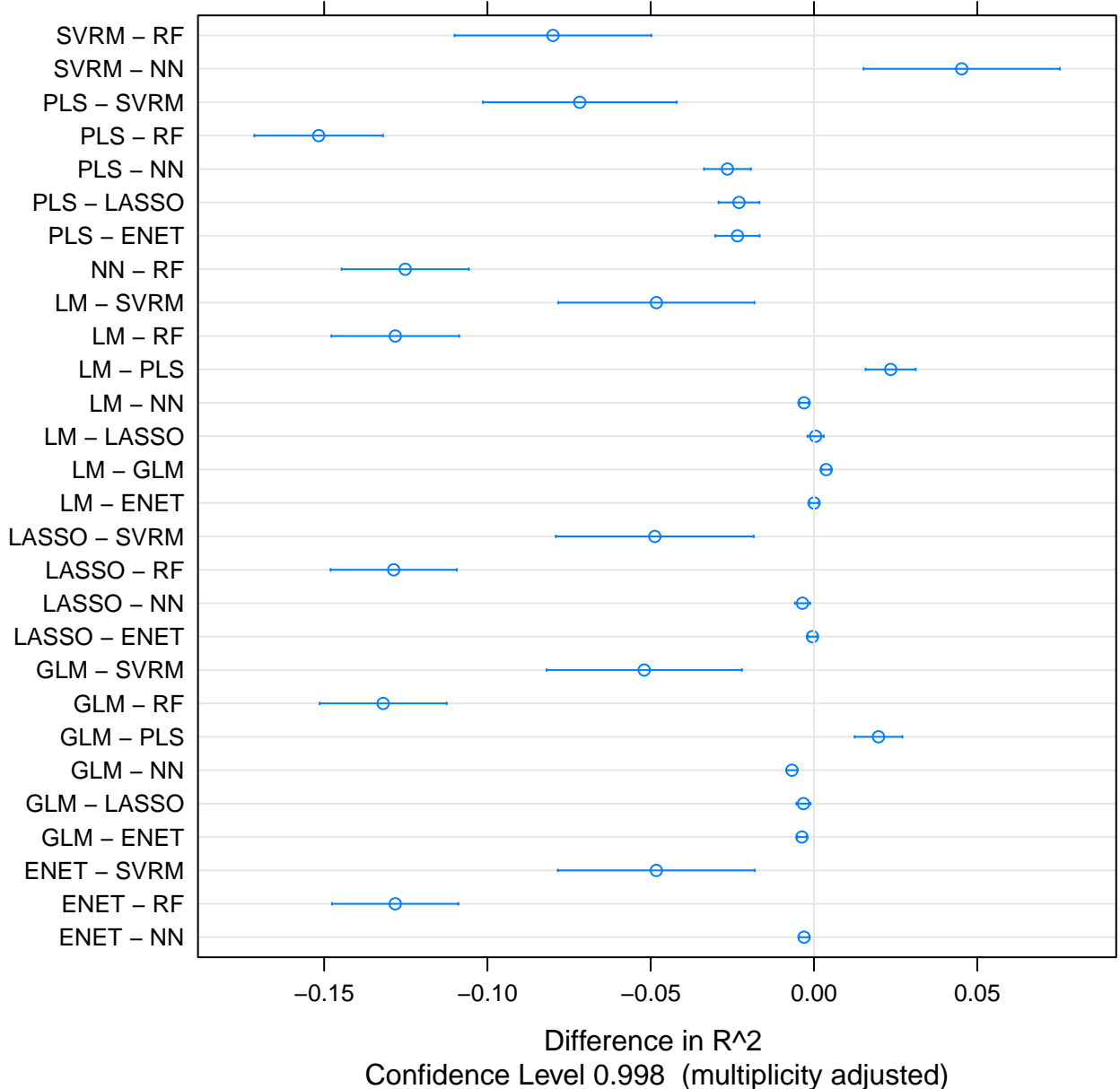

Supplement: Data S1 — Datailed results from UC Irvine Machine Learning Repository (Housing, Machine CPU, Wine Quality, Automobile and Parkinson) and the 3 Use Cases (Protein Corona, Gajewicz Metal Oxides and Aquatic Toxicity) [file peerj-04-2721-s001.zip › 3_winequality/DifModels.R2.iSplits.3.pdf]

# Models' differences on the training set (data split 4)

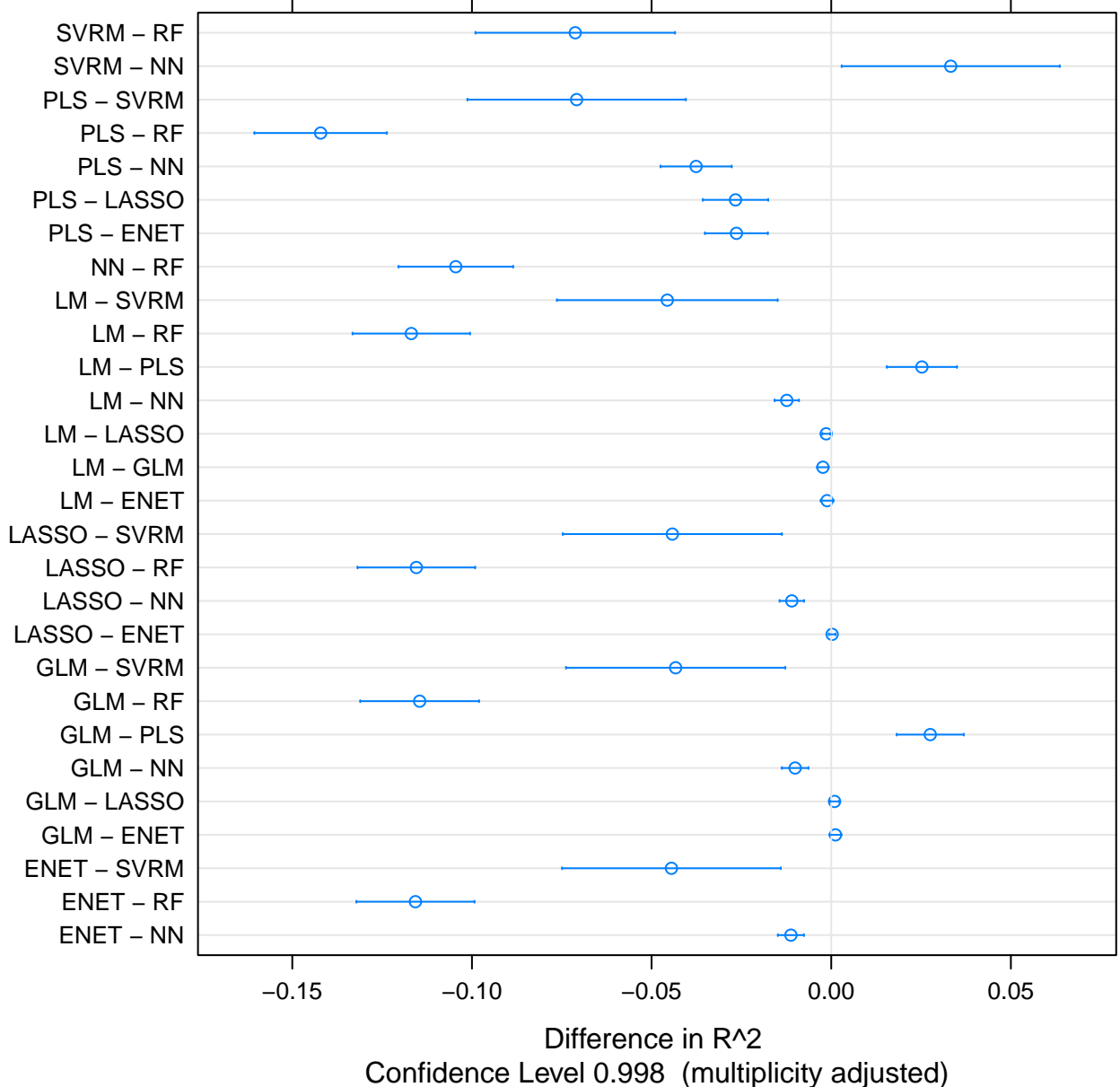

Supplement: Data S1 — Datailed results from UC Irvine Machine Learning Repository (Housing, Machine CPU, Wine Quality, Automobile and Parkinson) and the 3 Use Cases (Protein Corona, Gajewicz Metal Oxides and Aquatic Toxicity) [file peerj-04-2721-s001.zip › 3_winequality/DifModels.R2.iSplits.4.pdf]

# Models' differences on the training set (data split 5)

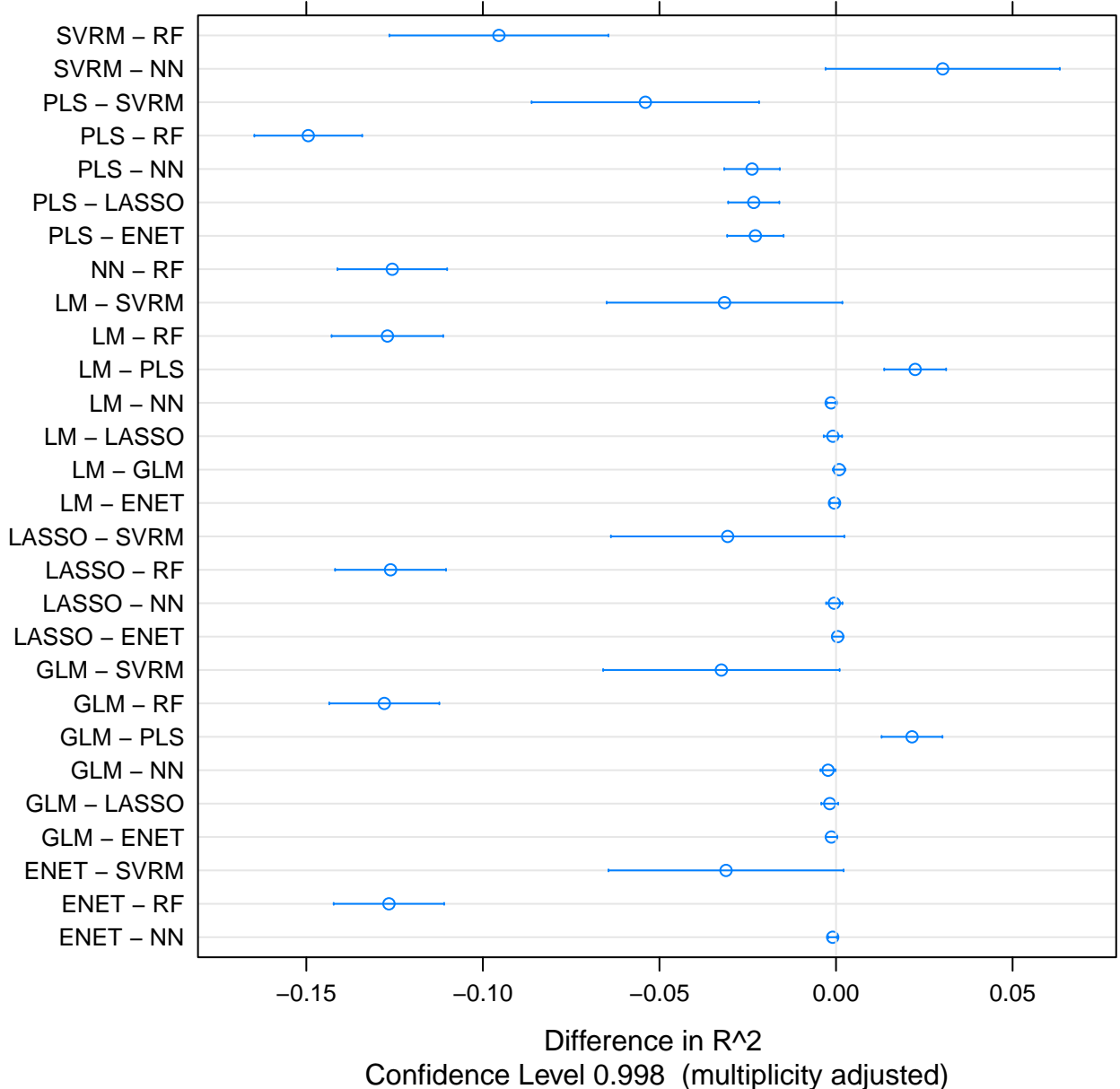

Supplement: Data S1 — Datailed results from UC Irvine Machine Learning Repository (Housing, Machine CPU, Wine Quality, Automobile and Parkinson) and the 3 Use Cases (Protein Corona, Gajewicz Metal Oxides and Aquatic Toxicity) [file peerj-04-2721-s001.zip › 3_winequality/DifModels.R2.iSplits.5.pdf]

# Models' differences on the training set (data split 6)

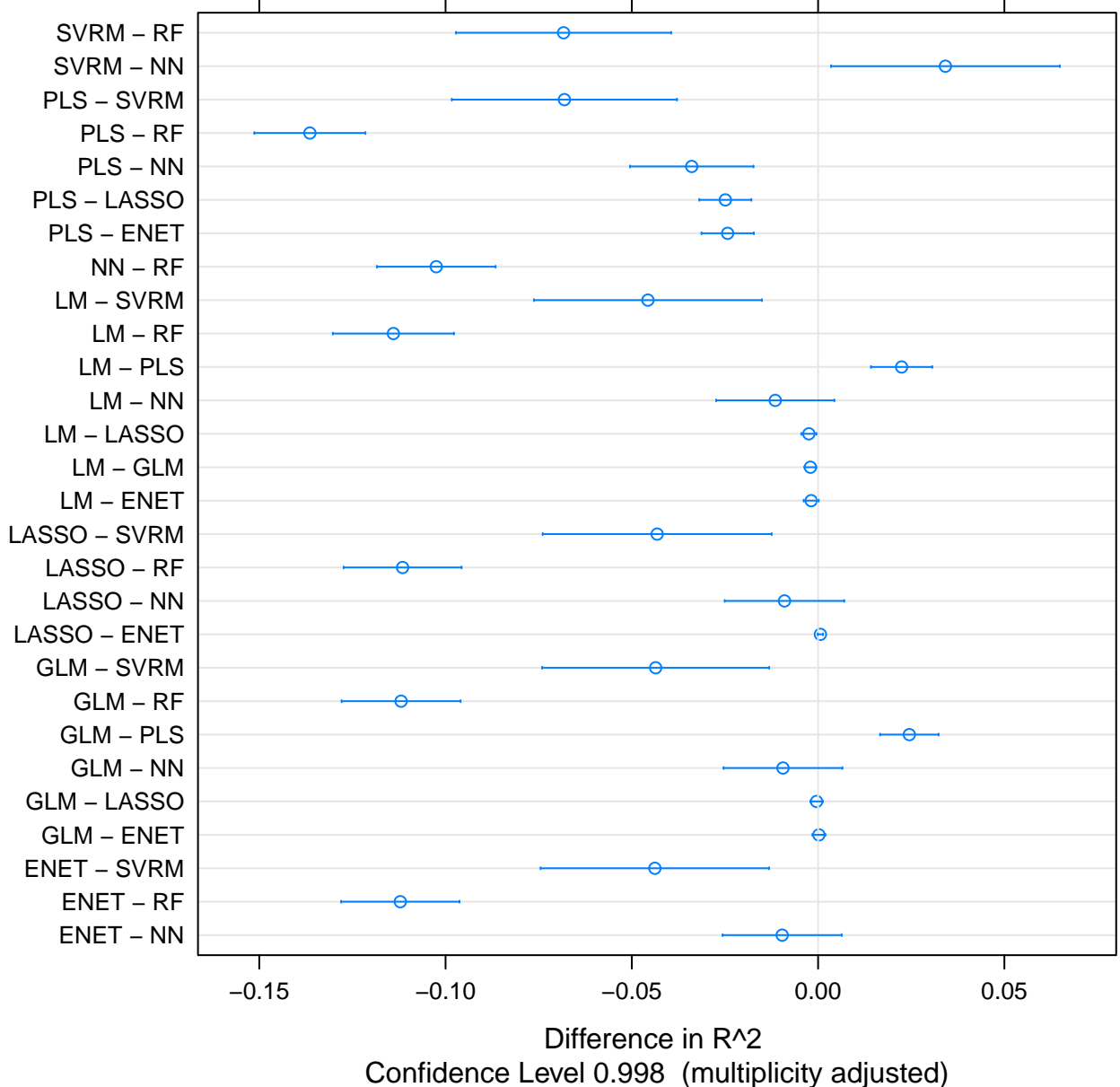

Supplement: Data S1 — Datailed results from UC Irvine Machine Learning Repository (Housing, Machine CPU, Wine Quality, Automobile and Parkinson) and the 3 Use Cases (Protein Corona, Gajewicz Metal Oxides and Aquatic Toxicity) [file peerj-04-2721-s001.zip › 3_winequality/DifModels.R2.iSplits.6.pdf]

# Models' differences on the training set (data split 7)

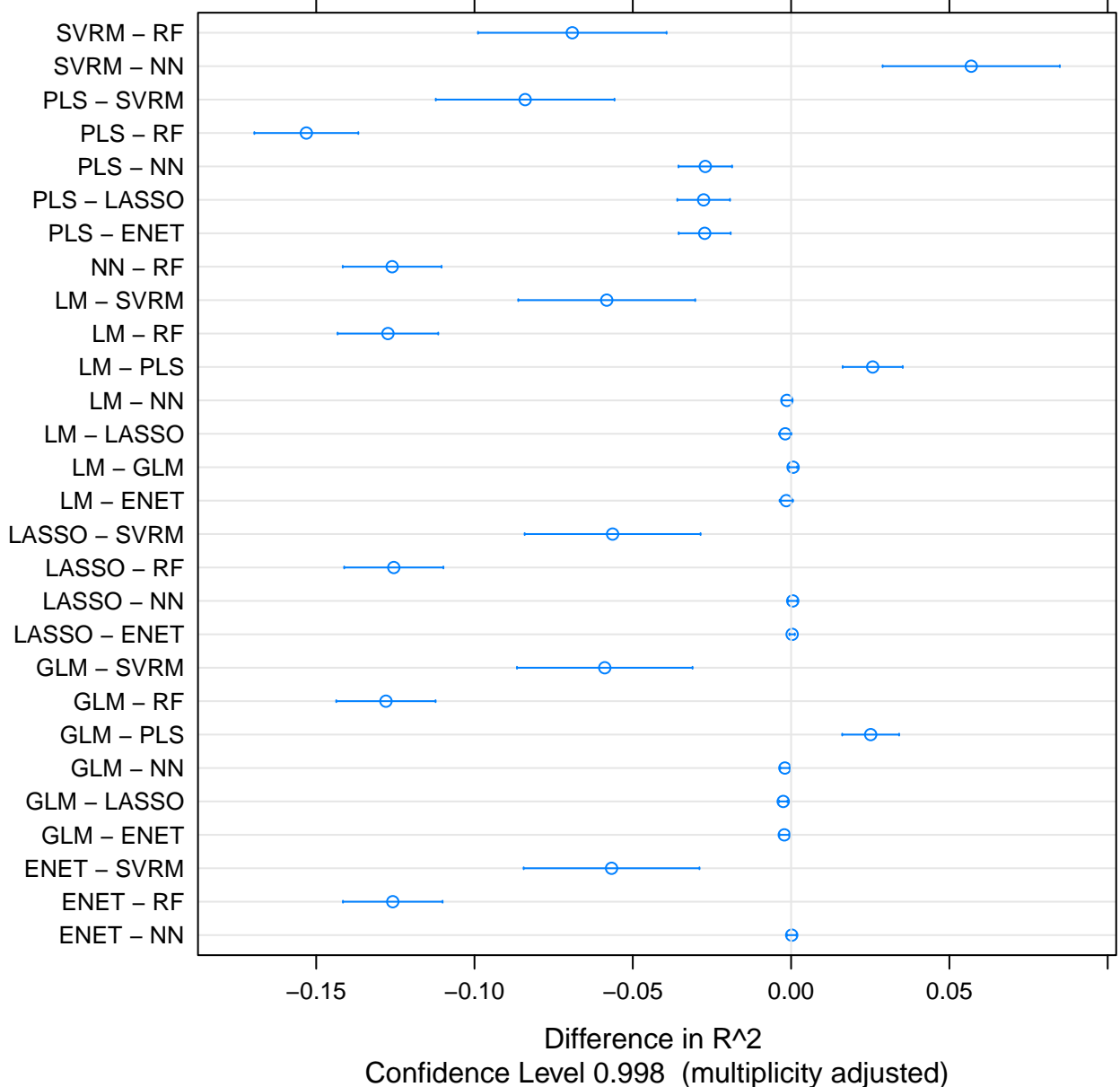

Supplement: Data S1 — Datailed results from UC Irvine Machine Learning Repository (Housing, Machine CPU, Wine Quality, Automobile and Parkinson) and the 3 Use Cases (Protein Corona, Gajewicz Metal Oxides and Aquatic Toxicity) [file peerj-04-2721-s001.zip › 3_winequality/DifModels.R2.iSplits.7.pdf]

# Models' differences on the training set (data split 8)

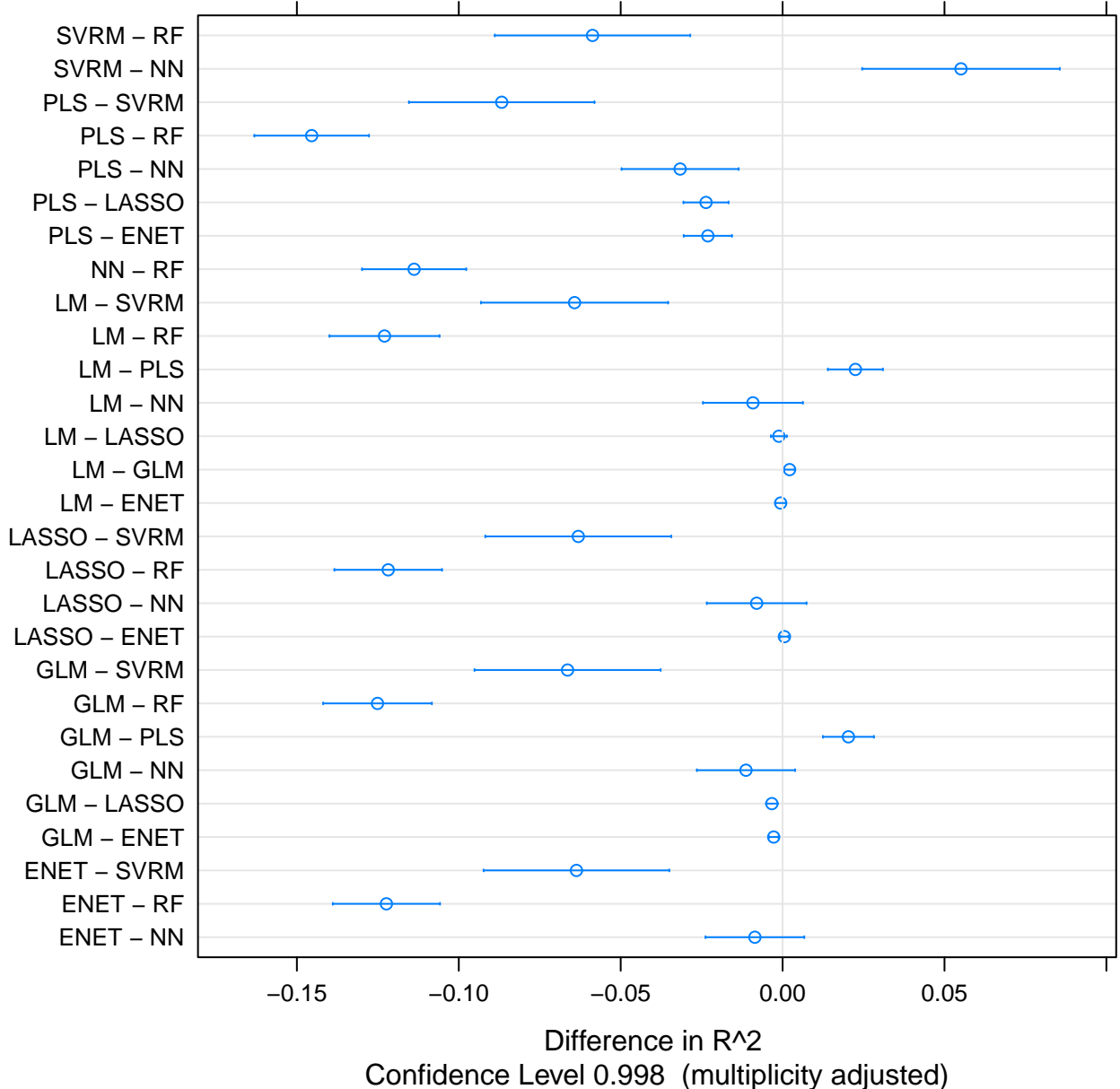

Supplement: Data S1 — Datailed results from UC Irvine Machine Learning Repository (Housing, Machine CPU, Wine Quality, Automobile and Parkinson) and the 3 Use Cases (Protein Corona, Gajewicz Metal Oxides and Aquatic Toxicity) [file peerj-04-2721-s001.zip › 3_winequality/DifModels.R2.iSplits.8.pdf]

# Models' differences on the training set (data split 9)

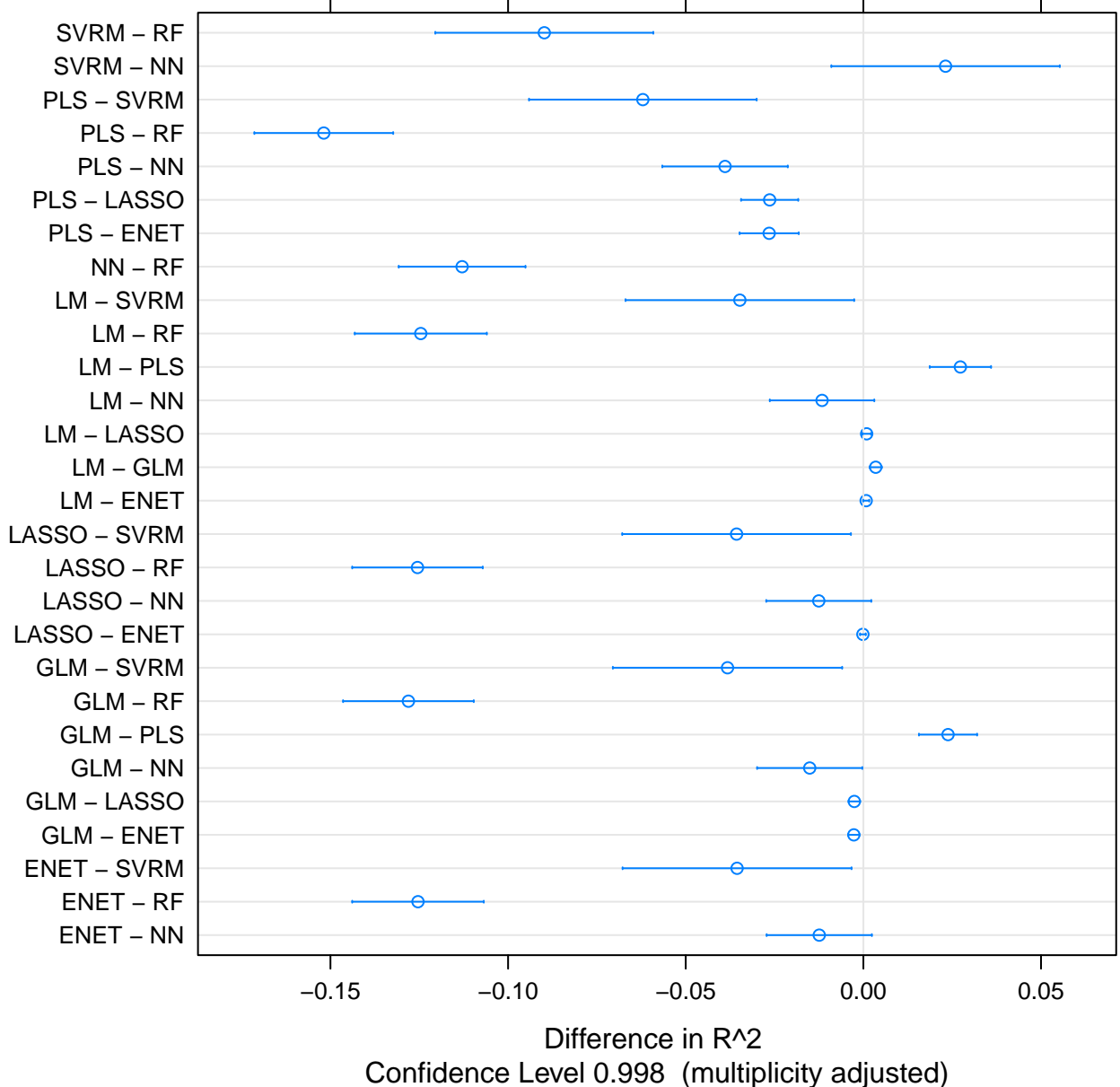

Supplement: Data S1 — Datailed results from UC Irvine Machine Learning Repository (Housing, Machine CPU, Wine Quality, Automobile and Parkinson) and the 3 Use Cases (Protein Corona, Gajewicz Metal Oxides and Aquatic Toxicity) [file peerj-04-2721-s001.zip › 3_winequality/DifModels.R2.iSplits.9.pdf]

# Models' differences on the training set (data split 1)

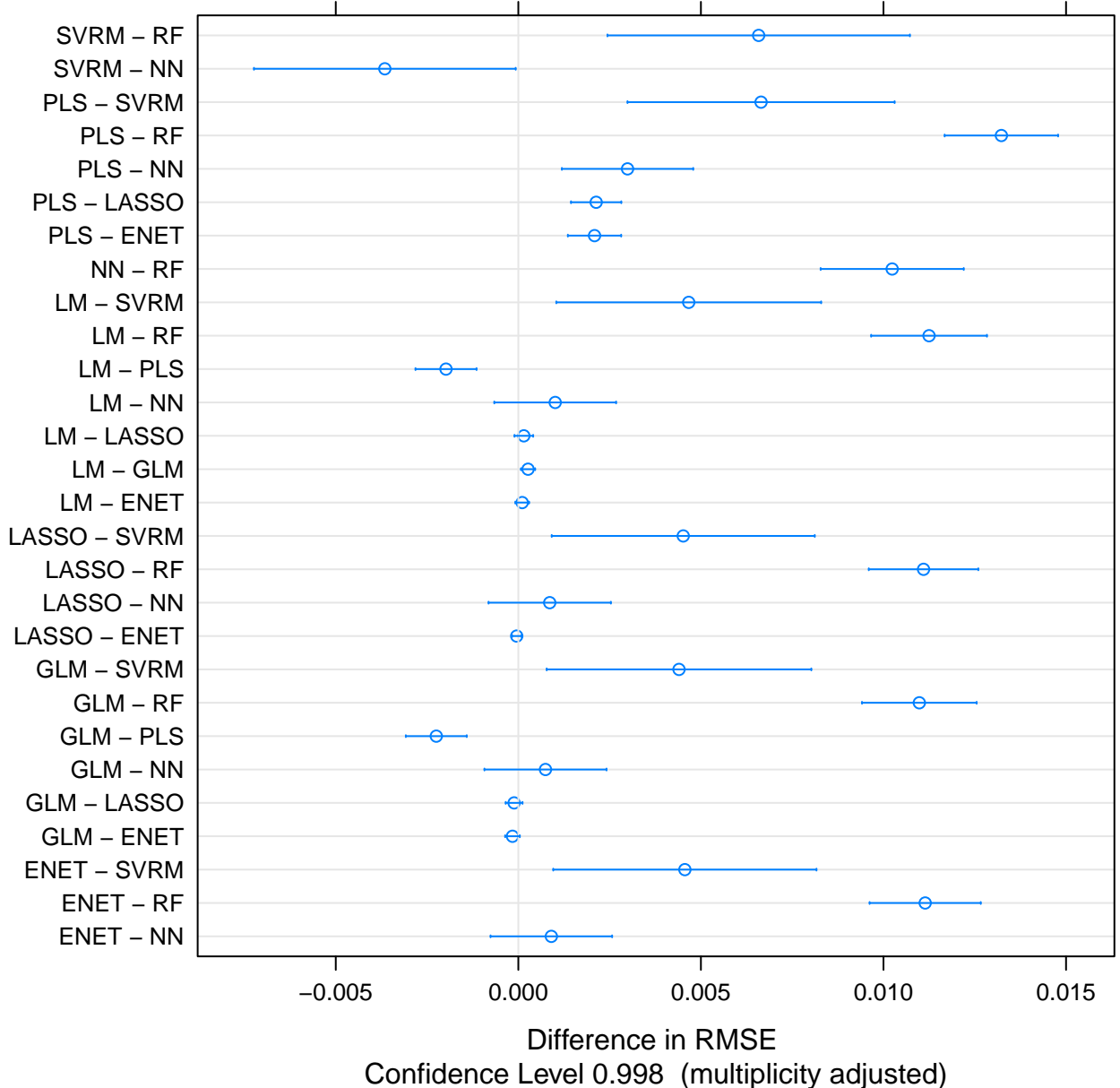

Supplement: Data S1 — Datailed results from UC Irvine Machine Learning Repository (Housing, Machine CPU, Wine Quality, Automobile and Parkinson) and the 3 Use Cases (Protein Corona, Gajewicz Metal Oxides and Aquatic Toxicity) [file peerj-04-2721-s001.zip › 3_winequality/DifModels.RMSE.iSplits.1.pdf]

# Models' differences on the training set (data split 10)

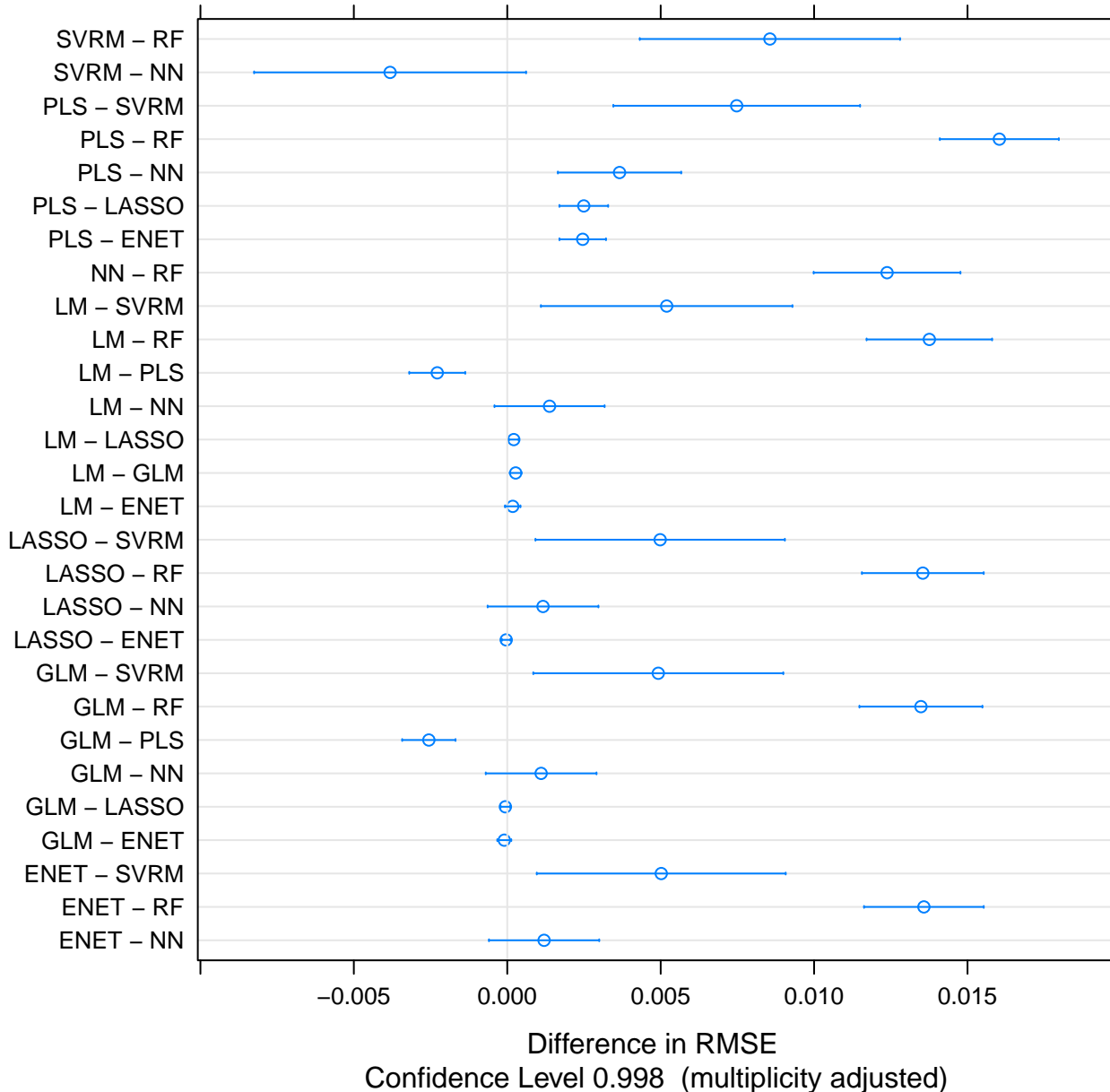

Supplement: Data S1 — Datailed results from UC Irvine Machine Learning Repository (Housing, Machine CPU, Wine Quality, Automobile and Parkinson) and the 3 Use Cases (Protein Corona, Gajewicz Metal Oxides and Aquatic Toxicity) [file peerj-04-2721-s001.zip › 3_winequality/DifModels.RMSE.iSplits.10.pdf]

# Models' differences on the training set (data split 2)

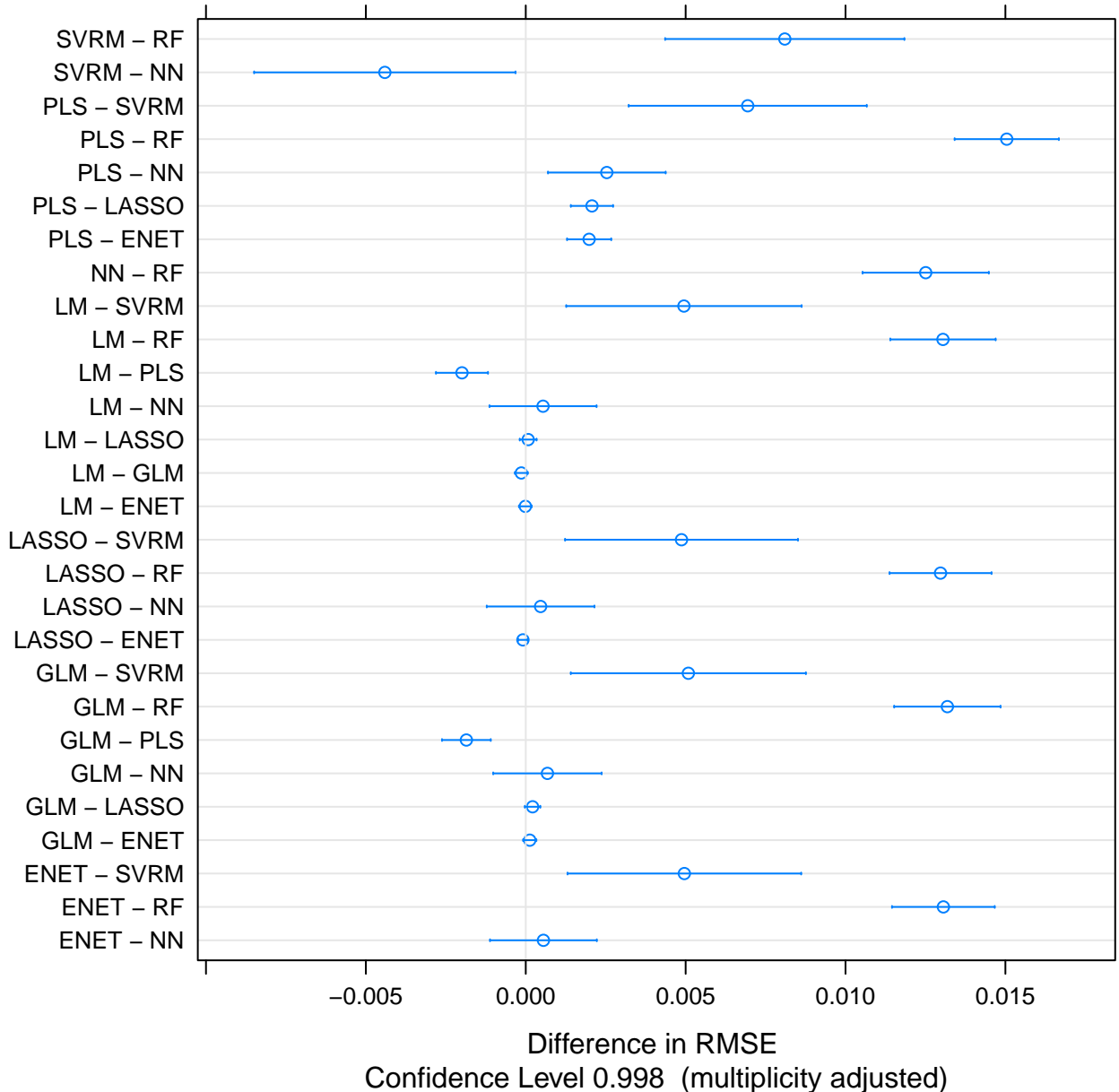

Supplement: Data S1 — Datailed results from UC Irvine Machine Learning Repository (Housing, Machine CPU, Wine Quality, Automobile and Parkinson) and the 3 Use Cases (Protein Corona, Gajewicz Metal Oxides and Aquatic Toxicity) [file peerj-04-2721-s001.zip › 3_winequality/DifModels.RMSE.iSplits.2.pdf]

# Models' differences on the training set (data split 3)

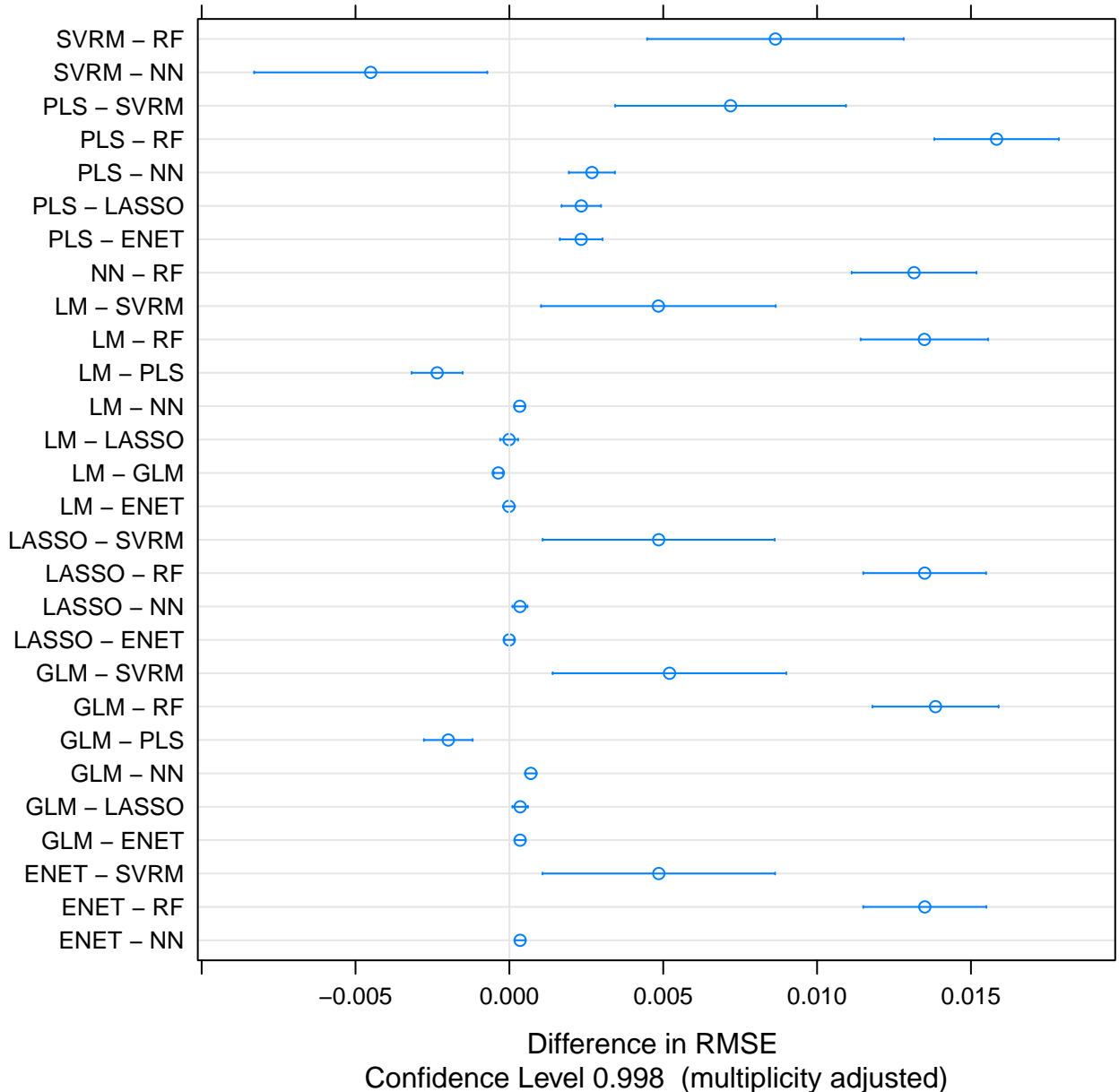

Supplement: Data S1 — Datailed results from UC Irvine Machine Learning Repository (Housing, Machine CPU, Wine Quality, Automobile and Parkinson) and the 3 Use Cases (Protein Corona, Gajewicz Metal Oxides and Aquatic Toxicity) [file peerj-04-2721-s001.zip › 3_winequality/DifModels.RMSE.iSplits.3.pdf]

# Models' differences on the training set (data split 4)

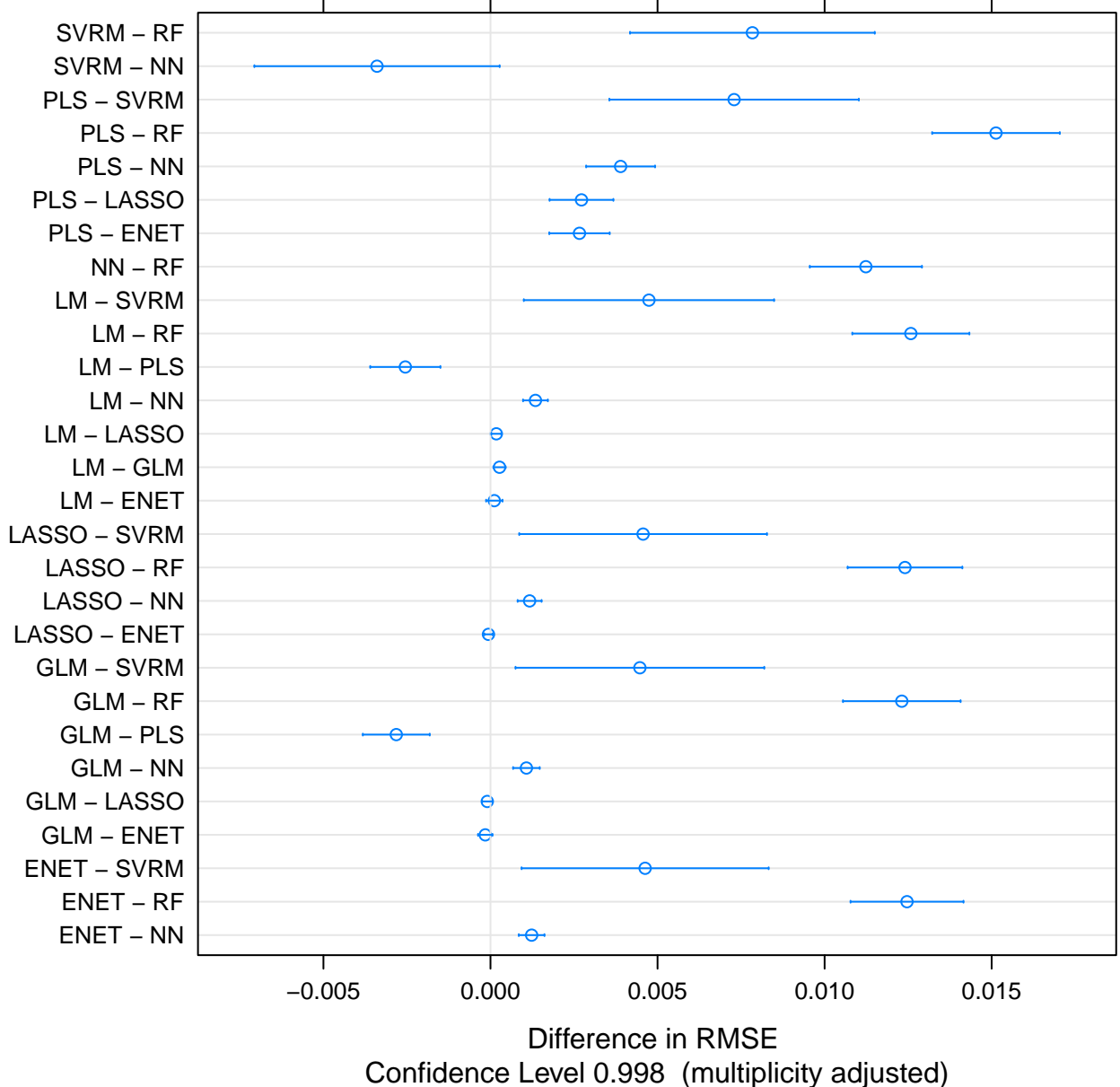

Supplement: Data S1 — Datailed results from UC Irvine Machine Learning Repository (Housing, Machine CPU, Wine Quality, Automobile and Parkinson) and the 3 Use Cases (Protein Corona, Gajewicz Metal Oxides and Aquatic Toxicity) [file peerj-04-2721-s001.zip › 3_winequality/DifModels.RMSE.iSplits.4.pdf]

# Models' differences on the training set (data split 5)

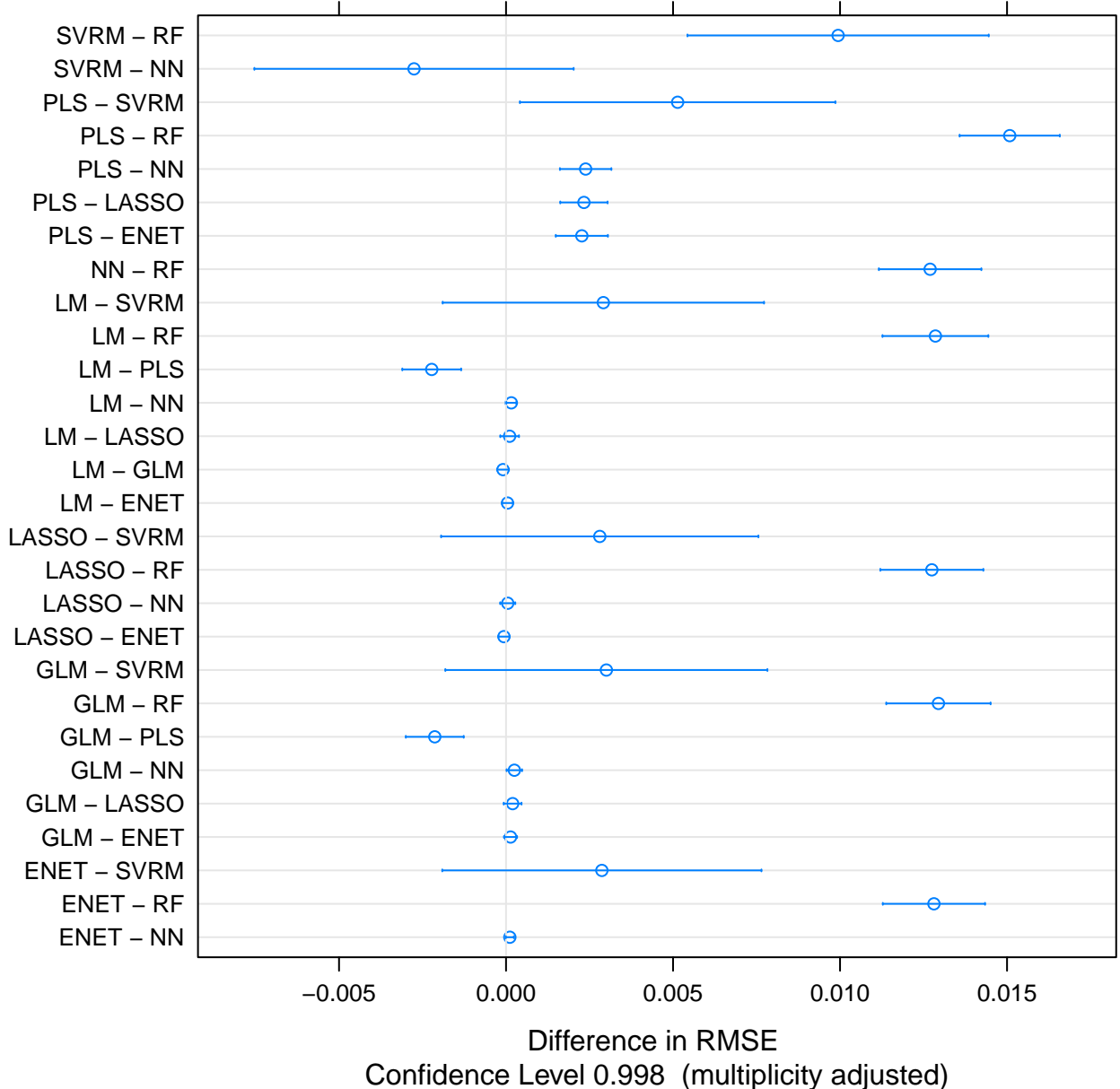

Supplement: Data S1 — Datailed results from UC Irvine Machine Learning Repository (Housing, Machine CPU, Wine Quality, Automobile and Parkinson) and the 3 Use Cases (Protein Corona, Gajewicz Metal Oxides and Aquatic Toxicity) [file peerj-04-2721-s001.zip › 3_winequality/DifModels.RMSE.iSplits.5.pdf]

# Models' differences on the training set (data split 6)

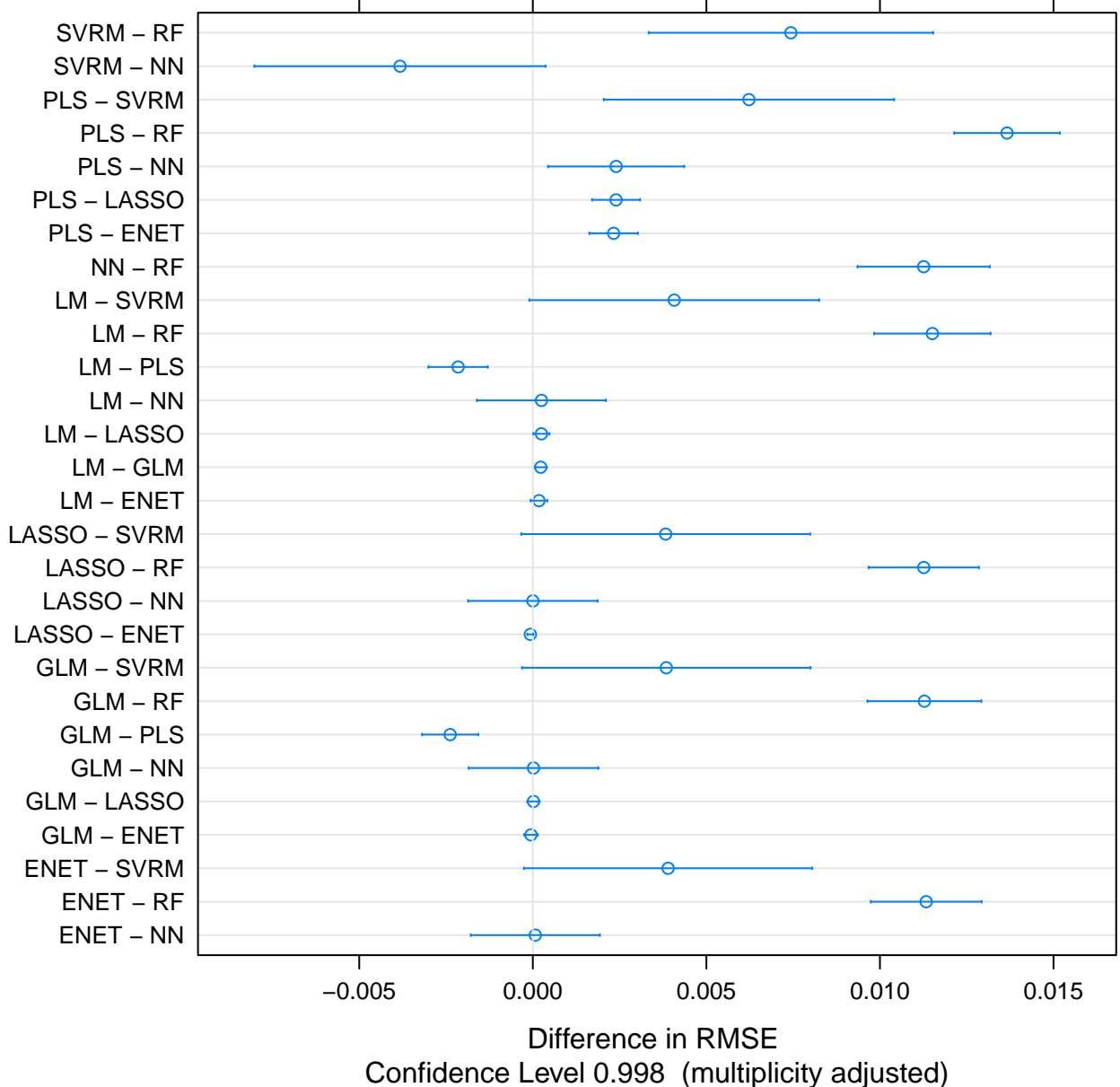

Supplement: Data S1 — Datailed results from UC Irvine Machine Learning Repository (Housing, Machine CPU, Wine Quality, Automobile and Parkinson) and the 3 Use Cases (Protein Corona, Gajewicz Metal Oxides and Aquatic Toxicity) [file peerj-04-2721-s001.zip › 3_winequality/DifModels.RMSE.iSplits.6.pdf]

# Models' differences on the training set (data split 7)

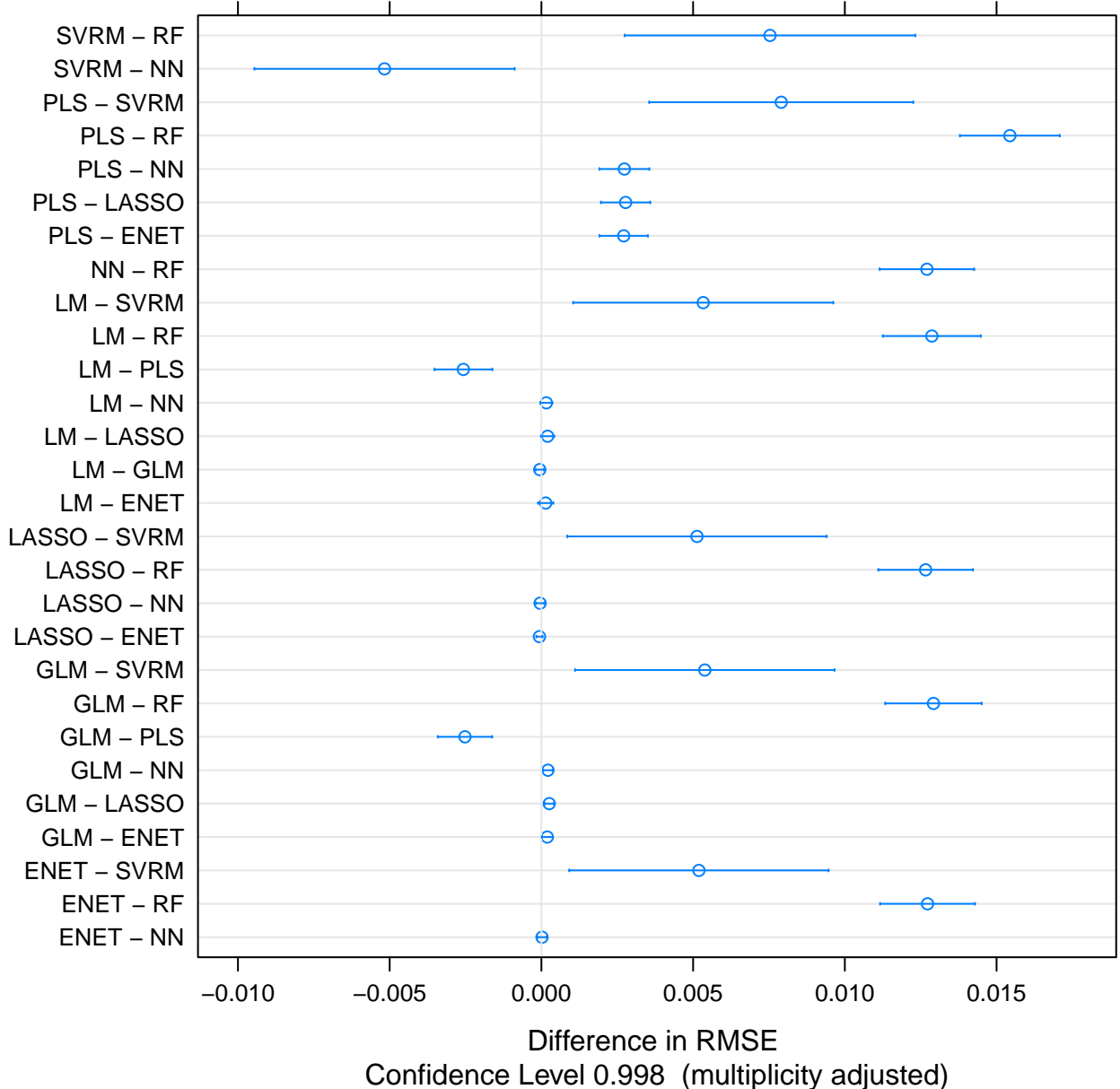

Supplement: Data S1 — Datailed results from UC Irvine Machine Learning Repository (Housing, Machine CPU, Wine Quality, Automobile and Parkinson) and the 3 Use Cases (Protein Corona, Gajewicz Metal Oxides and Aquatic Toxicity) [file peerj-04-2721-s001.zip › 3_winequality/DifModels.RMSE.iSplits.7.pdf]

# Models' differences on the training set (data split 8)

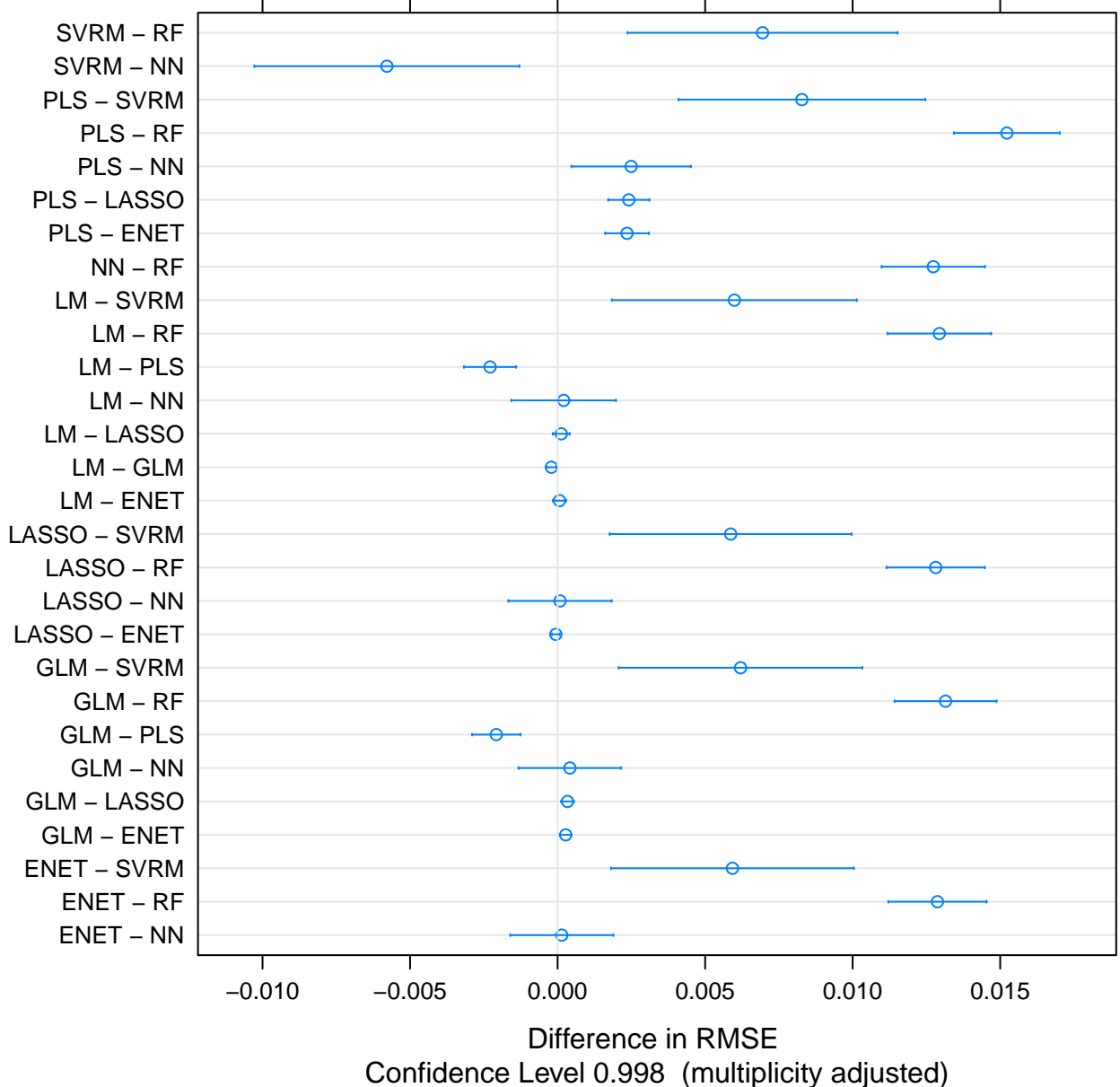

Supplement: Data S1 — Datailed results from UC Irvine Machine Learning Repository (Housing, Machine CPU, Wine Quality, Automobile and Parkinson) and the 3 Use Cases (Protein Corona, Gajewicz Metal Oxides and Aquatic Toxicity) [file peerj-04-2721-s001.zip › 3_winequality/DifModels.RMSE.iSplits.8.pdf]

# Models' differences on the training set (data split 9)

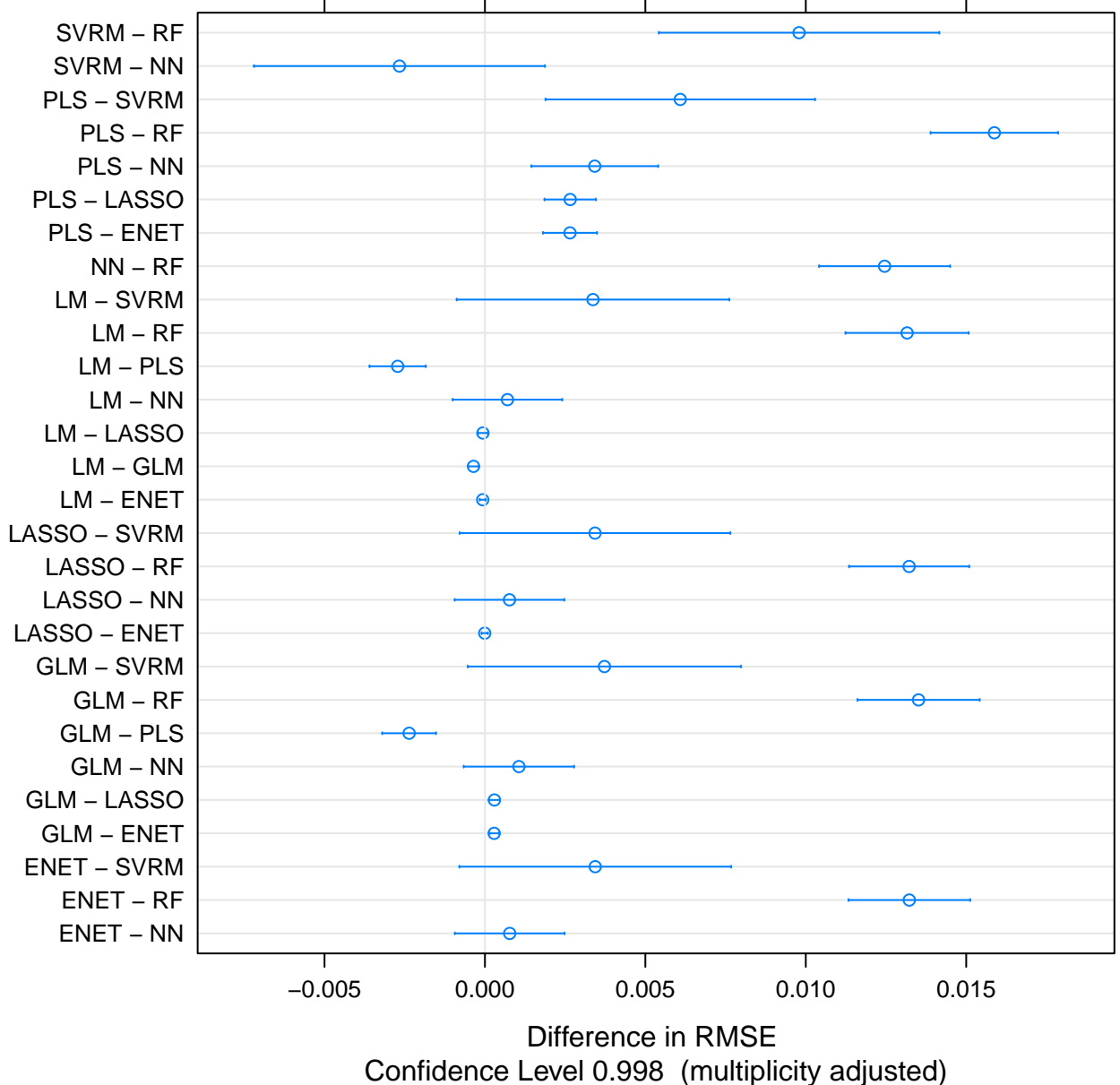

Supplement: Data S1 — Datailed results from UC Irvine Machine Learning Repository (Housing, Machine CPU, Wine Quality, Automobile and Parkinson) and the 3 Use Cases (Protein Corona, Gajewicz Metal Oxides and Aquatic Toxicity) [file peerj-04-2721-s001.zip › 3_winequality/DifModels.RMSE.iSplits.9.pdf]

# Resampling results on the training set (data split 1)

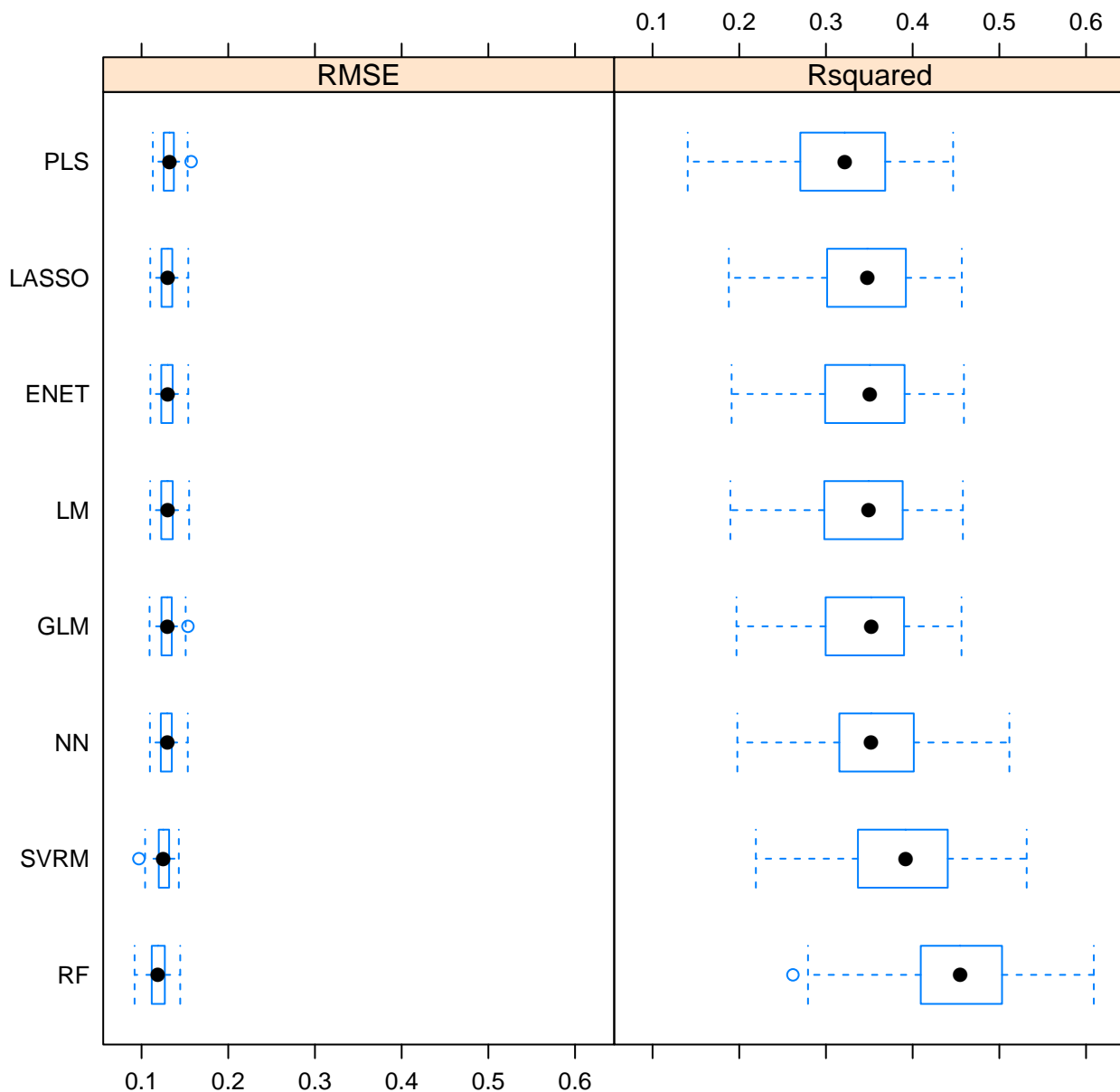

Supplement: Data S1 — Datailed results from UC Irvine Machine Learning Repository (Housing, Machine CPU, Wine Quality, Automobile and Parkinson) and the 3 Use Cases (Protein Corona, Gajewicz Metal Oxides and Aquatic Toxicity) [file peerj-04-2721-s001.zip › 3_winequality/ModelsComp.iSplits.1.pdf]

# Resampling results on the training set (data split 10)

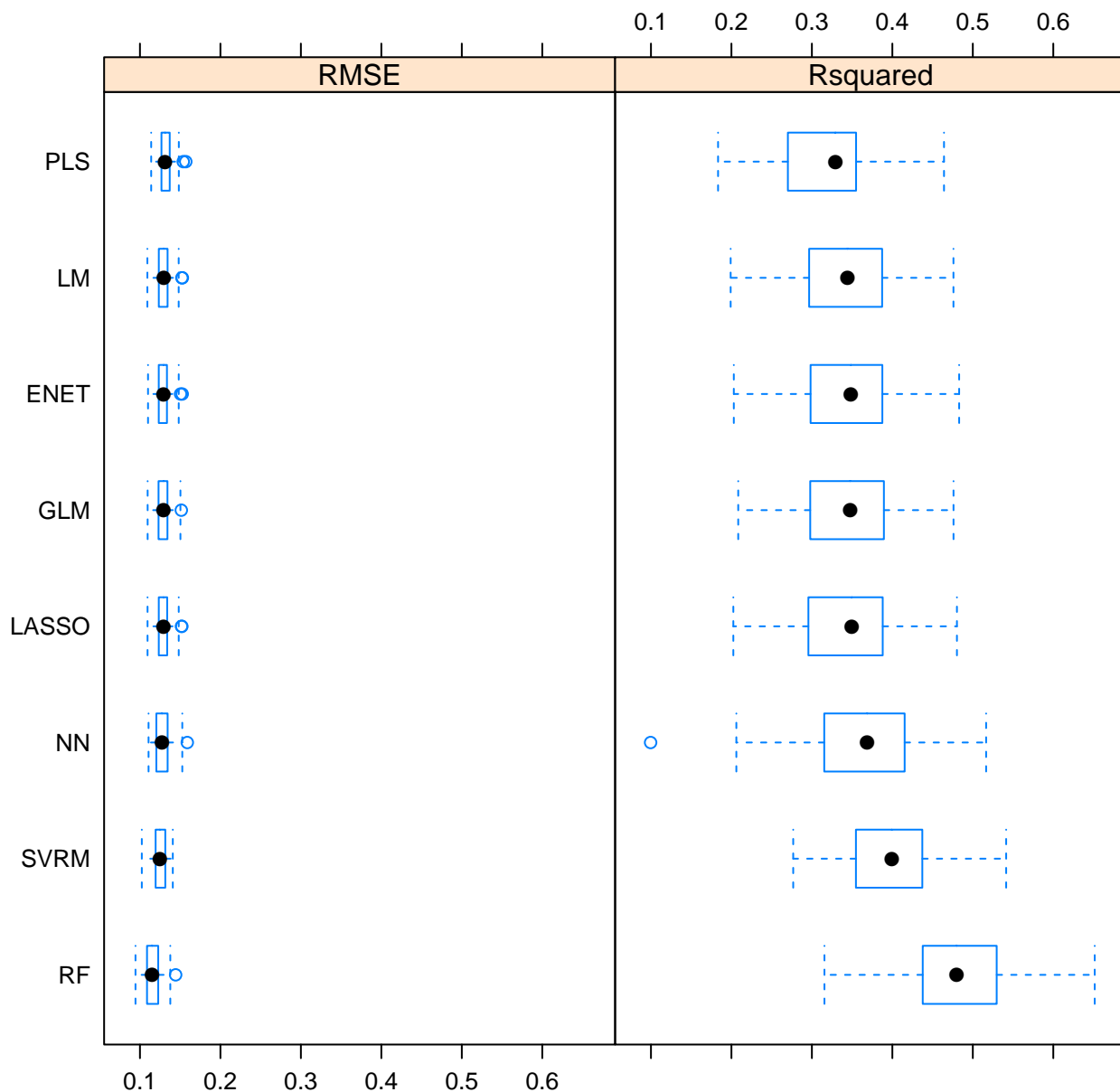

Supplement: Data S1 — Datailed results from UC Irvine Machine Learning Repository (Housing, Machine CPU, Wine Quality, Automobile and Parkinson) and the 3 Use Cases (Protein Corona, Gajewicz Metal Oxides and Aquatic Toxicity) [file peerj-04-2721-s001.zip › 3_winequality/ModelsComp.iSplits.10.pdf]

# Resampling results on the training set (data split 2)

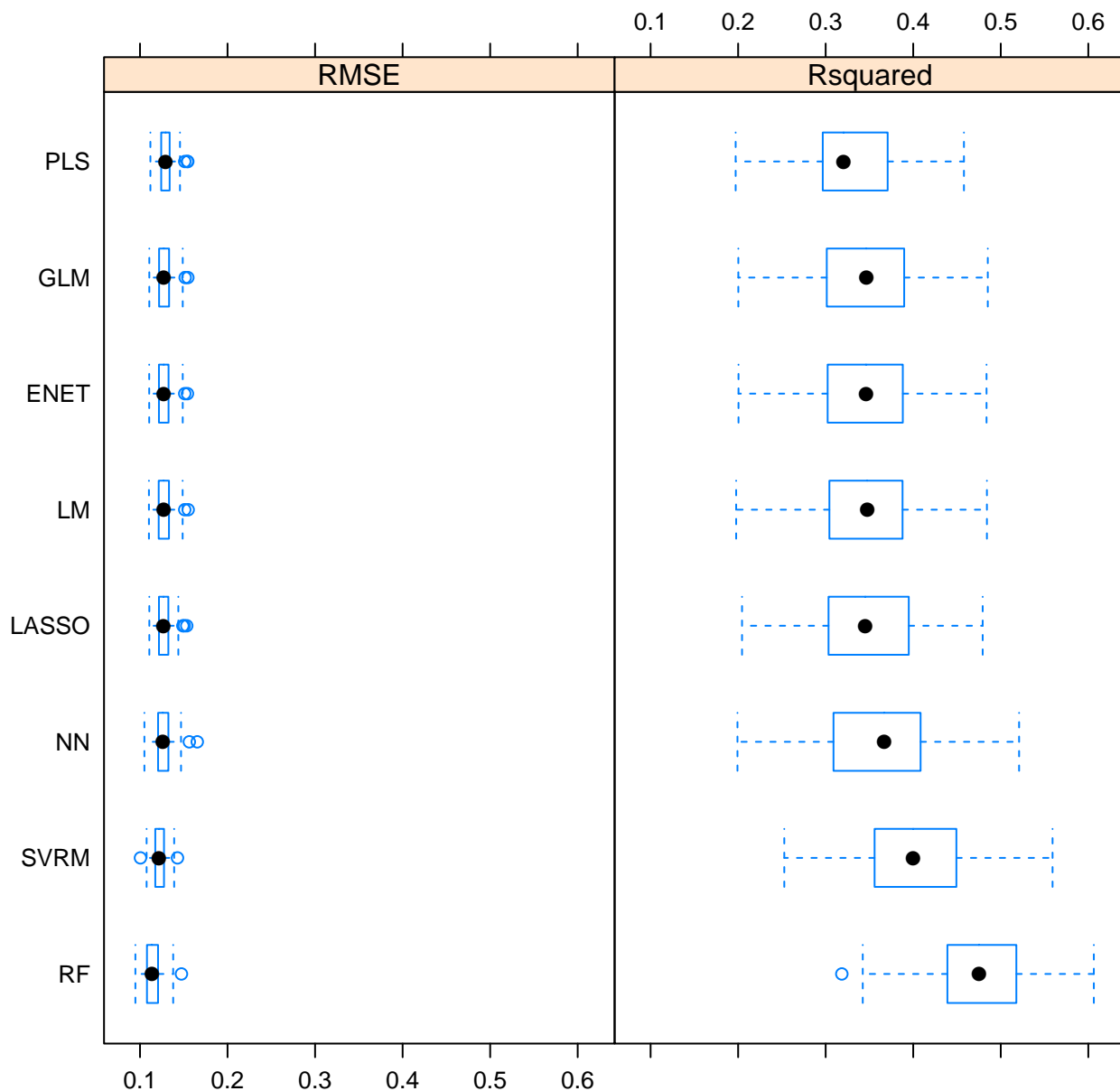

Supplement: Data S1 — Datailed results from UC Irvine Machine Learning Repository (Housing, Machine CPU, Wine Quality, Automobile and Parkinson) and the 3 Use Cases (Protein Corona, Gajewicz Metal Oxides and Aquatic Toxicity) [file peerj-04-2721-s001.zip › 3_winequality/ModelsComp.iSplits.2.pdf]

# Resampling results on the training set (data split 3)

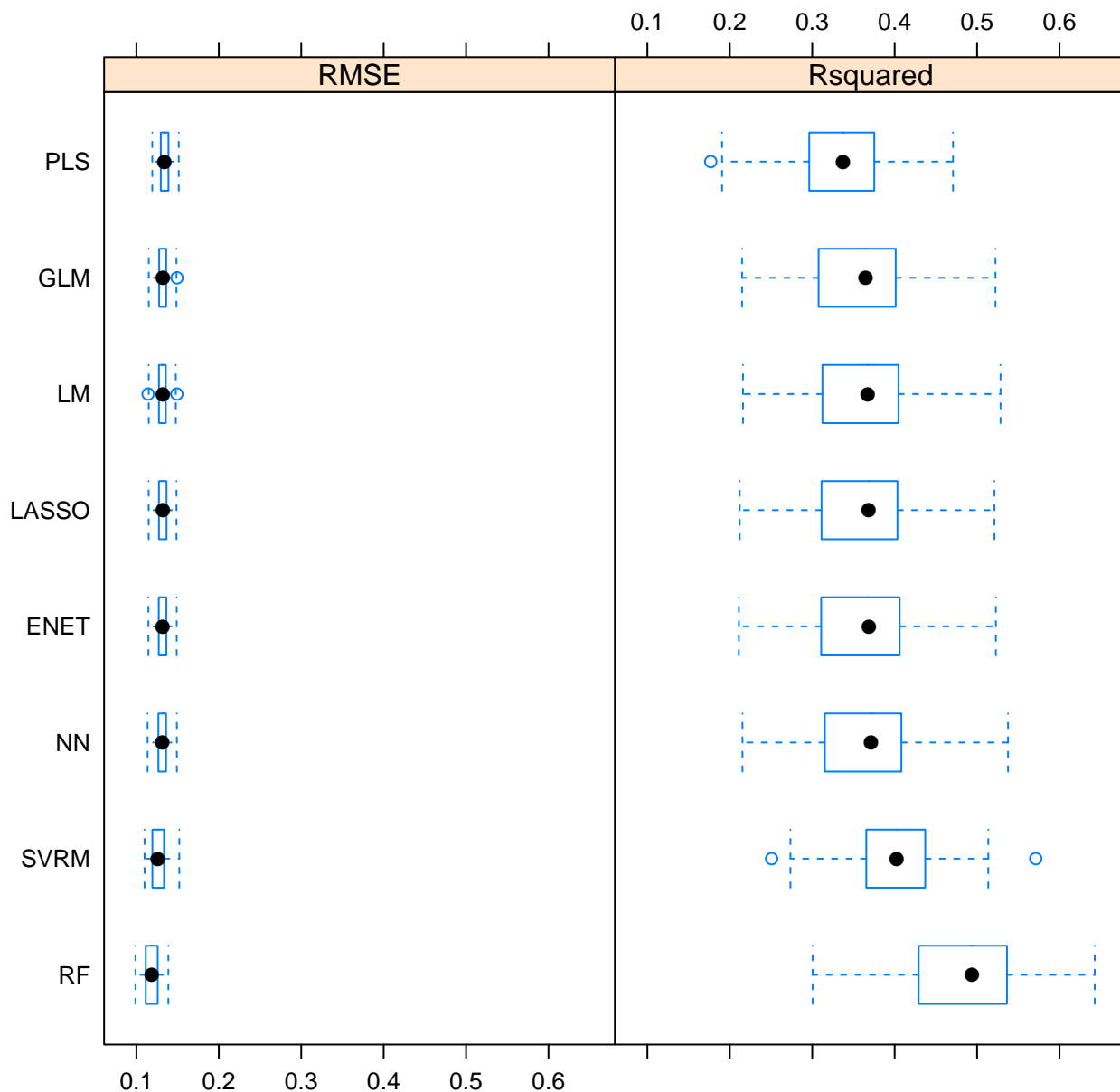

Supplement: Data S1 — Datailed results from UC Irvine Machine Learning Repository (Housing, Machine CPU, Wine Quality, Automobile and Parkinson) and the 3 Use Cases (Protein Corona, Gajewicz Metal Oxides and Aquatic Toxicity) [file peerj-04-2721-s001.zip › 3_winequality/ModelsComp.iSplits.3.pdf]

# Resampling results on the training set (data split 4)

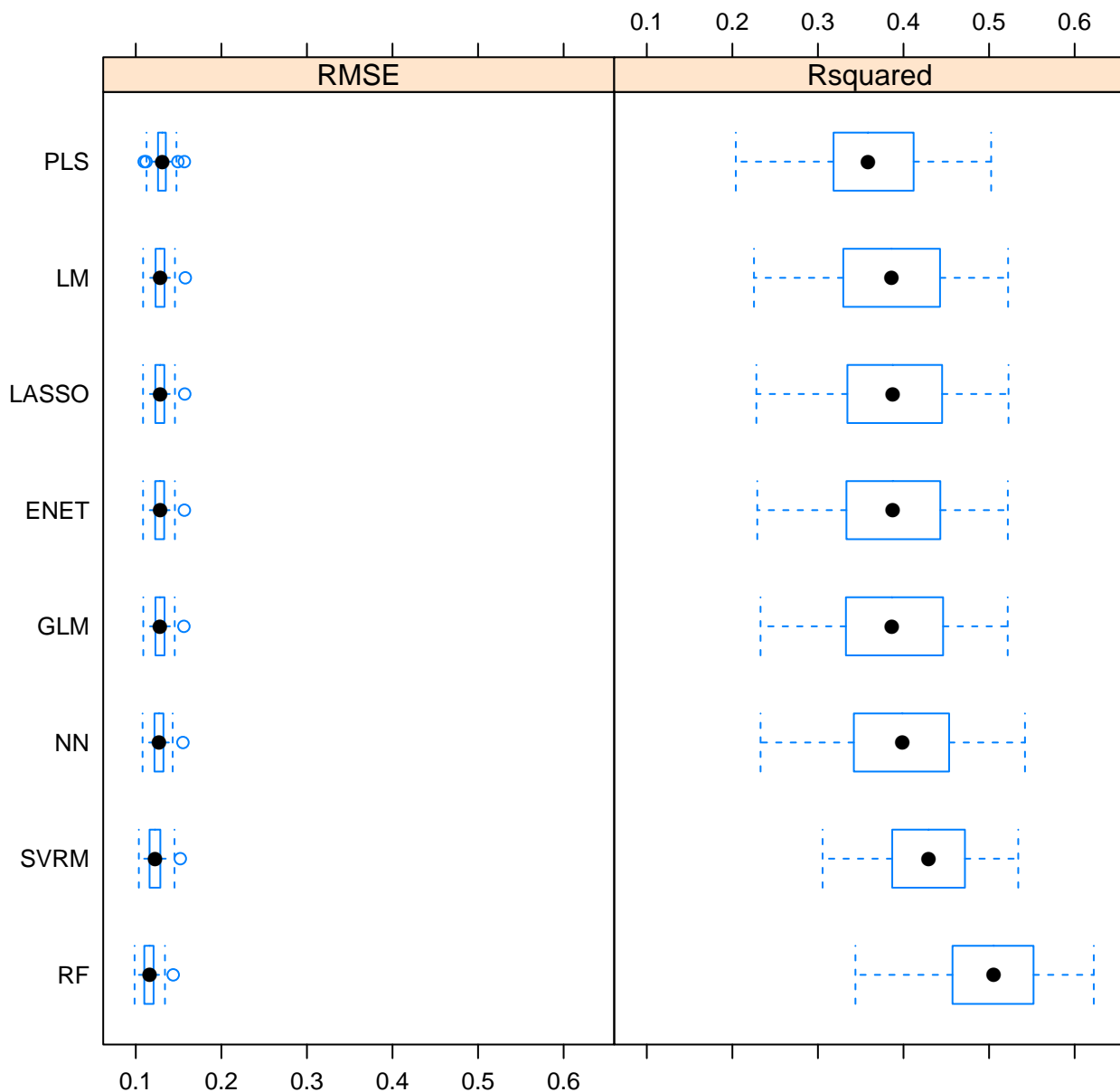

Supplement: Data S1 — Datailed results from UC Irvine Machine Learning Repository (Housing, Machine CPU, Wine Quality, Automobile and Parkinson) and the 3 Use Cases (Protein Corona, Gajewicz Metal Oxides and Aquatic Toxicity) [file peerj-04-2721-s001.zip › 3_winequality/ModelsComp.iSplits.4.pdf]

# Resampling results on the training set (data split 5)

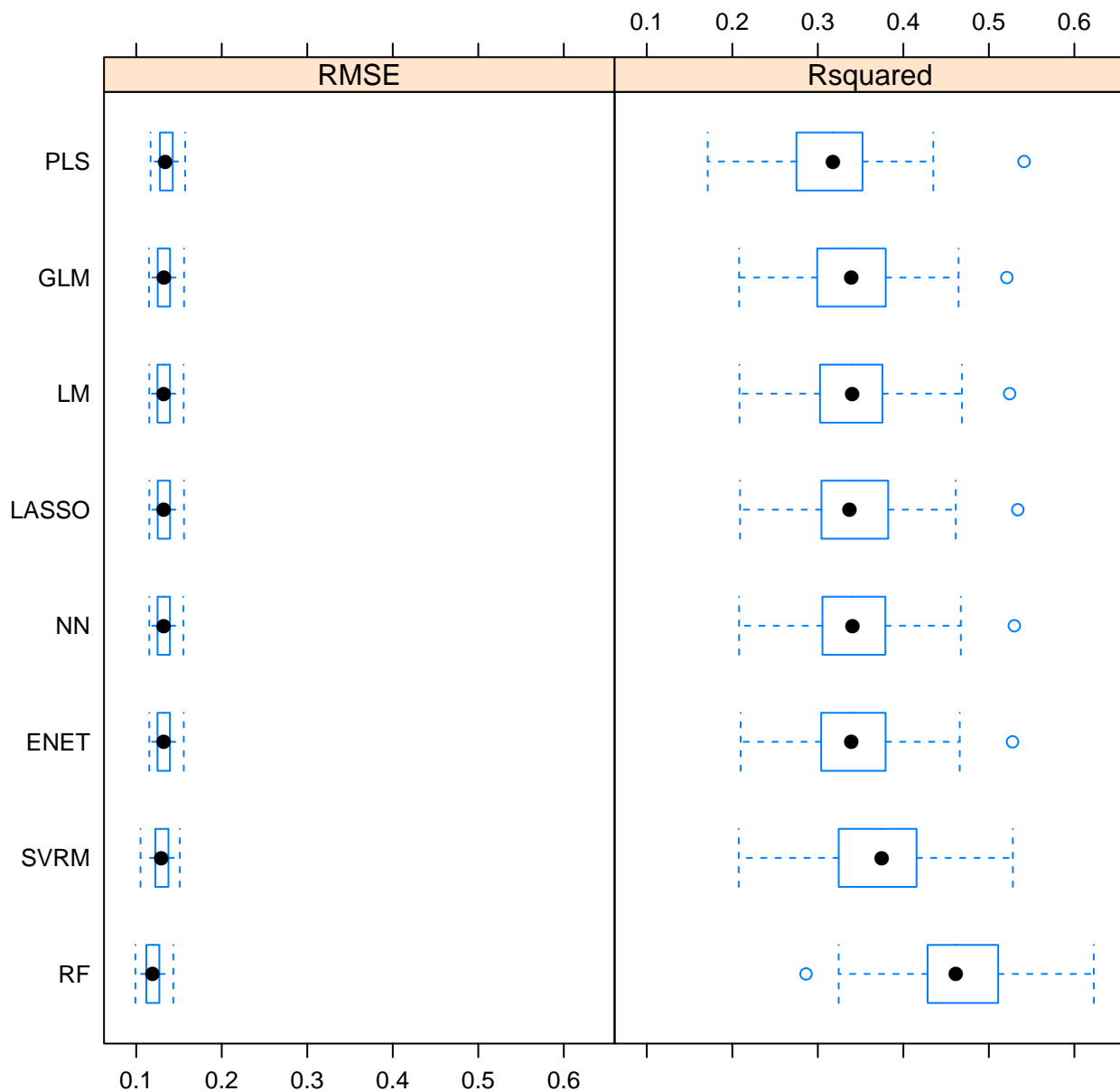

Supplement: Data S1 — Datailed results from UC Irvine Machine Learning Repository (Housing, Machine CPU, Wine Quality, Automobile and Parkinson) and the 3 Use Cases (Protein Corona, Gajewicz Metal Oxides and Aquatic Toxicity) [file peerj-04-2721-s001.zip › 3_winequality/ModelsComp.iSplits.5.pdf]

# Resampling results on the training set (data split 6)

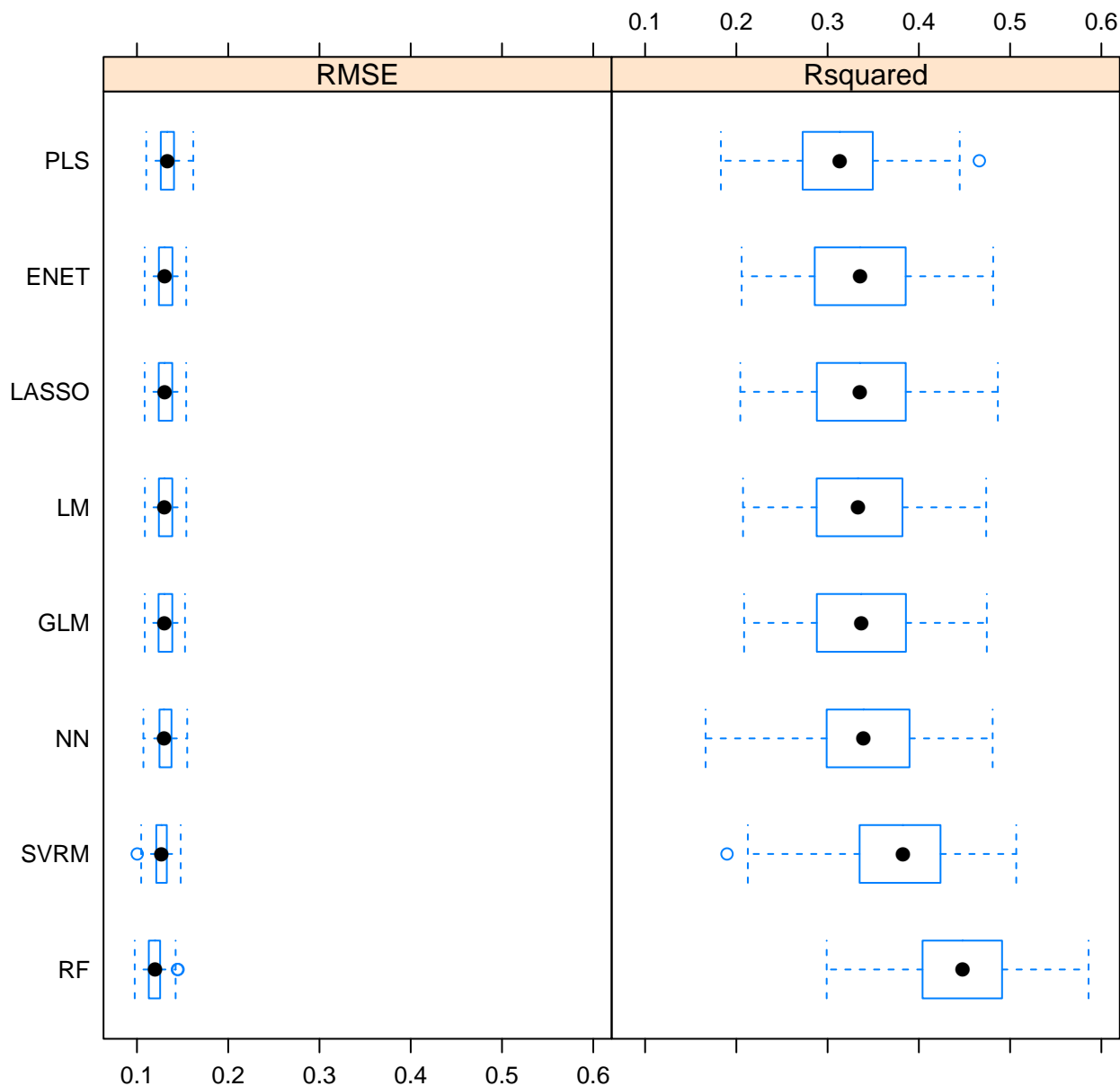

Supplement: Data S1 — Datailed results from UC Irvine Machine Learning Repository (Housing, Machine CPU, Wine Quality, Automobile and Parkinson) and the 3 Use Cases (Protein Corona, Gajewicz Metal Oxides and Aquatic Toxicity) [file peerj-04-2721-s001.zip › 3_winequality/ModelsComp.iSplits.6.pdf]

# Resampling results on the training set (data split 7)

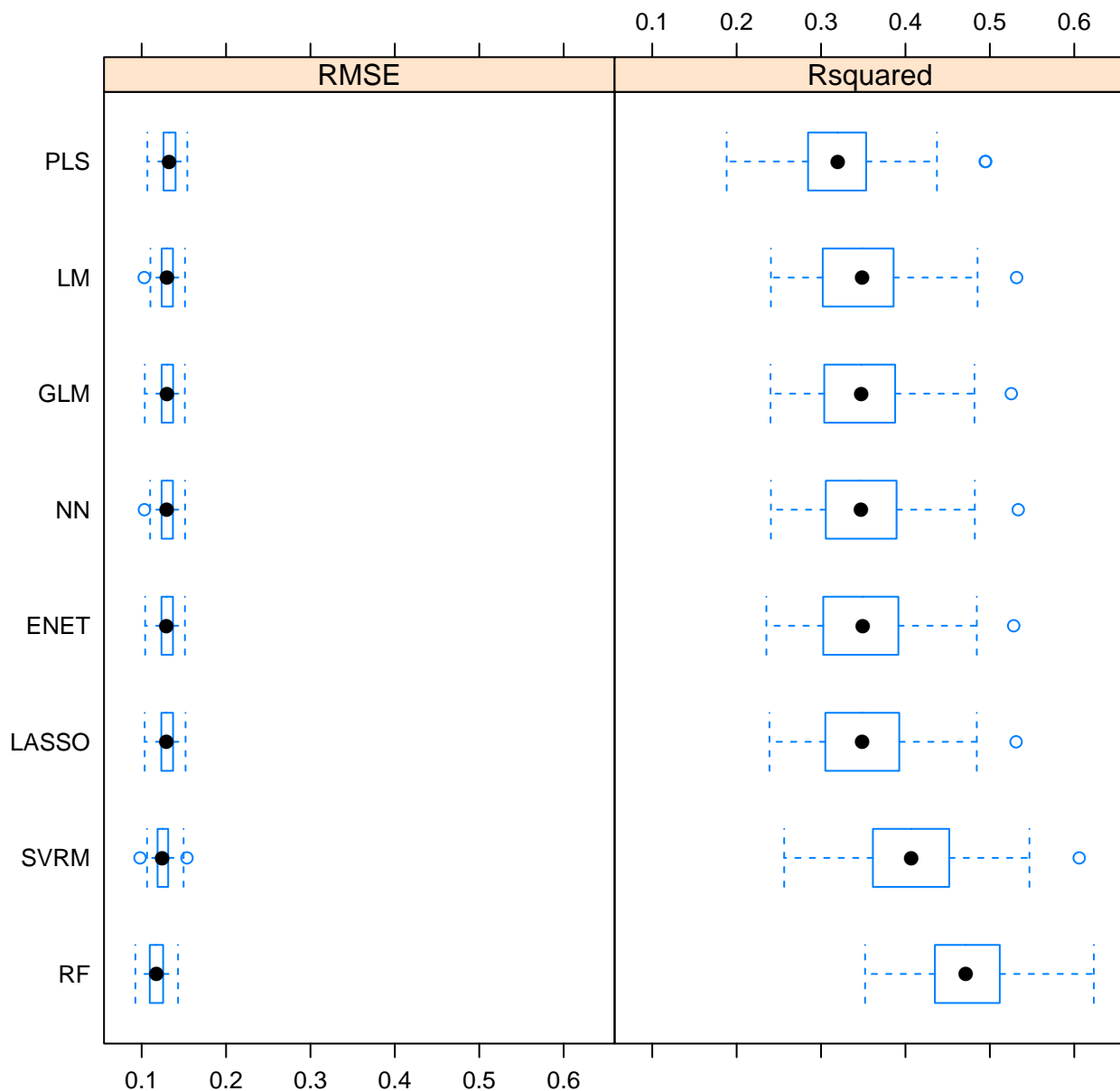

Supplement: Data S1 — Datailed results from UC Irvine Machine Learning Repository (Housing, Machine CPU, Wine Quality, Automobile and Parkinson) and the 3 Use Cases (Protein Corona, Gajewicz Metal Oxides and Aquatic Toxicity) [file peerj-04-2721-s001.zip › 3_winequality/ModelsComp.iSplits.7.pdf]

# Resampling results on the training set (data split 8)

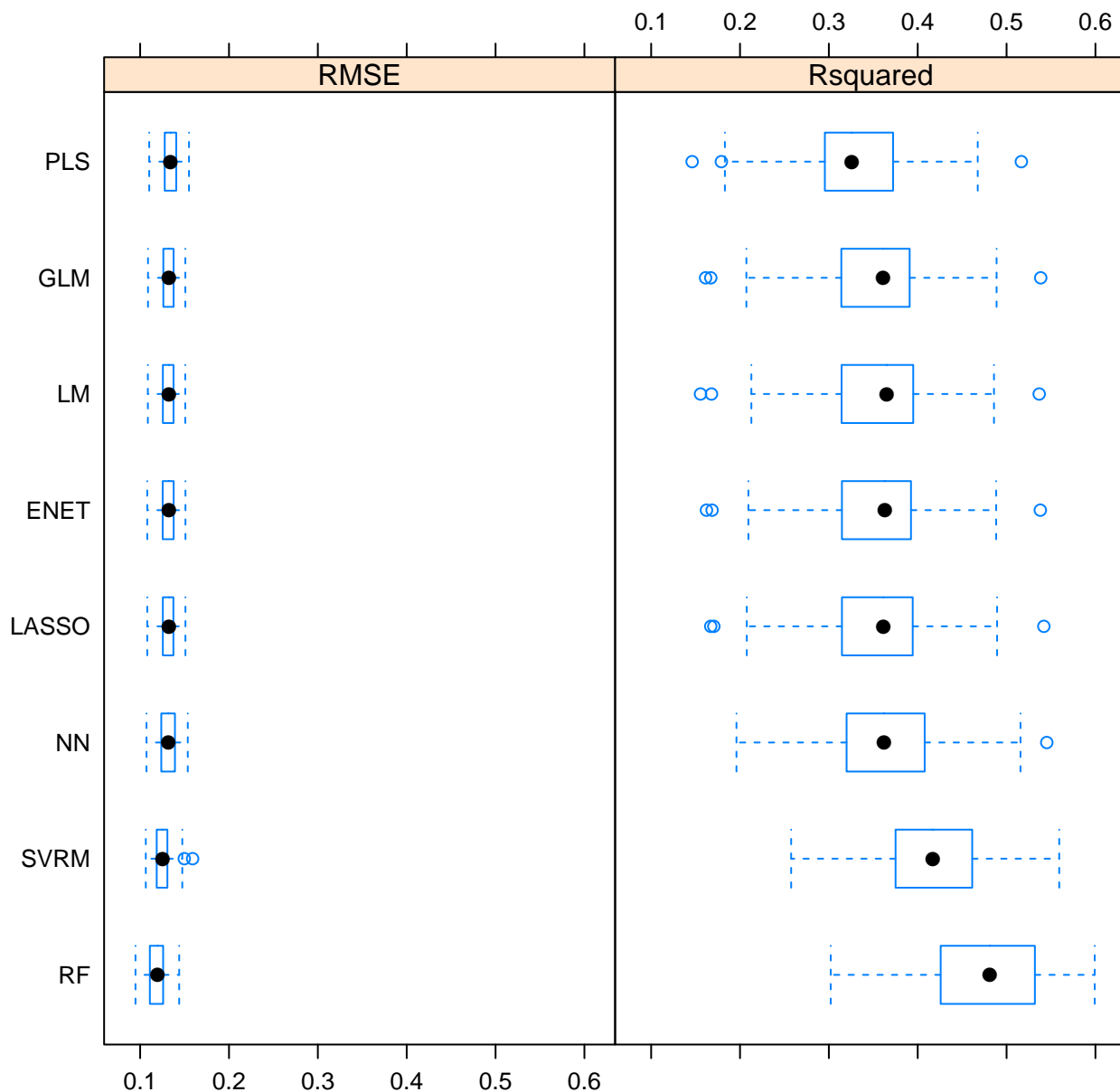

Supplement: Data S1 — Datailed results from UC Irvine Machine Learning Repository (Housing, Machine CPU, Wine Quality, Automobile and Parkinson) and the 3 Use Cases (Protein Corona, Gajewicz Metal Oxides and Aquatic Toxicity) [file peerj-04-2721-s001.zip › 3_winequality/ModelsComp.iSplits.8.pdf]

# Resampling results on the training set (data split 9)

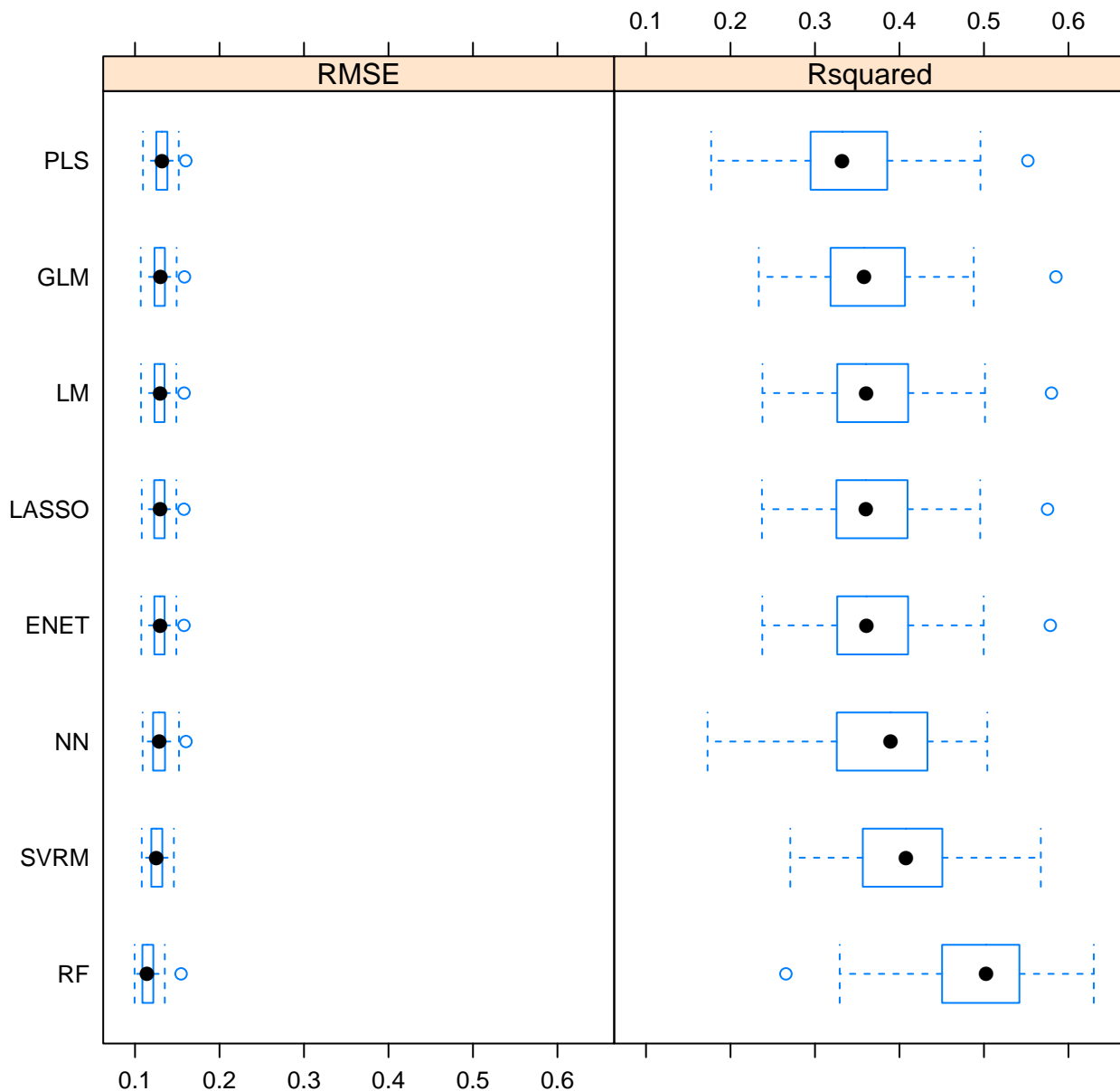

Supplement: Data S1 — Datailed results from UC Irvine Machine Learning Repository (Housing, Machine CPU, Wine Quality, Automobile and Parkinson) and the 3 Use Cases (Protein Corona, Gajewicz Metal Oxides and Aquatic Toxicity) [file peerj-04-2721-s001.zip › 3_winequality/ModelsComp.iSplits.9.pdf]

# Models' differences on the training set (data split 1)

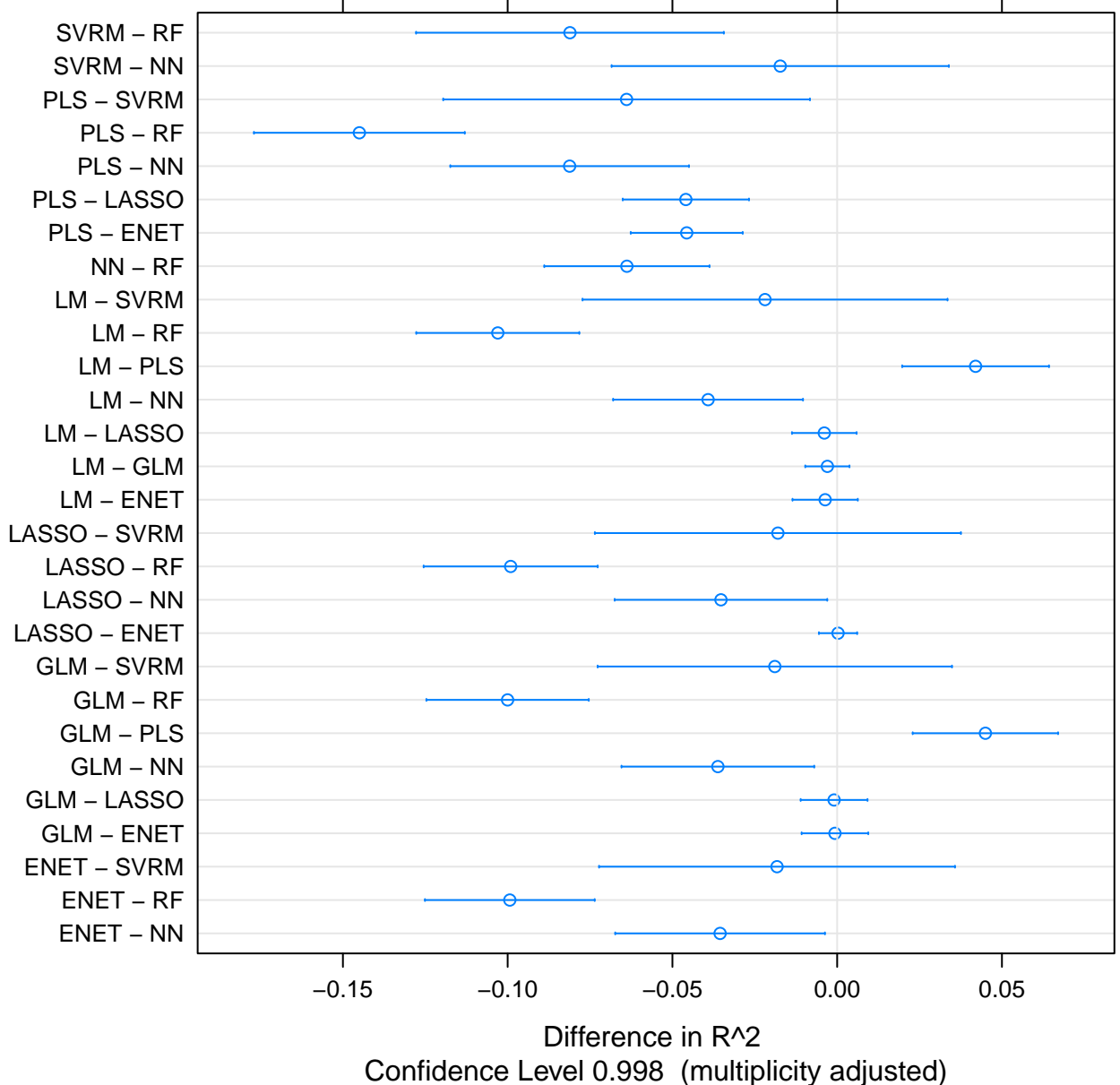

Supplement: Data S1 — Datailed results from UC Irvine Machine Learning Repository (Housing, Machine CPU, Wine Quality, Automobile and Parkinson) and the 3 Use Cases (Protein Corona, Gajewicz Metal Oxides and Aquatic Toxicity) [file peerj-04-2721-s001.zip › 4_automobile/DifModels.R2.iSplits.1.pdf]

# Models' differences on the training set (data split 10)

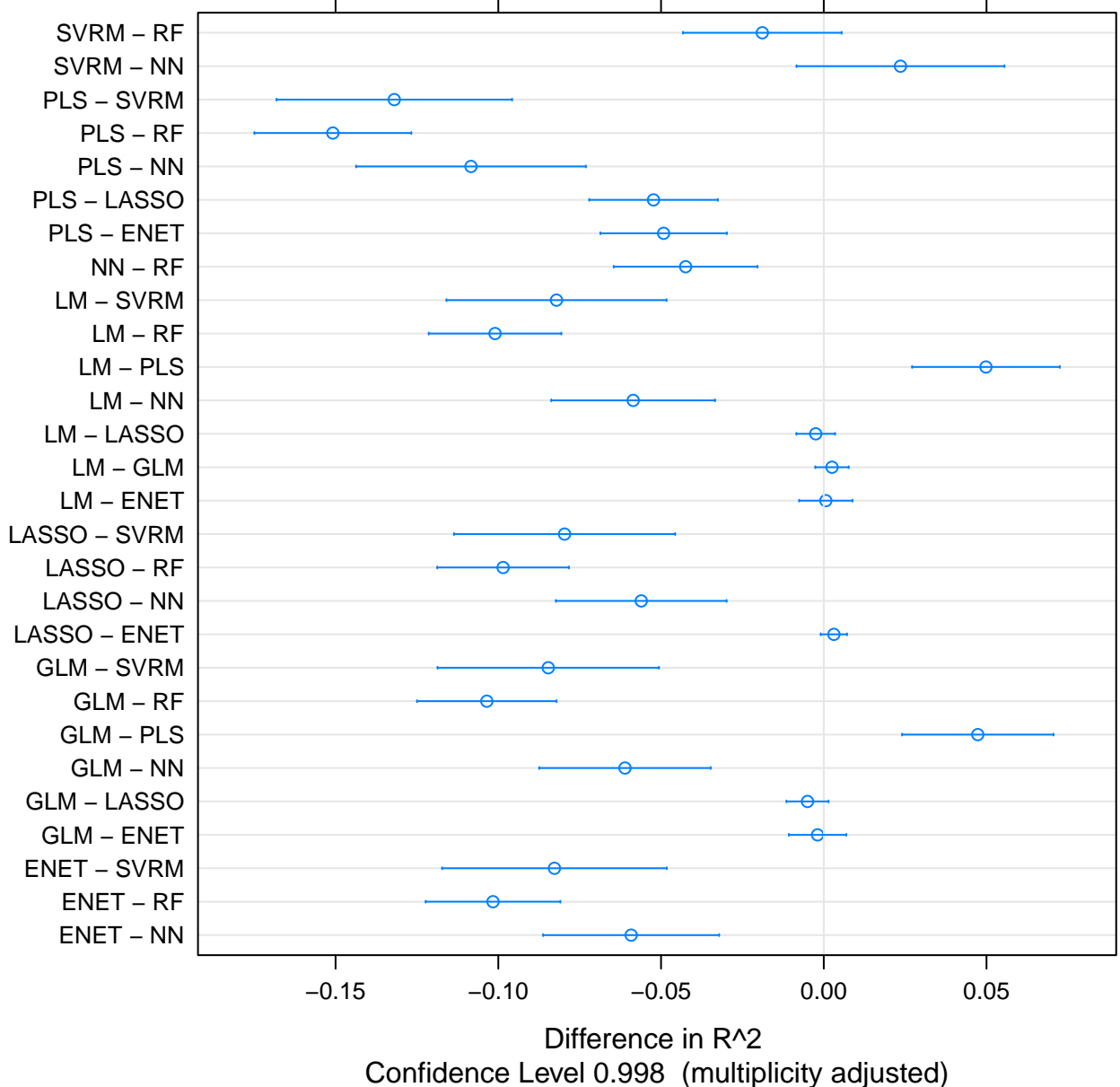

Supplement: Data S1 — Datailed results from UC Irvine Machine Learning Repository (Housing, Machine CPU, Wine Quality, Automobile and Parkinson) and the 3 Use Cases (Protein Corona, Gajewicz Metal Oxides and Aquatic Toxicity) [file peerj-04-2721-s001.zip › 4_automobile/DifModels.R2.iSplits.10.pdf]

# Models' differences on the training set (data split 2)

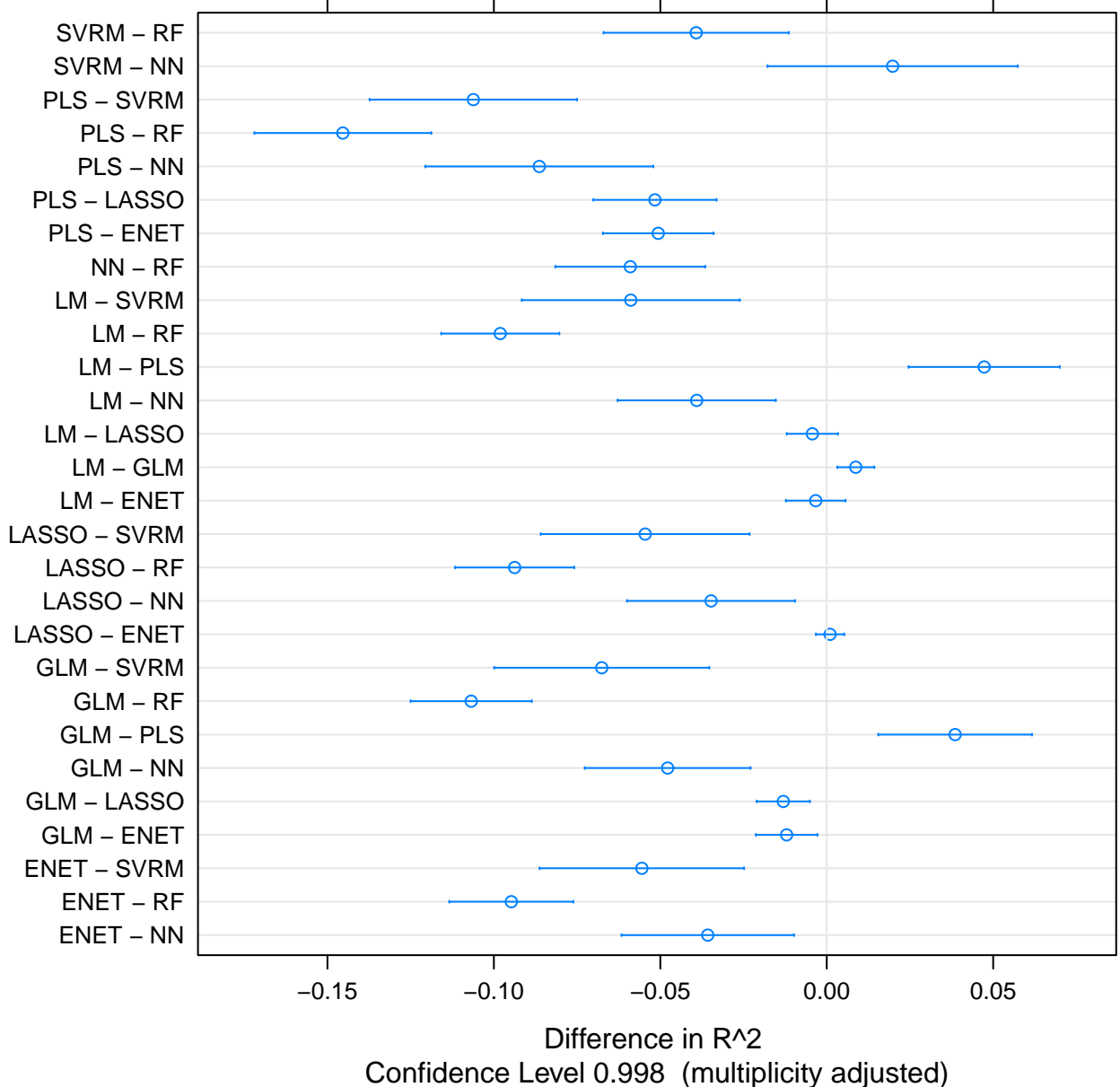

Supplement: Data S1 — Datailed results from UC Irvine Machine Learning Repository (Housing, Machine CPU, Wine Quality, Automobile and Parkinson) and the 3 Use Cases (Protein Corona, Gajewicz Metal Oxides and Aquatic Toxicity) [file peerj-04-2721-s001.zip › 4_automobile/DifModels.R2.iSplits.2.pdf]

# Models' differences on the training set (data split 3)

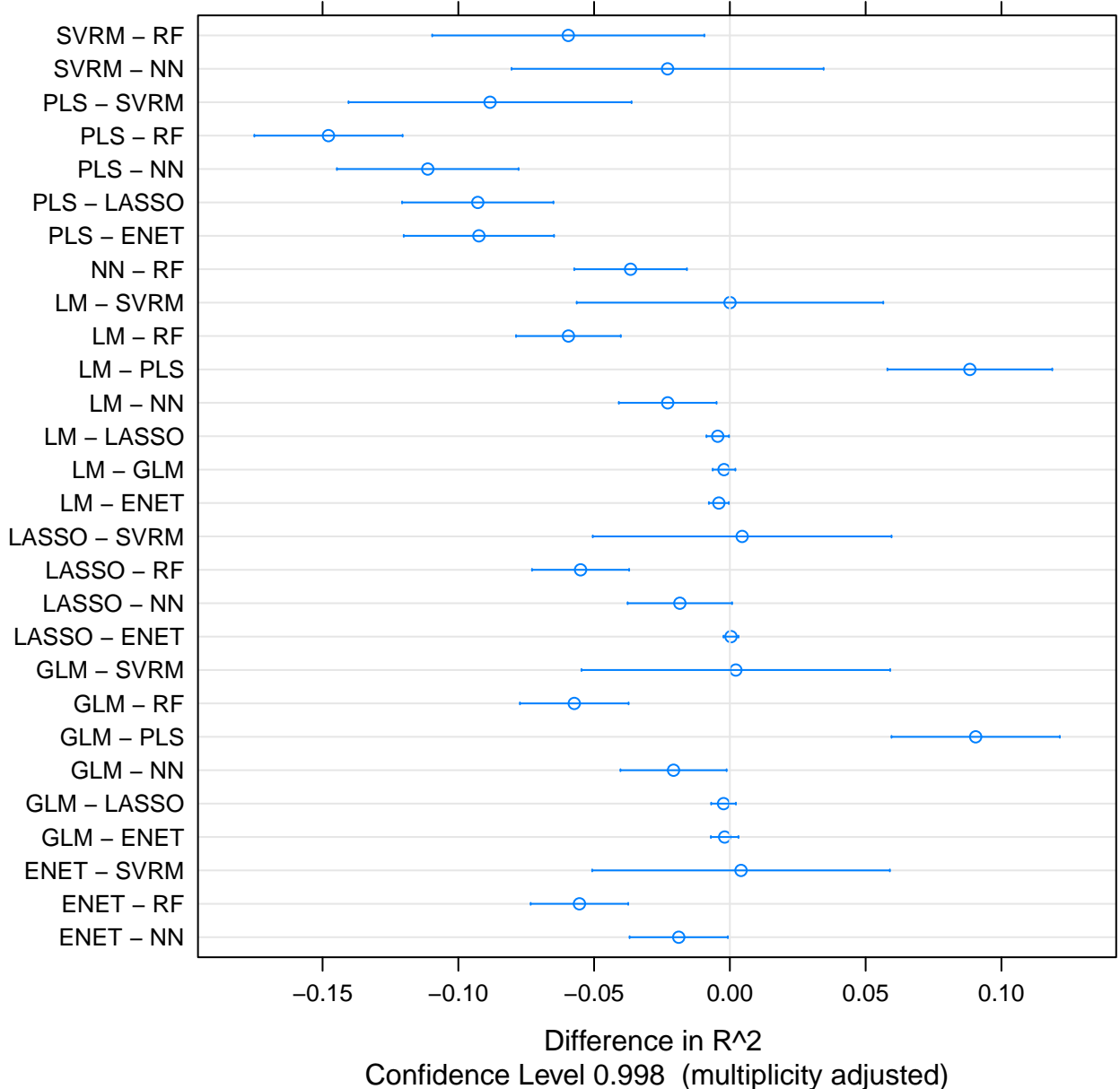

Supplement: Data S1 — Datailed results from UC Irvine Machine Learning Repository (Housing, Machine CPU, Wine Quality, Automobile and Parkinson) and the 3 Use Cases (Protein Corona, Gajewicz Metal Oxides and Aquatic Toxicity) [file peerj-04-2721-s001.zip › 4_automobile/DifModels.R2.iSplits.3.pdf]

# Models' differences on the training set (data split 4)

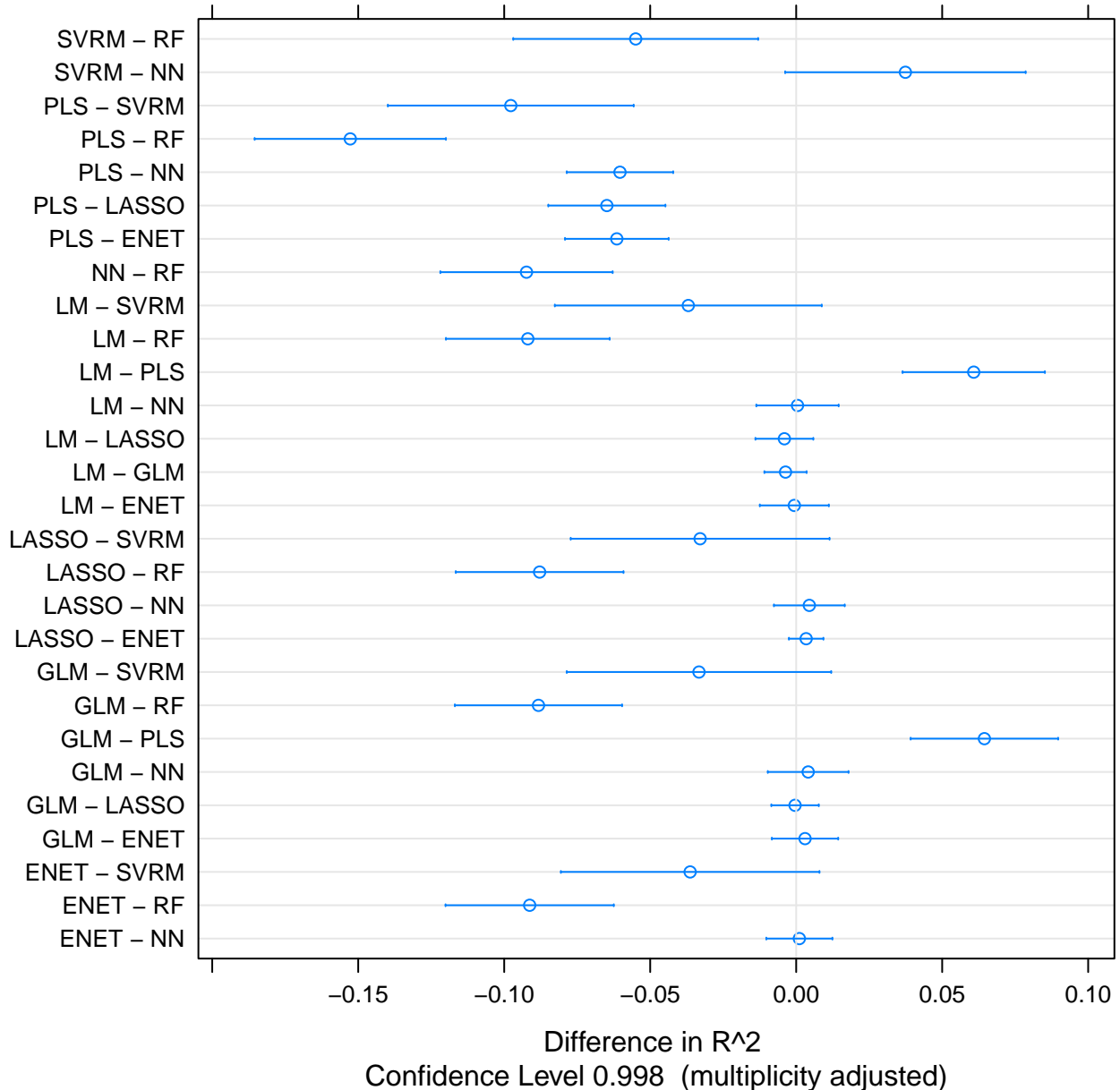

Supplement: Data S1 — Datailed results from UC Irvine Machine Learning Repository (Housing, Machine CPU, Wine Quality, Automobile and Parkinson) and the 3 Use Cases (Protein Corona, Gajewicz Metal Oxides and Aquatic Toxicity) [file peerj-04-2721-s001.zip › 4_automobile/DifModels.R2.iSplits.4.pdf]

# Models' differences on the training set (data split 5)

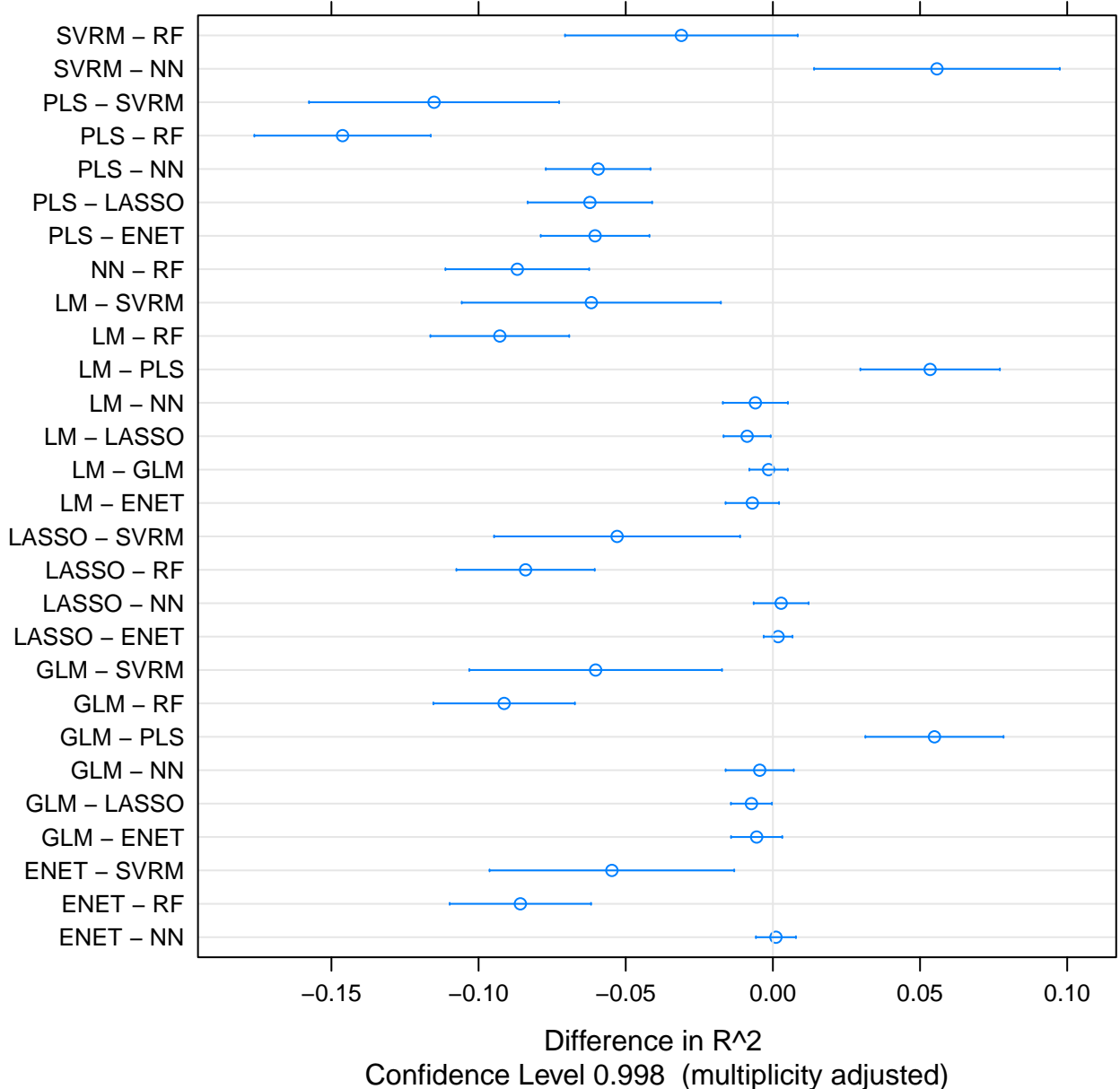

Supplement: Data S1 — Datailed results from UC Irvine Machine Learning Repository (Housing, Machine CPU, Wine Quality, Automobile and Parkinson) and the 3 Use Cases (Protein Corona, Gajewicz Metal Oxides and Aquatic Toxicity) [file peerj-04-2721-s001.zip › 4_automobile/DifModels.R2.iSplits.5.pdf]

# Models' differences on the training set (data split 6)

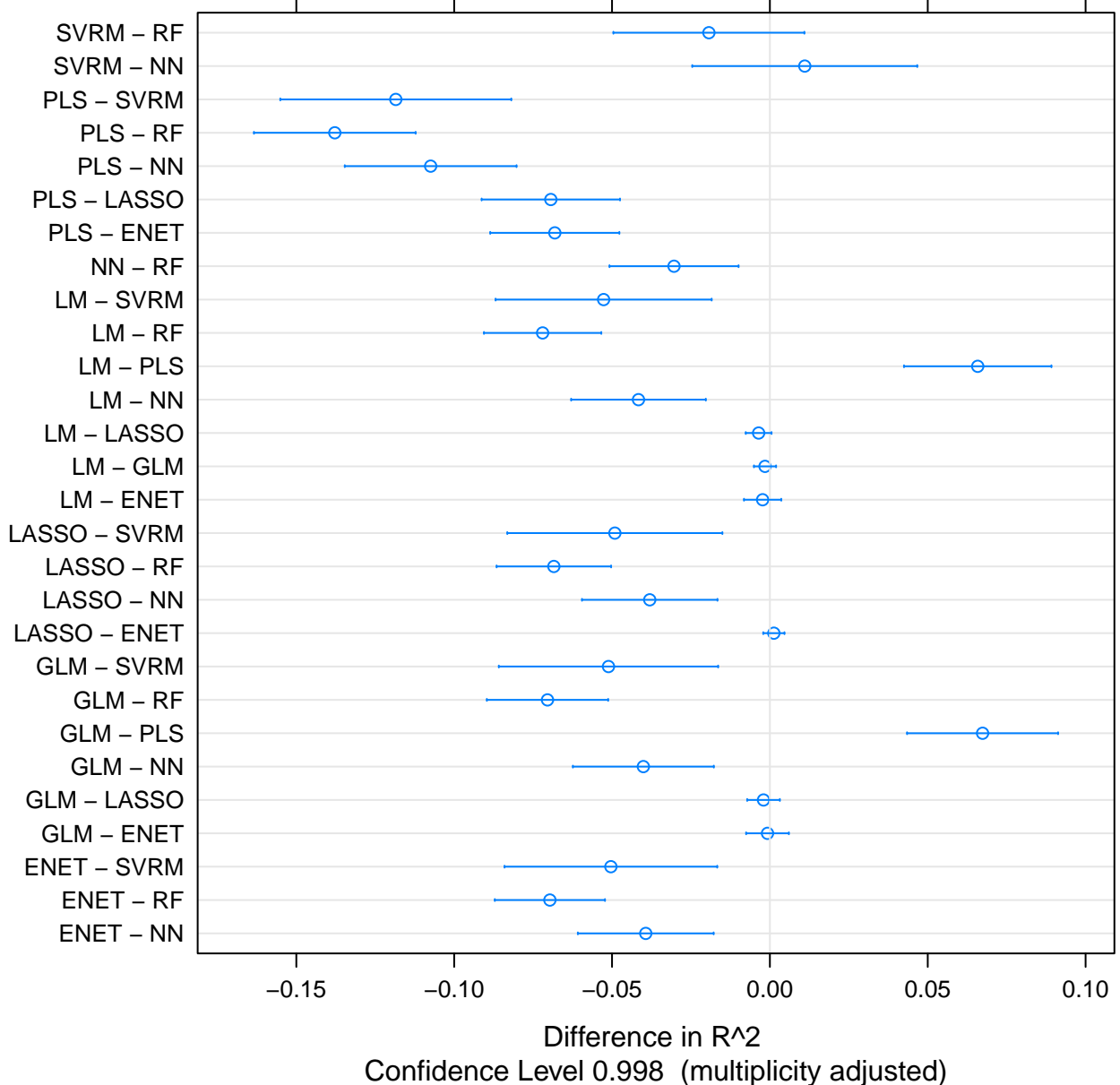

Supplement: Data S1 — Datailed results from UC Irvine Machine Learning Repository (Housing, Machine CPU, Wine Quality, Automobile and Parkinson) and the 3 Use Cases (Protein Corona, Gajewicz Metal Oxides and Aquatic Toxicity) [file peerj-04-2721-s001.zip › 4_automobile/DifModels.R2.iSplits.6.pdf]

# Models' differences on the training set (data split 7)

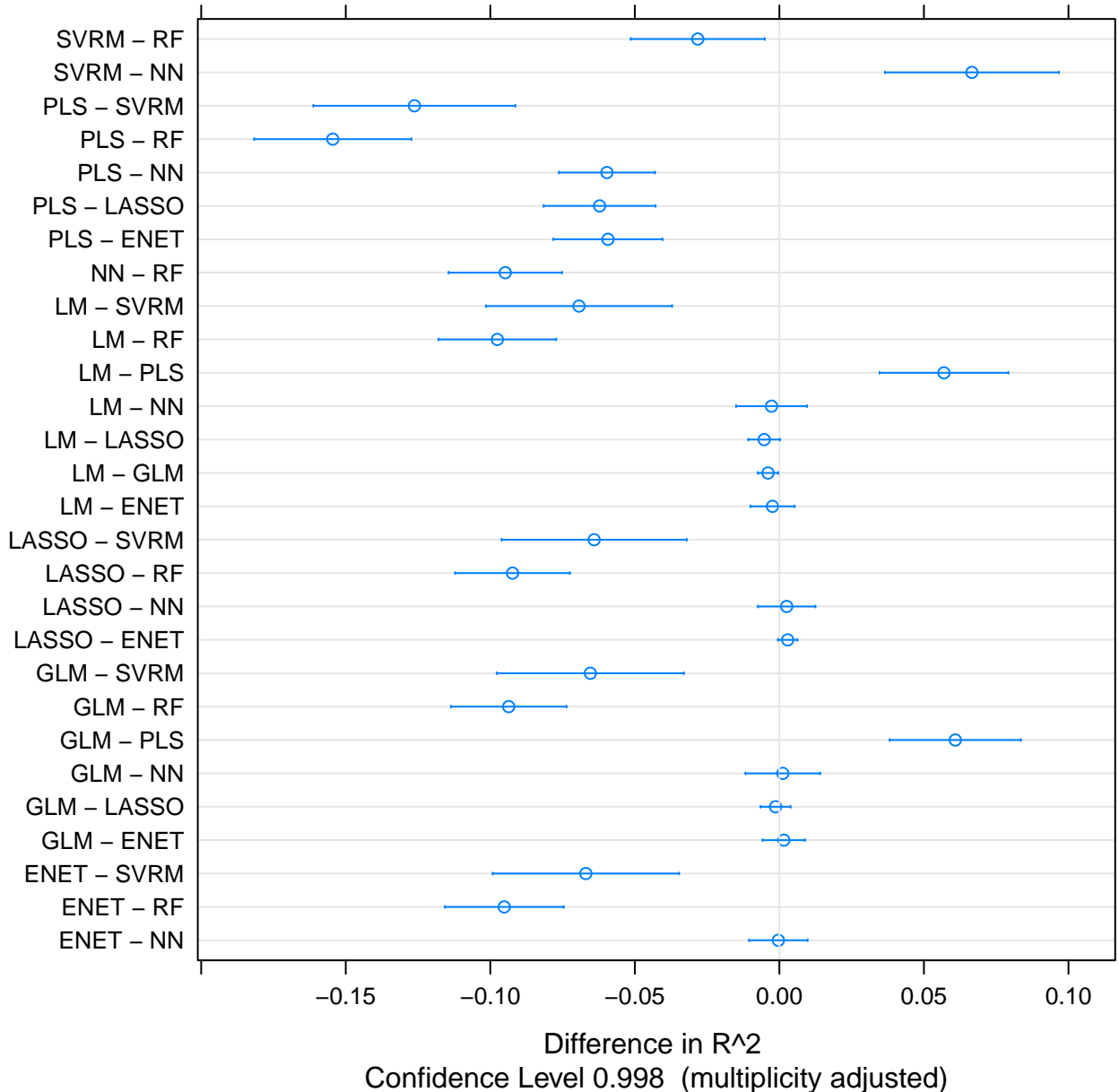

Supplement: Data S1 — Datailed results from UC Irvine Machine Learning Repository (Housing, Machine CPU, Wine Quality, Automobile and Parkinson) and the 3 Use Cases (Protein Corona, Gajewicz Metal Oxides and Aquatic Toxicity) [file peerj-04-2721-s001.zip › 4_automobile/DifModels.R2.iSplits.7.pdf]

# Models' differences on the training set (data split 8)

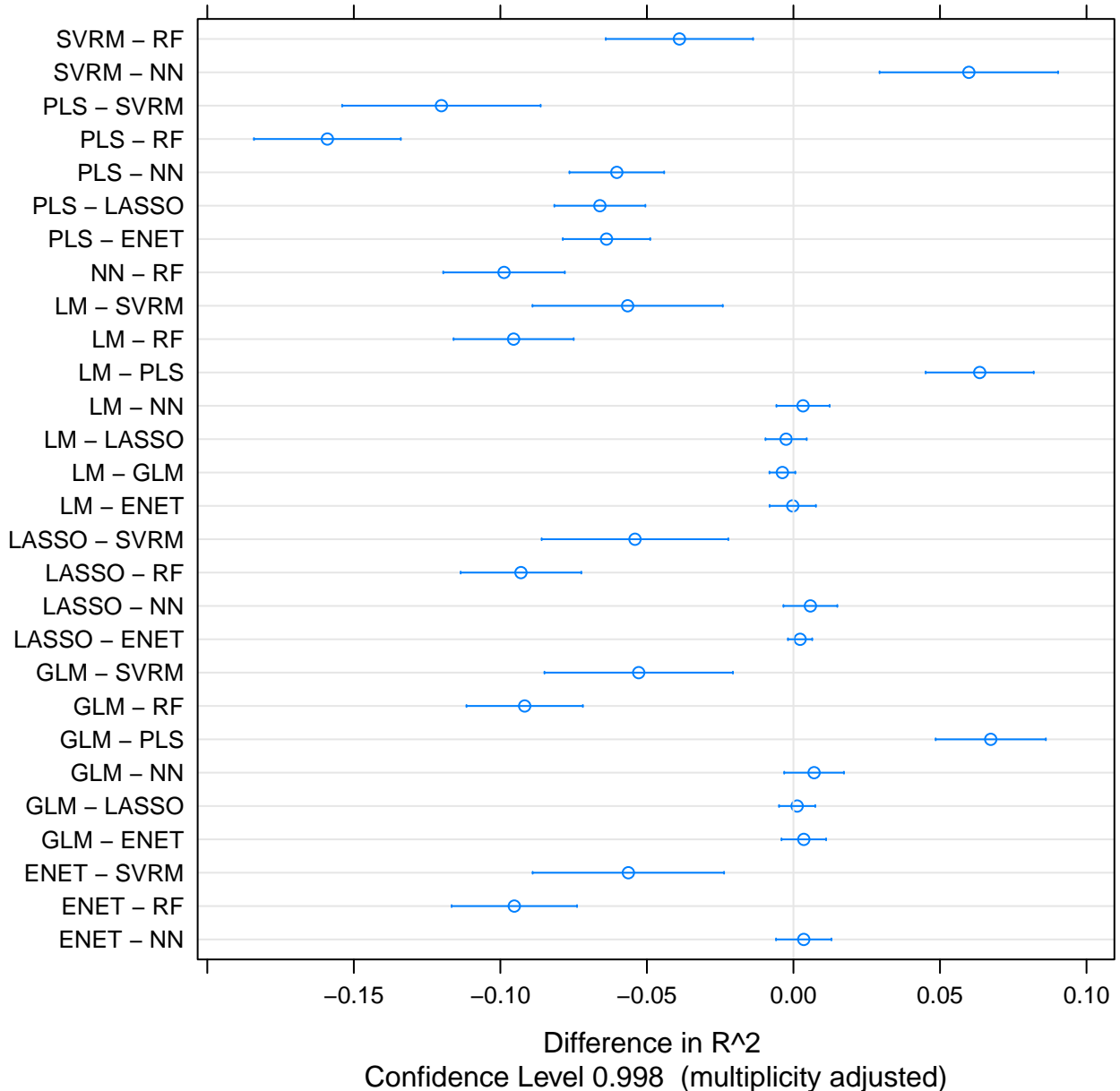

Supplement: Data S1 — Datailed results from UC Irvine Machine Learning Repository (Housing, Machine CPU, Wine Quality, Automobile and Parkinson) and the 3 Use Cases (Protein Corona, Gajewicz Metal Oxides and Aquatic Toxicity) [file peerj-04-2721-s001.zip › 4_automobile/DifModels.R2.iSplits.8.pdf]

# Models' differences on the training set (data split 9)

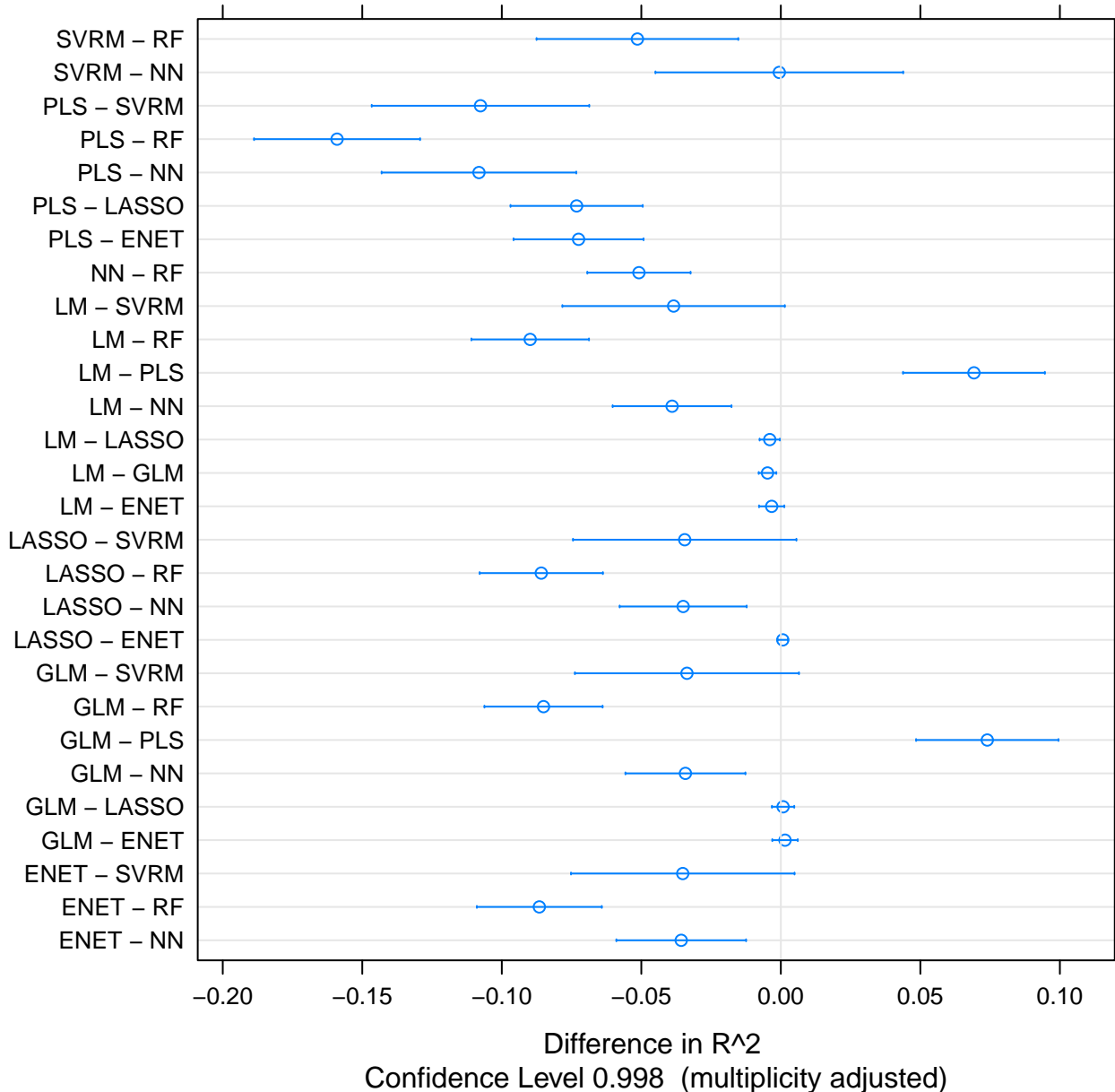

Supplement: Data S1 — Datailed results from UC Irvine Machine Learning Repository (Housing, Machine CPU, Wine Quality, Automobile and Parkinson) and the 3 Use Cases (Protein Corona, Gajewicz Metal Oxides and Aquatic Toxicity) [file peerj-04-2721-s001.zip › 4_automobile/DifModels.R2.iSplits.9.pdf]
